# Silanediol versus chlorosilanol: hydrolyses and hydrogen-bonding catalyses with fenchole-based silanes

**DOI:** 10.3762/bjoc.15.17

**Published:** 2019-01-18

**Authors:** Falco Fox, Jörg M Neudörfl, Bernd Goldfuss

**Affiliations:** 1 Department für Chemie, Institut für Organische Chemie, Greinstrasse 4, 50939 Köln, Germany; 2 Department für Chemie, Institut für Organische Chemie, Greinstrasse 6, 50939 Köln, Germany

**Keywords:** hydrogen bonds, hydrolysis, ion pairs, organocatalysis, silanediol

## Abstract

Biphenyl-2,2’-bisfenchyloxydichlorosilane (**7**, BIFOXSiCl_2_) is synthesized and employed as precursor for the new silanols biphenyl-2,2’-bisfenchyloxychlorosilanol (**8**, BIFOXSiCl(OH)) and biphenyl-2,2’-bisfenchyloxysilanediol (**9**, BIFOXSi(OH)_2_). BIFOXSiCl_2_ (**7**) shows a remarkable stability against hydrolysis, yielding silanediol **9** under enforced conditions. A kinetic study for the hydrolysis of dichlorosilane **7** shows a 263 times slower reaction compared to reference bis-(2,4,6-tri-*tert*-butylphenoxy)dichlorosilane (**14**), known for its low hydrolytic reactivity. Computational analyses explain the slow hydrolyses of BIFOXSiCl_2_ (**7**) to BIFOXSiCl(OH) (**8**, *E*_a_ = 32.6 kcal mol^−1^) and BIFOXSiCl(OH) (**8**) to BIFOXSi(OH)_2_ (**9**, *E*_a_ = 31.4 kcal mol^−1^) with high activation barriers, enforced by endo fenchone units. Crystal structure analyses of silanediol **9** with acetone show shorter hydrogen bonds between the Si–OH groups and the oxygen of the bound acetone (OH···O 1.88(3)–2.05(2) Å) than with chlorosilanol **8** (OH···2.16(0) Å). Due to its two hydroxy units, the silanediol **9** shows higher catalytic activity as hydrogen bond donor than chlorosilanol **8**, e.g., C–C coupling *N*-acyl Mannich reaction of silyl ketene acetals **11** with *N*-acylisoquinolinium ions (up to 85% yield and 12% ee), reaction of 1-chloroisochroman (**18**) and silyl ketene acetals **11** (up to 85% yield and 5% ee), reaction of chromen-4-one (**20**) and silyl ketene acetals **11** (up to 98% yield and 4% ee).

## Introduction

Silanediols are attractive target molecules due their hydrogen-bonding capabilities [1–6]. Two synthetic routes are available for syntheses of organosilanediols: If diphenylsilanes are used as building blocks, this route is well suited for syntheses of silanediols with electrophilic functions. In this case, the phenyl groups at the silicon atom are converted by acids (e.g., TFA or TfOH) and following aqueous work-up into silanediols [2,4,7–11]. Another route employs dichlorosilanes, which hydrolyze directly with nucleophiles (e.g., water [12–17] or hydroxide [18–22]) to the corresponding silanediols. While hydrolyses of dichlorosilanes have been studied extensively [23–25], hydrolyses of alkoxy dichlorosilanes are much less explored.

**Figure 1:**
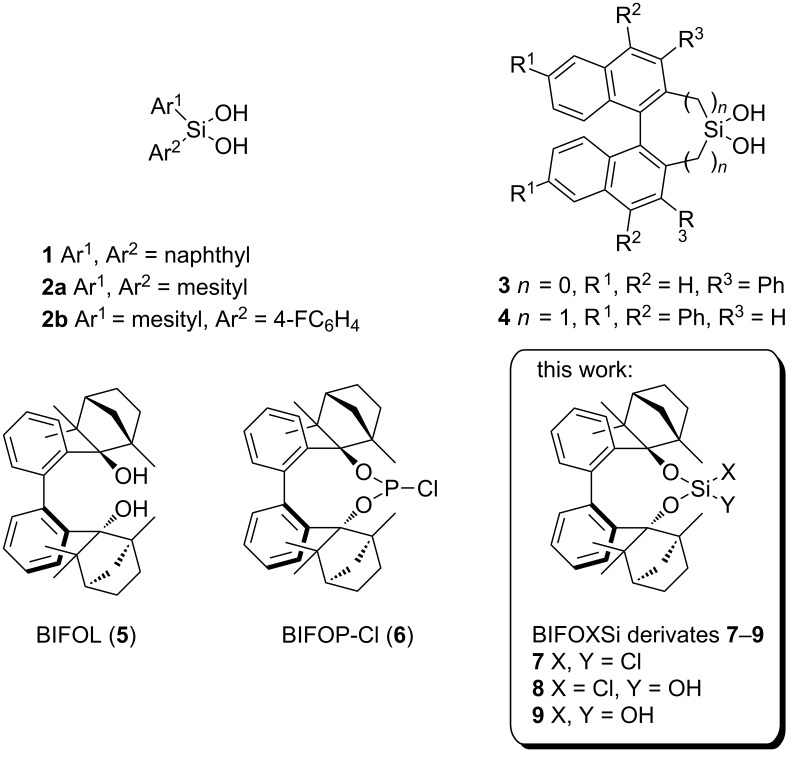
Hydrogen-bonding silanediols, i.e., di(1-naphthyl)silanediol (**1**) [39], silanediols **2** [41–43], binaphthylsilanediol derivatives **3** [45–46] and **4** [47–48] and novel biphenyl-2,2’-bisfenchyloxydichlorosilane (**7**), biphenyl-2,2’-bisfenchyloxychlorohydroxysilane (**8**) and biphenyl-2,2’-bisfenchyloxysilanediol (**9**) with precursor BIFOL (**5**) [52] and phosphite derivative BIFOP-Cl (**6**) [53].

**Scheme 1:**
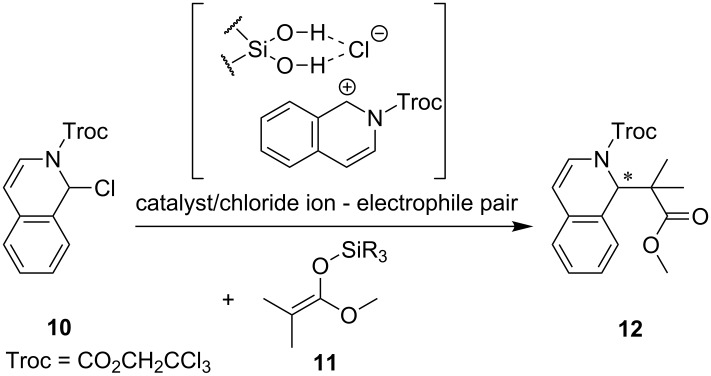
Hydrogen-bond-catalyzed *N*-acyl Mannich reaction of in situ-generated isoquinolin derivative **10** with different silyl ketene acetals **11** yielding C–C coupling product **12** [45,47].

Hydrogen bond donor (HBD) catalysis is an emerging field in organic synthesis [26–28], employing, e.g., squaramides [29], (thio)ureas [30–31] and phosphoric acid derivatives [27]. Cyclodiphosph(V)azanes [32–35] and silanediols [1,28,36] are two new hydrogen bonding scaffolds for anion recognition [37] and ion-pair catalysis [6,38]. Since Kondo et al. established silanediol **1** [39] as HBD for anion recognition in 2006, new chiral and achiral silanediols with organosilicon units have been developed by the groups of Franz (**2**) [40–44] and Mattson (**3**, **4**) [45–49] (Figure 1, Scheme 1). While these new catalysts have been proven to be potent HBD catalysts, the syntheses are challenging [47]. Compared to these stable carbon-connected silanediols, the readily accessible alkoxy silanediols undergo fast condensation reactions which often lead to unknown and insoluble polysiloxanes [50–51].

Previously, our group reports syntheses and applications of symmetric biphenyl-2,2`-bisfenchol (**5**, BIFOL, Figure 1) [52,54] and it´s derivative, the chiral chlorophosphite ligand **6** (BIFOP-Cl, Figure 1), e.g., in Cu-catalyzed 1,4-additions [53], in Pd-catalyzed alkyl–aryl cross coupling reactions [55–56], as well as for organoaluminum fencholate reagents [57]. Unexpected stability against hydrolysis [58] makes BIFOL (**5**) a potentially promising chiral backbone for new organo silicates, e.g., silanediol **9** (Figure 1). As a silicic acid ester, silanediol **9** should show increased acidity in comparison to C–Si(OH)_2_ derivates, e.g., **1**–**4** [40]. In this work the syntheses of BIFOXSiCl_2_ (**7**), BIFOXSi(OH)_2_ (**9**) and BIFOXSiCl(OH) (**8**) are described. The hydrolytic stability of dichlorosilane **7** is investigated in a kinetics study and is compared to analogue dichlorosilanes, i.e., **13** and **14** (Scheme 4). UV–vis titration experiments and catalyses are carried out with chlorosilanol **8** and silanediol **9**, to assess catalytic and anion binding characteristics.

## Results and Discussion

**Scheme 2:**
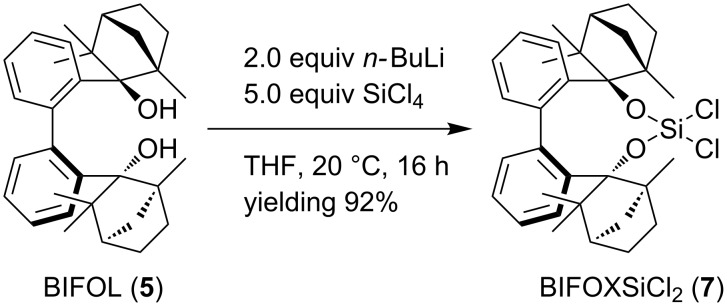
Synthesis of BIFOXSiCl_2_, starting with BIFOL (**5**) [52,54] yielding dichlorosilane **7**.

Enantiopure dichlorosilane **7** is readily accessible by lithiation of BIFOL (**5**) [28,52,54,59] and subsequent reaction with tetrachlorosilane (92% yield, Scheme 2).

**Scheme 3:**
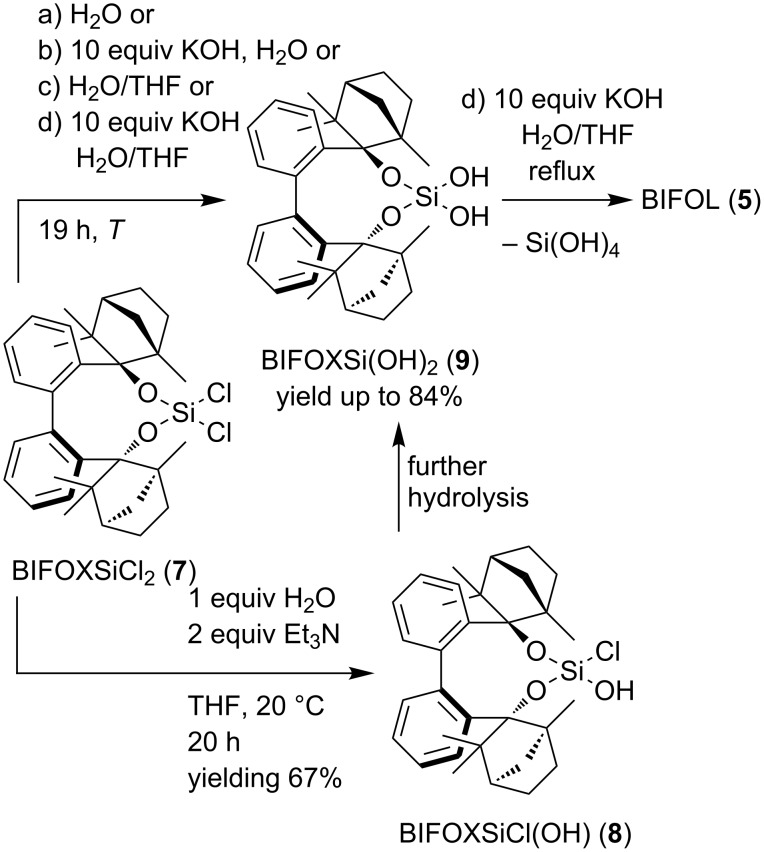
Hydrolysis of BIFOXSiCl_2_ (**7**) yielding the corresponding silanediol **9** and controlled hydrolysis of BIFOXSiCl_2_ (**7**) to BIFOXSiCl(OH) (**8**). For solvent mixtures and temperatures see Table 1.

Unlike the hydrolysis of BIFOP-Cl (**6**) to BIFOP-OH, the dichlorosilane **7** is not hydrolyzed by aqueous potassium hydroxide solution [53]. The heterolytic reaction of solid BIFOXSiCl_2_ (**7**) in an aqueous KOH solution is negligible (<1% yield, Table 1, Scheme 3 in a) H_2_O and b) H_2_O/KOH). The reluctance against hydrolysis of BIFOXSiCl_2_ (**7**) can be explained by the hydrophobic aryl backbone and the fenchyl groups, which result in a decrease of the solubility of BIFOXSiCl_2_ (**7**) in water. Thus, a H_2_O/THF mixture is used to increase solubility and yields (Table 1, Scheme 3c). While the solubility of BIFOXSiCl_2_ (**7**) in H_2_O/THF greatly increases (clear solution), potassium hydroxide is needed as a strong nucleophile to yield BIFOXSi(OH)_2_ (**9**) at 20 °C (14% yield, Table 1, Scheme 3d). By increasing the temperature to 50 °C the hydrolysis increases, resulting in 32% yield in H_2_O/THF and 64% yield in H_2_O/THF/KOH (Table 1, Scheme 3).

**Table 1:** Hydrolysis of BIFOXSiCl_2_ (**7**) to BIFOXSi(OH)_2_ (**9**) (Scheme 3) in different solvent mixtures, with or without KOH at different temperatures.

yield^a^ [%] in solvent:				

	a)	b)	c)	d)

T	H_2_O	H_2_O/KOH	H_2_O/THF	H_2_O/THF/KOH

20 °C	<1	<1	<1	14
50 °C	<1	<1	32	64
reflux^b^	<1	<1	84	53

^a^Isolated yields, reaction conditions: 0.09 mmol **7**, 2.5 mL solvent, 0.9 mmol KOH, H_2_O/THF 1:1. ^b^reflux conditions are: H_2_O ≈ 100 °C, H_2_O/KOH ≈ 107 °C, H_2_O/THF ≈ 77 °C, H_2_O/THF/KOH ≈ 80 °C.

At reflux conditions in H_2_O/THF, but without potassium hydroxide, BIFOXSi(OH)_2_ (**9**) is isolated in 89% yield, while with KOH, just 53% yield is achieved (Table 1). The lower yield of silanediol **9**, at H_2_O/THF/KOH reflux conditions, can be explained by the further hydrolysis to BIFOL (**5**, Scheme 3). Under the conditions described in Table 1, the monohydroxy compound BIFOXSiCl(OH) (**8**) cannot be isolated as an intermediate. For the synthesis of chlorosilanol **8** optimized reaction conditions are necessary. Here, 1 equiv of water and 2 equiv of triethylamine (relative to dichlorosilane **7**) are added to a THF solution at 20 °C. BIFOXSiCl(OH) (**8**) is isolated in 67% yield (Scheme 3).

### Hydrolysis studies

**Scheme 4:**
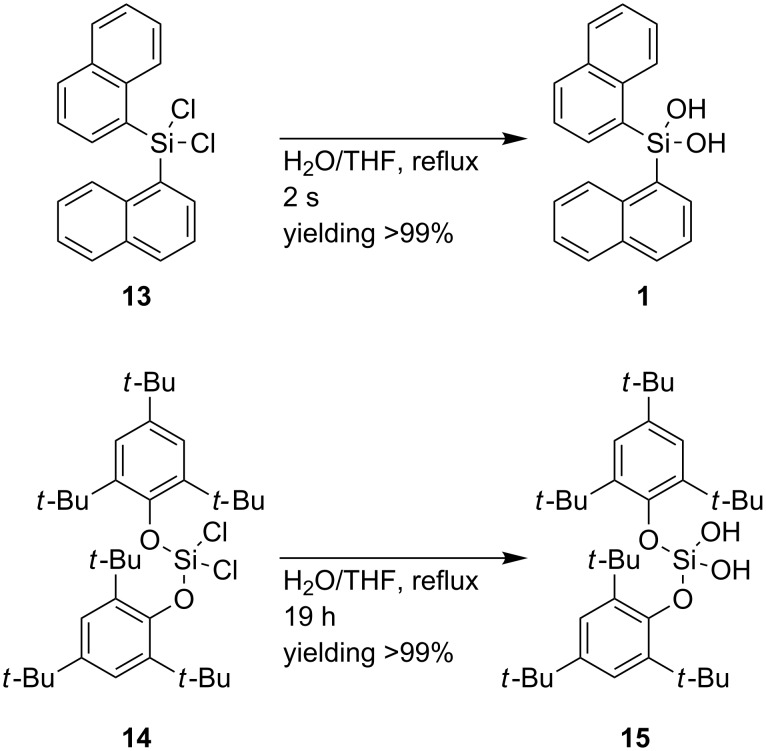
Hydrolysis of dichlorosilanes **13** and **14** to their corresponding silanediols **1** and **15** [51,60].

To assess the stability of dichlorosilane **7**, its hydrolysis relative to established silanediol motifs, i.e., di-*tert-*butoxydichlorosilane (((CH_3_)_3_CO)_2_SiCl_2_) [50], di(1-naphthyl)dichlorosilane (**13**) [39] and bis(2,4,6-tri-*tert-*butylphenoxy)dichlorosilane (**14**) [60–61] is examined (Scheme 4). In close analogy to BIFOXSiCl_2_ (**7**), di-*tert-*butoxydichlorosilane is substituted with tertiary alkoxy groups. While the close analogy of BIFOXSiCl_2_ (**7**) and ((CH_3_)_3_CO)_2_SiCl_2_ would make a comparison of these two dichlorosilanes preferable, the instability of the latter against hydrolyses and temperature resulting in further condensation products [50–51], leaves no comparison possible.

**Figure 2:**
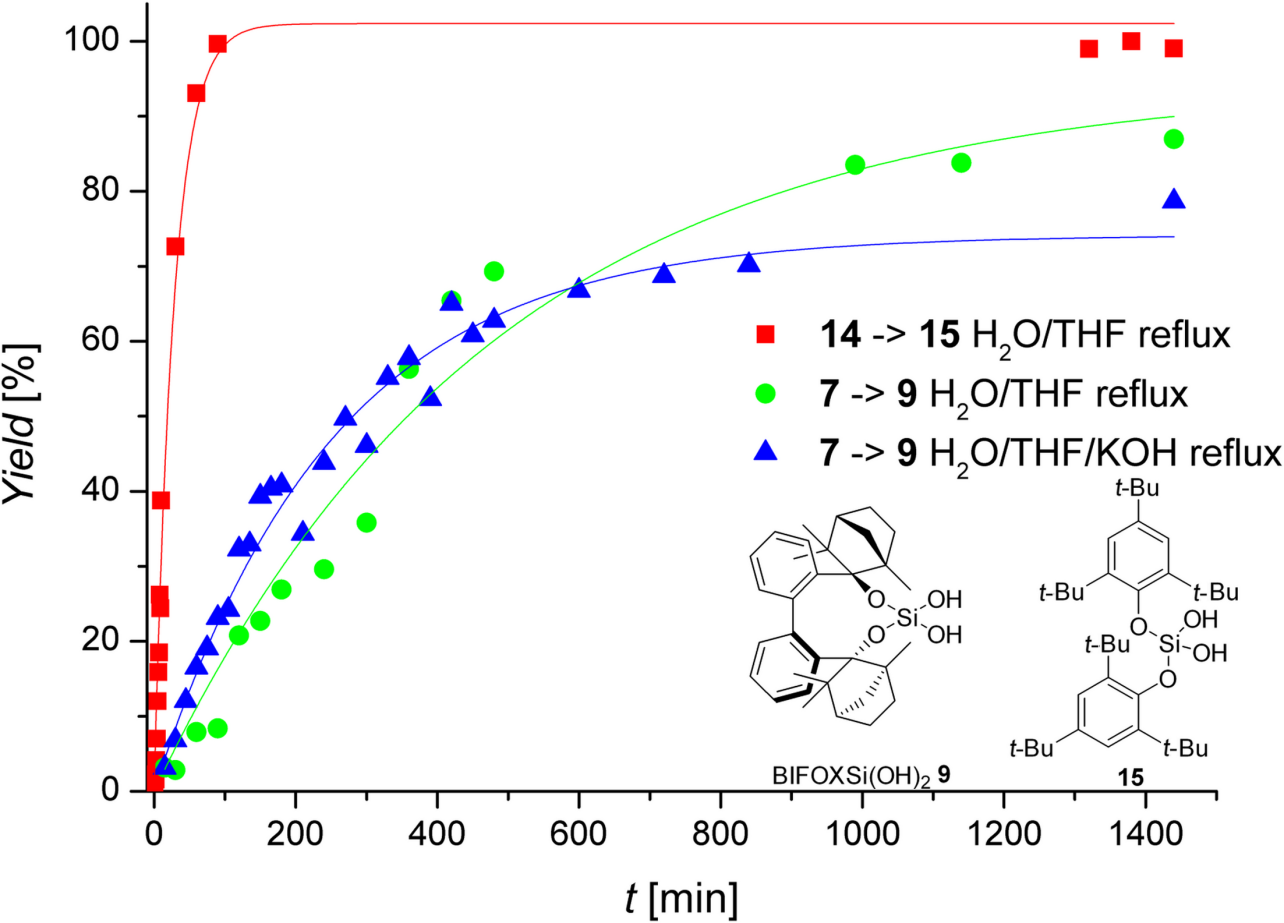
Hydrolyses of dichlorosilane **7** and **14** to BIFOXSi(OH)_2_ (**9**, green circle) and bis(2,4,6-tri-*tert-*butylphenoxy)silanediol (**15**, red square) in H_2_O/THF reflux and BIFOXSi(OH)_2_ (**9**, blue triangle) in H_2_O/THF/KOH reflux conditions. Isolated yields are plotted against reaction time (Table 1, Scheme 3 and Scheme 4). Reflux conditions are: H_2_O/THF ≈ 77 °C, H_2_O/THF/KOH ≈ 80 °C.

At H_2_O/THF reflux conditions, the hydrolysis of dichlorosilane **13** yields silanediol **1** with >99% yield, after a reaction time of two seconds (Scheme 4, addition of H_2_O, with instant extraction with Et_2_O). The stability of dichlorosilane **14**, which has been previously reported by Spirk et al. [60], has been found to be higher than that of dichlorosilane **13** under the same conditions (H_2_O/THF reflux) resulting in >99% yield after 100 min (Figure 2, red squares). The hydrolysis of dichlorosilanes **7** and **14** to the corresponding silanediols **9** (Scheme 3) and **15** (Scheme 4) is investigated further (Figure 2). The conversion of dichlorosilane **14** to silanediol **15** is completed (yield >99%) after 100 minutes at H_2_O/THF reflux conditions (Figure 2, red square). The hydrolysis of BIFOXSiCl_2_ (**7**) on the other hand is slower at the same conditions (24 h for 84% yield of BIFOXSi(OH)_2_ (**9**, Figure 2, green circle). With H_2_O/THF/KOH reflux conditions the initial reaction is found to be slightly faster, but resulting in a saturation at a lower yield of BIFOXSi(OH)_2_ (**9**, Figure 2, blue triangle, 70% yield), caused by a starting decomposition of BIFOXSi(OH)_2_ (**9**) to BIFOL (**5**) and Si(OH)_4_ (Scheme 3). To quantify the reactivity of the dichlorosilanes **7** and **14**, a higher concentration of water (solvent) or hydroxide (10 equiv) is used (Table 2), resulting in a pseudo first order reaction.

**Table 2:** Hydrolyses of dichlorosilanes **7** and **14** to the corresponding silanediols **9** and **15** (Figure 3, Scheme 3 and Scheme 4, absolute reaction constant *k* and relative reaction constant *k*_rel_).

reaction	*k*^a^ [min^−1^]	*k*_rel_^b^ [min^−1^]

**14** H_2_O/THF reflux^c^	0.848	263
**7** H_2_O/THF/KOH reflux^c^	0.005	1.5
**7** H_2_O/THF reflux^c^	0.003	1

^a^*k* is determined with *ln* ([A_0_]/[A_t_]) = k _*_ t and plotted in Figure 3. ^b^*k*_rel_ is normalized on the slowest hydrolysis reaction of BIFOXSiCl_2_ (**7**) in H_2_O/THF reflux conditions (Scheme 2, Scheme 3). ^c^Reflux conditions are: H_2_O/THF ≈ 77 °C, H_2_O/THF/KOH ≈ 80 °C.

**Figure 3:**
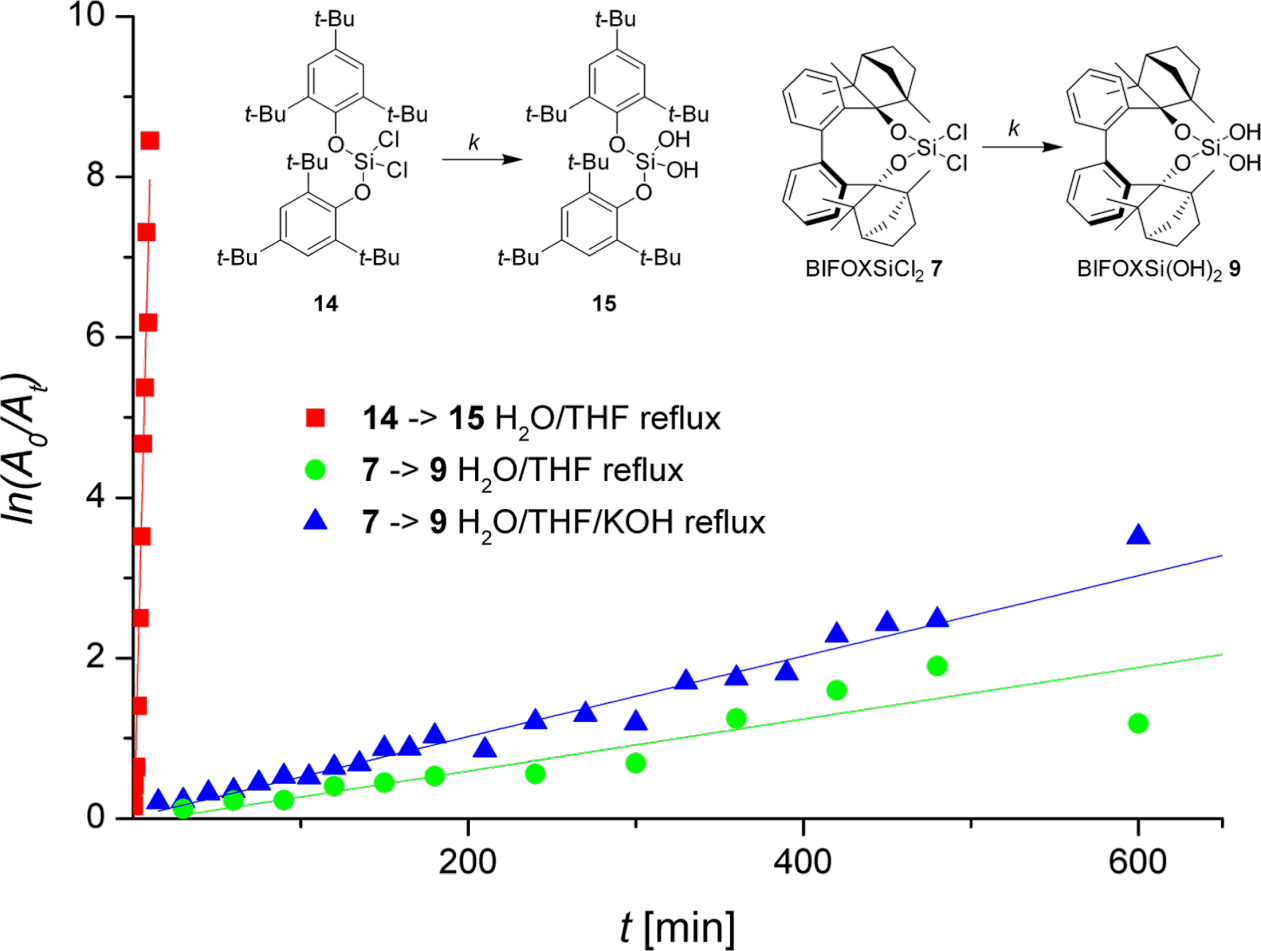
Hydrolyses of BIFOXSiCl_2_ (**7**) to BIFOXSi(OH)_2_ (**9**, green circle), bis(2,4,6-tri-*tert*-butylphenoxy)dichlorosilane (**14**) to bis(2,4,6-tri-*tert*-butylphenoxy)silanediol (**15**, red square) in H_2_O/THF reflux and BIFOXSiCl_2_ (**7**) to BIFOXSi(OH)_2_ (**9**, blue triangle) H_2_O/THF/KOH reflux conditions (Scheme 3 and Scheme 4). Reaction constants are evaluated with ln ([*A*_0_]/[*A*_t_]) = *k·t* for a pseudo-first-order reaction plotted against reaction time (Table 2). Reflux conditions are: H_2_O/THF ≈ 77 °C, H_2_O/THF/KOH ≈ 80 °C.

To remove the influence of the decomposition of silanediol **9**, just the reaction time from 0 to 600 min is considered. Compared to established dichlorosilanes, the observed stability of BIFOXSiCl_2_ (**7**) is clearly apparent from those studies (Figure 2 and Figure 3). Dichlorosilane **14**, which is known to show a comparably high resistancy against hydrolysis [60], exhibits a much faster hydrolysis reaction (*k*_rel_ = 263 min^−1^, Table 2) at H_2_O/THF reflux conditions than BIFOXSiCl_2_ (**7**). With KOH, the reaction rate of the hydrolysis of BIFOXSiCl_2_ (**7**) is just slightly increased (*k*_rel_ = 1.5 min^−1^, Table 2).

### Computational analyses

**Scheme 5:**
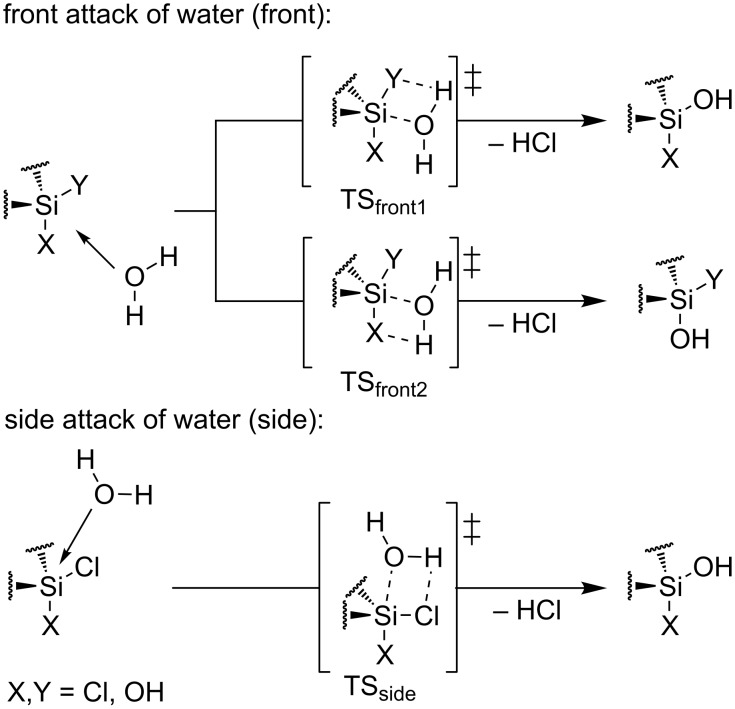
Two investigated pathways for the hydrolysis of the dichlorosilanes. Front attack mechanism (front) between the two chloro substituents or side attack mechanism (side) between one chloro and both additional substituents (TS = transition structure) [62–68].

Nucleophilic substitution at silicon is already discussed with S_N_2 mechanism, following a backside attack opposite of the leaving group, as well as a front side attack near the leaving group [62–68]. A backside attack at the silicon in dichlorosilane **7** and monochlorosilanol **8** is blocked by the backbone, making a consideration of the mechanism not necessary. A mechanism with a pentacoordination at the silicon is assumed for the hydrolyses of BIFOXSiCl_2_ (**7**) to BIFOXSiCl(OH) (**8**) as intermediate and BIFOXSI(OH)_2_ (**9**) as product [65–67]. Two pathways (front attack mechanism (front) or side attack mechanism (side)) for the approaching water molecule are considered (Scheme 5).

**Figure 4:**
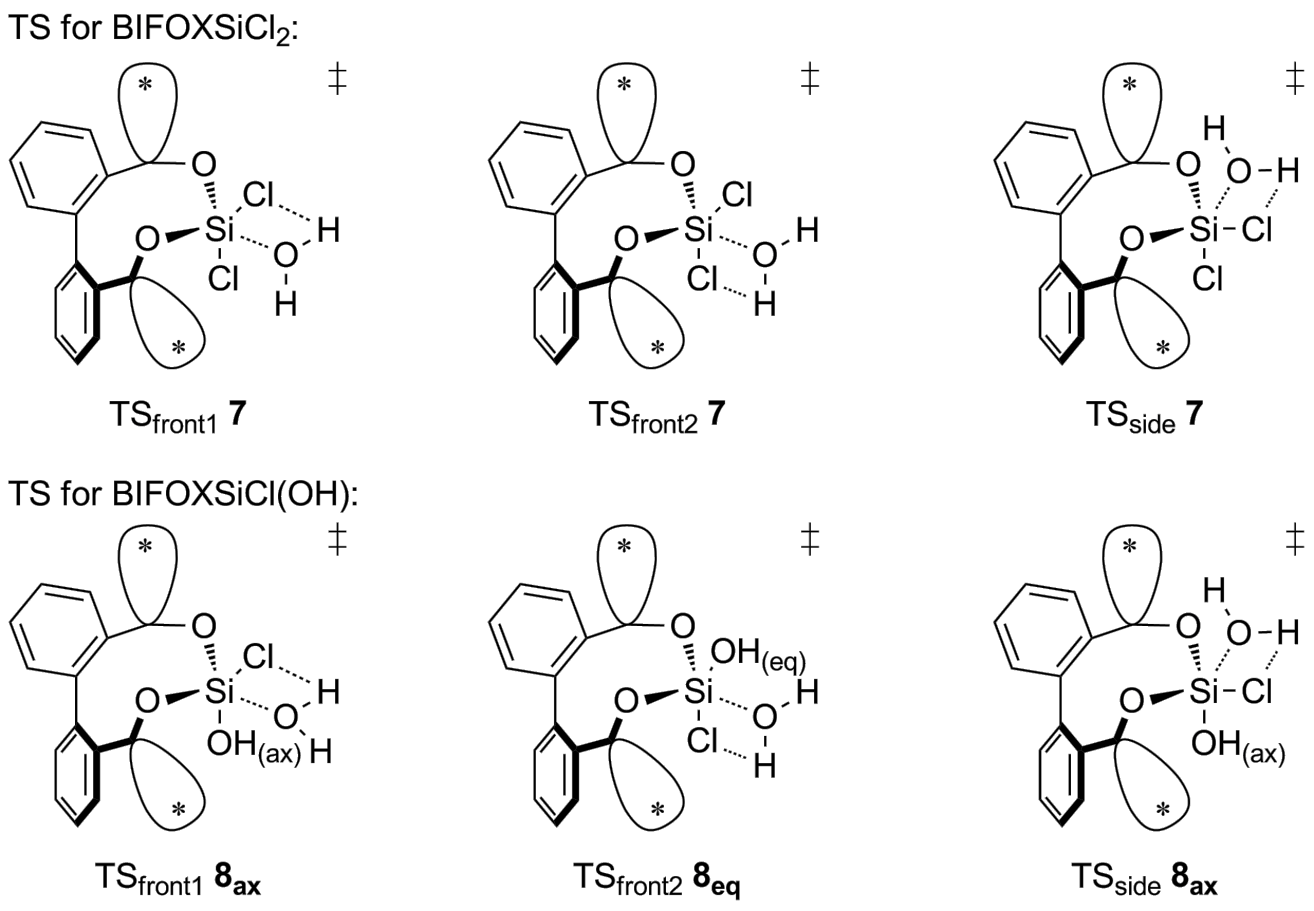
Three transition structures each, for the hydrolysis of BIFOXSiCl_2_ (**7**) and BIFOXSiCl(OH) (**8**) considering two possible configuration isomers of BIFOXSiCl(OH) (**8**). For **8_ax_** the OH group is parallel situated to the biaryl axis. For **8_eq_** the OH group is orthogonal oriented to the biaryl axis. The fenchyl groups are abbreviated with (*) for more clarity.

In both, front attack and side attack, the attacking water molecule is in plane with the Cl–Si–Cl unit for the first hydrolysis step. For the second hydrolysis step, analogue pathways are considered. These trajectories lead to three transition structures each, for the hydrolysis of BIFOXSiCl_2_ (**7**) and BIFOXSiCl(OH) (**8**, Figure 4).

**Figure 5:**
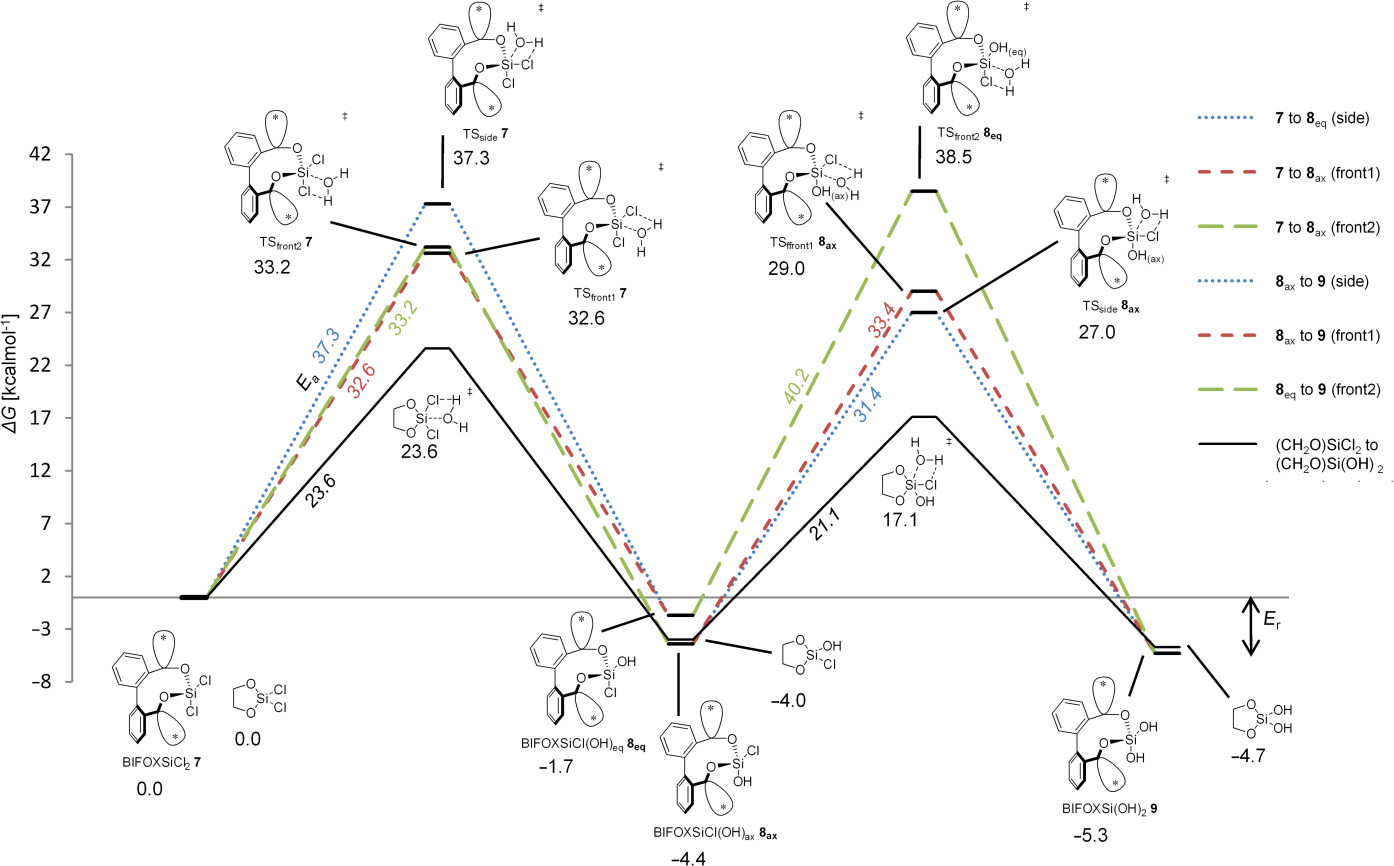
Computed hydrolyses of BIFOXSiCl_2_ (**7**) to BIFOXSiCl(OH) **8_ax_** and BIFOXSiCl(OH) **8_eq_** and subsequent computed hydrolysis to BIFOXSi(OH)_2_ (**9**) and comparison with glycoxydichlorosilane. The activation energy (*E*_a_) is the difference of the free Gibbs energy of the educt and the TS and the reaction energy (*E*_r_) is the difference the free Gibbs energies of the educts and products of the respective steps side and front1 resulting in BIFOXSiCl(OH) **8_eq_**, front2 results in BIFOXSiCl(OH) **8_ax_**. From BIFOXSiCl(OH) **8_eq_** only front2 is a possible path to BIFOXSi(OH)_2_ (**9**). From BIFOXSiCl(OH) **8_ax_** front1 and side are possible paths to BIFOXSi(OH)_2_ (**9**, Table 3, Figure 4, Scheme 5). Reaction energies are (*E*_r_) in kcal mol^−1^, activation energies are (*E*_a_) in kcal mol^−1^ and italic.

Geometry optimizations and frequency computations are performed in gas phase with B3LYP-D3BJ/6-31G(d) at 298 K. For single point energies, M06-2X-D3/6-311++G(d,p) in the solvent THF with the PCM model is used [69–70]. The free Gibbs energies of the respective structures are discussed. The activation energy (*E*_a_) is the difference between the educt and the TS and the reaction energy (*E*_r_) is the difference between the educts and products of the respective steps. The mechanism of hydrolysis, only one molecule of water per hydrolysis step is considered. Additional interactions by THF and water are only considered by the PCM model. Starting with BIFOXSiCl_2_ (**7**), the side and front1 attack mechanism are resulting in BIFOXSiCl(OH) **8_eq_**. The front2 attack mechanism results in BIFOXSiCl(OH) **8_ax_** (Figure 5).

**Figure 6:**
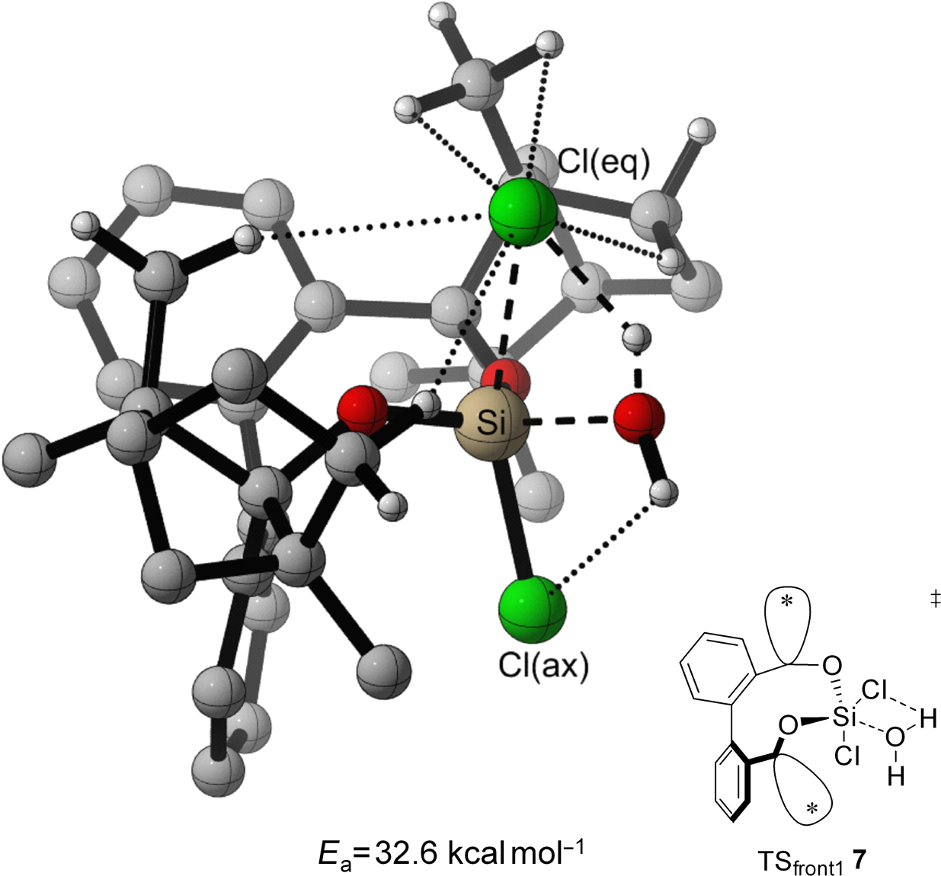
Transition state leading to **8_eq_** following front1 attack (*E*_a_ = 32.6 kcal mol^−1^, Figure 5, Table 3, entry 1). Breaking and forming bonds in dashed lines, additional C–H-interactions with dotted lines (M06-2X-D3/6-311++G(d,p)(PCM=THF)//B3LYP-D3BJ/6-31G(d) at 298 K).

**Figure 7:**
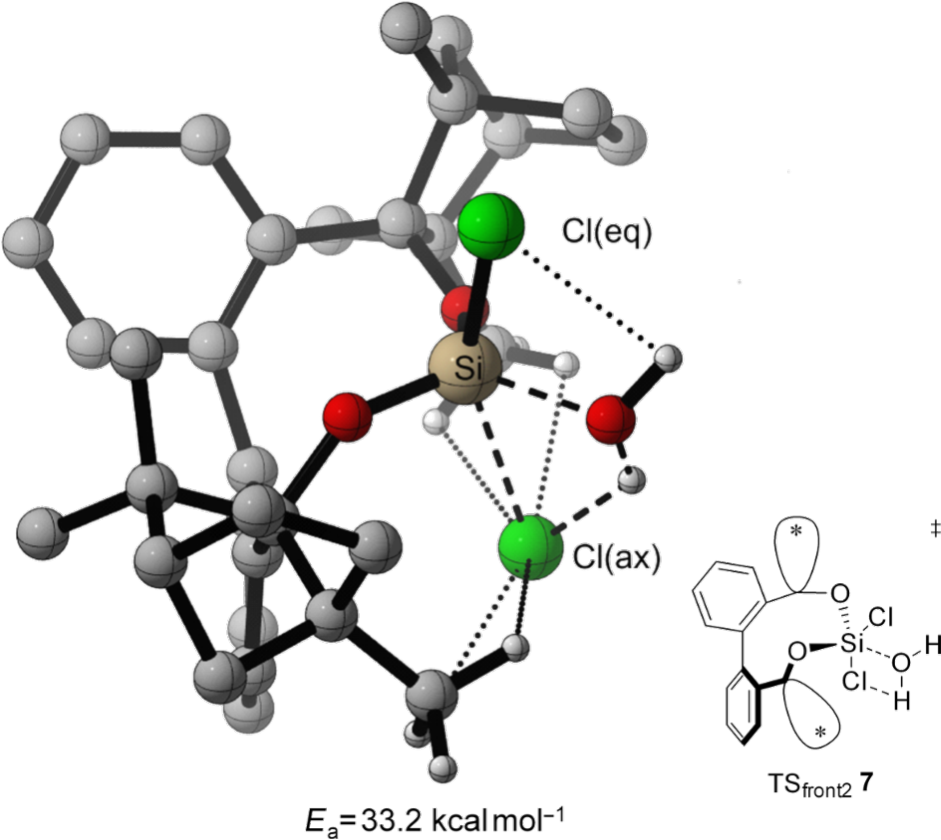
Transition state leading to **8_ax_** following front2 attack (*E*_a_ = 33.2 kcal mol^−1^, Figure 5, Table 3, entry 2). Breaking and forming bonds in dashed lines, additional C–H-interactions with dotted lines (M06-2X-D3/6-311++G(d,p)(PCM=THF)//B3LYP-D3BJ/6-31G(d) at 298 K).

**Figure 8:**
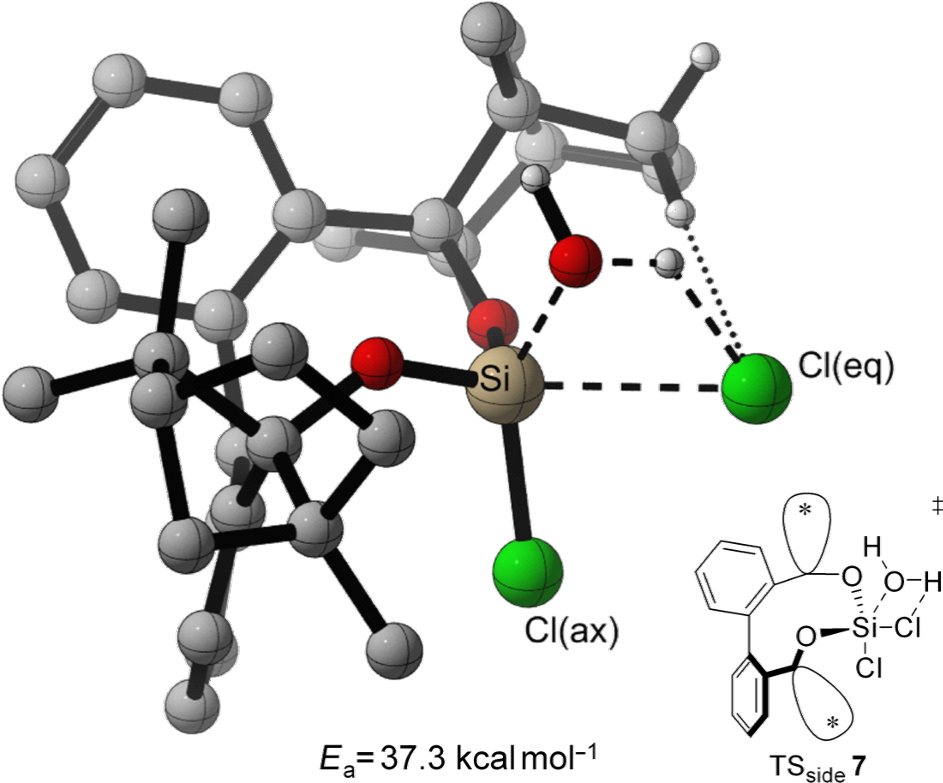
Transition state leading to **8_eq_** following side attack (*E*_a_ = 37.4 kcal mol^−1^, Figure 5, Table 3, entry 3). Breaking and forming bonds in dashed lines, additional C–H-interactions with dotted lines (M06-2X-D3/6-311++G(d,p)(PCM=THF)//B3LYP-D3BJ/6-31G(d) at 298 K).

**Figure 9:**
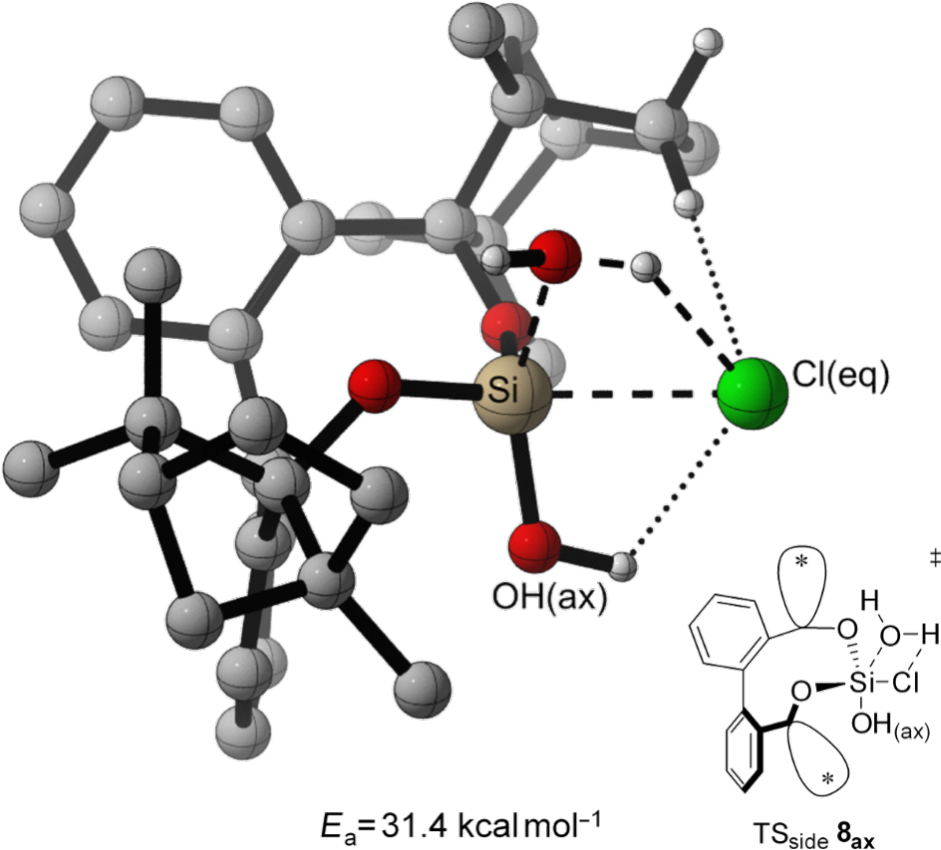
Transition state leading to **9** following side attack (*E*_a_ = 31.4 kcal mol^−1^, Figure 5, Table 3, entry 6). Breaking and forming bonds in dashed lines, additional C–H-interactions with dotted lines (M06-2X-D3/6-311++G(d,p)(PCM=THF)//B3LYP-D3BJ/6-31G(d) at 298 K).

**Figure 10:**
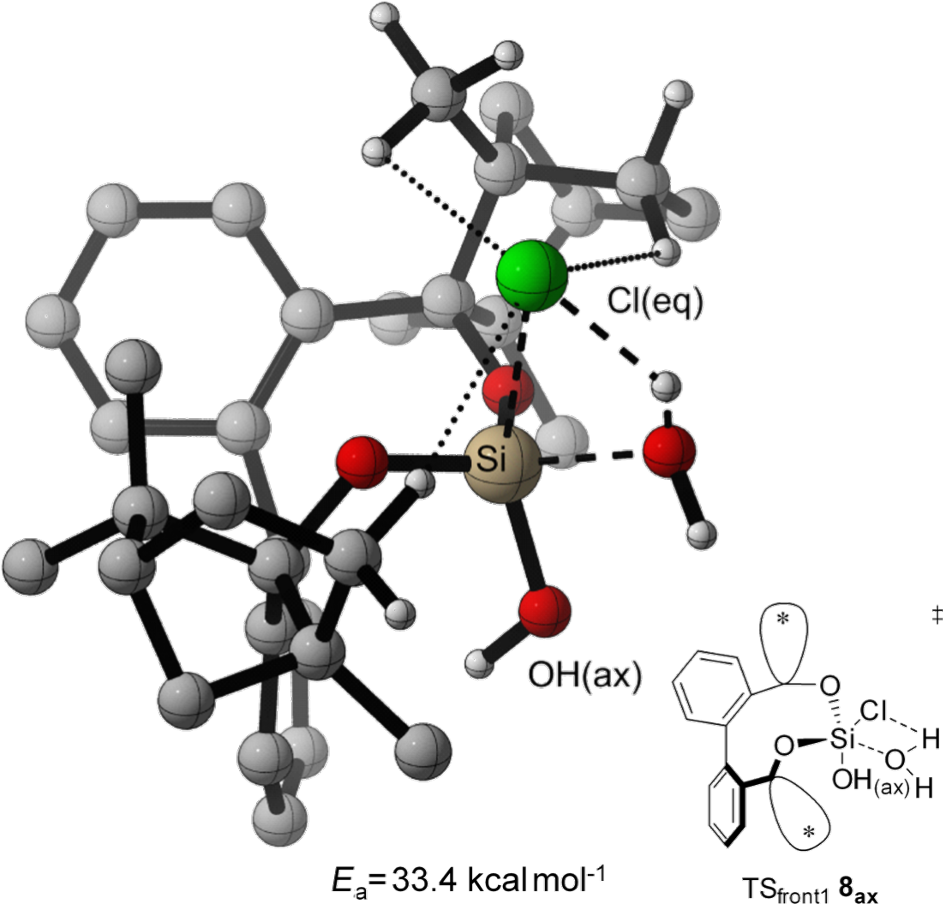
Transition state leading to **9** following front1 attack (*E*_a_ = 33.4 kcal mol^−1^, Figure 5, Table 3, entry 4). Breaking and forming bonds in dashed lines, additional C–H-interactions with dotted lines (M06-2X-D3/6-311++G(d,p)(PCM=THF)//B3LYP-D3BJ/6-31G(d) at 298 K).

For the TS of the front1 attack mechanism, the lowest activation energy (*E*_a_ = 32.6 kcal mol^−1^, Table 3, entry 1, Figure 5 and Figure 6) is found, closely followed by the front2 attack mechanism (*E*_a_ = 33.2 kcal mol^−1^, Table 3, entry 2, Figure 5 and Figure 7). The side attack mechanism leads to the highest TS for the first hydrolysis step of BIFOXSiCl_2_ (**7**) to BIFOXSiCl(OH) **8_eq_** (*E*_a_ = 37.3 kcal mol^−1^, Table 3, entry 3, Figure 5 and Figure 8). BIFOXSiCl(OH) **8_ax_** is found to be the more stable isomer with a reaction energy *E*_r_ of −4.4 kcal mol^−1^, compared to BIFOXSiCl(OH) **8_eq_** with a *E*_r_ of −1.7 kcal mol^−1^ (Δ*E*_r_ = 2.7 kcal mol^−1^, Table 3, entries 1 and 2, Figure 5), as it is found as the only isomere in crystal structure analysis (Figure 13). For the second hydrolysis step from BIFOXSiCl(OH) **8_ax_** to BIFOXSi(OH)_2_ (**9**), the side attack mechanism leads to the lowest TS (*E*_a_ = 31.4 kcal mol^−1^, Table 3, entry 6, Figure 5, Figure 9), followed by the front1 attack mechanism (*E*_a_ = 33.4 kcal mol^−1^, Table 3, entry 4, Figure 5, Figure 10) leading to product BIFOXSi(OH)_2_ (**9**).

**Table 3:** Computed^a^ activation energies and reaction energies (*E*_a_ and *E*_r_ [kcal mol^−1^]) and imaginary frequencies (ν [cm^−1^]) of the transition structure (TS) for the hydrolysis of dichlorosilane **7**, **8**, **13**, [CH_2_O]_2_SiCl_2_ and SiCl_4_ to the corresponding mono- and diols.

entry	reaction	TS	ν	*E*_a_	*E*_r_

1^b^	**7** to **8_eq_**	**TS_front1_** **7**	**–206.23**	**32.6**	**−1.7**
2	**7** to **8_ax_**	TS_front2_ **7**	–221.44	33.2	−4.4
3	**7** to **8_eq_**	TS_side_ **7**	–189.66	37.3	−1.7
4	**8_ax_** to **9**	TS_front1_ **8**_ax_	–208.01	33.4	−5.3
5	**8_eq_** to **9**	TS_front2_ **8**_eq_	–242.41	40.2	−5.3
6^b^	**8_ax_** to **9**	**TS_side_ 8_ax_**	**–162.28**	**31.4**	**−5.3**
7^b^	**13** to **13_ClOH_**	**TS_front_ 13**	**–167.52**	**27.7**	**−1.1**
8	**13** to **13_ClOH_**	TS_side_ **13**	–176.02	35.4	−1.1
9^b^	**13_ClOH_** to **1**	**TS_front_ 13_ClOH_**	**–137.30**	**29.1**	**1.5**
10	**13_ClOH_** to **1**	TS_side_ 13_ClOH_	−119.75	29.9	1.5
11	[CH_2_O]_2_SiCl_2_ to [CH_2_O]_2_SiClOH		–542.44	23.6	−4.0
12	[CH_2_O]_2_SiClOH to [CH_2_O]_2_Si(OH)_2_		–189.64	21.1	−4.7
13	Cl_2_SiCl_2_ to Cl_2_SiClOH		–415.78	28.4	−5.4
14	Cl_2_SiClOH to Cl_2_Si(OH)_2_		–209.82	25.0	−9.3
15	(OH)_2_SiCl_2_ to (OH)_2_SiClOH		–145.49	20.9	−3.4
16	(OH)_2_SiClOH to (OH)_2_Si(OH)_2_		–128.55	20.8	−4.8

^a^M06-2X-D3/6-311++G(d.p)(PCM=THF)//B3LYP-D3BJ/6-31G(d) at 298 K. ^b^Favored reaction structures are bolted.

**Figure 11:**
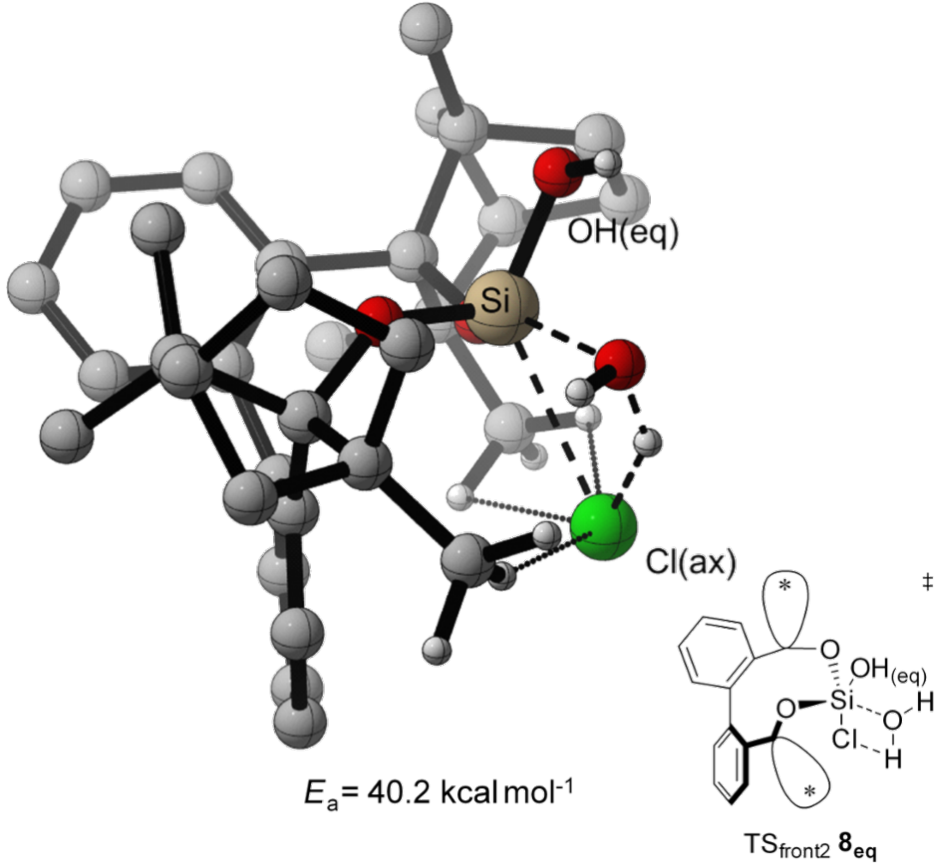
Transition state leading to **9** following front2 attack (*E*_a_ = 40.2 kcal mol^−1^, Figure 5, Table 3, entry 5). Breaking and forming bonds in dashed lines, additional C–H-interactions with dotted lines (M06-2X-D3/6-311++G(d,p)(PCM=THF)//B3LYP-D3BJ/6-31G(d) at 298 K).

From BIFOXSiCl(OH) **8_eq_** only the front attack mechanism TS_front2_ **8_eq_** is possible, which also leads to BIFOXSi(OH)_2_ (**9**), but with the highest *E*_a_ (40.2 kcal mol^−1^, Table 3, entry 5, Figure 5 and Figure 11). In accordance with the crystal structure analysis of BIFOXSiCl(OH) (**8**, Figure 13), it can be seen that the more stable isomer BIFOXSiCl(OH) **8_ax_** corresponds to the synthesized isomer. Considering the lowest *E*_a_ for both steps, the first hydrolysis step is the rate-determining step (**7** to **8_eq_**, TS_front1_ **7** *E*_a_ = 32.6 kcal mol^−1^ vs **8_ax_** to **9**, TS_side_ **8_ax_** *E*_a_ = 31.4 kcal mol^−1^, Table 3, entries 1 and 6, Figure 6 and Figure 9), which agrees with the experimental hydrolysis. Under H_2_O/THF reflux conditions, no BIFOXSiCl(OH) (**8**) has been isolated, but has to be synthesized separately (Scheme 3, Figure 2 and Figure 3). Both front attack TS have much lower energy, than the TS resulting by side attack mechanism, for the first hydrolysis step (TS_front2_ **7** *E*_a_ = 33.2 kcal mol^−1^, TS_front1_ **7** *E*_a_ = 32.6 kcal mol^−1^ vs TS_side_ **7** E_a_ = 37.3 kcal mol^−1^, Table 3, entries 1–3, Figures 6–8). Responsible for the lower *E*_a_ is an additional stabilization by an interaction of the remaining chloro atom to the attacking water (dotted line to the Cl(ax) Figure 6 and Cl(eq) Figure 7). The small energy difference for the TS_front1 _**7** and TS_front2_ **7** is to explained by additional C–H interactions between the fenchyl groups to the leaving chloride (four dotted lines in TS_front2_ **7**, Figure 7, five dotted lines in TS_front1_ **7**, Figure 6).

Through the approach of the attacking water molecule in the side attack mechanism, the chloro atoms are forced to get closer to each other leading to electrostatic repulsion (Figure 8). Stabilizing C–H interaction from the fenchyl group to the exiting chloride can be found as well (one dotted line in TS_side_ **7**, Figure 8).

At the second step, the side mechanism leads to a lower energy barrier (TS_side _**8_ax_** *E*_a_ = 31.4 kcal mol^−1^, Table 3, entry 6, Figure 9) than the front attack mechanisms (TS_front1 _**8_ax_** *E*_a_ = 33.4 kcal mol^−1^, TS_front2 _**8_eq _***E*_a_ = 40.2 kcal mol^−1^, Table 3, entries 4 and 5, Figure 10 and Figure 11). In the former mechanism the chloro atom comes closer to the already present hydroxy group (Figure 9).

A contact between the OH(ax) and the Cl(eq) is found, in addition to the C–H interaction (dotted line, Figure 9), which stabilized the leaving Cl ion with a weak hydrogen bond. In the front attack mechanisms for the second hydrolytic step only stabilizing C–H interactions from the fenchyl group to the chloro atom occur (dotted line, Figure 10 and Figure 11).

The highest energy barriers of the computed molecules are found for BIFOXSiCl_2_ for the first step (*E*_a_ = 32.6 kcal mol^−1^, *E*_a_ = 33.2 kcal mol^−1^, *E*_a_ = 37.3 kcal mol^−1^, Table 3, entries 1–3, Figures 6–8) and for BIFOXSiCl(OH) (**8**) for the second step (*E*_a_ = 33.4 kcal mol^−1^, *E*_a_ = 31.4 kcal mol^−1^, *E*_a_ = 40.2 kcal mol^−1^, Table 3, entries 4–6, Figures 9–11), which also confirms the kinetics study of BIFOXSiCl_2_ (**7**) and BIFOXSiCl(OH) (**8**) as the most stable dichlorosilane and chlorosilanol (Figure 2 and Figure 3, Table 2). In comparison the front attack mechanism for dichlorosilane **13** (*E*_a_ = 27.7 kcal mol^−1^, Table 3, entry 7) has a lower energy barrier than side attack mechanism (*E*_a_ = 35.4 kcal mol^−1^, Table 3, entry 8). The second hydrolysis, **13_ClOH_** to **1** has a higher energy barrier (*E*_a_ = 29.1 kcal mol^−1^ for front attack mechanism (Table 3, entry 9) and *E*_a_ = 29.9 kcal mol^−1^ for side attack mechanism (Table 3, entry 10)) than the first. In accordance with the kinetic study, dichlorosilane **13** hydrolysed faster than BIFOXSiCl_2_ (**7**, Scheme 4). In addition, the hydrolysis of glycol-based dichlorosilane (Table 3, entries 11 and 12) and tetrachlorosilane (Table 3, entries 13 to 16) is computed as model system. The first hydrolytic step of SiCl_4_ has a higher energy barrier (*E*_a_ = 28.4 kcal mol^−1^, Table 3, entry 13) than **13** to **13_ClOH_** (*E*_a_ = 27.7 kcal mol^−1^, Table 3, entry 7) and **13_ClOH_** to **1** (*E*_a_ = 29.1 kcal mol^−1^, Table 3, entry 9). The glycol-substituted dichlorosilane has a smaller energy barrier to the TS (*E*_a_ = 23.6 kcal mol^−1^, Table 3, entry 11, Figure 5 and *E*_a_ = 21.1 kcal mol^−1^, Table 3, entry 12, Figure 5). The stepwise hydrolysis of tetrachlorosilane shows that the energy barrier for the first step is higher (*E*_a_ = 28.4 kcal mol^−1^, Table 3, entry 13), than the second step (*E*_a_ = 25.0 kcal mol^−1^, Table 3, entry 14). With two hydroxy substituents, the energy barrier for the third TS is *E*_a_ = 20.9 kcal mol^−1^ (Table 3, entry 15) and for the fourth step (*E*_a_ = 20.8 kcal mol^−1^, Table 3, entry 16). For the computed values, it should be noted that THF is used for the PCM solvent correction, but the reactions are carried out in a 1/1 mixture of water/THF.

A hydrogen bridge is a displacement of electrons (lp or π) from a donor into the σ* orbital of an H–X bond. NBO analyses can be used to calculate the energy of such an interaction. With the shift of the electron density of a lone pair into the σ* orbital, the O–H bond of the HB donor is weakened. This causes a change in the O–H stretching frequency of the bond. A strong hydrogen bridge results from strong lone pair···σ* orbital interactions, resulting in a weakening of the O–H bond, which in turn results in a decrease of the O–H stretching frequency [71]. An alternative method is to determine the relaxed force constant [72]. In the following, the stretching frequency und the NBO analyses were calculated for different acceptors and water (Table 4).

**Table 4:** Computed^a^ stretching frequencies (ν [cm^−1^]), lp···σ* [kcal mol^−1^] and donor acceptor distances (D [Å]) of silanes **7**, **8**, [CH_2_O]_2_SiCl(OH), [CH_2_O]_2_Si(OH)_2,_ (OH)_2_SiCl(OH), Si(OH)_4_, methanol and [CH_2_O]_2_C(OH)_2_.

entry		ν_O-H_ [cm^−1^]	lp-σ* energy [kcal mol^−1^]	D O···X [Å]

1	CH_3_-OH···OH_2_	3741	11.88	2.785
2	SiH_3_-OH···OH_2_	3602	16.43	2.793
3	Si(OH)_3_-OH···OH_2_ H_2_O···OH-Si(OH)_3_	3441 3608	20.39 12.05	2.732 2.797
4	SiCl(OH)_2_-OH···OH_2_ H_2_O···OH-SiCl(OH)_2_	3450 3649	19.44 7.78	2.727 2.862
5	SiCl(OH)_2_-OH···OH_2_ SiCl(OH)_2_-OH···OH_2_ H_2_O···Cl-Si(OH)_3_	3725 3701 3832	6.78 6.82 1.58	2.865 2.863 2.804
6	(CH_2_)_2_O_2_Si-(OH)_2_···OH_2_	3745^b^ 3717^b^ 3691^c^	7.62 7.59	2.855 2.857
7	(CH_2_)_2_O_2_SiCl-OH···OH_2_ H_2_O···Cl-Si(OH)O_2_(CH_2_)_2_	3377 3819^c^ 3707^b^	22.16 1.58	2.705 3.331
8	(CH_2_)_2_O_2_C-(OH)_2_···OH_2_ (CH_2_)_2_O_2_C-(OH)_2_···OH_2_ H_2_O···O_2_(CH_2_)_2_C(OH)_2_	3678^c^ 3631^c^ 3604^c^	6.57 8.69 8.96	2.844 2.816 2.715
9	BIFOXSiCl-**OH**···OH_2_ BIFOXSi(OH)-**Cl**···H_2_O	3445 3691^b^ 3811^c^	24.90 5.19	2.734 3.273
10	BIFOXSi(OH)_2_···OH_2_	3733^b^ 3703^c^ 3685^c^	9.73 10.15	2.878 2.834
11	BIFOXSiCl-OH···Cl^-^	3165	36.87	2.994
12	BIFOXSi(OH)_2_···Cl^-^	3421 3151	17.36 33.61	3.082 2.927
13	BIFOXSi(OH)_2_ – dimer	3670 3490	7.55 21.63	2.880 2.752

^a^Computed with B3LYP-D3BJ/6-31G(d) at 298 K; ^b^symmetric stretching frequency ν_s_; ^c^asymmetric stretching frequency ν_as_.

The silanol group is a better hydrogen bond acceptor than an alcohol group for single hydrogen bonds (CH_3_OH (11.88 kcal mol^−1^) vs SiH_3_OH (16.43 kcal mol^−1^), Table 4, entries 1 and 2), which is more acidic and inconsistent with the results of West et al. [73]. In case of double hydrogen bonds in the glyoxal based system, both are equally strong, because of a third hydrogen bond, a rebond from where water is the acceptor and the oxygen in the ring is the donor ((CH_2_)_2_O_2_Si(OH)_2_ (15.21 kcal mol^−1^) vs (CH_2_)_2_O_2_C(OH)_2_ (15.26 kcal mol^−1^), Table 4, entries 6 and 8), Two possible geometries can be observed for SiCl(OH)_3_. On the one hand with two hydrogen bridges to water (13.60 kcal mol^−1^, Table 4, entry 5) or with one hydrogen bridge to water (19.44 kcal mol^−1^, Table 4, entry 4), with an additional rebond of the oxygen of one of the SiOH groups to water H–O (7.78 kcal mol^−1^, Table 4, entry 4). The trend of the stronger single hydrogen bridge compared to the double hydrogen bridge is also reflected in the systems glycolic (22.16 kcal mol^−1^ vs 15.21 kcal mol^−1^, Table 4, entries 6 and 7) and BIFOSi (24.90 kcal mol^−1^ vs 19.88 kcal mol^−1^, Table 4, entries 9 and 10). As was to be expected, the energies of the O–H bonds decreases with increasing lone pair···σ* orbital interactions (3502 cm^−1^ to 22.16 kcal mol^−1^ vs 3830 cm^−1^ to 3.06 kcal mol^−1^, Table 4, entries 5 and 7). With a stronger electron donor, as the chloride ion, the lone pair···σ* orbital interactions rises (36.87 kcal mol^−1^ vs 50.97 kcal mol^−1^, Table 4, entries 11 and 12). In here, the BIFOXSi(OH)_2_ (**9**) binds the chlorid stronger with two hydrogen bridges, than BIFOXSiCl(OH) (**8**) with just one hydrogen bridge. The BIFOXSi(OH)_2_ (**9**) dimer forms hydrogen bridges, which are stronger than hydrogen bridges with water, but less stronger than hydrogen bridges to chloride (50.97 kcal mol^−1^ vs 29.18 kcal mol^−1^ vs 19.88 kcal mol^−1^, Table 4, entries 13, 12, and 10).

### X-ray analyses

**Figure 12:**
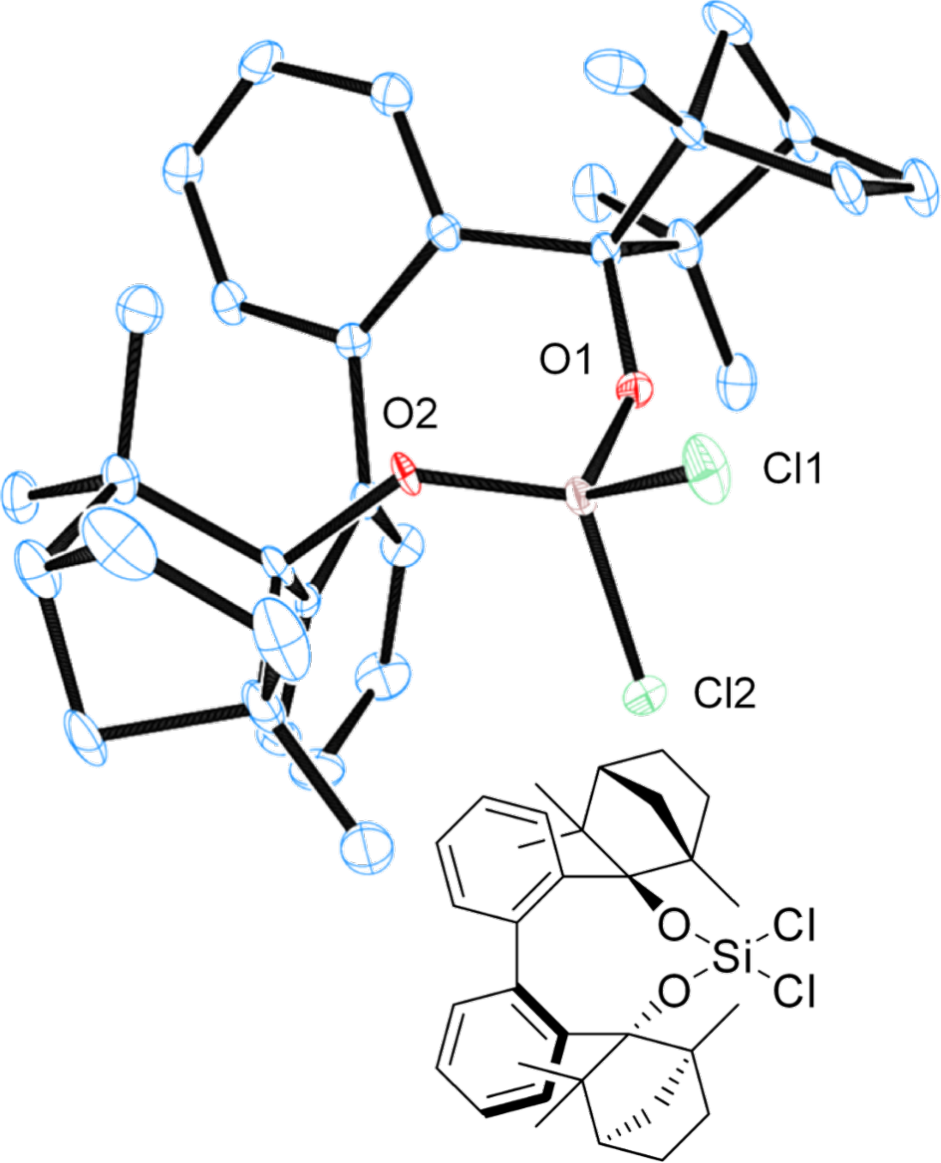
X-ray crystal structure of BIFOXSiCl_2_ (**7**). H atoms on the chiral backbone are omitted for clarity issues and the ellipsoids are shown with 65% probability.

**Figure 13:**
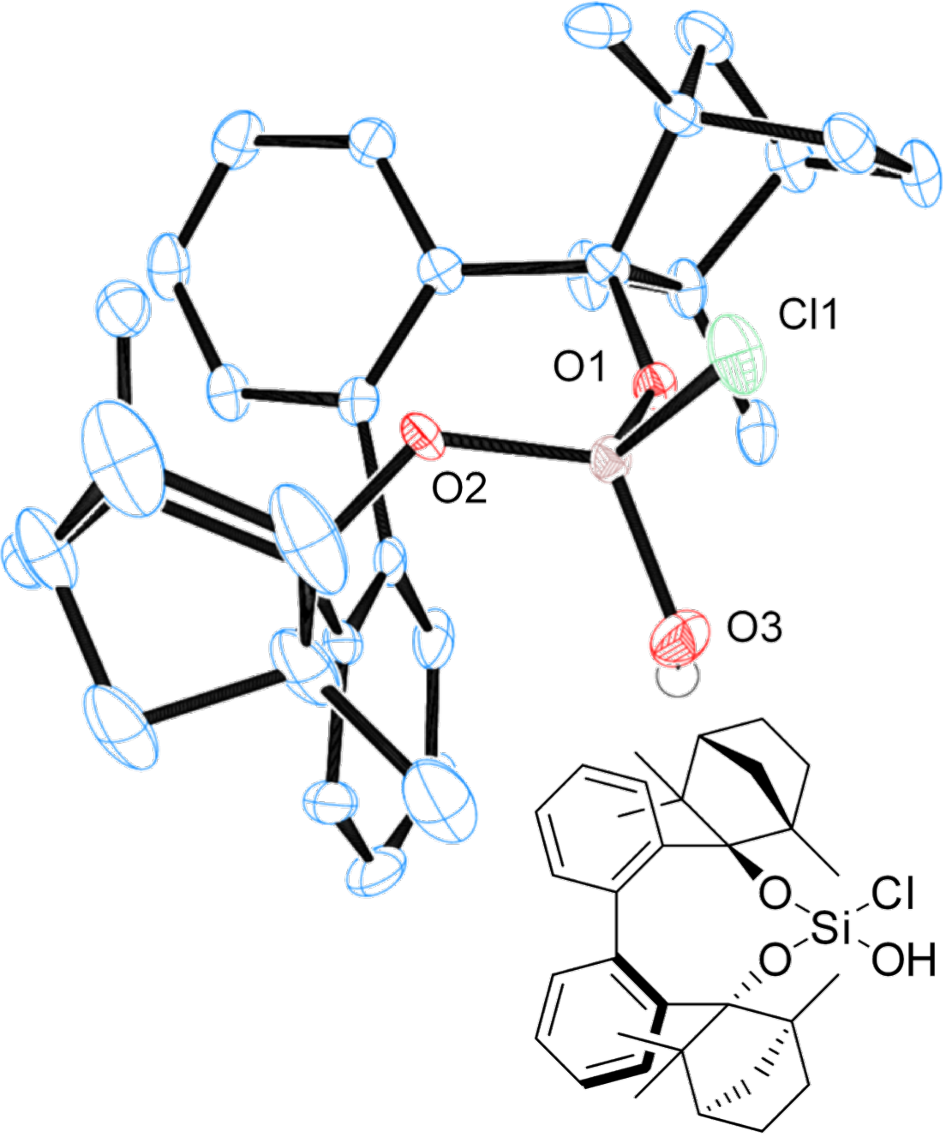
X-ray crystal structure of BIFOXSiCl(OH) (**8**). H atoms on the chiral backbone are omitted for clarity issues and the ellipsoids are shown with 65% probability.

Dichlorosilane **7** (Figure 12), as well as chlorosilanol **8** (Figure 13) crystallize as monomers from *n-*hexane. BIFOXSiCl(OH) (**8**) is obtained as the **8_ax_** isomer, which is computed to be the more stable isomer (Table 3, entry 1).

**Figure 14:**
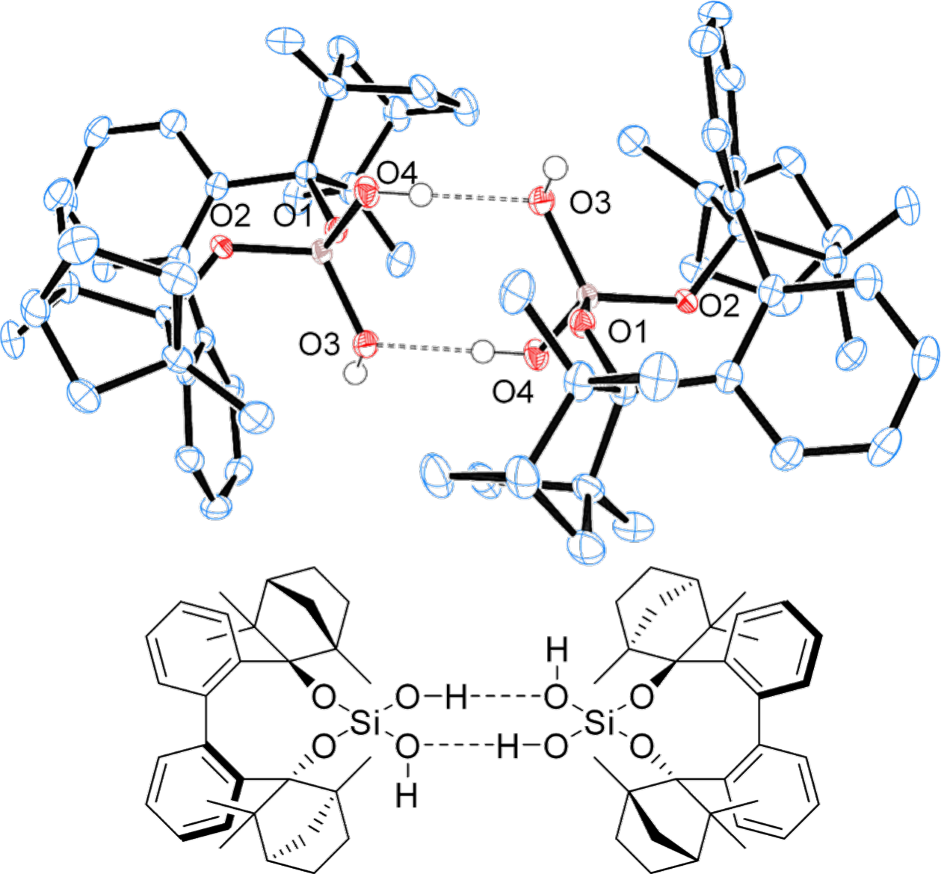
X-ray crystal structure ofrac-BIFOXSi(OH)_2_ (**9**) forming dimers. H atoms on the chiral backbone are omitted for clarity issues and the ellipsoids are shown with 65% probability. For bond lengths and angles see Table 5.

**Figure 15:**
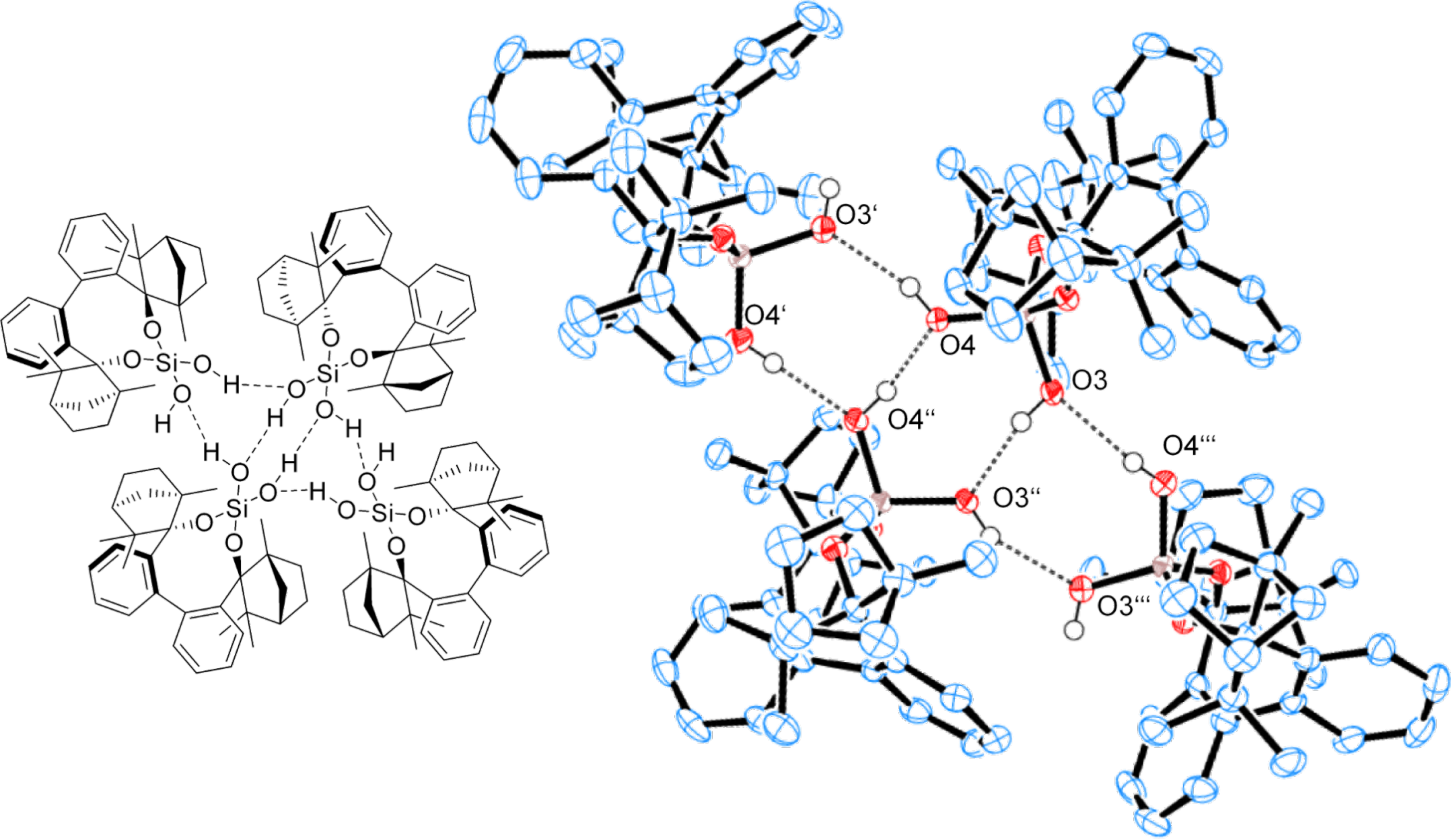
X-ray crystal structure of BIFOXSi(OH)_2_ (**9**) forming a tetramer. H atoms on the chiral backbone are omitted for clarity issues and the ellipsoids are shown with 65% probability. For bond lengths and angles see Table 5.

Since the commercially available (+)-fenchone has 98% enantiomeric purity, BIFOXSi(OH)_2_ can be further purified. rac-BIFOXSi(OH)_2_ crystallizes as a dimer from toluene (Figure 14). BIFOXSi(OH)_2_ (**9**) crystallizes as a tetramer from *n-*hexane (Figure 15), where six OH groups build a network of hydrogen bonds. Thus the polar core is shielded against the solvent (Figure 15). For silanediols **2a** and **2b** (Figure 1) dimeric structures are reported as well [41–44]. In these, hydrogen bond lengths of 1.86 Å to 2.01 Å (H···O) and 2.65–2.80 Å (O–H···O) are observed. Bond angles vary from 157.3° to 174.3° for the hydrogen bonds between the silanediols [41–44]. In the dimeric and tetrameric structure of BIFOXSi(OH)_2_ (**9**, Figure 14 and Figure 15) those distances are 1.82(4) Å to 2.02(4) Å (H···O) and 2.66(4) Å to 2.79(5) Å (O–H···O) with angles between 152,4(6)° and 172,0(7)° (Table 5). These distances indicate medium strong hydrogen bonds [74].

**Figure 16:**
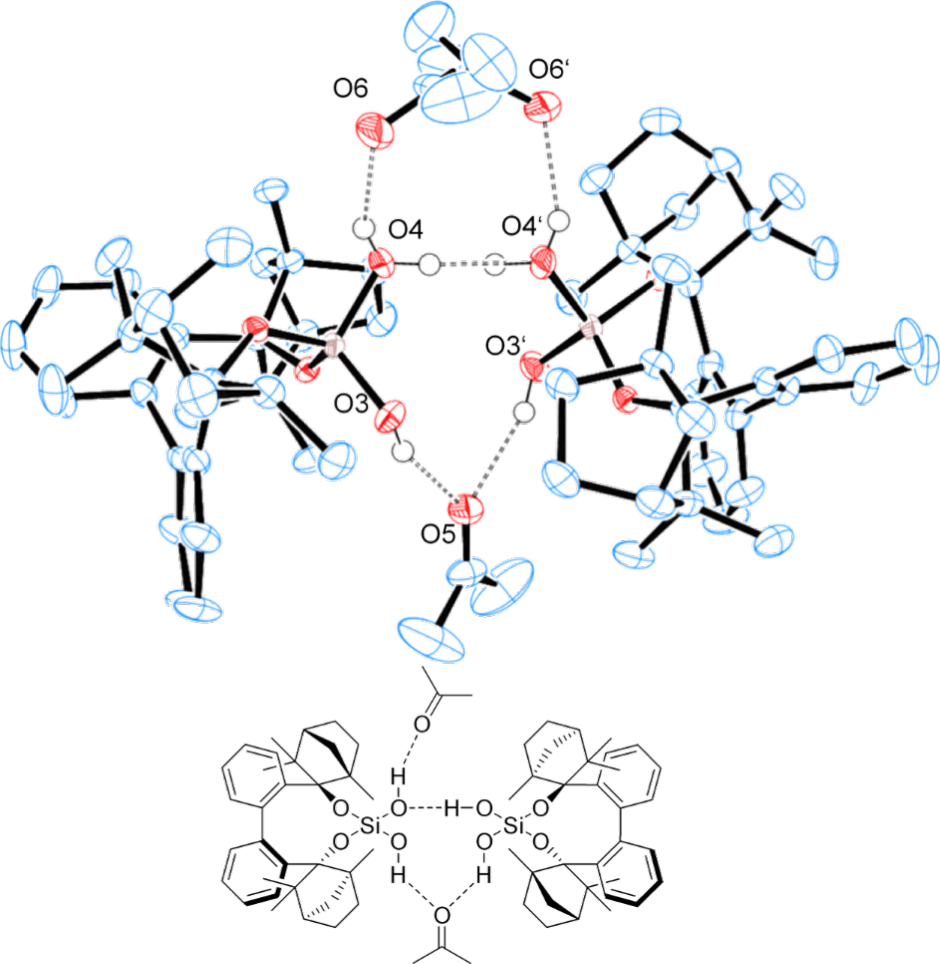
X-ray crystal structure of BIFOXSi(OH)_2_ (**9**) forming a dimeric structure with two bridged acetone molecules. H atoms on the chiral backbone are omitted for clarity issues. The acetone on the upper side is disordered by 50%. Ellipsoids are shown with 65% probability. For bond lengths and angles see Table 5.

**Figure 17:**
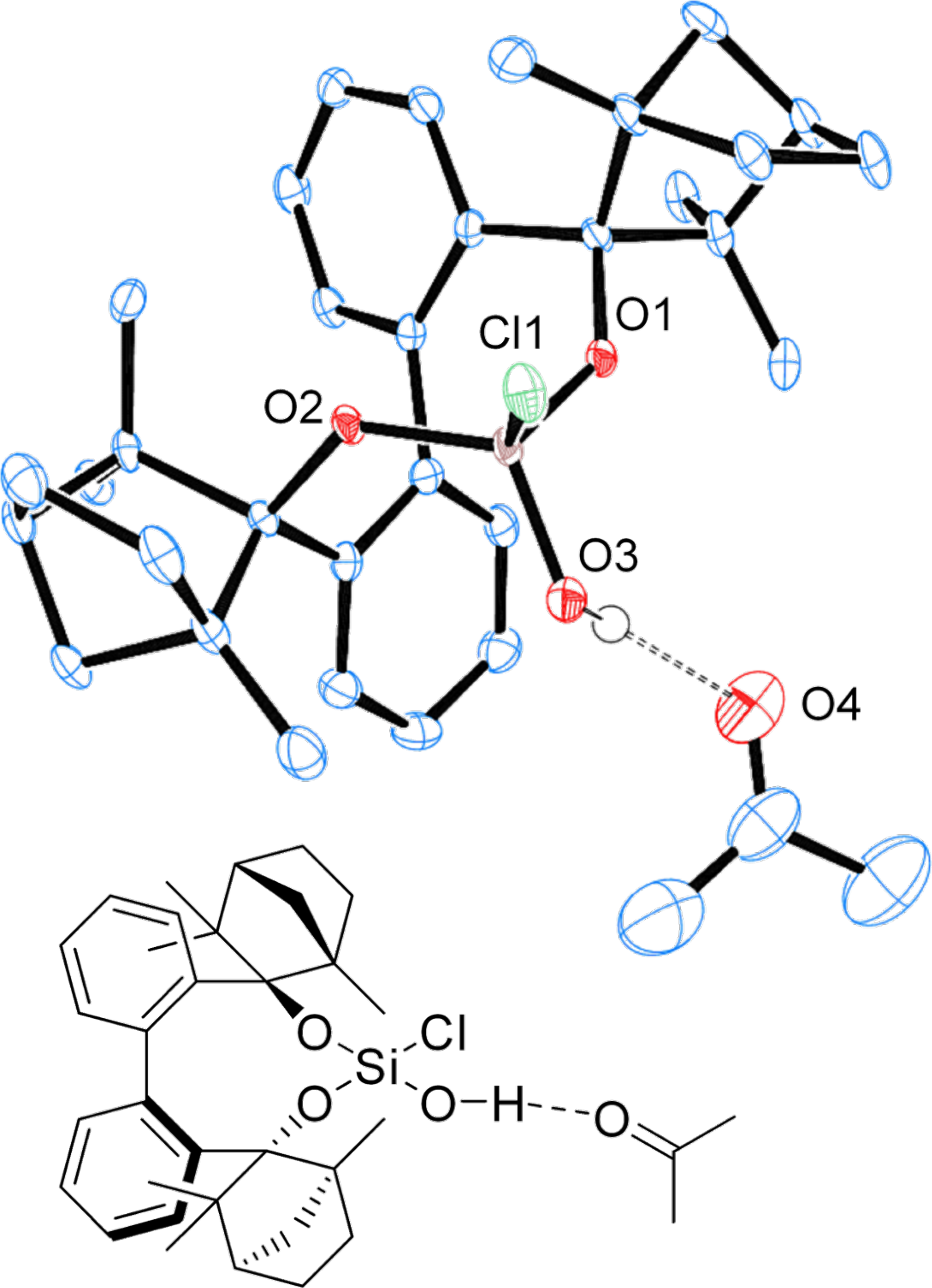
X-ray crystal structure of BIFOXSiCl(OH) (**8**), binding an acetone molecule. H atoms on the chiral backbone are omitted for clarity issues and the ellipsoids are shown with 65% probability. For bond lengths and angles see Table 5.

X-ray structures of BIFOXSi(OH)_2_ (**9**, Figure 16) and BIFOXSiCl(OH) (**8**, Figure 17) with co-crystallized acetone indicate the bonding behavior of the silanediols to carbonyl acceptors.

Chlorosilanol **8** binds one acetone with a bonding length of 2.16(0) Å (H3···O4) and 2.89(4) Å (O3–H···O4) (Table 5, Figure 17), which is the longest hydrogen bond for BIFOXSiCl(OH) (**8**) and BIFOXSi(OH)_2_ (**9**). In dimeric structures of silanediol **9** (Figure 14) the hydroxy group O4H is a hydrogen bond donor to O3. The hydrogen atom of the O–H group (O3H) is pointing outwards and can form a hydrogen bond to an additional molecule. For silanediols **2a** and **2b** Franz et al. observed hydrogen bond distances of 1.88 Å (H···O) and 2.68 Å (O–H···O) on average to guest molecules [41–44]. BIFOXSi(OH)_2_ (**9**, Figure 16) binds an acetone in a similar manner (O4’–H···O4–H···O6=C(CH_3_)_2_) with distances of 1.88(3) Å (H4···O6) and 2.62(1) Å (O4–H···O6) as well as 2.05(2) Å (H4···O4’) and 2.73(1) Å (O4–H···O4’) between the hydroxy groups. The second acetone is bonded with one hydroxy group of each of the BIFOXSi(OH)_2_ (**9**) with longer distances of 2.03(0) Å (H···O) and 2.80(3) Å (O–H···O) (Table 5). Similarly to the previously reported silanediol derivatives **2a** and **2b** [43], an increase of acidity of one outward facing OH group is achieved by intermolecular hydrogen bonds [43].

**Table 5:** Bond lengths and angles of hydrogen bonds in X-ray crystal structures of BIFOXSiCl(OH) (**8**) and BIFOXSi(OH)_2_ (**9**).

	D–H···A	D (D···A)^a^ [Å]	D (H···A)^b^ [Å]	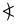 (D-H···A) [°]

BIFOXSi(OH)_2_ (**9**) (Figure 14)	O4H–O3	2.76(1)	1.92(0)	170.0(5)
BIFOXSi(OH)_2_ (**9**) (Figure 15)	O4H–O3’ O4’H–O4’’ O4’’H–O4 O3H–O3’’ O3’’H–O4’’’ O4’’’H–O3	2.76(2) 2.73(5) 2.66(4) 2.68(3) 2.79(9) 2.74(7)	1.98(0) 1.95(0) 1.82(4) 1.85(8) 2.02(1) 2.02(4)	163.7(4) 172.0(7) 165.5(0) 168.9(8) 152.4(6) 165.9(3)
BIFOXSi(OH)_2_ (**9**) (Figure 16)	O3H–O5 O4H–O4’ O4H–O6	2.80(3) 2.73(1) 2.62(1)	2.03(0) 2.05(2) 1.88(3)	161.1(8) 176.2(8) 146.3(6)
BIFOXSiCl(OH) (**8**) (Figure 17)	O3H–O4	2.89(4)	2.16(0)	167.1(8)

^a^Distance for hydrogen bond donor (D) to hydrogen bond acceptor (A). ^b^Distance for hydrogen (H) to hydrogen bond acceptor (A).

### Chloride binding

The X-ray crystal structures of chlorosilanol **8** (Figure 17) and silanediol **9** (Figure 16) with co-crystallized acetone indicate the ability of binding ions or molecules via hydrogen bonds. To investigate this ability, UV–vis titration experiments with tetrabutylammonium chloride (TBA-Cl) are carried out, with silanediol **1** as a reference (Table 6) [47,75]. For BIFOXSi(OH)_2_ (**9**, Figure 14, Figure 15) a binding constant of 5274.9 M^−1^ (13%) for chloride is determined, which is in the same order as di(1-naphthyl)silanediol (**1**, Figure 1) with 4688.0 M^−1^ (5%, Table 6). The binding constant of chlorosilanol **8** (Figure 13) for chloride is 451.1 M^−1^ (4%). Thus chlorosilanol **8** and silanediol **9** are feasible for anion binding of chloride.

**Table 6:** Chloride binding constants (*K* [M^−1^], error (%)) to BIFOXSiCl(OH) (**8**), BIFOXSi(OH)_2_ (**9**) and di(1-naphthyl)silanediol (**1**) in HCCl_3_ at 22 °C [75].

	**8**	**9**	**1**

binding constant^a^	451.1 (4%)	5274.9 (13%)	4688.0 (5%)

^a^Calculated for 1:1 binding mode between chloride and hydrogen bond donor [75].

### Counter ion catalyses

**Scheme 6:**
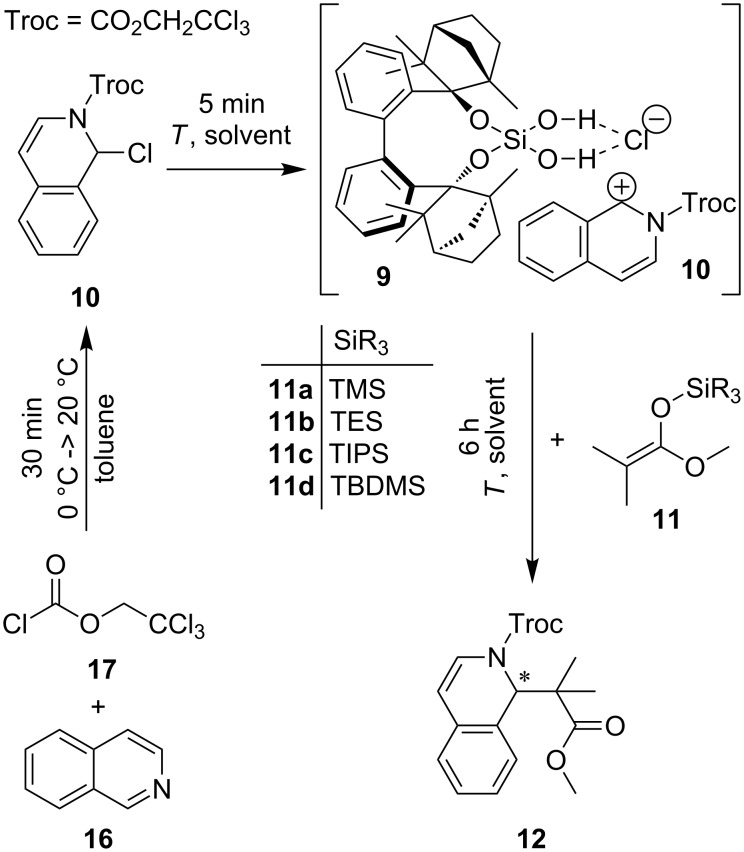
Hydrogen-bond-catalyzed *N*-acyl Mannich reaction of in situ-generated **10** with different silyl ketene acetals **11 a**–**d** and BIFOXSi(OH)_2_ (**9**) yielding C–C coupling product **12** (Tables 6–8); (TMS: trimethylsilane. TES: triethylsilane. TIPS: triisopropylsilane. TBDMS: *tert-*butyldimethylsilane).

The *N*-acyl Mannich reaction of isochinolin (**16**), which is activated with 2,2,2-trichloroethoxycarbonyl chloride (**17**, TrocCl) to carbamate **10**, and different silyl ketene acetals **11a**–**d** yielding product **12** (Scheme 6) [45,47], is studied. Mattson et al. proposed a mechanism where the chloride ion is abstracted from **10** and binds via hydrogen bonding to the catalyst (Scheme 6) [45,47]. This leads to an ion pair [cat•Cl]^−^ and [isoquinolinium cation]^+^ (Scheme 6). The nucleophilic silyl ketene acetal reacts with the [isoquinolinium cation]^+^ and forms the C–C bond, yielding product **12** (Scheme 6).

The reactivity and stability of such an ion pair depend on the employed solvent. For this reaction and BIFOXSi(OH)_2_ (**9**) as catalyst, several solvents are tested (Table 7). In a nonpolar solvent like *n-*hexane, no catalytic activity is observed (Table 7, entry 1). In halogenated solvents as dichloromethane (DCM) and 1,2-dichloroethane (1,2-DCE) the reaction takes place but without any enantiomeric excess (ee) (Table 7, entries 2 and 3). In DCM the highest yield is isolated, but that is due to a fast background reaction [45]. With toluene as solvent, no background reaction is observed (Table 9, entry 9). To stabilize and improve the ion pair, polar solvents are tested as diethyl ether and dimethylformamide gave no conversion and starting material is obtained (Table 7, entries 4 and 5), acetonitrile and acetone increase the yield (Table 7, entries 6 and 7), but without any enantiomeric excess. In toluene, BIFOXSi(OH)_2_ (**9**), forms 43% yield at −80 °C and 5% ee (Table 7, entry 8). At higher temperature, **12** is isolated with 52% yield and 12% ee (−60 °C, Table 7, entry 9). Further aromatic solvents are tested (Table 7, entries 10–13), but without any improvement in yield or ee.

**Table 7:** *N*-acyl Mannich reaction of **10** and **11c** catalyzed by silanediol **9** yielding product **12** with different solvents (Scheme 6)^a^.

entry	solvent	*T* [°C]	yield [%]^b^	ee [%]^c^

1	*n-*hexane	−60	0	–
2	DCM	−60	87	0
3	1.2-DCE	−30	33	0
4	diethyl ether	−60	0	–
5	dimethylformamide	−40	0	–
6	acetonitrile	−40	35	1
7	acetone	−80	10	0
8	toluene	−80	43	5
9	**toluene**	**–60**	**52**	**12**
10	benzene	rt	10	1
11	*m-*xylene	−60	<1	12
12	nitrobenzene	rt	40	0
13	pyridine	−30	0	–

^a^Reactions carried out with 20 mol % **9**, 0.1 mmol **16**, 0.11 mmol **17** and 0.15 mmol **11c** in 4 mL solvent; ^b^isolated yields; ^c^chiral HPLC OD-H, *n-*hexane/iPrOH 90:10, 1 mL/min, 220 nm, 25 °C, (−)-**12** correlates to (*S*)-**12** [45,76].

Variation of the catalyst loading suggests a ratio of 10 mol % of BIFOXSi(OH)_2_ (**9**, Table 8, entries 1–4) to be optimal. An increase of temperature results in decreasing yields and ee (Table 8, entries 5–8). The highest ee is found with 20 mol % catalyst (12% ee, 52% yield, Table 7, entry 9).

**Table 8:** Different catalyst loadings of BIFOXSi(OH)_2_ (**9**) and different temperatures in the *N*-acyl Mannich reaction of **10** and **11c** yielding product **12** (Scheme 6)^a^.

entry	cat loading [mol %]	*T* [°C]	yield [%]^b^	ee [%]^c^

1	20	−80	43	5
2	10	−80	52	7
3	5	−80	12	0
4	1	−80	2	2
5	10	−60	37	5
6	10	−40	18	7
7	10	0	9	1
8	10	rt	20	0

^a^Reactions carried out with 0.1 mmol **16**, 0.11 mmol **17** and 0.15 mmol **11c** in 4 mL toluene; ^b^isolated yields, ^c^chiral HPLC OD-H, *n-*hexane/iPrOH 90:10, 1 mL/min, 220 nm, 25 °C, (−)**-12** correlates to (*S*)-**12** [45,76].

BIFOXSi(OH)_2_ (**9**) performs better than BIFOXSiCl(OH) (**8**) which is in accordance with the determined binding constant for chloride (Table 6, Table 9). With BIFOXSiCl(OH) (**8**) a yield up to 60% is isolated (Table 9, entry 3), but as racemate. BIFOXSi(OH)_2_ (**9**) catalyses the reaction with good yields up to 73% and an ee value of 12% (Table 9, entry 7). For **11a**, **b** and **d** and silanediol **9** as catalyst, an enantiomeric inversion is observed (Table 9, entries 5, 6 and 8).

**Table 9:** Performance of catalyst **8** and **9** in the *N*-acyl Mannich reaction with **10** and different silyl ketene acetals **11a**–**d** and yielding in **12** (Scheme 6)^a^.

entry	catalyst	SiR_3_	yield [%]^b^	ee [%]^c^

1	**8**	**11a** TMS	56	3 ***S-12***
2	**8**	**11b** TES	42	4 ***S-12***
3	**8**	**11c** TIPS	60	4 ***S-12***
4	**8**	**11d** TBDMS	46	2 ***S-12***
5	**9**	**11a** TMS	73	6 ***R-12***
6	**9**	**11b** TES	67	2 ***R-12***
7	**9**	**11c** TIPS	52	12 ***S-12***
8	**9**	**11d** TBDMS	72	2 ***R-12***
9	–	**11c** TIPS	0	–

^a^Reactions carried out with 0.1 mmol **16**, 0.11 mmol **17**, 0.15 mmol **11a**–**d** and 20 mol % cat in 4 mL toluene at −60 °C; ^b^isolated yields; ^c^chiral HPLC OD-H, *n-*hexane/iPrOH 90:10, 1 mL/min, 220 nm, 25 °C, (−)-**12** correlates to (*S*)-**12** [45,76].

**Scheme 7:**
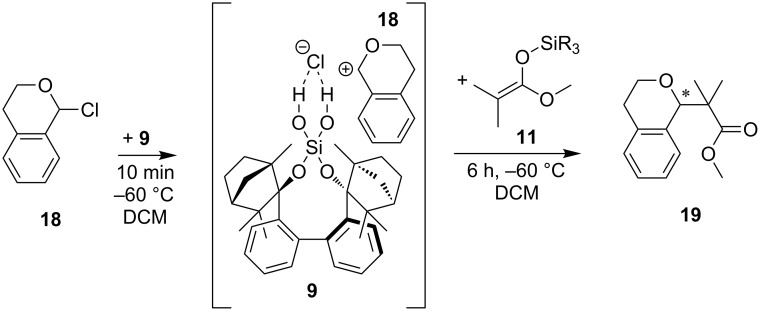
Hydrogen-bond-catalyzed nucleophilic substitution of **18** with BIFOXSi(OH)_2_ (**9**) and nucleophile silyl ketene acetals **11**. **18** and **9** form an activated electrophile ion pair complex which yields C–C coupling product **19** (Table 10).

The substrate scope is broadened with 1-chloroisochroman (**18**) as alternative substrate (Table 10). The reaction mechanism is analogue to the *N*-acyl Mannich reaction (Scheme 6 vs Scheme 7). The catalyst abstracts and binds the chloride anion and forms an ion pair [cat•Cl]^−^ and oxocarbenium ion [**18**]^+^. Silyl ketene acetal **11** reacts with this ion pair complex to product **19** [77–78]. Only with DCM as solvent, product **19** of the reaction has been isolated (Table 10). Silanediol **9** and silyl ketene acetal **11a** provide the highest yield (85%, Table 10, entry 5). The substitution pattern on the silyl ketene has a direct influence on the yield.

The highest yield is reached with TMS substitution (silanediol **9**, 85% yield, Table 10, entry 5; chlorosilanol **8**, 54% yield, Table 10, entry 9). The yield decreases as the substituents become larger (Table 10, entries 6–8, 10 and 11). This trend can also be seen in the uncatalyzed reaction (Table 10, entries 13, 14). Only for **11c** and silandiol **9** a considerable ee with 5% is determined (Table 10, entries 7 and 11). Chlorosilanol **8** does not show catalytic activity for the reaction of **18** with **11a**, as the background reaction is slightly faster (54% vs 58%, Table 10, entries 9 and 13). With increasing of the steric demand of the nucleophilic silyl group, the background reaction slows down and chlorosilanol **8** has a positive influence on the yields and the enantiomeric excess.

**Table 10:** Hydrogen-bond-catalyzed addition of silyl ketene acetals **11a**–**d** with 1-chloroisochroman (**18**) to product **19** with chlorosilanol **8** and silanediol **9** in different solvents^a^ (Scheme 7).

entry	cat.	solvent	SiR_3_	yield [%]^b^	ee [%]^c^

1	**9**	toluene	**11a** TMS	0	–
2	**9**	MTBE	**11a** TMS	0	–
3	**9**	THF	**11a** TMS	0	–
4	**9**	diethyl ether	**11a** TMS	0	–
5	**9**	DCM	**11a** TMS	85	1
6	**9**	DCM	**11b** TES	37	3
7	**9**	DCM	**11c** TIPS	12	5
8	**9**	DCM	**11d** TBDMS	8	3
9	**8**	DCM	**11a** TMS	54	0
10	**8**	DCM	**11b** TES	46	2
11	**8**	DCM	**11c** TIPS	16	5
12	**8**	DCM	**11d** TBDMS	12	3
13	–	DCM	**11a** TMS	58	–
14	–	DCM	**11c** TIPS	5	–

^a^Reactions carried out with 0.15 mmol **18**, 0.22 mmol **11a**–**d** and 20 mol % cat in 1.2 mL solvent at −60 °C; ^b^isolated yields; ^c^chiral HPLC OD-H, *n-*hexane/iPrOH 100:0, 1.0 mL/min, 210 nm, 25 °C, (−)-**18** correlates to (*S*)-**18** [77].

**Scheme 8:**
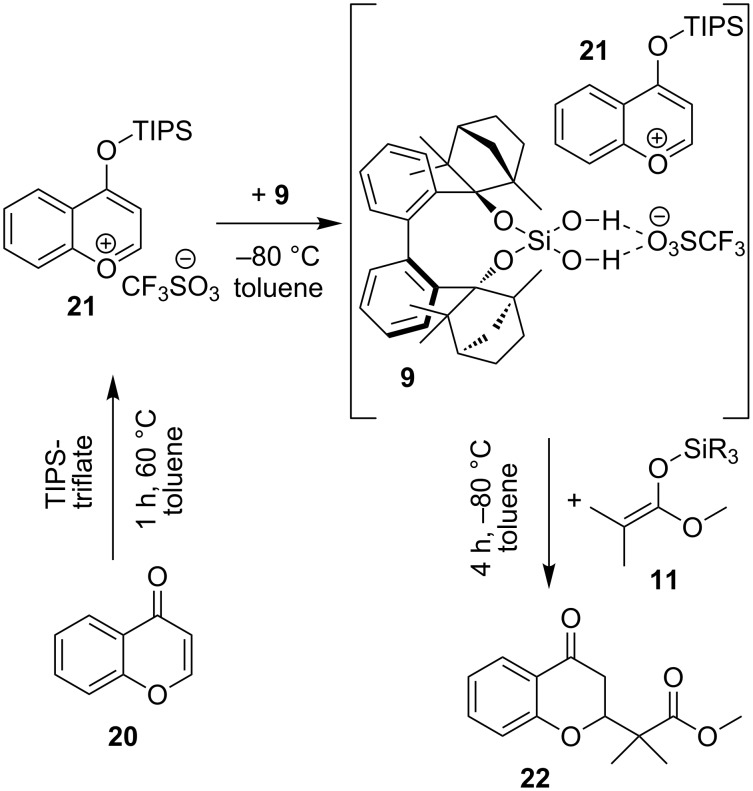
Nucleophilic substitution of **20** with BIFOXSi(OH)_2_ (**9**) and nucleophile silyl ketene acetals **11**, **20** and **9** form an activated electrophile ion pair complex which yields C–C coupling product **22** (Table 11).

In a third reaction, the 1,4 addition of silyl keten acetals **11** to chromone **20** is investigated (Table 11, Scheme 8). Chromone **20** is first transformed to the oxonium ion pair **21**. Catalyst BIFOXSi(OH)_2_ (**9**) binds the triflate anion via hydrogen bonding and leaves the pyrylium derivative **21** for the nucleophilic attack of silyl keten acetals **11** to form product **22** (Scheme 8).

With BIFOXSi(OH)_2_ (**9**) an increase of yield (71% yield, 4% ee, Table 11, entry 2) compared to the not catalyzed reaction (48%, Table 11, entry 10) is achieved. BIFOXSiCl(OH) (**8**) has no activation ability, but little effect on the ee (46% yield, 1% ee, Table 11, entry 6). Here, the sterically demanding silyl keten acetals increase yields. With BIFOXSi(OH)_2_ (**9**), the bulkiest acetal **11d** yields 98% and the smallest **11a** yields 60% of product **22** (Table 11, entries 1–4). This tendency is the same for BIFOXSiCl(OH) (**8**, Table 11, entries 5–8), with exception of acetal **11a**, which yields 55% of product **22**. Mattson et al. used 2,6-di-*tert*-butyl-4-methylpyridine as additive [48]. It is added at the beginning to the reaction, so it should support the formation of the ion pair **21**. As organic base it binds also to BIFOXSi(OH)_2_ (**9**), which results in a lower yield and ee (Table 11, entry 9).

**Table 11:** Hydrogen-bond-catalyzed addition of silyl ketene acetals **11a**–**d** with chromone **20** to product **22** with chlorosilanol **8** and silanediol **9** in toluene^a^ (Scheme 8).

entry	catalyst	SiR_3_	yield [%]^b^	ee [%]^c^

1	**9**	**11d** TBDMS	98	1
2	**9**	**11c** TIPS	71	4
3	**9**	**11b** TES	69	1
4	**9**	**11a** TMS	60	0
5	**8**	**11d** TBDMS	70	0
6	**8**	**11c** TIPS	46	1
7	**8**	**11b** TES	35	1
8	**8**	**11a** TMS	55	1
9^d^	**9**	**11c** TIPS	50	2
10^e^	–	**11c** TIPS	48	–

^a^Reactions carried out with 0.15 mmol **20**, 0.22 mmol **11a**–**d** and 20 mol % cat in 4 mL toluene at −80°C; ^b^isolated yields; ^c^chiral HPLC AD-H, *n-*hexane/iPrOH 98:2, 1 mL/min, 254 nm, 25 °C, (+)-**22** correlates to (*S*)***-*22** [48]; ^d^with 20 mol % 2.6-di-*tert*-butyl-4-methylpyridine as additive for step 1 (Scheme 8); ^e^without catalyst.

## Conclusion

Enantiopure fenchole-based silanediol BIFOXSi(OH)_2_ (**9**, Figure 1) and chlorosilanol BIFOXSiCl(OH) (**8**, Figure 1) are efficiently accessible from BIFOL (**5**) via enforced hydrolysis of dichlorosilane **7**, i.e., H_2_O/THF reflux, 19 h (Scheme 3, Figure 2).

DFT computations reveal two different hydrolysis mechanisms and explain the unusual low reactivity of BIFOXSiCl_2_ (**7**) and BIFOXSiCl(OH) (**8**, Table 3) with sterically demanding endo fenchone groups. For BIFOXSiCl(OH) (**8**) two isomers (**8_eq_** vs **8_ax_**) are found computationally. Chlorosilanol **8_ax_**, with the axial Si–OH alignment, is the thermodynamically more stable isomer (Δ*E*_r_ = 2.7 kcal mol^−1^, Table 3), in accordance with X-ray crystal structure analyses of **8** (Figure 13). The first hydrolysis has a higher activation barrier than the second step, and thus appears to be rate-determining.

In the X-ray crystal structures of BIFOXSiCl(OH) (**8**, Figure 13) and BIFOXSi(OH)_2_ (**9**, Figures 14–16), intermolecular hydrogen bonds are apparent. The lengths of these hydrogen bonds vary from 1.88(3) to 2.16(2) Å (Table 5). The longest bond appears between BIFOXSiCl(OH) (**8**) and acetone (Figure 17). This suggests that BIFOXSiCl(OH) (**8**) is the weaker hydrogen bond donor, compared to BIFOXSi(OH)_2_ (**9**). This is additionally supported by UV–vis titrations of chloride with BIFOXSiCl(OH) (**8**, 451.1 (4%) M^−1^) and BIFOXSi(OH)_2_ (**9**, 5274.9 (13%) M^−1^, Table 6).

Both new hydrogen bond catalysts can be used for the C–C coupling in the *N*-acyl Mannich reaction with activated isochinolin **10**, 1-chloroisochroman (**18**) and chromone **21** with different silyl ketene acetals. Due to more efficient bifunctional Si(OH)_2_-hydrogen bonding, silandiol **9** tops chlorosilanol **8**, also on catalytic application.

## Computational Details

In this work computations were performed using GAUSSIAN 09 [79]. Geometry optimizations and frequency computations were performed at the B3LYP-D3BJ/6-31G(d) level of theory. Zero-point energies were scaled by 0.96 [80]. Single point energies were performed at the M06-2X-D3/6-311++G(d,p) level of theory using the PCM method.

## Experimental

**General considerations:** All reactions were carried out under an argon atmosphere by using Schlenk techniques, unless otherwise stated. Solvents used in chemical conversions were dried by standard methods and distilled under argon prior to use unless otherwise specified. NMR spectra were recorded on a Bruker Avance II 300 instrument. UV–vis spectra were recorded by using a Perkin Elmer Lambda35 spectrometer. The samples were placed in quartz cells of 1 cm path length. NMR spectra, UV–vis spectra, crystal data and the coordinates of computed stationary points/transition states, as well as experimental details can be found in Supporting Information File 1. CCDC-1833170 (BIFOXSiCl_2_ (**7**)), CCDC-1833171 (BIFOXSiCl(OH) (**8**)), CCDC-1833172 (BIFOXSi(OH)_2_ (**9**, dimer)), CCDC-1833173 (BIFOXSi(OH)_2_ (**9**, tetramer)), CCDC-1833174 (BIFOXSiCl(OH) **8**·acetone) and CCDC-1833175 (BIFOXSi(OH)_2_ **9**·acetone) contain the supplementary crystallographic data for this paper. These data can be obtained free of charge from The Cambridge Crystallographic Data Centre via http://www.ccdc.cam.ac.uk/data_request/cif.

**Synthesis of BIFOXSiCl_2_** **(7):** In a dried Schlenk flask BIFOL (**5**, 2.3 g, 5 mmol. 1 equiv) was solved in THF (25 mL) in an inert gas atmosphere. After cooling to 0 °C, *n-*BuLi (4 mL, 10 mmol, 2 equiv, 2.5 M in *n-*hexane) was slowly added. The solution was stirred for 30 min und was allowed to warm up to 20 °C. After cooling to 0 °C again tetrachlorosilane (2.86 mL, 25 mmol, 5 equiv) was added dropwise. The reaction mixture was warmed to 20 °C and stirred overnight. Aqueous work-up with saturated NH_4_Cl followed by extracting with Et_2_O (three times) and concentrated in vacuo results in crude product. After purification with silica gel flash column chromatography (100% *n-*hexane, *R*_f_: 0.58) BIFOXSiCl_2_ (**7**) was obtained as a white solid (2.5 g, 4.6 mmol, 92%); mp 219.4 °C; [α]_436_^20^ = −54.90 (*c* 0.46, CHCl_3_); ^1^H NMR (300 MHz, CDCl_3_, 25 °C, TMS) δ 7.61 (dd, *J* = 8.1, 1.1 Hz, 2H), 7.26–7.19 (m, 2H), 7.14–7.06 (m, 2H), 6.97 (dd, *J* = 7.7, 1.6 Hz, 2H), 2.46–2.32 (m, 4H), 1.72–1.60 (m, 4H), 1.56 (s, 6H), 1.46–1.35 (m, 4H), 1.25 (td, *J* = 5.2 Hz, 2H), 0.64 (s, 6H), 0.49 (s, 6H); ^13^C NMR (75 MHz, CDCl_3_, 25 °C, TMS) δ 143.20, 141.37, 136.14, 128.83, 125.06, 124.63, 94.59, 55.33, 50.19, 48.39, 44.06, 35.91, 28.89, 23.47, 20.80, 20.74; ^29^Si NMR (60 MHz, CDCl_3_, 25 °C, TMS) δ −21.92.

**Synthesis of BIFOXSiCl(OH) (8):** In a dried Schlenk flask BIFOXSiCl_2_ (**7**, 1 g, 1.8 mmol, 1 equiv) was solved in THF (10 mL). Then triethylamine (0.52 mL, 3.6 mmol, 2 equiv) and H_2_O (0.032 mL, 1.8 mmol, 1 equiv) was added. The reaction mixture was stirred for 20 h at 20 °C and concentrated in vacuo. The residue was purified by silica gel flash column chromatography (*n-*hexane/ethyl acetate: 9:0.5, *R*_f_: 0.43) which resulted in BIFOXSiCl(OH) (**8**, 642.6 mg, 1.2 mmol, 67%) as a white solid and BIFOXSi(OH)_2_ (**9**, 90.6 mg. 0.1 mmol. 6%) as a white solid; mp 105.7–112.5 °C; [α]_589_^20^ = −3.24 (*c* 0.658, CHCl_3_); ^1^H NMR (300 MHz, CDCl_3_, 25 °C, TMS) δ 7.73 (d, *J* = 8.0 Hz, 1H), 7.61 (dd, *J* = 7.6, 1.7 Hz, 1H), 7.35–7.17 (m, 4H), 7.03 (t, *J* = 7.3 Hz, 1H), 6.72 (dd, *J* = 7.8, 1.5 Hz, 1H), 2.66–2.48 (m, 2H), 2.40 (s, 1H), 2.31 (d, *J* = 10.5 Hz, 1H), 1.72 (s, 3H), 1.61 (s, 5H), 1.52 (s, 3H), 1.51–1.20 (m, 6H), 0.90 (s, 3H), 0.82 (s, 3H), 0.45 (s, 3H), 0.08 (s, 3H); ^13^C NMR (75 MHz, CDCl_3_, 25 °C, TMS) δ 145.10, 144.39, 142.08, 140.14, 137.33, 133.86, 129.25, 129.05, 128.23, 125.63, 125.25, 124.60, 123.70, 92.83, 92.25, 56.38, 54.22, 50.96, 49.57, 49.31, 46.78, 43.87, 43.78, 35.80, 35.02, 29.45, 28.36, 23.86, 23.42, 22.28, 21.49, 20.63, 19.33, 19.18; ^29^Si NMR (60 MHz, CDCl_3_, 25 °C, TMS) δ −21.93; MS (HRMS ESI) *m*/*z*: [M + Na]^+^ calcd for C_32_H_41_O_3_ClNaSi, 559.2405; found, 559.2404 (−0.1 ppm).

**Synthesis BIFOXSi(OH)_2 _(9):** In a dried Schlenk flask BIFOXSiCl_2_ (**7**, 1 g, 1.8 mmol, 1 equiv) was solved in THF (25 mL) and H_2_O (25 mL). The solution was heated to reflux and stirred overnight. Then the solution was concentrated in vacuo and purified by silica gel flash column chromatography (*n-*hexane/ethyl acetate 9:1, *R*_f_: 0.26). BIFOXSi(OH)_2_ (**9**) was obtained as white solid (0.78 g, 1.5 mmol, 84%); mp 199.4 °C; [α]_589_^20^ = 24.02 (*c* 0.769, CHCl_3_); ^1^H NMR (300 MHz, CDCl_3_, 25 °C, TMS) δ 7.60 (d, *J* = 8.0 Hz, 2H), 7.20 (td, *J* = 7.8, 1.7 Hz, 2H), 7.07 (td, *J* = 7.5, 1.1 Hz, 2H), 6.95 (dd, *J* = 7.7, 1.5 Hz, 2H). 2.48–2.19 (m, 4H), 2.03 (d, *J* = 2.5 Hz, 2H), 1.70–1.57 (m, 4H), 1.49 (s, 6H), 1.43–1.30 (m, 4H), 1.18 (td, *J* = 12.5, 4.8 Hz, 2H), 0.59 (s, 6H), 0.45 (s, 6H); ^13^C NMR (75 MHz, CDCl_3_, 25 °C, TMS) δ 144.28, 141.97, 135.16, 128.80, 124.70, 124.17, 90.24, 55.04, 50.08, 48.11, 43.98, 35.33, 29.01, 23.65, 20.85, 19.76; MS (HRMS ESI) *m*/*z*: [M + Na]^+^ calcd for C_32_H_42_O_4_NaSi, 541.2744; found, 541.2742 (−0.4 ppm); ^29^Si NMR (60 MHz, CDCl_3_, 25 °C, TMS) δ −21.92.

**General procedure for the hydrolysis studies of dichlorosilanes 7, 13 and 14:** Dichlorosilane (0.09 mmol) was solved in THF (2.5 mL) or THF/H_2_O (1.25 mL/1.25 mL). For THF/H_2_O/KOH conditions, KOH (0.9 mmol, 50.5 mg, 10 equiv) was added. The reaction mixture was heated and stirred as stated. After the reaction time the mixture was extracted two times with diethyl ether (2 mL) and concentrated in vacuo. The residue was solved in THF (5 mL). A sample (0.5 mL) was transferred to a GC vial and *n-*tetradecane solution (0.01 M in THF, 0.5 mL) was added as standard for GC analysis.

**General procedure for the *N*-acyl Mannich reaction of isoquinolin 16 with silyl ketene acetals 11 to product 12:** In a heat dried Schlenk tube isoquinolin (**16**, 11 μL, 0.1 mmol, 1 equiv) was solved in solvent (4 mL) and cooled to 0 °C under inert gas atmosphere. To this solution 2,2,2-trichlorethoxycarbonyl chloride (15 μL, 0.11 mmol, 1.1 equiv) was added. The cooling was removed. The solution warmed to 20 °C and stirred for 30 min. After this the solution was cooled to reaction temperature. The catalyst was added and stirred for 10 minutes. Then silyl ketene acetall **11** (0.15 mmol, 1.5 equiv) was added and the reaction mixture stirred for 6 h. The reaction was quenched by adding NaOMe (0.2 mL, 0.5 M in MeOH), filtered through silica gel with ethyl acetate as eluent and concentrated in vacuo. After further purification by silica gel flash column chromatography (*n-*hexane/ethyl acetate 95:5) product **12** was obtained. The enantiomeric excess is determined by chiral HPLC analysis (see Supporting Information File 1).

**General procedure for addition of silyl ketene acetals 11 to 1-chloroisochroman (18) to product 19:** In a heat dried Schlenk tube 1-chloroisochroman (**18**, 0.15 mmol, 0.3 mL of 0.5 M in toluene) was solved in solvent (1.2 mL) under inert gas atmosphere and cooled to −60 °C. After this catalyst (0.03 mmol, 0.2 equiv) was added and stirred for 10 min. Then silyl ketene acetal **11** (0.22 mmol, 1.5 equiv) was added and the resulting reaction mixture was stirred for 6 h. The reaction was quenched by adding NaOMe (0.2 mL, 0.5 M in MeOH), concentrated in vacuo and purified by silica gel flash column chromatography (*n-*hexane/Et_2_O 9:1). The enantiomeric excess is determined by chiral HPLC analysis (see Supporting Information File 1).

**General procedure for addition of silyl ketene acetals 11 to chromone 20 to product 22:** In a heat dried Schlenk tube chromone **20** (14.6 mg, 0.1 mmol, 1 equiv) was solved in 2 mL dried toluene under inert gas atmosphere. TIPSOTf (29.5 μL, 0.11 mmol, 1.1 equiv) was added and heated to 60 °C for 1 h. After this, the reaction mixture was cooled to −80 °C, catalyst (0.02 mmol, 0.2 equiv) and silyl ketene acetal **11** (0.14 mmol, 1.25 equiv), solved in 2 mL dried toluene, were added. The resulting reaction mixture was stirred for 4 h. The reaction was quenched by adding 3 M HCl (0.2 mL), concentrated in vacuo and purified by silica gel flash column chromatography (*n-*hexane/ethyl acetate 9:1). The enantiomeric excess is determined by chiral HPLC analysis (see Supporting Information File 1).

## Supporting Information

File 1 Copies of all NMR spectra, HPLC graphs, GC graphs of the kinetic study.
