# Supplementary material for: Silanediol versus chlorosilanol: hydrolyses and hydrogen-bonding catalyses with fenchole-based silanes
Source: Beilstein J Org Chem. 2019 Jan 18;15:167–86. doi: 10.3762/bjoc.15.17 (PMC6350884; doi:10.3762/bjoc.15.17)
Supplement: File 1 — Copies of all NMR spectra, HPLC graphs, GC graphs of the kinetic study. [file Beilstein_J_Org_Chem-15-167-s001.pdf]

BEILSTEIN JOURNAL OF ORGANIC CHEMISTRY

# Supporting Information

for

# Silanediol versus chlorosilanol: hydrolyses and hydrogen-bonding catalyses with fenchole-based silanes

Falco Fox, Jörg M. Neudörfl and Bernd Goldfuss

*Beilstein J. Org. Chem.* **2019,** *15,* 167–186. doi:10.3762/bjoc.15.17

# Copies of all NMR spectra, HPLC graphs, GC graphs of the kinetic study

License and Terms: This is a supporting information file under the terms of the Creative Commons Attribution License (http://creativecommons.org/licenses/by/4.0). Please note that the reuse, redistribution and reproduction in particular requires that the authors and source are credited.
The license is subject to the *Beilstein Journal of Organic Chemistry* terms and conditions: (https://www.beilstein-journals.org/bjoc)

*Table of Contents*


1. General methods .......... S2

2. Dichlorosilanes, chlorosilanol and silanediol preparation .......... S2

3. General procedure for the preparation of silyl ketene acetals .......... S4

4. General procedure for the catalytic reactions .......... S6

5. General procedure for the kinetic hydrolysis .......... S11

6. General procedure for UV–vis titration .......... S17

7. Computational details .......... S22

   7.1. BIFOXSi derivatives .......... S22
   7.2. Kondo-silanes .......... S42
   7.3. glycol-silanes .......... S50
   7.4. tetrachlorosilane .......... S52
   7.5. NBO geometries .......... S56

8. X-ray details .......... S69

9. Selected NMR spectra .......... S75

   9.1. $BIFOXSiCl_2$ .......... S75
   9.2. BIFOXSiCl(OH) **8** .......... S77
   9.3. $BIFOXSi(OH)_2$ **9** .......... S79
   9.4. Kondo-$SiCl_2$ **13** .......... S81
   9.5. $KondoSi(OH)_2$ **1** .......... S83
   9.6. dichlorobis(2,4,6-tri-*tert*-butylphenoxy)silane **14** .......... S85
   9.7. bis(2,4,6-tri-*tert*-butylphenoxy)silandiol **15** .......... S87

10. References .......... S89

1. **General methods**

Diethyl ether, methyl *tert*-butyl ether, *n*-hexane and tetrahydrofuran were dried over sodium and fresh distilled before use. Methylene chloride was dried over phosphorus pentoxide and fresh distilled before use. Toluene, 1,2-DCE, dimethylformamide, acetonitrile, acetone, benzene, *m*-xylene, nitrobenzene and pyridine were purchased dry and used as received. Purification of reaction products was carried out by flash chromatography using silica gel (0.035–0.070 mm, 60 A). Analytical thin layer chromatography was performed on Merck silica gel 60 $F_{254}$ plates. Visualization was accomplished with UV light (254 nm, 330 nm) and potassium permanganate stains followed by heating. Melting points (mp) were obtained on a Stuart Scientific SMP 3 and are uncorrected. Proton nuclear magnetic resonances ($^1$H NMR) were recorded in deuterated solvents on a Bruker Avance AV Avance II (300 MHz) spectrometer unless otherwise noted. Chemical shifts are reported in parts per million (ppm, δ) using the solvent as internal standard. $^1$H NMR splitting patterns are designated as singlet (s), doublet (d), triplet (t), or quartet (q). Splitting patterns that could not be interpreted or easily visualized are designated as multiplet (m). Coupling constants are reported in Hertz (Hz). Proton-decoupled carbon ($^{13}$C NMR) spectra were recorded on a Bruker AV Avance II (300 MHz). Chemical shifts are reported in parts per million (ppm, δ) using the solvent as internal standard. Electrospray mass spectra (ESIMS) were obtained using a THERMO Scientific LTQ Orbitrap XL. HPLC analyses were obtained on a VWR Hitachi L-2130 (Pump) and VWR Hitachi L-2400 (UV Detector) equipped with a VWR Hitachi Chromaster 5310 (Column Oven). GC analyses were obtained on a Hewlett Packard 6890 GC-System. UV–vis spectra were obtained on a Perkin-Elmer UV–vis-Spectrometer Lambda 35, using quartz cuvettes with 1.00 cm width. Specific rotations were obtained on an Anton Paar MCP 200 polarimeter. X-ray structure analyses were obtained on a Bruker D8 Venture with KAPPA goniometer and a cupper microfocus source.

2. **Dichlorosilanes, chlorosilanol and silanediol preparation**

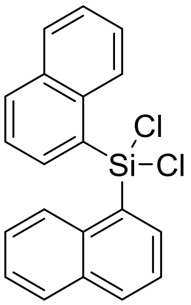


**Synthesis of dichlorodi(naphthalen-1-yl)silane (13):** In a dried round bottomed flask 1-bromonaphthalene (3.4 mL, 24.4 mmol, 1 equiv) was solved in 100 mL dried diethyl ether an cooled to -80 °C. Afterwards *n*-BuLi (2.5 *M* in hexane, 10.7 mL, 1.2 equiv) was added and stirred for 30 min at this temperature. In another flask, $SiCl_4$ (1.4 mL, 12.2 mmol, 0.5 equiv) was solved in 20 mL dried diethyl ether. That solution

was added drop wise to the 1-bromonaphthalen/*n*BuLi solution at -80 °C. The mixture was warmed to 20 °C and stirred for 2 d. The solvent was evaporated, the residue was solved in toluene and filtered through celite. After evaporation of the solvent, dichlorodi(naphthalen-1-yl)silane **13** was obtained as white solid (1.22 g, 3.45 mmol 14%). M.p.138.3°C; $^{1}$H NMR (300 MHz, Chloroform-d): δ=8.29 (d, J = 6.9 Hz, 2H), 8.14 (d, J = 5.2 Hz, 2H), 8.06 (d, J = 8.2 Hz, 2H), 7.92 (d, J = 7.8 Hz, 2H), 7.59 – 7.40 (m, 6H); $^{13}$C NMR (75 MHz, CDCl3): δ=136.11, 135.43, 133.59, 133.07, 129.61, 129.18, 127.91, 126.85, 126.22, 125.03, 109.99.

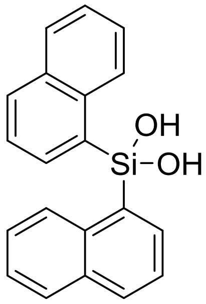


**Synthesis of di(naphthalen-1-yl)silanediol (1):** In a dried round bottomed flask dichlorodi(naphthalen-1-yl)silane **13** (0.5 g, 1.4 mmol, 1 equiv) was solved in 10 mL dried THF. To this solution, 5 mL water was added. This mixture was stirred for 2 h at 20 °C. The solvent was evaporated and the residue purified by silica gel flash column chromatography (*n*hexane/ethyl acetate: 4/1, $R_f$: 0.18). Di(naphthalen-1-yl)silanediol **1** was obtained as white solid (0.433 g, 1.3 mmol, 98%). M.p. 153.1°C; $^{1}$H NMR (300 MHz, Chloroform-*d*): δ=8.28 (d, *J* = 8.2 Hz, 2H), 7.85 (dd, *J* = 18.7, 7.7 Hz, 6H), 7.46 – 7.27 (m, 6H), 3.74 (s, 2H); $^{13}$C NMR (75 MHz, $CDCl_3$) δ 135.61, 134.56, 132.25, 131.56, 130.21, 127.79, 127.35, 125.35, 124.63, 124.09.

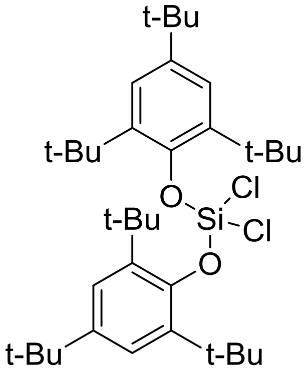


**Synthesis of dichlorobis(2,4,6-tri-*tert*-butylphenoxy)silane (14):** In a dried round bottomed flask potassium (1.0 g, 25.6 mmol, 3 equiv) was added to 70 mL dried toluene and heated to reflux for 2 2,4,6-tri-*tert*-butylphenol (6 g, 25.6 mmol, 3 equiv) was solved in 70 mL dried diethyl ether and added to the potassium/toluene

suspension. This mixture was heated to reflux for 24 h. After that, a solution of $SiCl_4$ (1.0 mL, 8.4 mmol, 1 equiv) and 16 mg 18-Crown-6 in 3 mL diethyl ether was added. This mixture was heated do reflux for 16 h. The diethyl ether was evaporated and the remaining mixture was heated to reflux for another 24 h. The solvent was evaporated, the residue solved in *n*-hexane and filtered through celite. After recrystallization with hexane, dichlorobis(2,4,6-tri-*tert*-butylphenoxy)silane (**14**) was obtained as white solid (0.97 g, 1.56 mmol, 19%). M.p. 205.6°C; $^1$H NMR (300 MHz, chloroform-*d*): δ=7.29 (s, 4H), 1.50 (s, 36H), 1.31 (s, 18H).$^{13}$C NMR (75 MHz, $CDCl_3$): δ=148.17, 144.51, 140.37, 123.35, 35.75, 34.47, 32.17, 31.50.

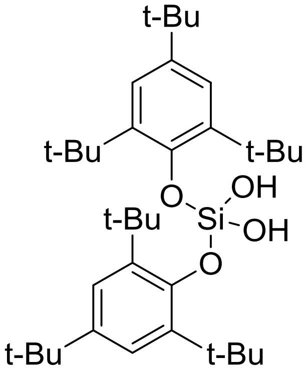


**Synthesis of bis(2,4,6-tri-*tert*-butylphenoxy)silandiol (15):** In a dried round bottomed flask dichlorobis(2,4,6-tri-*tert*-butylphenoxy)silane (**14**, 310.9 mg, 0.5 mmol,1 equiv) and KOH (280 mg, 5 mmol, 10 equiv) were solved in 13 mL $H_2O$ and 13 mL abs. THF. This mixture was heated to reflux for 16 h and extracted with diethyl ether thrice. The organic layer was dried over $Na_2SO_4$ and concentrated on a rotary evaporator. Bis(2,4,6-tri-*tert*-butylphenyl)silandiol **15** was obtained as white solid (289 mg, 0.49 mmol, 99%). M.p. 196.3°C; $^1$H NMR (300 MHz, chloroform-*d*): δ=7.21 (s, 4H), 5.03 (s, 2H), 1.45 (s, 36H), 1.30 (s, 18H); $^{13}$C NMR (75 MHz, $CDCl_3$):δ=151.33, 141.40, 134.93, 121.88, 31.73, 30.43.

Di-*tert*-butoxydichlorosilane and di-*tert*-butoxysilandiol were obtained as reported in literature[1]. While concentrating on a rotary evaporator at 40 °C, the colorless oil turns to a white solid as well. With this behavior further kinetic experiments were discarded.

3. **General procedure for the preparation of silyl ketene acetals**

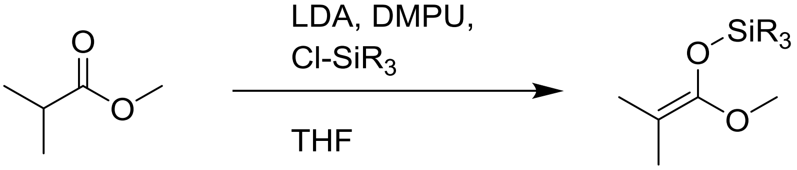


In a dried 250 mL round bottomed flask, di-*iso*propylamine (4.2 mL, 30 mmol, 1.2 equiv) was added to 50 mL anhydrous THF. The solution was cooled to 0 °C and *n*-

BuLi (11 mL, 27.5 mmol, 2.5 *M* in hexane, 1.1 equiv) was added dropwise. The mixture was stirred at 0 °C for 20 min. After that, the mixture was cooled to -78 °C and methyl isobutyrate (2.87 mL, 25 mmol, 1.0 equiv) was added slowly. The mixture was stirred at -78 °C for 30 min. Than DMPU (4.53 mL, 37.5 mmol, 1.2 equiv) and the respective chlorosilane (30 mmol, 1.2 equiv) were added. The mixture was stirred at −78 °C for 30 min and at 20 °C for 1 h. Afterwards, the solvent was removed under reduced pressure and the resulting mixture was taken up in 200 mL pentane, washed with water (1 × 100 mL), saturated $CuSO_4$ solution (1 × 100 mL), saturated $NaHCO_3$ solution (1 × 100 mL) and saturated NaCl solution (1 × 100 mL). The organic layer was dried over $Na_2SO_4$, concentrated on a rotary evaporator and purified via fractional distillation.

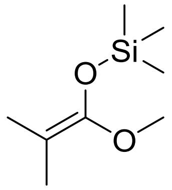

**Synthesis of ((1-methoxy-2-methylprop-1-en-1-yl)oxy)trimethylsilane (11a):** Chlorotrimethylsilane (3.77 mL, 30 mmol, 1.2 equiv) was added. After distillation, the product was obtained as clear colorless oil (2.25 g, 13.8 mmol, 55%). B.p: 47-49 °C (20 mbar), $^{1}$H NMR (300 MHz, chloroform-*d*): δ=3.50 (s, 3H), 1.57 (s, 3H), 1.51 (s, 3H), 0.20 (s, 9H); $^{13}$C NMR (75 MHz, $CDCl_3$) δ 90.87, 56.51, 16.84, 16.10, 0.01.

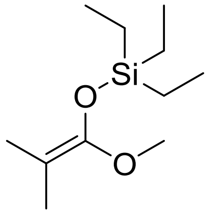

**Synthesis of ((1-methoxy-2-methylprop-1-en-1-yl)oxy)triethylsilane (11b):** Chlorotriethylsilane (5.08 mL, 30 mmol, 1.2 equiv) was added. After distillation, the product was obtained as clear colorless oil (3.09 g, 14.3 mmol, 57%). B.p: 57-59 °C (1.6 mbar). $^{1}$H NMR (300 MHz, chloroform-*d*): δ=3.51 (s, 3H), 1.55 (s, 3H), 1.53 (s, 3H), 0.99 (t, $J$ = 7.9 Hz, 9H), 0.69 (d, $J$ = 8.6 Hz, 6H); $^{13}$C NMR (75 MHz, $CDCl_3$): δ=90.93, 57.03, 16.79, 16.13, 6.54, 4.94.

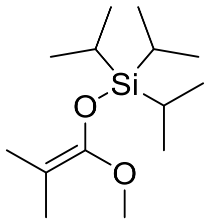

**Synthesis of ((1-methoxy-2-methylprop-1-en-1-yl)oxy)tri*iso*propylsilane (11c):** Chlorotri*iso*propylsilane (6.42 mL, 30 mmol, 1.2 equiv) was added. After distillation, the product was obtained as clear colorless oil (5.49 g, 14.3 mmol, 70%). B.p: 74 °C (0.4 mbar). $^{1}$H NMR (300 MHz, chloroform-*d*): δ=3.56 (s, 3H), 1.57 (s, 6H), 1.14 –

1.07 (m, 21H); $^{13}$C NMR (75 MHz, $CDCl_3$): δ=150.75, 90.97, 58.06, 17.76, 17.02, 16.27, 12.74.

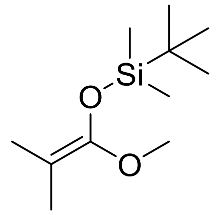

**Synthesis of *tert*-butyl((1-methoxy-2-methylprop-1-en-1-yl)oxy)dimethylsilane (11d):** *tert*-butylchlorodimethylsilane (4.52 g, 30 mmol, 1.2 equiv) was added. After distillation, the product was obtained as clear colorless oil (2.86 g, 13.0 mmol, 52%). B.p: 48-50 °C (1.8 mbar). $^{1}$H NMR (300 MHz, chloroform-*d*): δ=3.51 (s, 3H), 1.57 (s, 3H), 1.53 (s, 3H), 0.96 (s, 9H), 0.14 (s, 6H); $^{13}$C NMR (75 MHz, $CDCl_3$): δ=91.51, 77.16, 57.19, 25.86, 17.03, 16.39, -4.47.

4. **General procedure for the catalytic reactions**

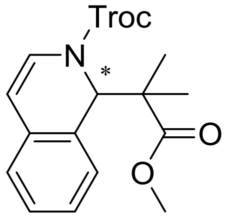

**General procedure for the *N*-acyl Mannich reaction of isoquinolin 16 with silyl ketene acetals 11 to product 12:** In a heat dried Schlenk tube isoquinolin **16** (11 µl, 0.1 mmol, 1 equiv) was solved in solvent (4 mL) and cooled to 0 °C under inert gas atmosphere. To this solution 2,2,2-trichlorethoxycarbonyl chloride (15 µL, 0.11 mmol, 1.1 equiv) was added. The cooling was removed. The solution warmed to 20 °C and stirred for 30 min. After this the solution was cooled to reaction temperature. The catalyst was added and stirred for 10 minutes. Then silyl ketene acetal **11** (0.15 mmol, 1.5 equiv) was added and the reaction mixture stirred for 6 h. The reaction was quenched by adding NaOMe (0.2 mL, 0.5 M in MeOH), filtered through silica gel with ethyl acetate as eluent and concentrated in vacuo. After further purification by silica gel flash column chromatography (*n*-hexane/ethyl acetate 95:5) product **12** was obtained. The enantiomeric excesses is determined by chiral HPLC analysis OD H, *n*-hexane/*i*PrOH 90/10, 1 mL/min, 220 nm, 25°C, $t_r$: 5.8 min (R) , $t_r$: 6.5 min (S), $[\alpha]^{20}_{D}$= -10.61 ° for 5% ee (c=1.1g/100 mL, (-)**12** correlates to **S-12**[2]); $^{1}$H NMR (300 MHz, Chloroform-*d*): δ=7.33 – 7.17 (m, 2H), 7.10 (t, *J* = 6.3 Hz, 2H), 6.98 (s, 1H), 6.02 (d, *J* = 30.9, 7.7 Hz, 1H), 5.78 (s, 1H), 4.86 (d, 2H), 3.64 (s, 3H), 1.27 (s, 3H), 1.14 (s, 3H). NMR spectra contain the data set of two rotameres of **12**.

**rac-12**

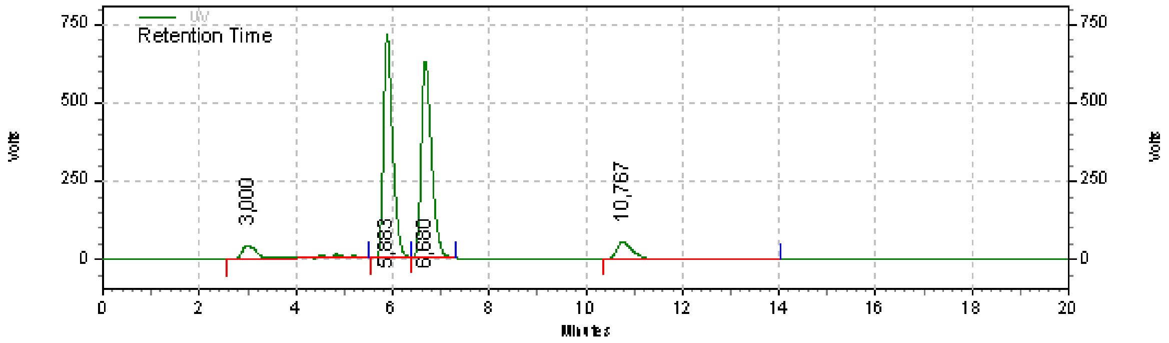


**UV Results**

| Retention Time | Area | Area % | Height | Height % |
|---|---|---|---|---|
| 3,000 | 5540425 | 6,47 | 167367 | 2,91 |
| 5,883 | 37507028 | 43,78 | 2858708 | 49,66 |
| 6,680 | 37381215 | 43,64 | 2507394 | 43,56 |
| 10,767 | 5233162 | 6,11 | 223254 | 3,88 |
| Totals | 85661830 | 100,00 | 5756723 | 100,00 |

Table 7, entry 9

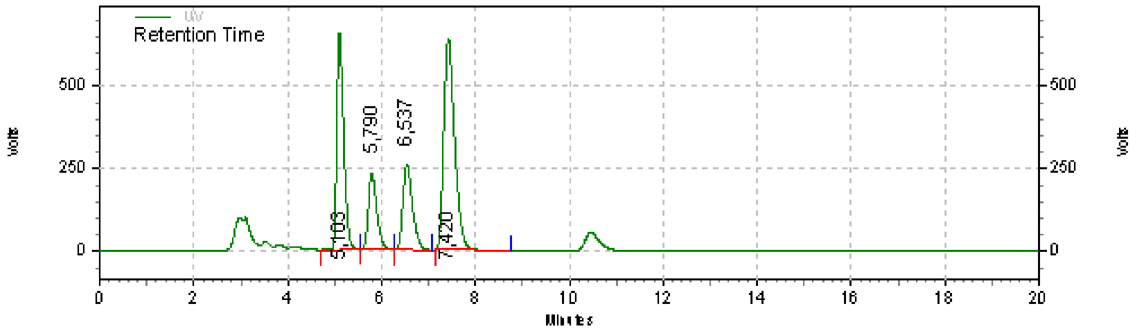


**UV Results**

| Retention Time | Area | Area % | Height | Height % |
|---|---|---|---|---|
| 5,103 | 26654293 | 28,41 | 2622781 | 36,73 |
| 5,790 | 11576902 | 12,34 | 927275 | 12,99 |
| 6,537 | 14710908 | 15,68 | 1025196 | 14,36 |
| 7,420 | 40894509 | 43,58 | 2565081 | 35,92 |
| Totals | 93836612 | 100,00 | 7140333 | 100,00 |

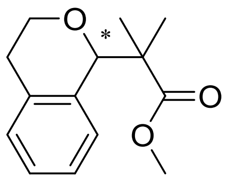

**General procedure for addition of silyl ketene acetals 11 to 1-chloroisochroman 18 to product 19:** In a heat dried Schlenk tube 1-chloroisochroman (0.15 mmol, 0.3 mL of 0.5 M in toluene) was solved in solvent (1.2 mL) under inert gas atmosphere and cooled to −60 °C. After this catalyst (0.03 mmol, 0.2 equiv) was added and stirred for 10 min. Then silyl ketene acetal **11** (0.22 mmol, 1.5 equiv) was added and the resulting reaction mixture was stirred for 6 h. The reaction was quenched by adding NaOMe (0.2 mL, 0.5 M in MeOH), concentrated in vacuo and purified by silica gel flash column chromatography (*n*-hexane/$Et_2O$ 9/1). The enantiomeric excesses is determined by chiral HPLC analysis OD H, n-hexane/*i*PrOH 100/0, 1.0 mL/min, 210 nm, 25°C, $t_r$: 23.9 min (S) , $t_r$: 29.1 min (R), $[\alpha]^{20}_{D}$= -0.63 ° for 1% ee (c=0.52g/100 mL (−)**19** correlates to ***S*-19**[3]). $^1$H NMR (500 MHz, Chloroform-*d*): δ=7.20 – 7.08 (m, 3H), 6.97 (d, *J* = 7.1 Hz, 1H), 5.18 (s, 1H), 4.15 (dddd, *J* = 14.6, 10.7, 5.2, 1.6 Hz, 1H), 3.76 (s, 3H), 3.58 (tdd, *J* = 11.8, 10.8, 2.4, 1.0 Hz, 1H), 3.00 (ddt, *J* = 16.5, 11.2, 5.1, 4.7 Hz, 1H), 2.53 (d, *J* = 15.8 Hz, 1H), 1.13 (s, 3H), 1.11 (s, 3H).

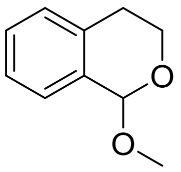

**Synthesis of 1-methoxyisochroman:** In a round bottomed flask isochromane (2.16 g, 16 mmol, 1 equiv) was solved in 100 mL DCM. methanol (0.44 mL, 10.8 mmol, 0.7 equiv) and 2,3-dichloro-5,6-dicyano-1,4-benzoquinone (2.5 g, 11.0 mmol, 0.7 equiv) were added. This mixture was stirred at room temperature for 24 h, quenched with 200 mL $NaHCO_3$ (5%). The mixture was extracted with DCM (3 × 200 mL), dried over $Na_2SO_4$ and concentrated with a rotary evaporator. The residue was purified by flash chromatography (n-hexane/ethyl acetate 10/1). 1-Methoxyisochroman was obtained as colorless liquid (1.45 g, 8.8 mmol, 55%). $^1$H NMR (300 MHz, Chloroform-*d*): δ=7.34 – 7.22 (m, 3H), 7.20 – 7.12 (m, 1H), 5.51 (s, 1H), 4.18 (td, *J* = 11.6, 3.4 Hz, 1H), 3.95 (ddd, *J* = 11.2, 6.0, 1.5 Hz, 1H), 3.60 (s, 3H), 3.07 (ddd, *J* = 17.3, 12.0, 6.0 Hz, 1H), 2.71 – 2.60 (m, 1H); $^{13}$C NMR (75 MHz, $CDCl_3$): δ=134.19, 134.06, 128.48, 128.18, 127.47, 126.34, 97.84, 57.79, 55.36, 28.02.

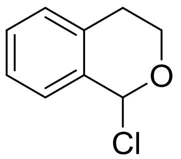

**Synthesis of 1-chloroisochroman:** In a dried Schlenk tube 1- methoxyisochroman (1.0 g, 6.1 mmol, 1 equiv) was solved in 8 mL DCM. The mixture as cooled to 0 °C

and $BCl_3$ (1 *M*, 2.44 mL, 2.44 mmol, 0.4 equiv) was added dropwise. The mixture was warmed to 20 °C and stirred for 1.5 h. The solvent was evaporated and the residue distilled. 1-chloroisochroman was obtained as colorless liquid (0.69 g, 4.1 mmol, 67%). Bp: 75-77 °C (0.5 mbar), $^1$H NMR (300 MHz, chloroform-*d*): δ=7.36 – 7.22 (m, 3H), 7.16 (d, *J* = 7.0 Hz, 2H), 4.48 – 4.29 (m, 1H), 4.19 (s, 1H), 3.29 – 3.01 (m, 1H), 2.77 (d, *J* = 15.9 Hz, 1H).

**rac-18** with just 0.7 mL flow.

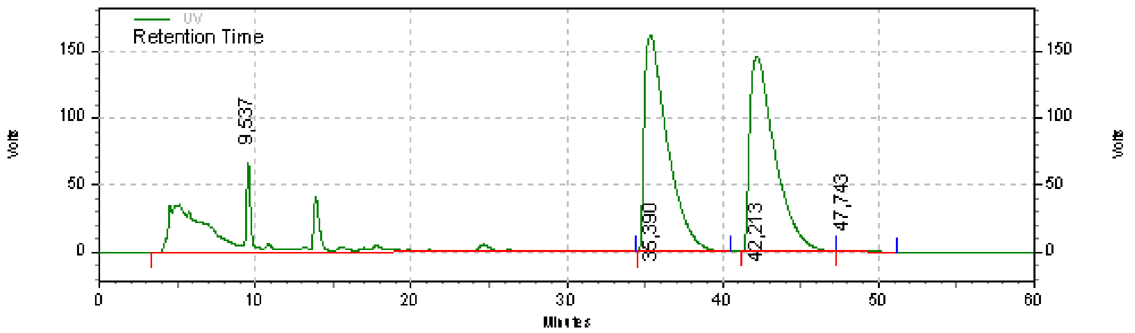


**UV Results**

| Retention Time | Area | Area % | Height | Height % |
|---|---|---|---|---|
| 9,537 | 40758355 | 23,69 | 264537 | 17,77 |
| 35,390 | 66199736 | 38,47 | 644215 | 43,27 |
| 42,213 | 64833216 | 37,68 | 578814 | 38,88 |
| 47,743 | 277506 | 0,16 | 1203 | 0,08 |
| Totals | 172068813 | 100,00 | 1488769 | 100,00 |

Table 10, entry 7

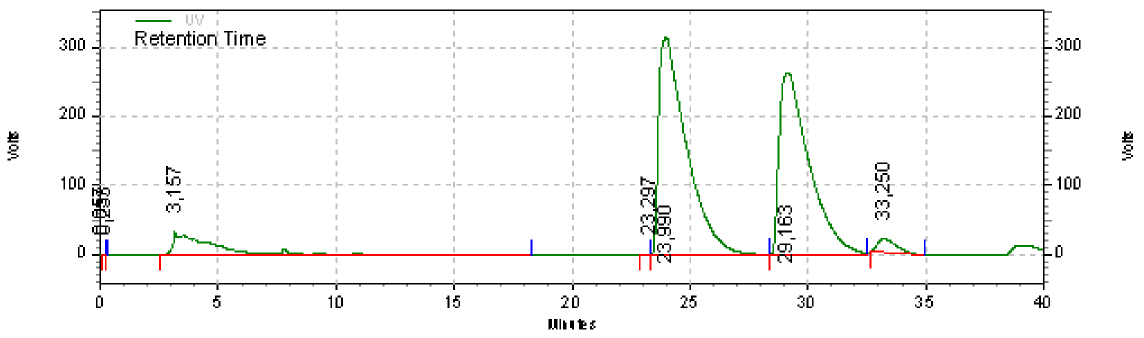


**UV Results**

| Retention Time | Area | Area % | Height | Height % |
|---|---|---|---|---|
| 0,057 | 520 | 0,00 | 94 | 0,00 |
| 0,233 | 322 | 0,00 | 85 | 0,00 |
| 3,157 | 15714805 | 7,24 | 130866 | 5,17 |
| 23,297 | 33222 | 0,02 | 1375 | 0,05 |
| 23,990 | 103230964 | 47,55 | 1261744 | 49,86 |
| 29,163 | 93668533 | 43,15 | 1057490 | 41,79 |
| 33,250 | 4452675 | 2,05 | 78696 | 3,11 |
| Totals | 217101041 | 100,00 | 2530350 | 100,00 |

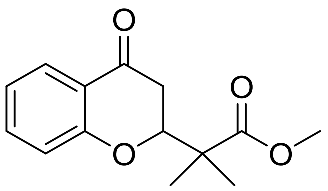

**General procedure for addition of silyl ketene acetals 11 to chromone 20 to product 22:** In a heat dried Schlenk tube chromone (14.6 mg, 0.1 mmol, 1 equiv) was solved in 2 mL dried toluene under inert gas atmosphere. TIPSOTf (29.5 µL, 0.11 mmol, 1.1 equiv) was added and heated to 60 °C for 1 h. After this, the reaction mixture was cooled to -80 °C, catalyst (0.02 mmol, 0.2 equiv) and silyl ketene acetal **19** (0.14 mmol, 1.25 equiv), solved in 2 mL dried toluene, were added. The resulting reaction mixture was stirred for 4 h. The reaction was quenched by adding 3 M HCl (0.2 mL), concentrated in vacuo and purified by silica gel flash column chromatography (*n*hexane/ethyl acetate 9:1).The enantiomeric excesses is determined by chiral HPLC analysis AD H, *n*-hexane/*i*PrOH 98/2, 1 mL/min, 254 nm, 25 °C, $t_r$: 12.1 min (R), $t_r$: 14.5 min (S), $[\alpha]^{20}_{D}$=12.17 ° for 4% ee (c=0.05g/100 mL (+)**22** correlates to **S-22**[4]). $^1$H NMR (300 MHz, chloroform-*d*): δ=7.87 (dd, *J* = 7.8, 1.5 Hz, 1H), 7.46 (d, *J* = 8.2 Hz, 1H), 7.06 – 6.89 (m, 2H), 4.65 (dd, *J* = 14.1, 2.5 Hz, 1H), 3.73 (s, 3H), 2.88 – 2.52 (m, 2H), 1.38 (s, 3H), 1.28 (s, 3H); $^{13}$C NMR (75 MHz, $CDCl_3$): δ=192.28, 175.51, 161.55, 136.00, 126.96, 121.48, 120.81, 117.88, 81.69, 52.25, 46.15, 38.36, 20.80, 20.62.

**rac-22**

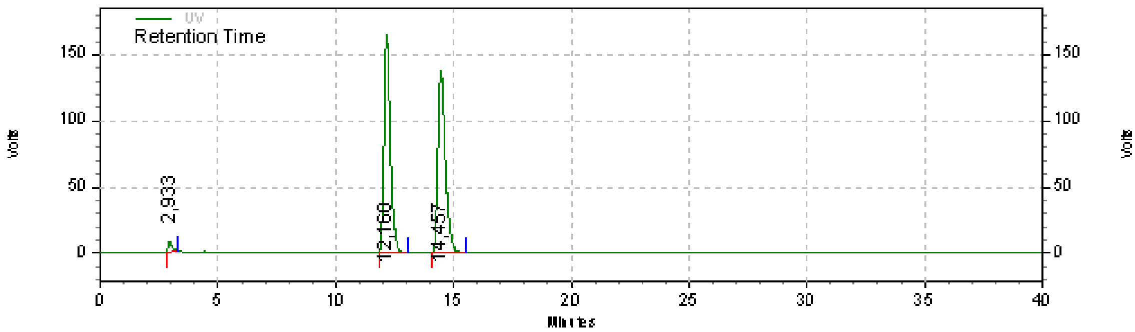


**UV Results**

| Retention Time | Area | Area % | Height | Height % |
|---|---|---|---|---|
| 2,933 | 354942 | 1,44 | 28423 | 2,31 |
| 12,160 | 12167173 | 49,29 | 658212 | 53,41 |
| 14,457 | 12161171 | 49,27 | 545679 | 44,28 |
| Totals | 24683286 | 100,00 | 1232314 | 100,00 |

Table 10, entry 2

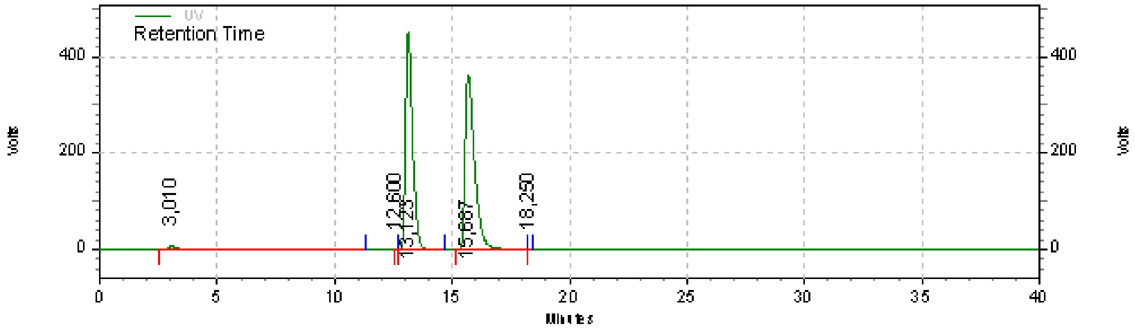


**UV Results**

| Retention Time | Area | Area % | Height | Height % |
|---|---|---|---|---|
| 3,010 | 905963 | 1,15 | 35793 | 1,09 |
| 12,600 | 3172 | 0,00 | 465 | 0,01 |
| 13,123 | 37468203 | 47,47 | 1806273 | 54,94 |
| 15,687 | 40546403 | 51,37 | 1445364 | 43,96 |
| 18,250 | 423 | 0,00 | 43 | 0,00 |
| Totals | 78924164 | 100,00 | 3287938 | 100,00 |

## 5. General procedure for the kinetic hydrolysis

**General procedure for the hydrolysis studies of dichlorosilanes 7, 13 and 14:** Dichlorosilane (0.09 mmol) was solved in THF (2.5 mL) or THF/$H_2O$ (1.25 mL/1.25 mL). For THF/$H_2O$/KOH conditions, KOH (0.9 mmol, 50.5 mg, 10 equiv) was added. The reaction mixture was heated and stirred as stated. After

reaction time the mixture was extracted two times with diethyl ether (2 mL) and concentrated in vacuo. The residue was solved in THF (5 mL). A sample (0.5 mL) was transferred to a GC vial and *n*-tetradecane solution (0.01 M in THF. 0.5 mL) was added as standard for GC analysis.

GC method: Inlet: 300 °C, carrier gas $N_2$, Split ratio 2.5/1, Flow: 4.5mL/min, column flow: 1.8 mL/min; oven: 150 °C (0m)-10 °C/min -> 200 °C (1min) – 30 °C/min -> 300 °C(20min) (Run Time 29.33 min)
column: HP-35, 30 m x 0.25 mm x 0.25 μm
For the dichlorosilanes, a calibration line was prepared according to the usual method of the internal standard. The internal standard used, was *n*-tetradecane.

| substance | $t_r$ [min] |
|---|---|
| ntetradecane | 1.9 |
| BIFOL **5** | 15.3 |
| BIFOXSi$Cl_2$ **7** | 23.2 |
| BIFOXSiCl(OH) **8** | 25.8 |
| BIFOXSi$(OH)_2$ **9** | 24.5 |
| dichlorobis(2,4,6-tri-*tert*-butylphenoxy)silane **14** | 11.4 |
| bis(2,4,6-tri-*tert*-butylphenoxy)silandiol **15** | 11.7 |

*n*-tetradecane

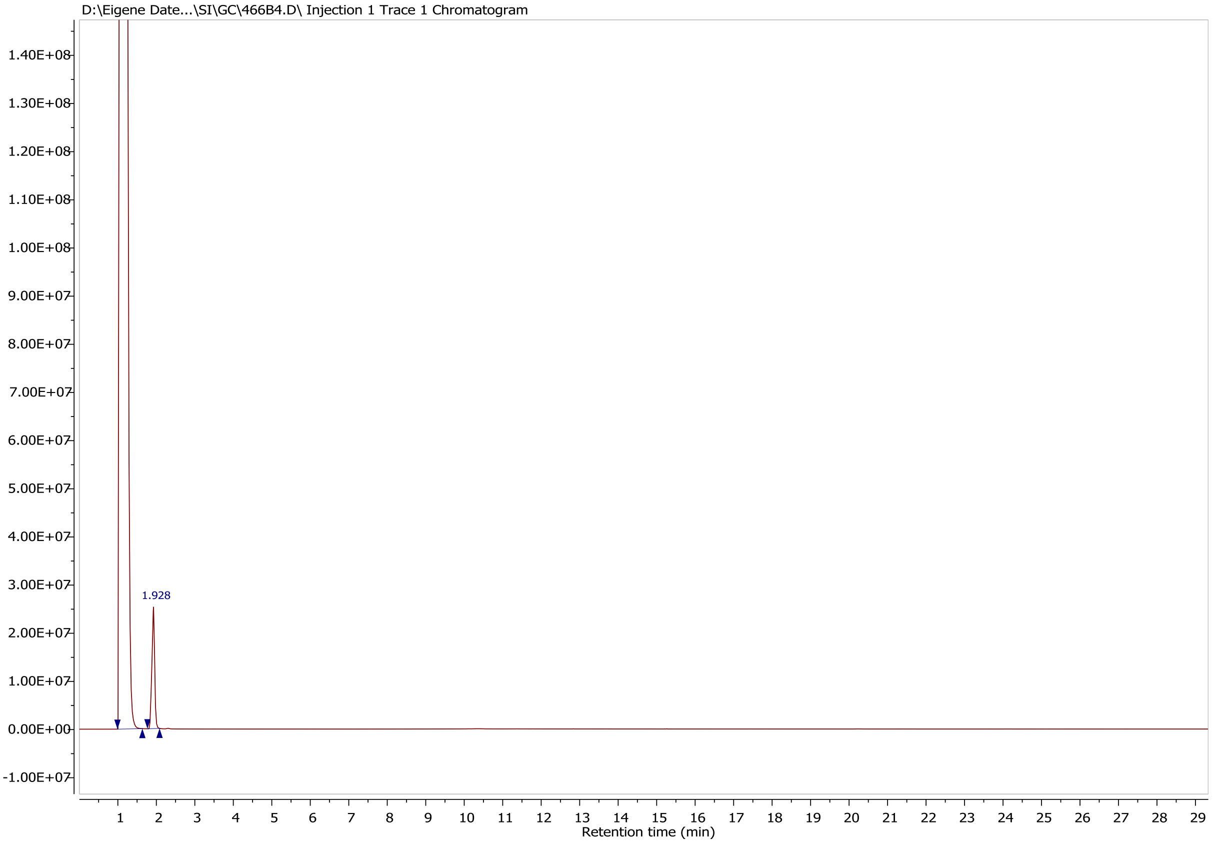

BIFOL **5**

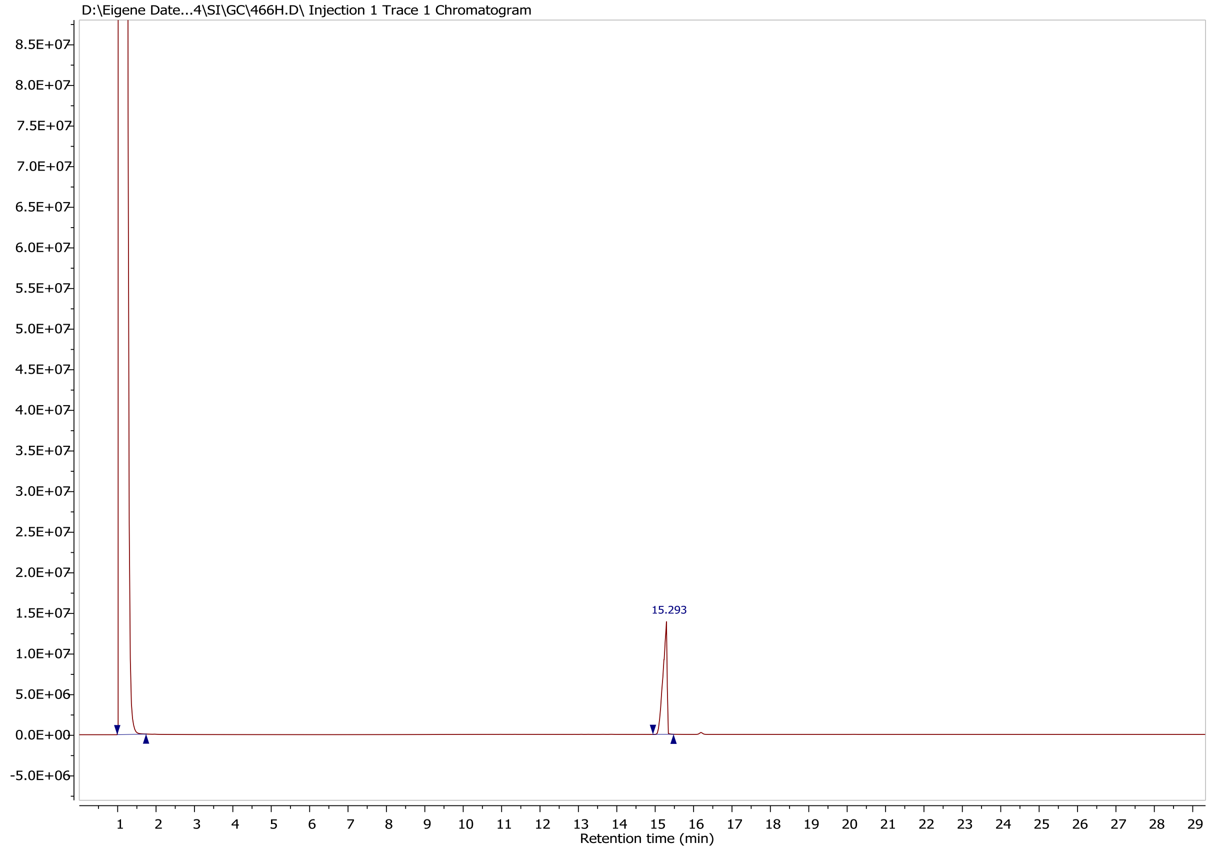


BIFOXSiCl$_2$ **7**

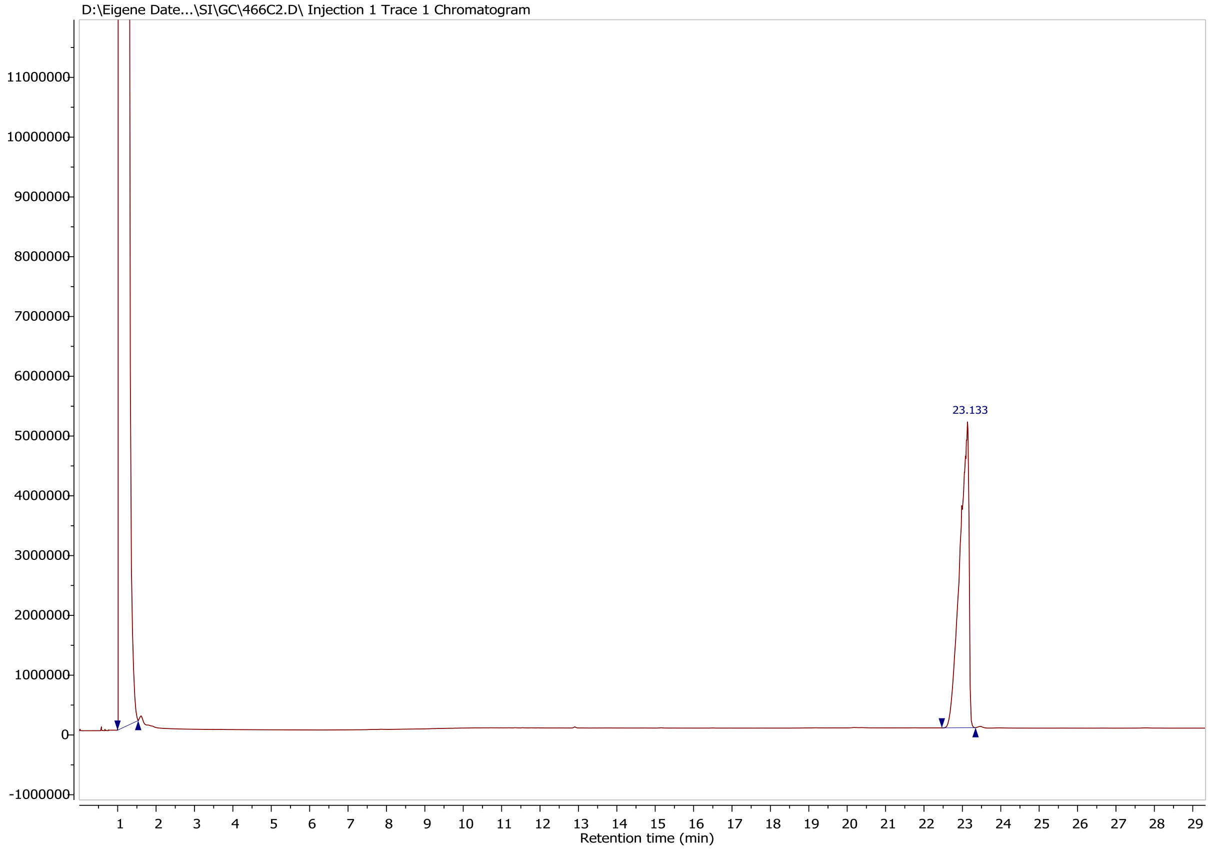

BIFOXSiCl(OH) **8**

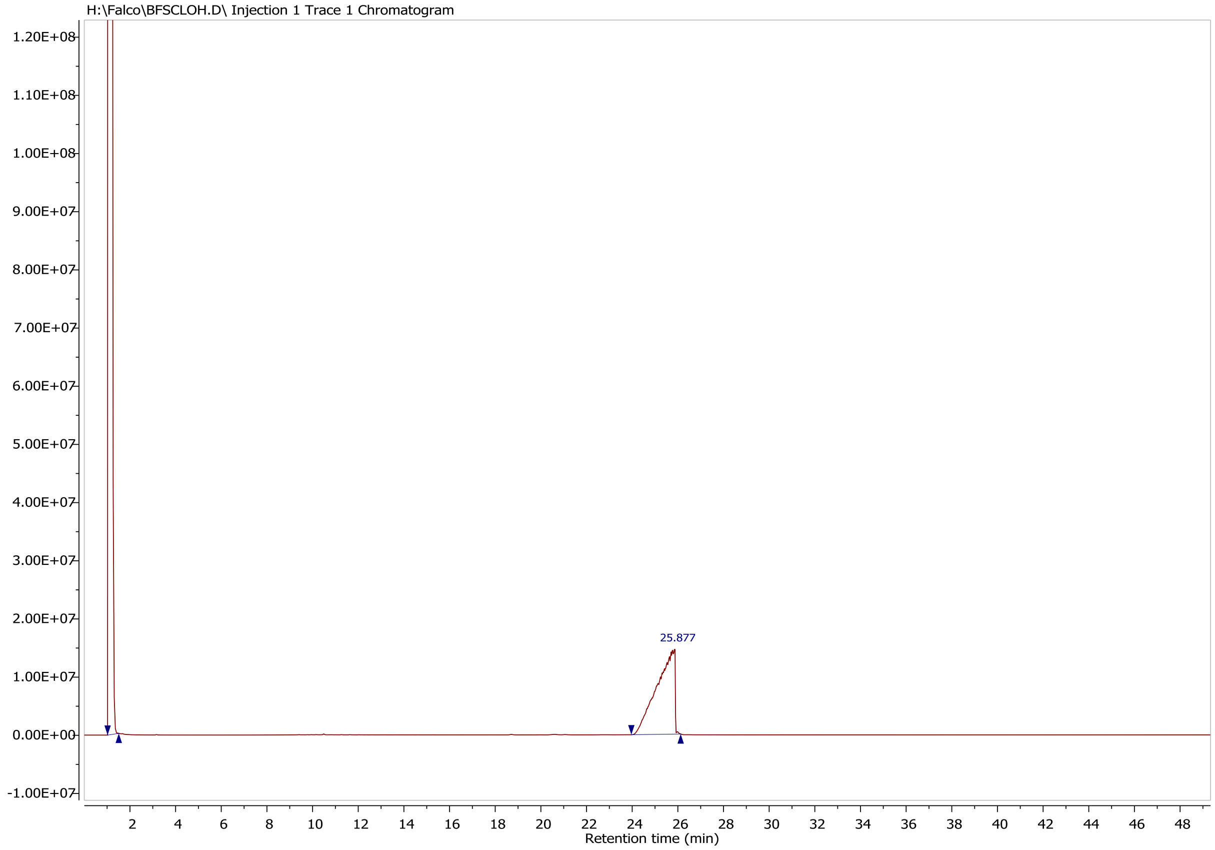


BIFOXSi(OH)$_2$ **9**

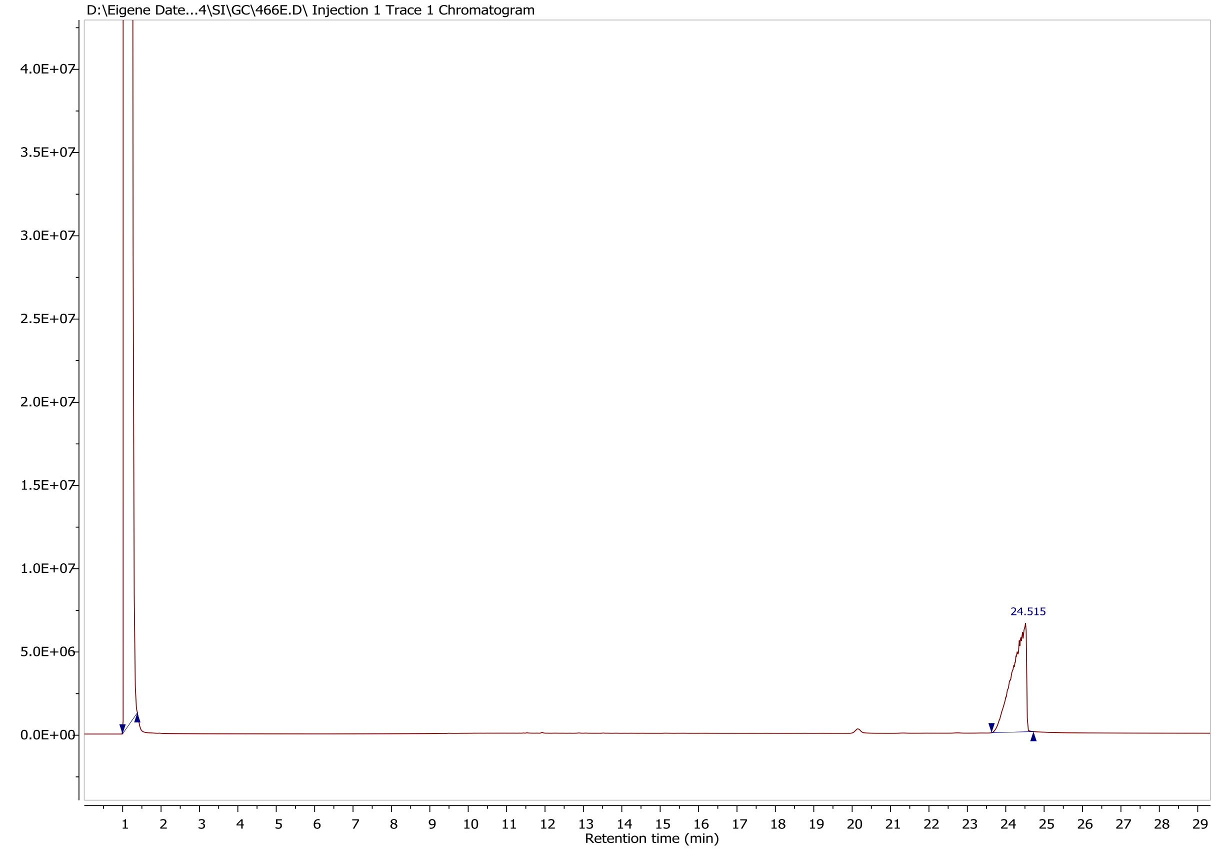

dichlorobis(2,4,6-tri-*tert*-butylphenoxy)silane (**14**)

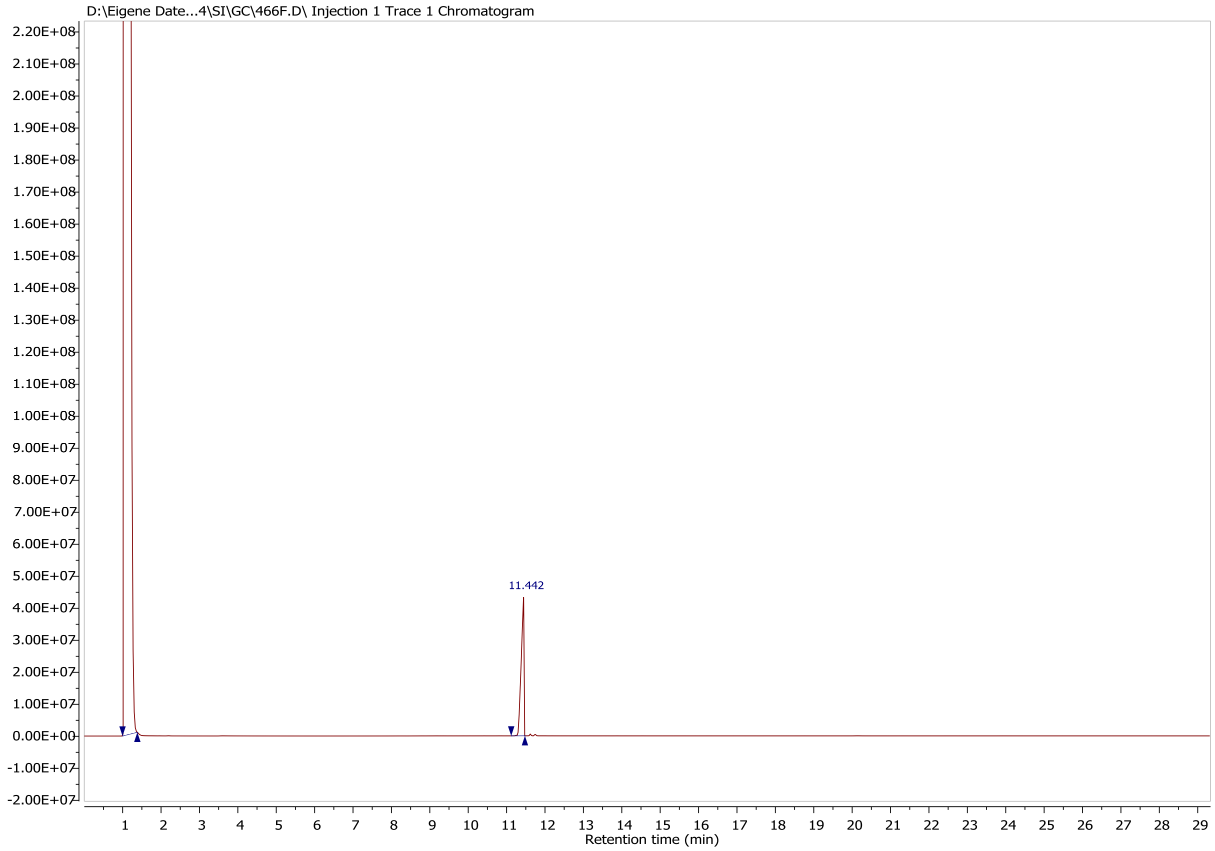


bis(2,4,6-tri-*tert*-butylphenoxy)silandiol (**15**)

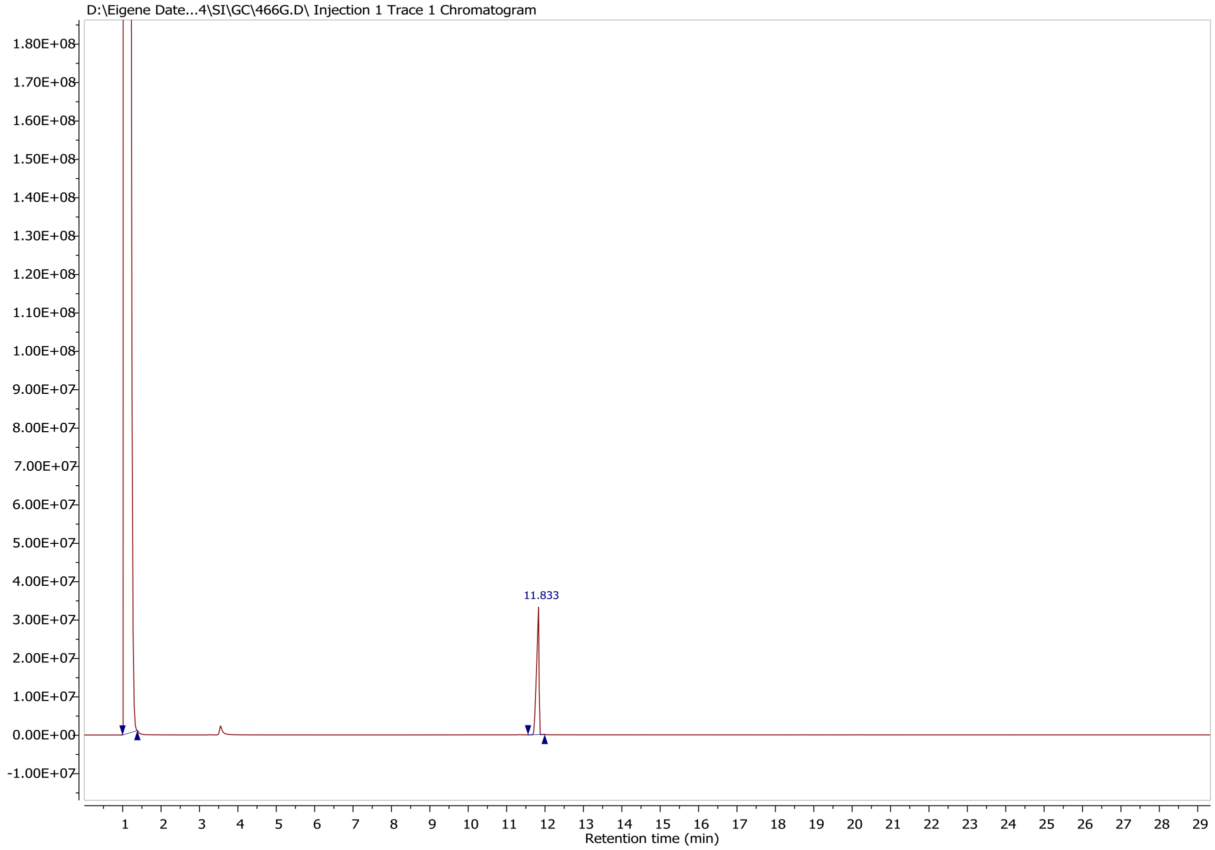

example for $BIFOXSiCl_2$ **7** to $BIFOXSi(OH)_2$ **9** in $THF/H_2O$ reflux 10h

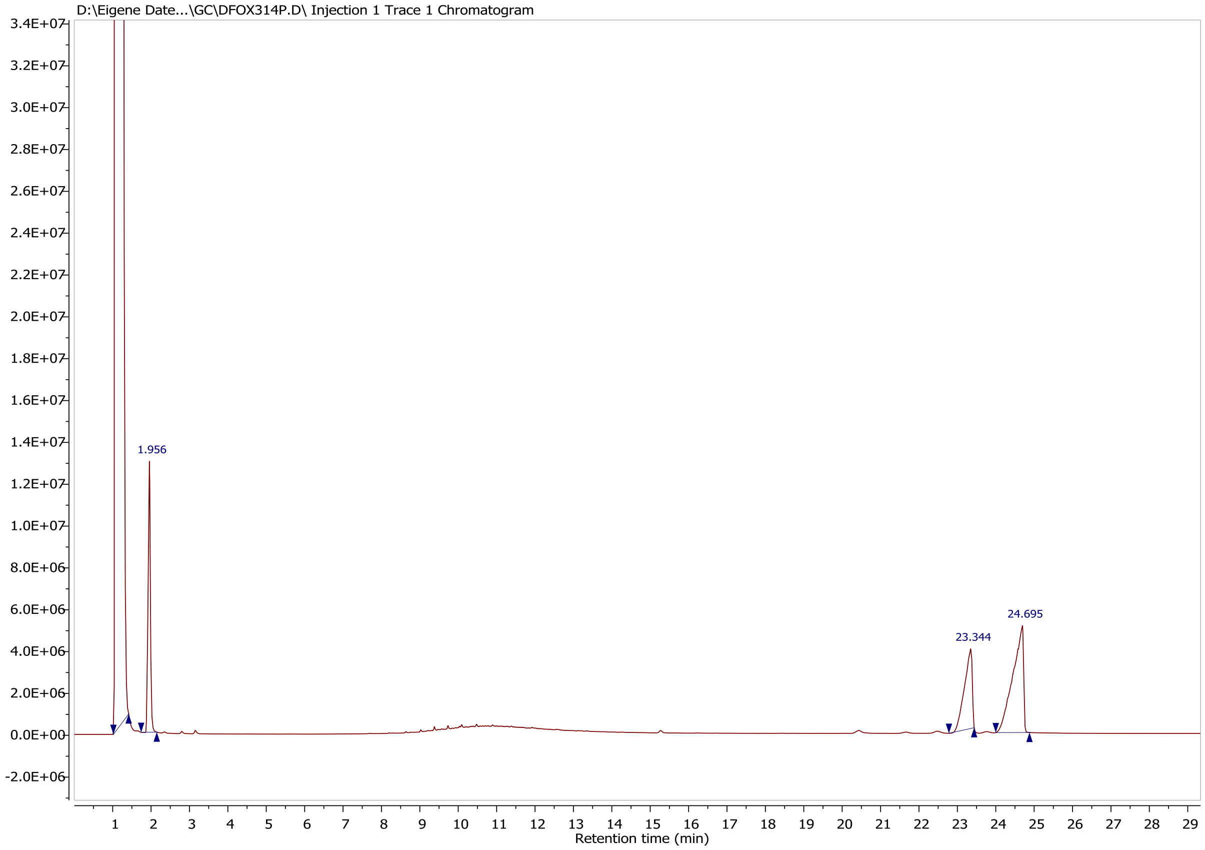


example for $BIFOXSiCl_2$ **7** to $BIFOXSi(OH)_2$ **9** in $THF/H_2O/KOH$ reflux 10 h

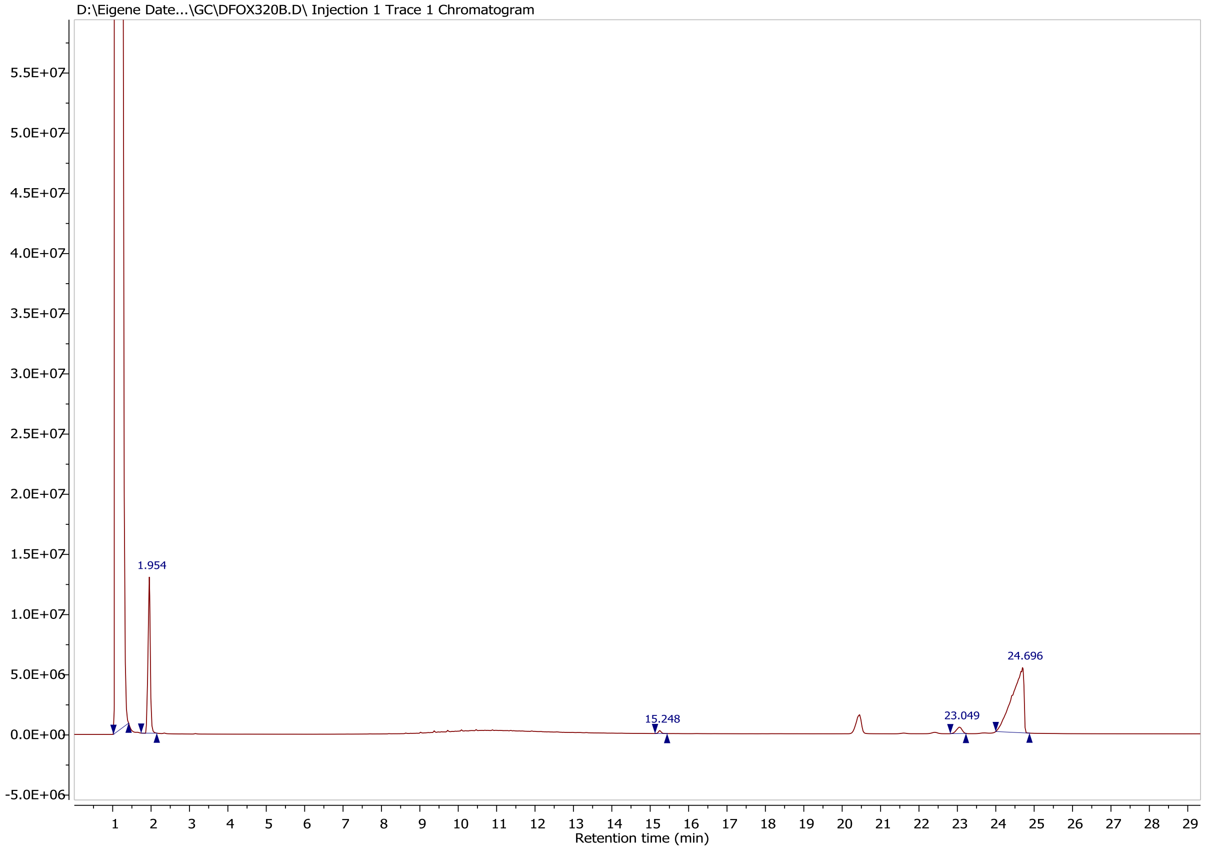

example for dichlorobis(2,4,6-tri-*tert*-butylphenoxy)silane (**14**)
to bis(2,4,6-tri-*tert*-butylphenoxy)silandiol (**15**) in THF/$H_2O$ reflux 30 min

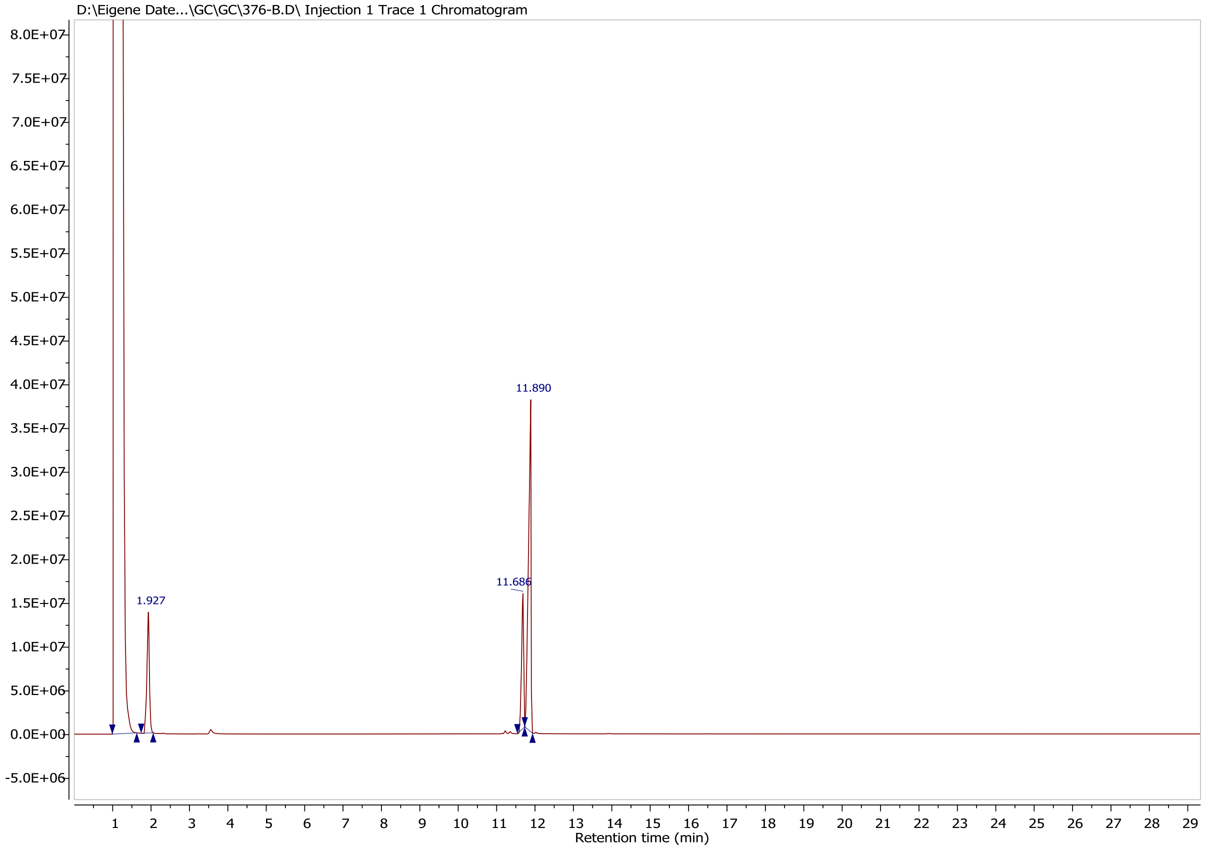


## 6. General procedure for UV–vis titration

Stock solutions of the employed compounds with a concentration of 1 × $10^{-2}$ M in dry HPLC-grade chloroform were prepared. The examined anion $Cl^{-}$ was employed as the *tert*-butylammonium salt and stock solution with a concentration of 1 × $10^{-2}$ M was prepared. The appropriate amount of anion stock solution was added between UV/Vis-measurements. The measurements were recorded on a Perkin Elmer Lambda35 spectrometer featuring a double-beam and all-reflecting system. A deuterium (UV) and a halogen (Vis) lamp, which automatically change at a wavelength of 326 nm, are used to cover the spectral range.

### 1) BIFOXSi$(OH)_2$ **9**

| K | K error (%) | SSR | Data points fitted | Params fitted | H coeffs | HG coeffs | Raw coeffs 1 | Raw coeffs 2 |
|---|---|---|---|---|---|---|---|---|
| 5274,85 | 13,93 | 6,4424E-05 | 22 | 3 | 1457,62 | 1819,75 | 1457,62 | 1819,75 |

| | |
|---|---|
| **RMS: 270.0** | 0,00171124 |
| **RMS: Total** | 0,00171124 |
| **Covariance: 270.0** | 0,03495925 |
| **Covariance: Total** | 0,03495925 |

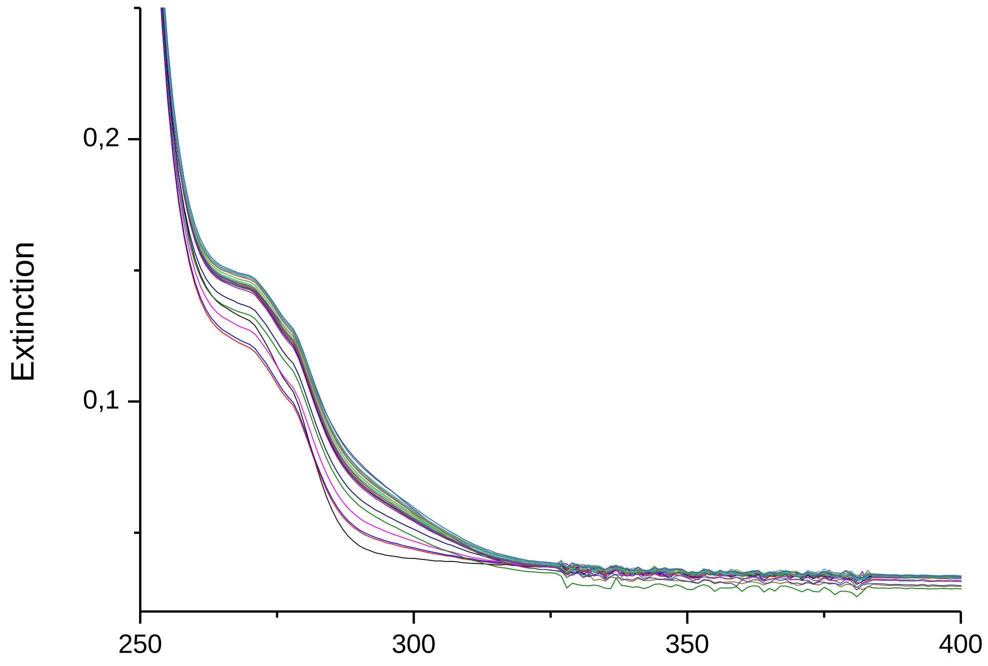


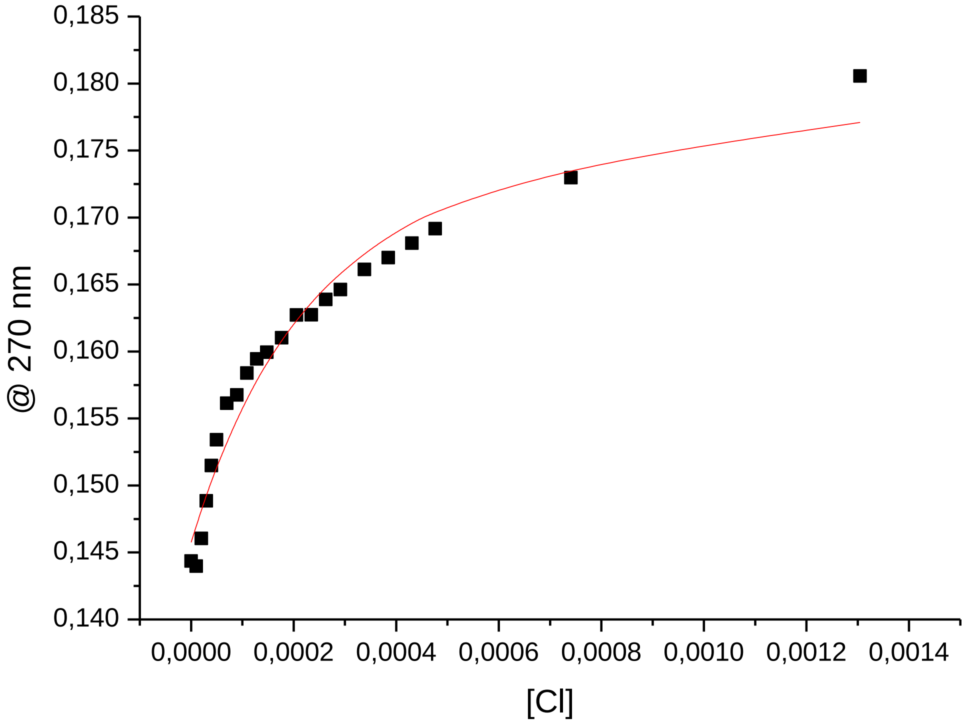


2) BIFOXSiCl(OH) **8**

| K | K error (%) | SSR | Data points fitted | Params fitted | H coeffs | HG coeffs | Raw coeffs 1 | Raw coeffs 2 |
|---|---|---|---|---|---|---|---|---|
| 451,10 | 4,10 | 1,7963E-06 | 22 | 3 | 1368,37 | 1740,62 | 1368,37 | 1740,62 |

| | |
|---|---|
| **RMS: 270.0** | 0,00028575 |
| **RMS: Total** | 0,00028575 |
| **Covariance: 270.0** | 0,00782941 |
| **Covariance: Total** | 0,00782941 |

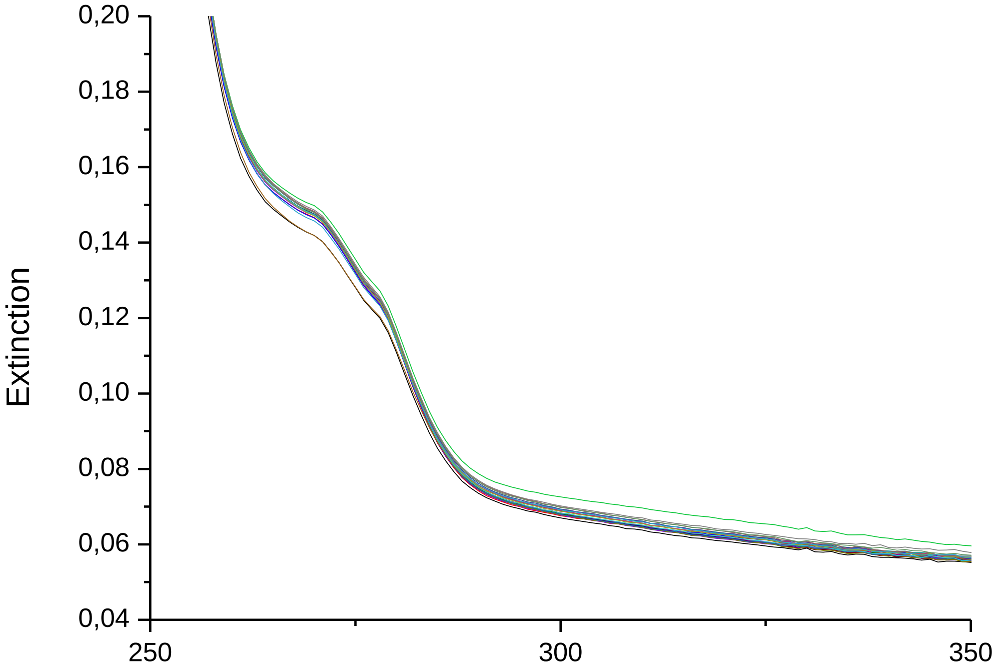

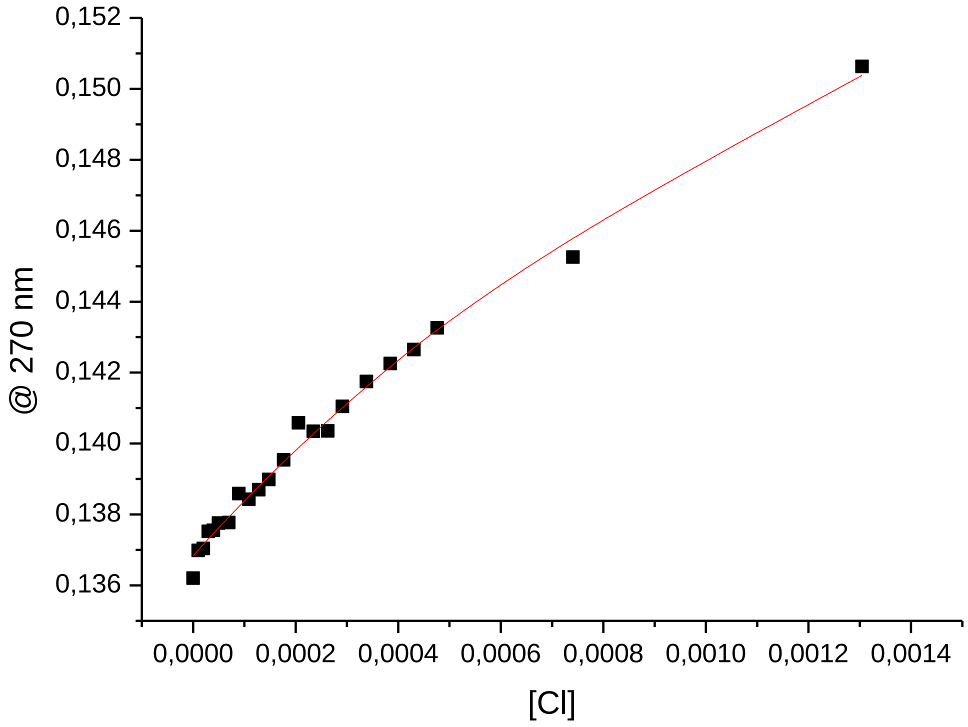


3) Kondo **1**

| K | K error (%) | SSR | Data points fitted | Params fitted | H coeffs | HG coeffs | Raw coeffs 1 | Raw coeffs 2 |
|---|---|---|---|---|---|---|---|---|
| 4688,04 | 4,96 | 0,00255167 | 22 | 3 | 12512,08 | 18875,09 | 12512,08 | 18875,09 |

| | |
|---|---|
| **RMS: 283.0** | 0,01076964 |
| **RMS: Total** | 0,01076964 |
| **Covariance: 283.0** | 0,00487214 |
| **Covariance: Total** | 0,00487214 |

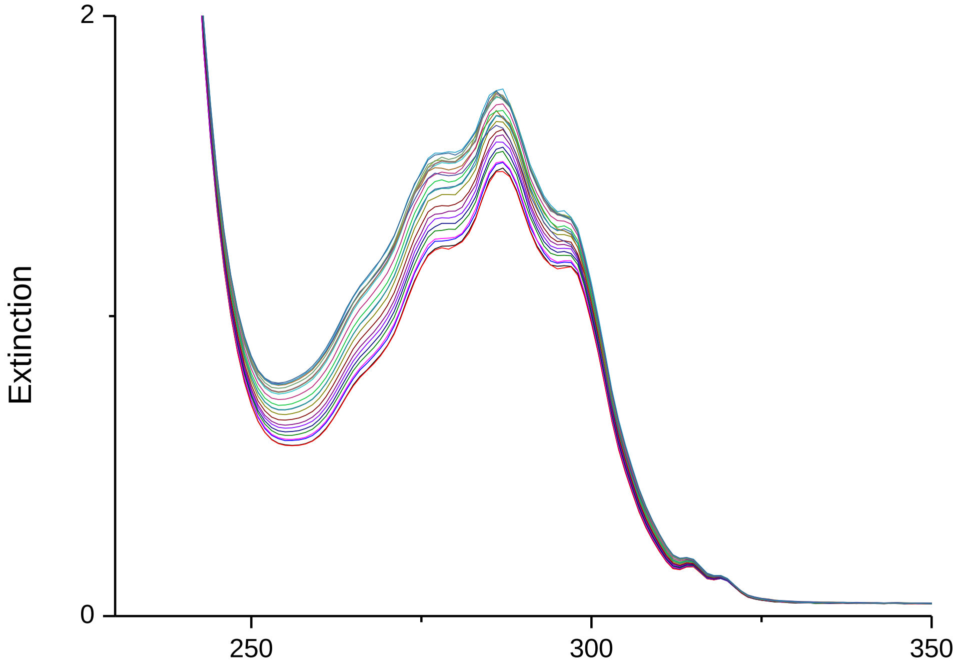
2
0
Extinction
250
300
350

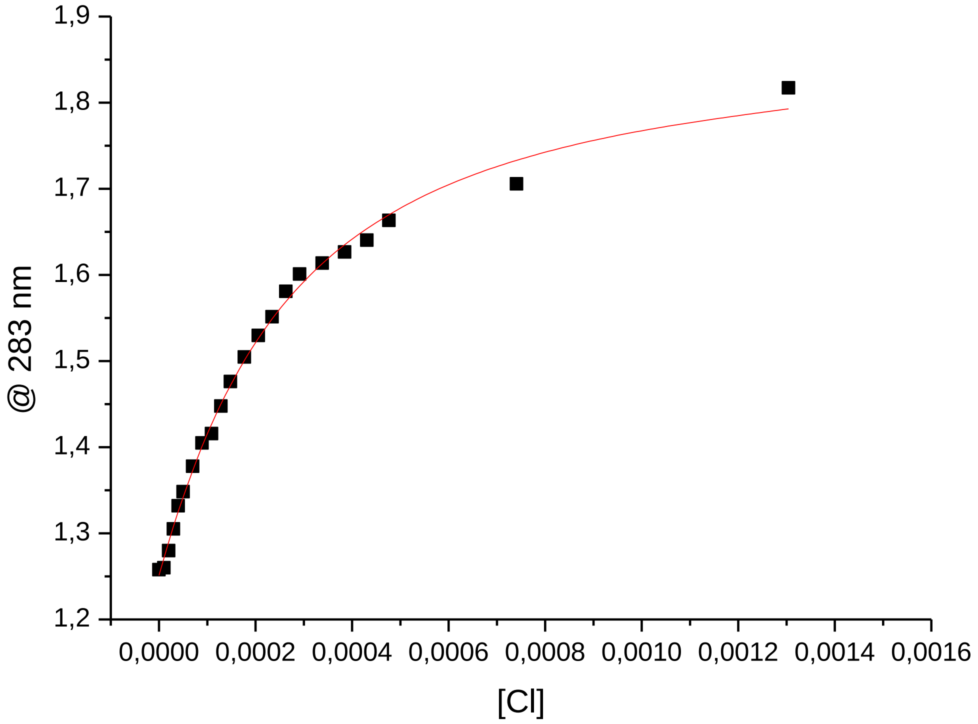
1,9
1,8
1,7
1,6
1,5
1,4
1,3
1,2
@ 283 nm
0,0000
0,0002
0,0004
0,0006
0,0008
0,0010
0,0012
0,0014
0,0016
[Cl]

7. **Computational details**

**Computational details:** In this work computations were performed using GAUSSIAN09[5]. Geometry optimizations were performed at the B3LYP-D3BJ/6-31G(d) level of theory. Zero-point energies were scaled by 0.96[6]. Frequency computations were performed at the M06-2X-D3/6-311++G(d,p) level of theory using the SCRF method with THF as solvent.

a. **BIFOXSi derivatives**

BIFOXSiCl$_2$ **7**

HF=-2603.7857186
Thermal correction to Gibbs Free Energy= 0.574622
NImag=0

| | | | | | |
|---|---|---|---|---|---|
| 1 | 17 | 0 | 0.516141 | -2.107481 | 2.240961 |
| 2 | 14 | 0 | 0.033856 | -1.590143 | 0.305178 |
| 3 | 1 | 0 | 2.940397 | -2.745736 | 1.095431 |
| 4 | 17 | 0 | -0.105566 | -3.384988 | -0.727559 |
| 5 | 8 | 0 | -1.395317 | -0.875870 | 0.508842 |
| 6 | 8 | 0 | 1.078348 | -0.705556 | -0.576413 |
| 7 | 6 | 0 | 3.609250 | -1.885418 | 1.173689 |
| 8 | 1 | 0 | -2.677805 | -2.686673 | -0.230420 |
| 9 | 6 | 0 | -2.253639 | 0.016389 | -0.195509 |
| 10 | 6 | 0 | 2.233556 | 0.055490 | -0.151983 |
| 11 | 6 | 0 | 3.460861 | -0.961795 | -0.038686 |
| 12 | 1 | 0 | 3.422404 | -1.420583 | 2.139770 |
| 13 | 1 | 0 | 4.632826 | -2.278859 | 1.179029 |
| 14 | 6 | 0 | -3.486864 | -2.146637 | -0.713262 |
| 15 | 6 | 0 | -1.469470 | 1.266953 | -0.632100 |
| 16 | 6 | 0 | -3.028591 | -0.781712 | -1.321394 |
| 17 | 6 | 0 | -3.457400 | 0.416064 | 0.819083 |
| 18 | 6 | 0 | 1.766283 | 0.798365 | 1.116305 |
| 19 | 6 | 0 | 2.737556 | 1.022597 | -1.341862 |
| 20 | 6 | 0 | 4.662867 | -0.056575 | -0.381585 |
| 21 | 6 | 0 | 3.439236 | -1.855120 | -1.323442 |
| 22 | 6 | 0 | -4.639509 | -1.788329 | 0.260863 |
| 23 | 1 | 0 | -3.844610 | -2.775033 | -1.535642 |
| 24 | 6 | 0 | -0.408018 | 1.856520 | 0.104746 |
| 25 | 6 | 0 | -1.980289 | 2.007746 | -1.713486 |
| 26 | 6 | 0 | -4.408355 | -0.072274 | -1.351420 |

| | | | | | |
|---|---|---|---|---|---|
| 27 | 6 | 0 | -2.280229 | -1.007032 | -2.633444 |
| 28 | 6 | 0 | -4.694576 | -0.256763 | 0.148809 |
| 29 | 6 | 0 | -3.188645 | -0.079746 | 2.245954 |
| 30 | 6 | 0 | -3.723420 | 1.930799 | 0.907867 |
| 31 | 6 | 0 | 0.450701 | 1.347494 | 1.250183 |
| 32 | 6 | 0 | 2.620381 | 0.888447 | 2.220801 |
| 33 | 6 | 0 | 4.110507 | 0.402236 | -1.740873 |
| 34 | 6 | 0 | 1.746434 | 1.099432 | -2.509232 |
| 35 | 6 | 0 | 3.039676 | 2.453770 | -0.862473 |
| 36 | 1 | 0 | 4.842372 | 0.755224 | 0.326799 |
| 37 | 1 | 0 | 5.586481 | -0.639912 | -0.469855 |
| 38 | 6 | 0 | 3.930859 | -0.936788 | -2.467655 |
| 39 | 1 | 0 | 2.456522 | -2.285779 | -1.506488 |
| 40 | 1 | 0 | 4.132959 | -2.686547 | -1.160828 |
| 41 | 1 | 0 | -5.589840 | -2.219346 | -0.072656 |
| 42 | 1 | 0 | -4.466080 | -2.145311 | 1.277791 |
| 43 | 6 | 0 | -0.097060 | 3.206811 | -0.160169 |
| 44 | 6 | 0 | -1.576292 | 3.300427 | -2.025329 |
| 45 | 1 | 0 | -2.757810 | 1.577068 | -2.321915 |
| 46 | 1 | 0 | -4.406812 | 0.968800 | -1.671764 |
| 47 | 1 | 0 | -5.117892 | -0.616211 | -1.985673 |
| 48 | 1 | 0 | -1.405574 | -1.644669 | -2.474419 |
| 49 | 1 | 0 | -1.931006 | -0.090061 | -3.109257 |
| 50 | 1 | 0 | -2.933313 | -1.528412 | -3.342618 |
| 51 | 1 | 0 | -5.631606 | 0.178958 | 0.509362 |
| 52 | 1 | 0 | -2.337833 | 0.445951 | 2.686322 |
| 53 | 1 | 0 | -2.960491 | -1.143418 | 2.292750 |
| 54 | 1 | 0 | -4.070072 | 0.115119 | 2.868634 |
| 55 | 1 | 0 | -4.552595 | 2.100518 | 1.603944 |
| 56 | 1 | 0 | -3.997772 | 2.376740 | -0.050513 |
| 57 | 1 | 0 | -2.853660 | 2.477359 | 1.282037 |
| 58 | 6 | 0 | 0.044617 | 1.794346 | 2.516107 |
| 59 | 1 | 0 | 3.647387 | 0.567757 | 2.116352 |
| 60 | 6 | 0 | 2.200493 | 1.359529 | 3.462669 |
| 61 | 1 | 0 | 4.725125 | 1.122557 | -2.289124 |
| 62 | 1 | 0 | 0.862645 | 1.680017 | -2.241907 |
| 63 | 1 | 0 | 1.405800 | 0.114417 | -2.830268 |
| 64 | 1 | 0 | 2.229341 | 1.593798 | -3.361076 |
| 65 | 1 | 0 | 3.497460 | 3.010338 | -1.688373 |
| 66 | 1 | 0 | 3.730819 | 2.484861 | -0.016692 |
| 67 | 1 | 0 | 2.137682 | 2.982967 | -0.573823 |
| 68 | 1 | 0 | 4.889395 | -1.279876 | -2.872530 |
| 69 | 1 | 0 | 3.230718 | -0.878120 | -3.303386 |
| 70 | 1 | 0 | 0.621587 | 3.690720 | 0.491870 |

| | | | | | |
|---|---|---|---|---|---|
| 71 | 6 | 0 | -0.655910 | 3.934904 | -1.202022 |
| 72 | 1 | 0 | -2.015349 | 3.811401 | -2.877174 |
| 73 | 6 | 0 | 0.883507 | 1.770335 | 3.626938 |
| 74 | 1 | 0 | -0.948828 | 2.220849 | 2.611159 |
| 75 | 1 | 0 | 2.900062 | 1.387412 | 4.292923 |
| 76 | 1 | 0 | -0.364113 | 4.967716 | -1.366690 |
| 77 | 1 | 0 | 0.521956 | 2.116583 | 4.590628 |

BIFOXSi$Cl_2$ **7** to BIFOXSiCl(OH) **8$_{ax}$** TS$_{front1}$ **7**

HF =-2680.1774268
Thermal correction to Gibbs Free Energy= 0.594561
NImag=1
Frequencies -- -221.4407 Hz

| | | | | | |
|---|---|---|---|---|---|
| 1 | 17 | 0 | 0.416731 | -1.067098 | 2.744203 |
| 2 | 14 | 0 | -0.013824 | -1.557411 | 0.220395 |
| 3 | 1 | 0 | 2.823176 | -2.200271 | 2.023649 |
| 4 | 17 | 0 | -0.384123 | -2.841157 | -1.456155 |
| 5 | 8 | 0 | -1.405572 | -0.818768 | 0.544465 |
| 6 | 8 | 0 | 1.224974 | -0.788050 | -0.446024 |
| 7 | 6 | 0 | 3.547342 | -1.398530 | 1.867665 |
| 8 | 1 | 0 | -2.775585 | -2.672825 | 0.196293 |
| 9 | 6 | 0 | -2.338413 | 0.028661 | -0.145596 |
| 10 | 6 | 0 | 2.337454 | 0.048295 | -0.099013 |
| 11 | 6 | 0 | 3.515598 | -0.905529 | 0.420517 |
| 12 | 1 | 0 | 3.336557 | -0.641538 | 2.619417 |
| 13 | 1 | 0 | 4.543809 | -1.814031 | 2.063030 |
| 14 | 6 | 0 | -3.669867 | -2.136500 | -0.114169 |
| 15 | 6 | 0 | -1.573477 | 1.115939 | -0.922232 |
| 16 | 6 | 0 | -3.356915 | -0.882529 | -0.984075 |
| 17 | 6 | 0 | -3.310997 | 0.663986 | 0.982104 |
| 18 | 6 | 0 | 1.790742 | 1.128681 | 0.847582 |
| 19 | 6 | 0 | 2.976298 | 0.626467 | -1.467279 |
| 20 | 6 | 0 | 4.777599 | -0.176353 | -0.094209 |
| 21 | 6 | 0 | 3.559273 | -2.150969 | -0.523622 |
| 22 | 6 | 0 | -4.512064 | -1.597869 | 1.072737 |
| 23 | 1 | 0 | -4.258161 | -2.824534 | -0.729609 |
| 24 | 6 | 0 | -0.396432 | 1.797005 | -0.491668 |
| 25 | 6 | 0 | -2.199456 | 1.591745 | -2.089351 |
| 26 | 6 | 0 | -4.706647 | -0.145280 | -0.794268 |
| 27 | 6 | 0 | -3.005054 | -1.256042 | -2.438147 |
| 28 | 6 | 0 | -4.644044 | -0.099013 | 0.735869 |

| | | | | | |
|---|---|---|---|---|---|
| 29 | 6 | 0 | -2.772073 | 0.490523 | 2.405981 |
| 30 | 6 | 0 | -3.582804 | 2.167434 | 0.771697 |
| 31 | 6 | 0 | 0.477945 | 1.685325 | 0.756142 |
| 32 | 6 | 0 | 2.620163 | 1.582156 | 1.880239 |
| 33 | 6 | 0 | 4.331215 | -0.132959 | -1.564038 |
| 34 | 6 | 0 | 2.070323 | 0.411733 | -2.686572 |
| 35 | 6 | 0 | 3.315497 | 2.126187 | -1.383619 |
| 36 | 1 | 0 | 4.957034 | 0.805056 | 0.349414 |
| 37 | 1 | 0 | 5.674729 | -0.785966 | 0.063369 |
| 38 | 6 | 0 | 4.122748 | -1.624977 | -1.866275 |
| 39 | 1 | 0 | 2.591506 | -2.639073 | -0.626589 |
| 40 | 1 | 0 | 4.240894 | -2.879224 | -0.071157 |
| 41 | 1 | 0 | -5.502031 | -2.066267 | 1.097953 |
| 42 | 1 | 0 | -4.046311 | -1.778551 | 2.043276 |
| 43 | 6 | 0 | -0.016518 | 2.948040 | -1.214982 |
| 44 | 6 | 0 | -1.754894 | 2.683656 | -2.822583 |
| 45 | 1 | 0 | -3.097593 | 1.105206 | -2.430718 |
| 46 | 1 | 0 | -4.762942 | 0.833259 | -1.273071 |
| 47 | 1 | 0 | -5.536633 | -0.752091 | -1.174934 |
| 48 | 1 | 0 | -2.003942 | -0.941883 | -2.733206 |
| 49 | 1 | 0 | -3.712998 | -0.804680 | -3.142966 |
| 50 | 1 | 0 | -3.052089 | -2.338128 | -2.580191 |
| 51 | 1 | 0 | -5.484449 | 0.390313 | 1.237792 |
| 52 | 1 | 0 | -1.853807 | 1.058897 | 2.557877 |
| 53 | 1 | 0 | -2.532886 | -0.542344 | 2.649557 |
| 54 | 1 | 0 | -3.524323 | 0.853288 | 3.117513 |
| 55 | 1 | 0 | -4.290921 | 2.505176 | 1.536722 |
| 56 | 1 | 0 | -4.009822 | 2.401957 | -0.205205 |
| 57 | 1 | 0 | -2.674758 | 2.763716 | 0.870217 |
| 58 | 6 | 0 | 0.063437 | 2.547420 | 1.783480 |
| 59 | 1 | 0 | 3.642634 | 1.234664 | 1.919006 |
| 60 | 6 | 0 | 2.186466 | 2.447599 | 2.879024 |
| 61 | 1 | 0 | 5.015676 | 0.364586 | -2.257964 |
| 62 | 1 | 0 | 1.190366 | 1.056456 | -2.640075 |
| 63 | 1 | 0 | 1.718029 | -0.616218 | -2.773177 |
| 64 | 1 | 0 | 2.626368 | 0.666790 | -3.597118 |
| 65 | 1 | 0 | 3.863842 | 2.412170 | -2.288607 |
| 66 | 1 | 0 | 3.936574 | 2.381531 | -0.522488 |
| 67 | 1 | 0 | 2.420356 | 2.739310 | -1.336348 |
| 68 | 1 | 0 | 5.079860 | -2.099231 | -2.109766 |
| 69 | 1 | 0 | 3.452191 | -1.801252 | -2.709547 |
| 70 | 1 | 0 | 0.803666 | 3.533495 | -0.819109 |
| 71 | 6 | 0 | -0.651914 | 3.391676 | -2.366394 |
| 72 | 1 | 0 | -2.290395 | 2.987745 | -3.717201 |

| | | | | | |
|---|---|---|---|---|---|
| 73 | 6 | 0 | 0.875781 | 2.903595 | 2.855219 |
| 74 | 1 | 0 | -0.919588 | 2.996602 | 1.707296 |
| 75 | 1 | 0 | 2.869514 | 2.747571 | 3.668359 |
| 76 | 1 | 0 | -0.296381 | 4.281211 | -2.877939 |
| 77 | 1 | 0 | 0.497079 | 3.565659 | 3.628313 |
| 78 | 8 | 0 | 0.254825 | -3.122875 | 1.163240 |
| 79 | 1 | 0 | -0.300479 | -3.882613 | 0.898929 |
| 80 | 1 | 0 | 0.229753 | -2.799757 | 2.152212 |

BIFOXSiCl$_2$ **7** to BIFOXSiCl(OH) **8$_{eq}$** TS$_{front2}$ **7**

HF= -2680.172952
Thermal correction to Gibbs Free Energy= 0.594578
NImag=1
Frequencies -- -206.2297

| | | | | | |
|---|---|---|---|---|---|
| 1 | 8 | 0 | 1.399738 | -0.753786 | 0.329382 |
| 2 | 8 | 0 | -1.170442 | -0.646519 | -0.634608 |
| 3 | 6 | 0 | 2.257166 | 0.254004 | -0.202464 |
| 4 | 6 | 0 | -2.313276 | 0.076027 | -0.152493 |
| 5 | 6 | 0 | -1.855364 | 0.813108 | 1.121051 |
| 6 | 6 | 0 | -2.724688 | 0.891242 | 2.214788 |
| 7 | 6 | 0 | -2.321538 | 1.335065 | 3.471909 |
| 8 | 6 | 0 | -1.003409 | 1.729228 | 3.662751 |
| 9 | 6 | 0 | -0.155500 | 1.782712 | 2.559836 |
| 10 | 6 | 0 | -0.548352 | 1.372737 | 1.276995 |
| 11 | 1 | 0 | -3.752376 | 0.580202 | 2.090505 |
| 12 | 1 | 0 | -3.033821 | 1.353222 | 4.291557 |
| 13 | 1 | 0 | -0.650093 | 2.050050 | 4.638158 |
| 14 | 1 | 0 | 2.115507 | 0.367679 | 2.721030 |
| 15 | 6 | 0 | -3.539755 | -0.960427 | -0.023685 |
| 16 | 6 | 0 | -2.846743 | 1.046412 | -1.333430 |
| 17 | 6 | 0 | -3.515829 | -1.877521 | -1.285146 |
| 18 | 6 | 0 | -4.750892 | -0.078324 | -0.403968 |
| 19 | 6 | 0 | -4.184234 | 0.374883 | -1.758126 |
| 20 | 6 | 0 | -3.948086 | -0.970607 | -2.462502 |
| 21 | 1 | 0 | -2.550459 | -2.366984 | -1.423814 |
| 22 | 1 | 0 | -4.241520 | -2.681039 | -1.123818 |
| 23 | 1 | 0 | -4.957053 | 0.737759 | 0.291106 |
| 24 | 1 | 0 | -5.662341 | -0.679620 | -0.498379 |
| 25 | 1 | 0 | -4.809811 | 1.062678 | -2.334341 |
| 26 | 1 | 0 | -3.206034 | -0.906428 | -3.261797 |
| 27 | 1 | 0 | -4.876216 | -1.331309 | -2.918277 |

| | | | | | |
|---|---|---|---|---|---|
| 28 | 6 | 0 | 3.156419 | -0.353508 | -1.364016 |
| 29 | 6 | 0 | 3.363801 | 0.567819 | 0.945346 |
| 30 | 6 | 0 | 3.613404 | -1.775397 | -0.919013 |
| 31 | 6 | 0 | 4.505597 | 0.390042 | -1.169762 |
| 32 | 6 | 0 | 4.669258 | 0.004926 | 0.308916 |
| 33 | 6 | 0 | 4.641861 | -1.530492 | 0.215174 |
| 34 | 1 | 0 | 2.792240 | -2.414982 | -0.608707 |
| 35 | 1 | 0 | 4.088757 | -2.255125 | -1.781801 |
| 36 | 1 | 0 | 4.492199 | 1.466055 | -1.343556 |
| 37 | 1 | 0 | 5.282684 | -0.042862 | -1.810140 |
| 38 | 1 | 0 | 5.559311 | 0.401830 | 0.807333 |
| 39 | 1 | 0 | 4.362247 | -2.018956 | 1.149836 |
| 40 | 1 | 0 | 5.634106 | -1.904801 | -0.059156 |
| 41 | 6 | 0 | -3.716765 | -1.861809 | 1.204858 |
| 42 | 1 | 0 | -3.490486 | -1.393504 | 2.159673 |
| 43 | 1 | 0 | -4.759945 | -2.200160 | 1.230781 |
| 44 | 1 | 0 | -3.090498 | -2.753323 | 1.133176 |
| 45 | 6 | 0 | -3.206653 | 2.459891 | -0.837302 |
| 46 | 1 | 0 | -3.783670 | 2.971405 | -1.616287 |
| 47 | 1 | 0 | -3.804897 | 2.459615 | 0.075940 |
| 48 | 1 | 0 | -2.316804 | 3.053197 | -0.650810 |
| 49 | 6 | 0 | -1.847020 | 1.196928 | -2.486516 |
| 50 | 1 | 0 | -0.900019 | 3.693391 | 0.589488 |
| 51 | 1 | 0 | -1.515889 | 0.238344 | -2.888353 |
| 52 | 1 | 0 | -2.316030 | 1.762939 | -3.300496 |
| 53 | 6 | 0 | 3.569118 | 2.071581 | 1.211858 |
| 54 | 1 | 0 | 4.334628 | 2.187952 | 1.987166 |
| 55 | 1 | 0 | 3.902303 | 2.625376 | 0.331337 |
| 56 | 1 | 0 | 2.655870 | 2.554444 | 1.568303 |
| 57 | 6 | 0 | 3.008279 | -0.087846 | 2.286152 |
| 58 | 1 | 0 | 0.831790 | 2.216539 | 2.676035 |
| 59 | 1 | 0 | 2.809085 | -1.154769 | 2.201039 |
| 60 | 1 | 0 | 3.839826 | 0.056354 | 2.986486 |
| 61 | 6 | 0 | 1.437690 | 1.511099 | -0.545150 |
| 62 | 6 | 0 | 1.977185 | 2.378857 | -1.514612 |
| 63 | 6 | 0 | 0.297341 | 1.973457 | 0.164499 |
| 64 | 6 | 0 | 1.496312 | 3.656741 | -1.768561 |
| 65 | 1 | 0 | 2.840182 | 2.062172 | -2.075380 |
| 66 | 6 | 0 | -0.109130 | 3.309806 | -0.044730 |
| 67 | 6 | 0 | 0.454324 | 4.149590 | -0.994518 |
| 68 | 1 | 0 | 1.965392 | 4.266105 | -2.535520 |
| 69 | 1 | 0 | -0.955772 | 1.739212 | -2.167803 |
| 70 | 1 | 0 | 0.086646 | 5.164090 | -1.116074 |
| 71 | 6 | 0 | 2.568241 | -0.415989 | -2.774491 |

| | | | | | |
|---|---|---|---|---|---|
| 72 | 1 | 0 | 3.358319 | -0.702889 | -3.478353 |
| 73 | 1 | 0 | 1.789369 | -1.178654 | -2.829898 |
| 74 | 1 | 0 | 2.132639 | 0.519818 | -3.125989 |
| 75 | 8 | 0 | 0.183465 | -2.287374 | -1.632235 |
| 76 | 1 | 0 | 0.460237 | -3.262652 | -1.434132 |
| 77 | 1 | 0 | -0.656751 | -2.190215 | -2.119243 |
| 78 | 17 | 0 | 0.954802 | -4.081949 | 0.144539 |
| 79 | 14 | 0 | 0.025023 | -1.532485 | 0.085809 |
| 80 | 17 | 0 | -0.615262 | -2.024011 | 1.987794 |

BIFOXSiCl$_2$ **7** to BIFOXSiCl(OH) $\mathbf{8_{eq}}$ TS$_{side}$ **7**

HF=-2680.1727746
Thermal correction to Gibbs Free Energy= 0.596455
NImag=1
Frequencies -- -189.6553

| | | | | | |
|---|---|---|---|---|---|
| 1 | 17 | 0 | 0.642859 | -2.133150 | 1.937293 |
| 2 | 14 | 0 | -0.038703 | -1.568625 | 0.075775 |
| 3 | 1 | 0 | 3.008919 | -2.878636 | 0.908728 |
| 4 | 8 | 0 | -1.409755 | -0.773095 | 0.354003 |
| 5 | 8 | 0 | 1.093959 | -0.609169 | -0.640155 |
| 6 | 6 | 0 | 3.670902 | -2.017038 | 1.012233 |
| 7 | 1 | 0 | -2.863777 | -2.361690 | -0.646065 |
| 8 | 6 | 0 | -2.204340 | 0.280383 | -0.180579 |
| 9 | 6 | 0 | 2.279519 | 0.048408 | -0.161017 |
| 10 | 6 | 0 | 3.478628 | -1.024280 | -0.140766 |
| 11 | 1 | 0 | 3.511185 | -1.610266 | 2.008535 |
| 12 | 1 | 0 | 4.699636 | -2.394442 | 0.961836 |
| 13 | 6 | 0 | -3.631364 | -1.672288 | -0.985398 |
| 14 | 6 | 0 | -1.341422 | 1.515952 | -0.492555 |
| 15 | 6 | 0 | -3.079270 | -0.270738 | -1.382307 |
| 16 | 6 | 0 | -3.336844 | 0.626002 | 0.928246 |
| 17 | 6 | 0 | 1.875439 | 0.722591 | 1.165251 |
| 18 | 6 | 0 | 2.812648 | 1.078395 | -1.291551 |
| 19 | 6 | 0 | 4.705850 | -0.149423 | -0.477515 |
| 20 | 6 | 0 | 3.384214 | -1.842354 | -1.468426 |
| 21 | 6 | 0 | -4.690649 | -1.391571 | 0.109661 |
| 22 | 1 | 0 | -4.097732 | -2.107857 | -1.876450 |
| 23 | 6 | 0 | -0.247370 | 1.969681 | 0.290334 |
| 24 | 6 | 0 | -1.799288 | 2.380709 | -1.504591 |
| 25 | 6 | 0 | -4.396509 | 0.538166 | -1.238457 |
| 26 | 6 | 0 | -2.385484 | -0.311607 | -2.759602 |

| | | | | | |
|---|---|---|---|---|---|
| 27 | 6 | 0 | -4.641921 | 0.141687 | 0.226359 |
| 28 | 6 | 0 | -3.061431 | -0.075303 | 2.264359 |
| 29 | 6 | 0 | -3.481477 | 2.131735 | 1.221199 |
| 30 | 6 | 0 | 0.588545 | 1.312310 | 1.373906 |
| 31 | 6 | 0 | 2.761262 | 0.710040 | 2.246947 |
| 32 | 6 | 0 | 4.130642 | 0.414160 | -1.784120 |
| 33 | 6 | 0 | 1.803695 | 1.314694 | -2.420630 |
| 34 | 6 | 0 | 3.212033 | 2.448315 | -0.712235 |
| 35 | 1 | 0 | 4.948602 | 0.606135 | 0.272890 |
| 36 | 1 | 0 | 5.597221 | -0.767694 | -0.633477 |
| 37 | 6 | 0 | 3.858359 | -0.872257 | -2.578257 |
| 38 | 1 | 0 | 2.385055 | -2.240103 | -1.638291 |
| 39 | 1 | 0 | 4.062895 | -2.696898 | -1.379796 |
| 40 | 1 | 0 | -5.687820 | -1.711027 | -0.211533 |
| 41 | 1 | 0 | -4.473085 | -1.907470 | 1.045760 |
| 42 | 6 | 0 | 0.146251 | 3.317158 | 0.145802 |
| 43 | 6 | 0 | -1.319044 | 3.670422 | -1.698276 |
| 44 | 1 | 0 | -2.590894 | 2.054124 | -2.158582 |
| 45 | 1 | 0 | -4.313778 | 1.613651 | -1.393561 |
| 46 | 1 | 0 | -5.167925 | 0.156187 | -1.917110 |
| 47 | 1 | 0 | -1.363774 | 0.086853 | -2.729953 |
| 48 | 1 | 0 | -2.915518 | 0.286141 | -3.508325 |
| 49 | 1 | 0 | -2.365239 | -1.333912 | -3.159434 |
| 50 | 1 | 0 | -5.531258 | 0.577597 | 0.691627 |
| 51 | 1 | 0 | -2.161460 | 0.327989 | 2.735372 |
| 52 | 1 | 0 | -2.910037 | -1.148412 | 2.159875 |
| 53 | 1 | 0 | -3.906695 | 0.095281 | 2.941874 |
| 54 | 1 | 0 | -4.286889 | 2.270026 | 1.951111 |
| 55 | 1 | 0 | -3.728519 | 2.722878 | 0.336471 |
| 56 | 1 | 0 | -2.568545 | 2.555162 | 1.646745 |
| 57 | 6 | 0 | 0.226357 | 1.671469 | 2.679590 |
| 58 | 1 | 0 | 3.774303 | 0.366935 | 2.090519 |
| 59 | 6 | 0 | 2.389279 | 1.100838 | 3.531722 |
| 60 | 1 | 0 | 4.761264 | 1.132862 | -2.316265 |
| 61 | 1 | 0 | 0.947741 | 1.894539 | -2.074939 |
| 62 | 1 | 0 | 1.425446 | 0.381270 | -2.840390 |
| 63 | 1 | 0 | 2.288836 | 1.878219 | -3.227017 |
| 64 | 1 | 0 | 3.709274 | 3.030859 | -1.496567 |
| 65 | 1 | 0 | 3.899843 | 2.371018 | 0.132279 |
| 66 | 1 | 0 | 2.347120 | 3.015570 | -0.384016 |
| 67 | 1 | 0 | 4.781878 | -1.227187 | -3.048833 |
| 68 | 1 | 0 | 3.125454 | -0.737519 | -3.377191 |
| 69 | 1 | 0 | 0.895910 | 3.692640 | 0.833347 |
| 70 | 6 | 0 | -0.365392 | 4.170709 | -0.822181 |

| | | | | | |
|---|---|---|---|---|---|
| 71 | 1 | 0 | -1.721862 | 4.280686 | -2.501300 |
| 72 | 6 | 0 | 1.089681 | 1.532879 | 3.763788 |
| 73 | 1 | 0 | -0.746708 | 2.126380 | 2.833747 |
| 74 | 1 | 0 | 3.111351 | 1.048041 | 4.341300 |
| 75 | 1 | 0 | -0.010148 | 5.194260 | -0.893360 |
| 76 | 1 | 0 | 0.762716 | 1.813527 | 4.760573 |
| 77 | 17 | 0 | -1.184505 | -4.018216 | 0.159299 |
| 78 | 8 | 0 | -0.201100 | -2.348866 | -1.631310 |
| 79 | 1 | 0 | -0.534304 | -1.728864 | -2.309885 |
| 80 | 1 | 0 | -0.802679 | -3.162915 | -1.459060 |

BIFOXSiCl(OH) $\mathbf{8}_{\mathbf{ax}}$

HF=-2219.4193095
Thermal correction to Gibbs Free Energy= 0.587261
NImag=0

| | | | | | |
|---|---|---|---|---|---|
| 1 | 1 | 0 | -5.530435 | -2.302116 | 0.280819 |
| 2 | 6 | 0 | -4.597425 | -1.796562 | 0.552854 |
| 3 | 1 | 0 | -4.444923 | -1.958600 | 1.621558 |
| 4 | 6 | 0 | -3.410765 | -2.298644 | -0.310725 |
| 5 | 6 | 0 | -4.675145 | -0.310942 | 0.164994 |
| 6 | 1 | 0 | -3.734540 | -3.066107 | -1.021709 |
| 7 | 1 | 0 | -2.611412 | -2.731584 | 0.282681 |
| 8 | 6 | 0 | -2.949452 | -1.052097 | -1.132298 |
| 9 | 6 | 0 | -4.337812 | -0.389169 | -1.333479 |
| 10 | 1 | 0 | -5.631625 | 0.160364 | 0.412384 |
| 11 | 6 | 0 | -3.474009 | 0.497774 | 0.744298 |
| 12 | 17 | 0 | -0.049955 | -3.500260 | 0.083796 |
| 13 | 6 | 0 | -2.224826 | -0.057450 | -0.136064 |
| 14 | 6 | 0 | -2.148592 | -1.482404 | -2.359049 |
| 15 | 1 | 0 | -4.342960 | 0.579147 | -1.832805 |
| 16 | 1 | 0 | -5.014721 | -1.052452 | -1.884349 |
| 17 | 6 | 0 | -3.762228 | 1.998074 | 0.549521 |
| 18 | 6 | 0 | -3.259849 | 0.273244 | 2.247046 |
| 19 | 14 | 0 | 0.061456 | -1.519559 | 0.675865 |
| 20 | 1 | 0 | -1.257523 | -2.045199 | -2.064381 |
| 21 | 8 | 0 | -1.385899 | -0.790856 | 0.745063 |
| 22 | 6 | 0 | -1.441626 | 1.110389 | -0.764845 |
| 23 | 1 | 0 | -2.758828 | -2.147587 | -2.980656 |
| 24 | 1 | 0 | -1.813854 | -0.652325 | -2.982899 |
| 25 | 1 | 0 | -4.609630 | 2.278032 | 1.185705 |
| 26 | 1 | 0 | -4.020165 | 2.256999 | -0.479551 |

| | | | | | |
|---|---|---|---|---|---|
| 27 | 1 | 0 | -2.907891 | 2.618400 | 0.834861 |
| 28 | 1 | 0 | -2.465995 | 0.922515 | 2.626844 |
| 29 | 1 | 0 | -2.978491 | -0.750734 | 2.487776 |
| 30 | 1 | 0 | -4.182878 | 0.519048 | 2.785687 |
| 31 | 8 | 0 | 1.032129 | -0.789861 | -0.430353 |
| 32 | 8 | 0 | 0.578593 | -1.617209 | 2.230884 |
| 33 | 6 | 0 | -0.393718 | 1.817001 | -0.119691 |
| 34 | 6 | 0 | -1.931212 | 1.661738 | -1.962194 |
| 35 | 6 | 0 | 2.209204 | -0.026566 | -0.088327 |
| 36 | 1 | 0 | 0.507567 | -0.778743 | 2.712945 |
| 37 | 6 | 0 | 0.446380 | 1.488084 | 1.098606 |
| 38 | 6 | 0 | -0.071112 | 3.104553 | -0.594317 |
| 39 | 1 | 0 | -2.695026 | 1.134141 | -2.509007 |
| 40 | 6 | 0 | -1.520243 | 2.887767 | -2.472915 |
| 41 | 6 | 0 | 3.400513 | -1.051503 | 0.206059 |
| 42 | 6 | 0 | 2.775950 | 0.752253 | -1.381405 |
| 43 | 6 | 0 | 1.753332 | 0.903179 | 1.059410 |
| 44 | 6 | 0 | 0.030734 | 2.114899 | 2.283714 |
| 45 | 6 | 0 | -0.612777 | 3.650978 | -1.750506 |
| 46 | 1 | 0 | 0.643123 | 3.685882 | -0.021386 |
| 47 | 1 | 0 | -1.943426 | 3.251436 | -3.404728 |
| 48 | 6 | 0 | 3.382791 | -2.108619 | -0.948277 |
| 49 | 6 | 0 | 3.482356 | -1.803877 | 1.537310 |
| 50 | 6 | 0 | 4.639307 | -0.231404 | -0.215063 |
| 51 | 6 | 0 | 4.143507 | 0.050991 | -1.642472 |
| 52 | 6 | 0 | 3.100197 | 2.231257 | -1.101895 |
| 53 | 6 | 0 | 1.820550 | 0.684857 | -2.578044 |
| 54 | 6 | 0 | 2.593179 | 1.128017 | 2.157284 |
| 55 | 6 | 0 | 0.853461 | 2.226474 | 3.402974 |
| 56 | 1 | 0 | -0.956179 | 2.565637 | 2.301307 |
| 57 | 1 | 0 | -0.316168 | 4.642301 | -2.079481 |
| 58 | 1 | 0 | 4.027008 | -2.942581 | -0.651139 |
| 59 | 1 | 0 | 2.385891 | -2.512894 | -1.112329 |
| 60 | 6 | 0 | 3.954444 | -1.372023 | -2.183559 |
| 61 | 1 | 0 | 4.469886 | -2.277425 | 1.595661 |
| 62 | 1 | 0 | 2.736094 | -2.598505 | 1.590415 |
| 63 | 1 | 0 | 3.352551 | -1.191482 | 2.428242 |
| 64 | 1 | 0 | 4.819867 | 0.666272 | 0.380741 |
| 65 | 1 | 0 | 5.549423 | -0.841687 | -0.190990 |
| 66 | 1 | 0 | 4.795997 | 0.674797 | -2.261043 |
| 67 | 1 | 0 | 3.598687 | 2.653269 | -1.982238 |
| 68 | 1 | 0 | 3.765927 | 2.369684 | -0.246249 |
| 69 | 1 | 0 | 2.203208 | 2.816816 | -0.924865 |
| 70 | 1 | 0 | 0.947097 | 1.319248 | -2.421584 |

| | | | | | |
|---|---|---|---|---|---|
| 71 | 1 | 0 | 1.458626 | -0.326446 | -2.764237 |
| 72 | 1 | 0 | 2.339804 | 1.040765 | -3.476401 |
| 73 | 1 | 0 | 3.612838 | 0.773341 | 2.116139 |
| 74 | 6 | 0 | 2.164240 | 1.769332 | 3.318317 |
| 75 | 1 | 0 | 0.486173 | 2.707833 | 4.304469 |
| 76 | 1 | 0 | 4.921326 | -1.790072 | -2.485618 |
| 77 | 1 | 0 | 3.298234 | -1.419505 | -3.055473 |
| 78 | 1 | 0 | 2.851470 | 1.894182 | 4.149913 |

BIFOXSiCl(OH) $\mathbf{8_{eq}}$

HF=- 2219.4141309
Thermal correction to Gibbs Free Energy= 0.586319
NImag=0

| | | | | | |
|---|---|---|---|---|---|
| 1 | 1 | 0 | -5.550817 | -2.304345 | -0.470583 |
| 2 | 6 | 0 | -4.609119 | -1.917196 | -0.066347 |
| 3 | 1 | 0 | -4.433396 | -2.425724 | 0.883251 |
| 4 | 6 | 0 | -3.447550 | -2.101240 | -1.077137 |
| 5 | 6 | 0 | -4.684399 | -0.387107 | 0.062769 |
| 6 | 1 | 0 | -3.786281 | -2.604005 | -1.989238 |
| 7 | 1 | 0 | -2.630009 | -2.689452 | -0.666643 |
| 8 | 6 | 0 | -3.002554 | -0.653532 | -1.459095 |
| 9 | 6 | 0 | -4.390821 | 0.035423 | -1.386528 |
| 10 | 1 | 0 | -5.630529 | -0.028299 | 0.479854 |
| 11 | 6 | 0 | -3.461201 | 0.187271 | 0.840734 |
| 12 | 6 | 0 | -2.243555 | -0.037123 | -0.210236 |
| 13 | 6 | 0 | -2.248355 | -0.661971 | -2.788028 |
| 14 | 1 | 0 | -4.396229 | 1.114220 | -1.535773 |
| 15 | 1 | 0 | -5.089795 | -0.406263 | -2.106540 |
| 16 | 6 | 0 | -3.746878 | 1.666241 | 1.162166 |
| 17 | 6 | 0 | -3.197803 | -0.521763 | 2.175589 |
| 18 | 14 | 0 | 0.010598 | -1.711757 | -0.106720 |
| 19 | 1 | 0 | -1.401759 | -1.353375 | -2.743742 |
| 20 | 8 | 0 | -1.376517 | -1.007401 | 0.359558 |
| 21 | 6 | 0 | -1.471218 | 1.272156 | -0.440515 |
| 22 | 1 | 0 | -2.916022 | -1.011306 | -3.584609 |
| 23 | 1 | 0 | -1.859345 | 0.314092 | -3.081063 |
| 24 | 1 | 0 | -4.582047 | 1.717219 | 1.869953 |
| 25 | 1 | 0 | -4.019773 | 2.253722 | 0.282783 |
| 26 | 1 | 0 | -2.885279 | 2.156238 | 1.623352 |
| 27 | 1 | 0 | -2.362982 | -0.054890 | 2.703700 |

| | | | | | |
|---|---|---|---|---|---|
| 28 | 1 | 0 | -2.942670 | -1.573891 | 2.057819 |
| 29 | 1 | 0 | -4.089839 | -0.445829 | 2.809238 |
| 30 | 8 | 0 | 1.082873 | -0.666295 | -0.739248 |
| 31 | 6 | 0 | -0.411927 | 1.727780 | 0.387698 |
| 32 | 6 | 0 | -1.975757 | 2.180839 | -1.387759 |
| 33 | 6 | 0 | 2.224322 | -0.012368 | -0.159068 |
| 34 | 6 | 0 | 0.434834 | 1.027320 | 1.435416 |
| 35 | 6 | 0 | -0.095359 | 3.101574 | 0.351350 |
| 36 | 1 | 0 | -2.749836 | 1.858663 | -2.065158 |
| 37 | 6 | 0 | -1.566218 | 3.506052 | -1.479728 |
| 38 | 6 | 0 | 3.453528 | -1.039186 | -0.205518 |
| 39 | 6 | 0 | 2.751812 | 1.148622 | -1.149746 |
| 40 | 6 | 0 | 1.744669 | 0.493300 | 1.217463 |
| 41 | 6 | 0 | 0.025865 | 1.265959 | 2.755629 |
| 42 | 6 | 0 | -0.647717 | 3.992918 | -0.558981 |
| 43 | 1 | 0 | 0.621320 | 3.468008 | 1.077848 |
| 44 | 1 | 0 | -1.999162 | 4.152370 | -2.237695 |
| 45 | 6 | 0 | 3.442025 | -1.702059 | -1.622096 |
| 46 | 6 | 0 | 3.601107 | -2.153873 | 0.835180 |
| 47 | 6 | 0 | 4.660803 | -0.091960 | -0.378886 |
| 48 | 6 | 0 | 4.121696 | 0.594616 | -1.642977 |
| 49 | 6 | 0 | 3.061527 | 2.469067 | -0.420292 |
| 50 | 6 | 0 | 1.775621 | 1.444543 | -2.294322 |
| 51 | 6 | 0 | 2.591181 | 0.389489 | 2.327263 |
| 52 | 6 | 0 | 0.856704 | 1.050616 | 3.851716 |
| 53 | 1 | 0 | -0.962027 | 1.686450 | 2.913699 |
| 54 | 1 | 0 | -0.352504 | 5.037839 | -0.550742 |
| 55 | 1 | 0 | 4.135628 | -2.549460 | -1.598234 |
| 56 | 1 | 0 | 2.459178 | -2.093286 | -1.881946 |
| 57 | 6 | 0 | 3.943571 | -0.601728 | -2.588069 |
| 58 | 1 | 0 | 4.637524 | -2.511538 | 0.804908 |
| 59 | 1 | 0 | 2.962319 | -3.004651 | 0.589997 |
| 60 | 1 | 0 | 3.372897 | -1.870293 | 1.860736 |
| 61 | 1 | 0 | 4.836875 | 0.585540 | 0.459666 |
| 62 | 1 | 0 | 5.583957 | -0.655483 | -0.557412 |
| 63 | 1 | 0 | 4.744997 | 1.393103 | -2.057193 |
| 64 | 1 | 0 | 3.553633 | 3.151106 | -1.123386 |
| 65 | 1 | 0 | 3.725776 | 2.339625 | 0.437553 |
| 66 | 1 | 0 | 2.159200 | 2.959557 | -0.069492 |
| 67 | 1 | 0 | 0.885585 | 1.961861 | -1.934537 |
| 68 | 1 | 0 | 1.443041 | 0.537826 | -2.800315 |
| 69 | 1 | 0 | 2.267682 | 2.092372 | -3.030429 |
| 70 | 1 | 0 | 3.616784 | 0.084552 | 2.175679 |
| 71 | 6 | 0 | 2.169422 | 0.654424 | 3.628181 |

| | | | | | |
|---|---|---|---|---|---|
| 72 | 1 | 0 | 0.493120 | 1.240816 | 4.857298 |
| 73 | 1 | 0 | 4.905972 | -0.872990 | -3.036432 |
| 74 | 1 | 0 | 3.252018 | -0.402433 | -3.409481 |
| 75 | 1 | 0 | 2.864692 | 0.537936 | 4.454431 |
| 76 | 17 | 0 | 0.481582 | -2.713779 | 1.647025 |
| 77 | 8 | 0 | -0.133606 | -2.837204 | -1.305744 |
| 78 | 1 | 0 | -0.748706 | -3.562168 | -1.127955 |

BIFOXSiCl(OH) **$8_{ax}$** to BIFOXSi(OH)$_2$ **9** TS$_{front1}$ **8ax**

HF=-2295.8124297
Thermal correction to Gibbs Free Energy= 0.608924
NImag=1
Frequencies -- -208.0116

| | | | | | |
|---|---|---|---|---|---|
| 1 | 1 | 0 | 5.483668 | -2.027748 | -1.089875 |
| 2 | 6 | 0 | 4.510804 | -1.524373 | -1.111990 |
| 3 | 1 | 0 | 4.160240 | -1.552927 | -2.145510 |
| 4 | 6 | 0 | 3.516820 | -2.179467 | -0.115744 |
| 5 | 6 | 0 | 4.648923 | -0.092729 | -0.556348 |
| 6 | 1 | 0 | 4.006936 | -2.973136 | 0.456811 |
| 7 | 1 | 0 | 2.649379 | -2.622697 | -0.596100 |
| 8 | 6 | 0 | 3.149572 | -1.044133 | 0.885803 |
| 9 | 6 | 0 | 4.536183 | -0.353460 | 0.951937 |
| 10 | 1 | 0 | 5.556457 | 0.421872 | -0.888433 |
| 11 | 6 | 0 | 3.375335 | 0.757945 | -0.836655 |
| 12 | 6 | 0 | 2.266526 | 0.019319 | 0.090699 |
| 13 | 6 | 0 | 2.576030 | -1.592461 | 2.204474 |
| 14 | 1 | 0 | 4.578331 | 0.548914 | 1.563602 |
| 15 | 1 | 0 | 5.299969 | -1.045500 | 1.325236 |
| 16 | 6 | 0 | 3.659522 | 2.214470 | -0.422363 |
| 17 | 6 | 0 | 2.978229 | 0.782851 | -2.318632 |
| 18 | 14 | 0 | -0.052693 | -1.336168 | -0.919091 |
| 19 | 1 | 0 | 1.517042 | -1.359691 | 2.324693 |
| 20 | 8 | 0 | 1.399606 | -0.665288 | -0.799384 |
| 21 | 6 | 0 | 1.487735 | 1.034910 | 0.944784 |
| 22 | 1 | 0 | 2.656187 | -2.680808 | 2.234315 |
| 23 | 1 | 0 | 3.110172 | -1.198188 | 3.076314 |
| 24 | 1 | 0 | 4.454028 | 2.614605 | -1.062660 |
| 25 | 1 | 0 | 3.983884 | 2.316826 | 0.614737 |
| 26 | 1 | 0 | 2.778617 | 2.849824 | -0.545234 |

| | | | | | |
|---|---|---|---|---|---|
| 27 | 1 | 0 | 2.100371 | 1.413121 | -2.480124 |
| 28 | 1 | 0 | 2.737175 | -0.203572 | -2.710550 |
| 29 | 1 | 0 | 3.806377 | 1.198030 | -2.905941 |
| 30 | 8 | 0 | -1.107810 | -0.802248 | 0.215927 |
| 31 | 6 | 0 | 0.383746 | 1.817560 | 0.512605 |
| 32 | 6 | 0 | 2.040956 | 1.359023 | 2.196501 |
| 33 | 6 | 0 | -2.261430 | 0.032197 | 0.026384 |
| 34 | 6 | 0 | -0.474620 | 1.785559 | -0.741481 |
| 35 | 6 | 0 | 0.033539 | 2.947521 | 1.281707 |
| 36 | 1 | 0 | 2.863580 | 0.772613 | 2.568416 |
| 37 | 6 | 0 | 1.615469 | 2.422265 | 2.982658 |
| 38 | 6 | 0 | -3.468544 | -0.871724 | -0.522453 |
| 39 | 6 | 0 | -2.830028 | 0.472708 | 1.472955 |
| 40 | 6 | 0 | -1.785682 | 1.215840 | -0.844401 |
| 41 | 6 | 0 | -0.073844 | 2.705405 | -1.723453 |
| 42 | 6 | 0 | 0.617775 | 3.259018 | 2.501620 |
| 43 | 1 | 0 | -0.730723 | 3.606522 | 0.885699 |
| 44 | 1 | 0 | 2.090451 | 2.606557 | 3.941816 |
| 45 | 6 | 0 | -3.450645 | -2.198919 | 0.303097 |
| 46 | 6 | 0 | -3.591076 | -1.236695 | -2.005670 |
| 47 | 6 | 0 | -4.700857 | -0.194787 | 0.116812 |
| 48 | 6 | 0 | -4.183358 | -0.292322 | 1.560358 |
| 49 | 6 | 0 | -3.168755 | 1.972273 | 1.558350 |
| 50 | 6 | 0 | -1.864631 | 0.129755 | 2.614623 |
| 51 | 6 | 0 | -2.634774 | 1.729298 | -1.836285 |
| 52 | 6 | 0 | -0.906634 | 3.118868 | -2.760606 |
| 53 | 1 | 0 | 0.912646 | 3.146746 | -1.632606 |
| 54 | 1 | 0 | 0.293673 | 4.134758 | 3.055802 |
| 55 | 1 | 0 | -4.134547 | -2.903371 | -0.183227 |
| 56 | 1 | 0 | -2.465334 | -2.660521 | 0.336581 |
| 57 | 6 | 0 | -3.967236 | -1.805406 | 1.708252 |
| 58 | 1 | 0 | -4.582145 | -1.678319 | -2.162942 |
| 59 | 1 | 0 | -2.850373 | -1.984608 | -2.294560 |
| 60 | 1 | 0 | -3.490707 | -0.405962 | -2.704353 |
| 61 | 1 | 0 | -4.894963 | 0.825700 | -0.221930 |
| 62 | 1 | 0 | -5.608546 | -0.785106 | -0.053879 |
| 63 | 1 | 0 | -4.831431 | 0.138001 | 2.329936 |
| 64 | 1 | 0 | -3.678510 | 2.161945 | 2.509956 |
| 65 | 1 | 0 | -3.827517 | 2.313897 | 0.755865 |
| 66 | 1 | 0 | -2.274982 | 2.588760 | 1.540744 |
| 67 | 1 | 0 | -0.993051 | 0.786960 | 2.604725 |
| 68 | 1 | 0 | -1.499226 | -0.895256 | 2.556724 |
| 69 | 1 | 0 | -2.379619 | 0.267728 | 3.573532 |
| 70 | 1 | 0 | -3.658955 | 1.387106 | -1.871468 |

| | | | | | |
|---|---|---|---|---|---|
| 71 | 6 | 0 | -2.217637 | 2.659987 | -2.787725 |
| 72 | 1 | 0 | -0.545228 | 3.825879 | -3.501291 |
| 73 | 1 | 0 | -4.916619 | -2.301433 | 1.938858 |
| 74 | 1 | 0 | -3.264901 | -2.063300 | 2.502666 |
| 75 | 1 | 0 | -2.916111 | 3.008988 | -3.542565 |
| 76 | 1 | 0 | 0.240507 | -3.662075 | -0.978937 |
| 77 | 8 | 0 | 0.348216 | -2.978460 | -1.732967 |
| 78 | 1 | 0 | -0.224681 | -3.115140 | -2.511324 |
| 79 | 8 | 0 | -0.653472 | -0.985310 | -2.435844 |
| 80 | 1 | 0 | -1.061923 | -0.111218 | -2.521833 |
| 81 | 17 | 0 | 0.087724 | -3.344104 | 0.871462 |

BIFOXSiCl(OH) $\mathbf{8_{eq}}$ to BIFOXSi(OH)$_2$ **9** TS$_{front2}$ **8eq**

HF=-2295.796119
Thermal correction to Gibbs Free Energy= 0.607763
NImag=1
Frequencies -- -242.4084

| | | | | | |
|---|---|---|---|---|---|
| 1 | 1 | 0 | -5.566429 | -2.243600 | -0.515697 |
| 2 | 6 | 0 | -4.587712 | -1.897179 | -0.164768 |
| 3 | 1 | 0 | -4.294411 | -2.546055 | 0.662368 |
| 4 | 6 | 0 | -3.550467 | -1.878087 | -1.317128 |
| 5 | 6 | 0 | -4.668731 | -0.412804 | 0.224221 |
| 6 | 1 | 0 | -3.994087 | -2.229211 | -2.255244 |
| 7 | 1 | 0 | -2.682951 | -2.501651 | -1.123437 |
| 8 | 6 | 0 | -3.174020 | -0.373349 | -1.503988 |
| 9 | 6 | 0 | -4.548955 | 0.254041 | -1.152370 |
| 10 | 1 | 0 | -5.562101 | -0.157373 | 0.803030 |
| 11 | 6 | 0 | -3.372317 | 0.067937 | 0.941504 |
| 12 | 6 | 0 | -2.275670 | 0.055404 | -0.261217 |
| 13 | 6 | 0 | -2.647536 | -0.126172 | -2.920309 |
| 14 | 1 | 0 | -4.578161 | 1.343096 | -1.118213 |
| 15 | 1 | 0 | -5.319857 | -0.079894 | -1.857011 |
| 16 | 6 | 0 | -3.650056 | 1.473397 | 1.509782 |
| 17 | 6 | 0 | -2.965825 | -0.835599 | 2.110574 |
| 18 | 14 | 0 | 0.002126 | -1.575576 | -0.650951 |
| 19 | 1 | 0 | -1.832994 | -0.811228 | -3.153128 |
| 20 | 8 | 0 | -1.310938 | -0.958019 | 0.061778 |
| 21 | 6 | 0 | -1.548146 | 1.406967 | -0.355921 |
| 22 | 1 | 0 | -3.459582 | -0.315193 | -3.633134 |
| 23 | 1 | 0 | -2.286900 | 0.888286 | -3.093633 |
| 24 | 1 | 0 | -4.403360 | 1.387642 | 2.300997 |

| | | | | | |
|---|---|---|---|---|---|
| 25 | 1 | 0 | -4.030538 | 2.173729 | 0.762397 |
| 26 | 1 | 0 | -2.757775 | 1.926247 | 1.945665 |
| 27 | 1 | 0 | -2.096116 | -0.438790 | 2.635995 |
| 28 | 1 | 0 | -2.695522 | -1.841636 | 1.797381 |
| 29 | 1 | 0 | -3.800317 | -0.897010 | 2.820454 |
| 30 | 8 | 0 | 1.180620 | -0.481953 | -0.897579 |
| 31 | 6 | 0 | -0.445509 | 1.781267 | 0.458237 |
| 32 | 6 | 0 | -2.127947 | 2.415409 | -1.147329 |
| 33 | 6 | 0 | 2.257566 | 0.118102 | -0.160000 |
| 34 | 6 | 0 | 0.391190 | 0.995654 | 1.452615 |
| 35 | 6 | 0 | -0.116646 | 3.150983 | 0.529914 |
| 36 | 1 | 0 | -2.967062 | 2.170357 | -1.776634 |
| 37 | 6 | 0 | -1.715558 | 3.742568 | -1.134307 |
| 38 | 6 | 0 | 3.499704 | -0.897977 | -0.206904 |
| 39 | 6 | 0 | 2.840540 | 1.365199 | -1.002003 |
| 40 | 6 | 0 | 1.701457 | 0.475841 | 1.230642 |
| 41 | 6 | 0 | -0.047750 | 1.128552 | 2.778346 |
| 42 | 6 | 0 | -0.714631 | 4.129132 | -0.253220 |
| 43 | 1 | 0 | 0.632246 | 3.446610 | 1.255450 |
| 44 | 1 | 0 | -2.209048 | 4.465752 | -1.777228 |
| 45 | 6 | 0 | 3.589412 | -1.455544 | -1.669600 |
| 46 | 6 | 0 | 3.575683 | -2.030181 | 0.840807 |
| 47 | 6 | 0 | 4.712218 | 0.056365 | -0.252418 |
| 48 | 6 | 0 | 4.231497 | 0.847504 | -1.476172 |
| 49 | 6 | 0 | 3.114803 | 2.603038 | -0.128229 |
| 50 | 6 | 0 | 1.923470 | 1.788753 | -2.155819 |
| 51 | 6 | 0 | 2.508820 | 0.256198 | 2.353778 |
| 52 | 6 | 0 | 0.744095 | 0.809730 | 3.876332 |
| 53 | 1 | 0 | -1.033296 | 1.545328 | 2.949496 |
| 54 | 1 | 0 | -0.407029 | 5.166786 | -0.163649 |
| 55 | 1 | 0 | 4.313518 | -2.277040 | -1.674356 |
| 56 | 1 | 0 | 2.644778 | -1.840132 | -2.056704 |
| 57 | 6 | 0 | 4.100036 | -0.267546 | -2.523293 |
| 58 | 1 | 0 | 3.830714 | -2.988977 | 0.367069 |
| 59 | 1 | 0 | 2.657831 | -2.157739 | 1.419340 |
| 60 | 1 | 0 | 4.374373 | -1.851634 | 1.566454 |
| 61 | 1 | 0 | 4.838736 | 0.655029 | 0.651335 |
| 62 | 1 | 0 | 5.645005 | -0.491834 | -0.430125 |
| 63 | 1 | 0 | 4.875058 | 1.671345 | -1.797764 |
| 64 | 1 | 0 | 3.679606 | 3.332983 | -0.719724 |
| 65 | 1 | 0 | 3.693835 | 2.378829 | 0.770394 |
| 66 | 1 | 0 | 2.192171 | 3.080969 | 0.184099 |
| 67 | 1 | 0 | 1.006456 | 2.247698 | -1.781808 |
| 68 | 1 | 0 | 1.630916 | 0.951405 | -2.790161 |

| | | | | | |
|---|---|---|---|---|---|
| 69 | 1 | 0 | 2.443460 | 2.529108 | -2.776116 |
| 70 | 1 | 0 | 3.532251 | -0.056236 | 2.211988 |
| 71 | 6 | 0 | 2.054898 | 0.409198 | 3.659694 |
| 72 | 1 | 0 | 0.349428 | 0.918686 | 4.882110 |
| 73 | 1 | 0 | 5.077006 | -0.492714 | -2.964046 |
| 74 | 1 | 0 | 3.426298 | -0.011599 | -3.342879 |
| 75 | 1 | 0 | 2.722657 | 0.203769 | 4.491074 |
| 76 | 17 | 0 | 0.085328 | -2.682170 | 1.911158 |
| 77 | 8 | 0 | -0.521233 | -2.160683 | -2.105938 |
| 78 | 1 | 0 | 0.031020 | -2.865928 | -2.468557 |
| 79 | 1 | 0 | 0.886970 | -3.423160 | 0.492375 |
| 80 | 8 | 0 | 1.083206 | -3.185501 | -0.509369 |
| 81 | 1 | 0 | 2.032135 | -2.961616 | -0.556432 |

BIFOXSiCl(OH) $\mathbf{8_{ax}}$ to BIFOXSi(OH)$_2$ **9** TS$_{side}$ **8ax**

HF=-2295.8158455
Thermal correction to Gibbs Free Energy= 0.609060
NImag=1
Frequencies -- -162.8018

| | | | | | |
|---|---|---|---|---|---|
| 1 | 1 | 0 | 5.623777 | 1.925985 | 0.119717 |
| 2 | 6 | 0 | 4.633092 | 1.535025 | 0.376545 |
| 3 | 1 | 0 | 4.380669 | 1.927291 | 1.363186 |
| 4 | 6 | 0 | 3.584927 | 1.905979 | -0.704598 |
| 5 | 6 | 0 | 4.644602 | -0.003344 | 0.311163 |
| 6 | 1 | 0 | 4.053515 | 2.459304 | -1.525911 |
| 7 | 1 | 0 | 2.780335 | 2.522809 | -0.314950 |
| 8 | 6 | 0 | 3.096312 | 0.542406 | -1.277121 |
| 9 | 6 | 0 | 4.439402 | -0.230648 | -1.192833 |
| 10 | 1 | 0 | 5.542345 | -0.456486 | 0.743557 |
| 11 | 6 | 0 | 3.348027 | -0.615771 | 0.919183 |
| 12 | 6 | 0 | 2.220042 | -0.170173 | -0.159892 |
| 13 | 6 | 0 | 2.449989 | 0.703868 | -2.667546 |
| 14 | 1 | 0 | 4.397384 | -1.282215 | -1.478157 |
| 15 | 1 | 0 | 5.208394 | 0.254031 | -1.805701 |
| 16 | 6 | 0 | 3.538462 | -2.141404 | 1.021429 |
| 17 | 6 | 0 | 3.039026 | -0.098901 | 2.330376 |
| 18 | 14 | 0 | 0.001210 | 1.568675 | 0.270019 |
| 19 | 1 | 0 | 1.427104 | 0.321492 | -2.699663 |
| 20 | 8 | 0 | 1.385842 | 0.778678 | 0.492921 |
| 21 | 6 | 0 | 1.389913 | -1.377807 | -0.629851 |

| | | | | | |
|---|---|---|---|---|---|
| 22 | 1 | 0 | 2.412854 | 1.757011 | -2.958658 |
| 23 | 1 | 0 | 3.019086 | 0.174556 | -3.440110 |
| 24 | 1 | 0 | 4.335420 | -2.349641 | 1.744390 |
| 25 | 1 | 0 | 3.818615 | -2.605254 | 0.073293 |
| 26 | 1 | 0 | 2.631873 | -2.643061 | 1.367997 |
| 27 | 1 | 0 | 2.154785 | -0.589875 | 2.743686 |
| 28 | 1 | 0 | 2.849907 | 0.973236 | 2.356474 |
| 29 | 1 | 0 | 3.889486 | -0.319057 | 2.987283 |
| 30 | 8 | 0 | -1.120289 | 0.682239 | -0.588585 |
| 31 | 6 | 0 | 0.286927 | -1.929130 | 0.072114 |
| 32 | 6 | 0 | 1.873060 | -2.106514 | -1.732377 |
| 33 | 6 | 0 | -2.277562 | 0.001974 | -0.080338 |
| 34 | 6 | 0 | -0.531026 | -1.422429 | 1.246293 |
| 35 | 6 | 0 | -0.110562 | -3.242224 | -0.256759 |
| 36 | 1 | 0 | 2.678862 | -1.696136 | -2.318051 |
| 37 | 6 | 0 | 1.394974 | -3.358347 | -2.100654 |
| 38 | 6 | 0 | -3.460873 | 1.075575 | 0.140814 |
| 39 | 6 | 0 | -2.898240 | -0.910962 | -1.262913 |
| 40 | 6 | 0 | -1.813514 | -0.800028 | 1.153037 |
| 41 | 6 | 0 | -0.140260 | -1.958393 | 2.482933 |
| 42 | 6 | 0 | 0.416951 | -3.961665 | -1.320845 |
| 43 | 1 | 0 | -0.867736 | -3.704799 | 0.366199 |
| 44 | 1 | 0 | 1.816091 | -3.863411 | -2.965156 |
| 45 | 6 | 0 | -3.459008 | 2.033907 | -1.088768 |
| 46 | 6 | 0 | -3.556652 | 1.933359 | 1.410273 |
| 47 | 6 | 0 | -4.718741 | 0.249590 | -0.216810 |
| 48 | 6 | 0 | -4.226936 | -0.178609 | -1.606850 |
| 49 | 6 | 0 | -3.283874 | -2.327777 | -0.796775 |
| 50 | 6 | 0 | -1.961609 | -1.058382 | -2.468501 |
| 51 | 6 | 0 | -2.661258 | -0.905291 | 2.262244 |
| 52 | 6 | 0 | -0.966265 | -1.951696 | 3.602974 |
| 53 | 1 | 0 | 0.826697 | -2.446543 | 2.543754 |
| 54 | 1 | 0 | 0.060002 | -4.964817 | -1.534356 |
| 55 | 1 | 0 | -4.149071 | 2.855680 | -0.871475 |
| 56 | 1 | 0 | -2.482643 | 2.492689 | -1.253817 |
| 57 | 6 | 0 | -3.969464 | 1.181659 | -2.276092 |
| 58 | 1 | 0 | -4.595200 | 2.272049 | 1.516662 |
| 59 | 1 | 0 | -2.925191 | 2.819048 | 1.327695 |
| 60 | 1 | 0 | -3.262700 | 1.429811 | 2.327365 |
| 61 | 1 | 0 | -4.926513 | -0.582328 | 0.458060 |
| 62 | 1 | 0 | -5.611264 | 0.885121 | -0.252570 |
| 63 | 1 | 0 | -4.901684 | -0.823373 | -2.177974 |
| 64 | 1 | 0 | -3.871666 | -2.811879 | -1.585477 |
| 65 | 1 | 0 | -3.880655 | -2.335272 | 0.117417 |

| | | | | | |
|---|---|---|---|---|---|
| 66 | 1 | 0 | -2.405179 | -2.941688 | -0.621769 |
| 67 | 1 | 0 | -1.083616 | -1.655041 | -2.218757 |
| 68 | 1 | 0 | -1.604616 | -0.099708 | -2.847077 |
| 69 | 1 | 0 | -2.496686 | -1.565450 | -3.280875 |
| 70 | 1 | 0 | -3.673247 | -0.533946 | 2.186698 |
| 71 | 6 | 0 | -2.260264 | -1.463295 | 3.473340 |
| 72 | 1 | 0 | -0.616994 | -2.372523 | 4.541341 |
| 73 | 1 | 0 | -4.901016 | 1.589003 | -2.683549 |
| 74 | 1 | 0 | -3.261077 | 1.118985 | -3.105295 |
| 75 | 1 | 0 | -2.957099 | -1.502290 | 4.305705 |
| 76 | 8 | 0 | -0.748907 | 1.836093 | 1.705771 |
| 77 | 1 | 0 | -0.361039 | 2.581658 | 2.188721 |
| 78 | 17 | 0 | 0.869805 | 3.937923 | 0.653608 |
| 79 | 8 | 0 | 0.226725 | 2.400836 | -1.462626 |
| 80 | 1 | 0 | -0.575125 | 2.228003 | -1.991125 |
| 81 | 1 | 0 | 0.394636 | 3.364798 | -1.299359 |

BIFOXSi$(OH)_2$ **9**

HF=-1835.0468439
Thermal correction to Gibbs Free Energy= 0.599402
NImag=0

| | | | | | |
|---|---|---|---|---|---|
| 1 | 14 | 0 | 0.043969 | -1.618470 | 0.703806 |
| 2 | 8 | 0 | 1.001473 | -0.882301 | -0.410549 |
| 3 | 8 | 0 | -1.375464 | -0.814914 | 0.826736 |
| 4 | 8 | 0 | -0.059922 | -3.173646 | 0.159032 |
| 5 | 8 | 0 | 0.556555 | -1.709574 | 2.268252 |
| 6 | 6 | 0 | 2.183843 | -0.134170 | -0.085365 |
| 7 | 6 | 0 | -2.204574 | -0.154176 | -0.112614 |
| 8 | 1 | 0 | -0.475486 | -3.788171 | 0.779699 |
| 9 | 6 | 0 | 3.366546 | -1.169355 | 0.221118 |
| 10 | 6 | 0 | 2.754095 | 0.615881 | -1.393751 |
| 11 | 6 | 0 | 1.748999 | 0.820644 | 1.050559 |
| 12 | 6 | 0 | -2.869517 | -1.206580 | -1.094415 |
| 13 | 6 | 0 | -3.495699 | 0.403509 | 0.702407 |
| 14 | 6 | 0 | -1.432255 | 1.005359 | -0.768542 |
| 15 | 6 | 0 | 3.315887 | -2.252959 | -0.906050 |
| 16 | 6 | 0 | 4.613116 | -0.380975 | -0.233275 |
| 17 | 6 | 0 | 3.455458 | -1.891238 | 1.568664 |
| 18 | 6 | 0 | 4.102624 | -0.118526 | -1.658983 |
| 19 | 6 | 0 | 3.106629 | 2.092611 | -1.137666 |
| 20 | 6 | 0 | 1.787448 | 0.550129 | -2.581615 |
| 21 | 6 | 0 | 0.449273 | 1.419971 | 1.087952 |

| | | | | | |
|---|---|---|---|---|---|
| 22 | 6 | 0 | 2.599419 | 1.058904 | 2.137187 |
| 23 | 6 | 0 | -3.317114 | -2.436301 | -0.244418 |
| 24 | 6 | 0 | -2.018963 | -1.656244 | -2.280511 |
| 25 | 6 | 0 | -4.271995 | -0.596818 | -1.357087 |
| 26 | 6 | 0 | -3.317403 | 0.258397 | 2.219421 |
| 27 | 6 | 0 | -3.822105 | 1.883909 | 0.431941 |
| 28 | 6 | 0 | -4.651873 | -0.470558 | 0.128531 |
| 29 | 6 | 0 | -0.392674 | 1.730374 | -0.132725 |
| 30 | 6 | 0 | -1.911860 | 1.521339 | -1.985319 |
| 31 | 6 | 0 | 3.873697 | -1.549297 | -2.167464 |
| 32 | 1 | 0 | 3.960831 | -3.084373 | -0.601890 |
| 33 | 1 | 0 | 2.311217 | -2.651554 | -1.038013 |
| 34 | 1 | 0 | 4.817424 | 0.525476 | 0.341808 |
| 35 | 1 | 0 | 5.512893 | -1.006780 | -0.208646 |
| 36 | 1 | 0 | 4.439938 | -2.371402 | 1.629153 |
| 37 | 1 | 0 | 2.701844 | -2.676802 | 1.645165 |
| 38 | 1 | 0 | 3.337597 | -1.257549 | 2.446288 |
| 39 | 1 | 0 | 4.759028 | 0.477421 | -2.300836 |
| 40 | 1 | 0 | 3.599667 | 2.496691 | -2.029626 |
| 41 | 1 | 0 | 3.785455 | 2.231052 | -0.292349 |
| 42 | 1 | 0 | 2.220718 | 2.694147 | -0.954670 |
| 43 | 1 | 0 | 2.309199 | 0.870955 | -3.491851 |
| 44 | 1 | 0 | 0.934232 | 1.212708 | -2.433057 |
| 45 | 1 | 0 | 1.394485 | -0.453985 | -2.741199 |
| 46 | 6 | 0 | 0.046712 | 2.071859 | 2.263621 |
| 47 | 6 | 0 | 2.185458 | 1.727118 | 3.288520 |
| 48 | 1 | 0 | 3.616283 | 0.695707 | 2.093772 |
| 49 | 1 | 0 | -3.607289 | -3.234012 | -0.936761 |
| 50 | 1 | 0 | -2.512372 | -2.825060 | 0.372942 |
| 51 | 6 | 0 | -4.532528 | -1.936837 | 0.579405 |
| 52 | 1 | 0 | -2.614264 | -2.305086 | -2.933886 |
| 53 | 1 | 0 | -1.159721 | -2.240703 | -1.937784 |
| 54 | 1 | 0 | -1.635279 | -0.831329 | -2.882812 |
| 55 | 1 | 0 | -4.293241 | 0.350773 | -1.893712 |
| 56 | 1 | 0 | -4.913658 | -1.301320 | -1.899322 |
| 57 | 1 | 0 | -2.547221 | 0.943981 | 2.584352 |
| 58 | 1 | 0 | -3.018155 | -0.745156 | 2.517993 |
| 59 | 1 | 0 | -4.259389 | 0.509188 | 2.722078 |
| 60 | 1 | 0 | -4.697400 | 2.166595 | 1.028244 |
| 61 | 1 | 0 | -4.052638 | 2.090659 | -0.614978 |
| 62 | 1 | 0 | -2.994100 | 2.539681 | 0.716472 |
| 63 | 1 | 0 | -5.631516 | -0.026647 | 0.332422 |
| 64 | 6 | 0 | -0.071731 | 3.008058 | -0.634072 |
| 65 | 6 | 0 | -1.504358 | 2.738501 | -2.519796 |

| | | | | | |
|---|---|---|---|---|---|
| 66 | 1 | 0 | -2.660763 | 0.970116 | -2.530864 |
| 67 | 1 | 0 | 4.825144 | -1.991988 | -2.483594 |
| 68 | 1 | 0 | 3.196884 | -1.598833 | -3.023277 |
| 69 | 1 | 0 | -0.937269 | 2.529895 | 2.280037 |
| 70 | 6 | 0 | 0.879284 | 2.197336 | 3.374178 |
| 71 | 1 | 0 | 2.880441 | 1.863057 | 4.112043 |
| 72 | 1 | 0 | -4.393474 | -2.048497 | 1.656602 |
| 73 | 1 | 0 | -5.443955 | -2.484114 | 0.314696 |
| 74 | 1 | 0 | 0.640385 | 3.603374 | -0.072632 |
| 75 | 6 | 0 | -0.609120 | 3.524772 | -1.806149 |
| 76 | 1 | 0 | -1.918765 | 3.077145 | -3.464970 |
| 77 | 1 | 0 | 0.523499 | 2.699354 | 4.269165 |
| 78 | 1 | 0 | -0.315060 | 4.509551 | -2.156565 |
| 79 | 1 | 0 | 0.448851 | -0.879932 | 2.757183 |

### b. Kondo-silanes

dichlorodi(naphthalen-1-yl)silane **13**

HF=-1980.4604433
Thermal correction to Gibbs Free Energy= 0.220359
NImag=0

| | | | | | |
|---|---|---|---|---|---|
| 1 | 14 | 0 | 0.039826 | -1.091581 | 0.137046 |
| 2 | 6 | 0 | -1.745247 | -0.809108 | -0.331616 |
| 3 | 6 | 0 | -2.503205 | 0.295948 | 0.193226 |
| 4 | 6 | 0 | -2.359088 | -1.687271 | -1.211268 |
| 5 | 6 | 0 | -1.962261 | 1.253476 | 1.094002 |
| 6 | 6 | 0 | -3.870419 | 0.456449 | -0.210336 |
| 7 | 6 | 0 | -3.706516 | -1.524524 | -1.605108 |
| 8 | 1 | 0 | -1.801936 | -2.527333 | -1.611733 |
| 9 | 6 | 0 | -2.721430 | 2.300113 | 1.566615 |
| 10 | 1 | 0 | -0.931940 | 1.164979 | 1.418446 |
| 11 | 6 | 0 | -4.627647 | 1.545129 | 0.295828 |
| 12 | 6 | 0 | -4.443982 | -0.475592 | -1.113721 |
| 13 | 1 | 0 | -4.150835 | -2.234973 | -2.295748 |
| 14 | 6 | 0 | -4.068794 | 2.450659 | 1.166564 |
| 15 | 1 | 0 | -2.280698 | 3.015540 | 2.254644 |
| 16 | 1 | 0 | -5.662089 | 1.648400 | -0.021835 |
| 17 | 1 | 0 | -5.481550 | -0.341749 | -1.409509 |

| | | | | | |
|---|---|---|---|---|---|
| 18 | 1 | 0 | -4.657113 | 3.280027 | 1.547988 |
| 19 | 6 | 0 | 1.175782 | 0.279032 | -0.406476 |
| 20 | 6 | 0 | 2.584715 | 0.323337 | -0.126422 |
| 21 | 6 | 0 | 0.603922 | 1.294958 | -1.159737 |
| 22 | 6 | 0 | 3.262785 | -0.680748 | 0.614948 |
| 23 | 6 | 0 | 3.355750 | 1.427124 | -0.621460 |
| 24 | 6 | 0 | 1.368702 | 2.378672 | -1.646886 |
| 25 | 1 | 0 | -0.457531 | 1.271918 | -1.383100 |
| 26 | 6 | 0 | 4.615652 | -0.596940 | 0.857521 |
| 27 | 1 | 0 | 2.711005 | -1.530376 | 0.998555 |
| 28 | 6 | 0 | 4.747863 | 1.486131 | -0.352099 |
| 29 | 6 | 0 | 2.714150 | 2.441934 | -1.378717 |
| 30 | 1 | 0 | 0.884557 | 3.156290 | -2.230012 |
| 31 | 6 | 0 | 5.368878 | 0.496371 | 0.372880 |
| 32 | 1 | 0 | 5.108475 | -1.379638 | 1.426733 |
| 33 | 1 | 0 | 5.314698 | 2.331322 | -0.734378 |
| 34 | 1 | 0 | 3.312590 | 3.271847 | -1.746464 |
| 35 | 1 | 0 | 6.435047 | 0.549959 | 0.572815 |
| 36 | 17 | 0 | 0.158237 | -1.372180 | 2.204740 |
| 37 | 17 | 0 | 0.686591 | -2.882231 | -0.716004 |

dichlorodi(naphthalen-1-yl)silane **13** to $\mathbf{13_{ClOH}}$ $TS_{front}$ 13

HF=-2056.8616445
Thermal correction to Gibbs Free Energy= 0.241033
NImag=1
Frequencies -- -167.5153

| | | | | | |
|---|---|---|---|---|---|
| 1 | 6 | 0 | -1.848525 | -0.791494 | -0.169168 |
| 2 | 6 | 0 | -2.494839 | 0.464303 | 0.108600 |
| 3 | 6 | 0 | -2.623217 | -1.881569 | -0.542198 |
| 4 | 6 | 0 | -1.805508 | 1.636171 | 0.522349 |
| 5 | 6 | 0 | -3.919693 | 0.552206 | -0.048261 |
| 6 | 6 | 0 | -4.026481 | -1.794876 | -0.659730 |
| 7 | 1 | 0 | -2.148562 | -2.830822 | -0.767031 |
| 8 | 6 | 0 | -2.474240 | 2.819088 | 0.742094 |
| 9 | 1 | 0 | -0.739013 | 1.597823 | 0.690884 |
| 10 | 6 | 0 | -4.578934 | 1.788012 | 0.184490 |
| 11 | 6 | 0 | -4.657672 | -0.597679 | -0.427438 |
| 12 | 1 | 0 | -4.594873 | -2.674110 | -0.947543 |
| 13 | 6 | 0 | -3.873976 | 2.903740 | 0.567183 |
| 14 | 1 | 0 | -1.917608 | 3.694672 | 1.063180 |
| 15 | 1 | 0 | -5.657454 | 1.830463 | 0.055153 |
| 16 | 1 | 0 | -5.736517 | -0.511475 | -0.530881 |

| | | | | | |
|---|---|---|---|---|---|
| 17 | 1 | 0 | -4.387431 | 3.844412 | 0.743741 |
| 18 | 6 | 0 | 1.250065 | 0.203664 | -0.505613 |
| 19 | 6 | 0 | 2.615709 | 0.253736 | -0.080763 |
| 20 | 6 | 0 | 0.792907 | 1.121633 | -1.438676 |
| 21 | 6 | 0 | 3.176692 | -0.653284 | 0.855470 |
| 22 | 6 | 0 | 3.474832 | 1.262162 | -0.630759 |
| 23 | 6 | 0 | 1.639023 | 2.123747 | -1.964861 |
| 24 | 1 | 0 | -0.236898 | 1.085948 | -1.783425 |
| 25 | 6 | 0 | 4.501138 | -0.584151 | 1.220685 |
| 26 | 1 | 0 | 2.539122 | -1.394125 | 1.319826 |
| 27 | 6 | 0 | 4.836159 | 1.312580 | -0.231456 |
| 28 | 6 | 0 | 2.952022 | 2.190071 | -1.568429 |
| 29 | 1 | 0 | 1.242321 | 2.832422 | -2.685522 |
| 30 | 6 | 0 | 5.344462 | 0.408283 | 0.670794 |
| 31 | 1 | 0 | 4.899588 | -1.288668 | 1.944911 |
| 32 | 1 | 0 | 5.472043 | 2.084072 | -0.658135 |
| 33 | 1 | 0 | 3.614450 | 2.951645 | -1.971844 |
| 34 | 1 | 0 | 6.387903 | 0.456467 | 0.967774 |
| 35 | 14 | 0 | -0.014501 | -1.092863 | -0.063676 |
| 36 | 17 | 0 | 0.378180 | -2.456652 | -1.706689 |
| 37 | 8 | 0 | 0.304610 | -2.552224 | 1.051122 |
| 38 | 1 | 0 | 1.007853 | -3.170318 | 0.773903 |
| 39 | 1 | 0 | 0.414959 | -2.096808 | 1.973885 |
| 40 | 17 | 0 | 0.088096 | -0.314629 | 2.451850 |

dichlorodi(naphthalen-1-yl)silane **13** to $\mathbf{13}_{\mathbf{ClOH}}$ $TS_{side}$ 13

HF=-2056.8492045
Thermal correction to Gibbs Free Energy= 0.240761
NImag=1
Frequencies -- -176.0179

| | | | | | |
|---|---|---|---|---|---|
| 1 | 6 | 0 | -1.720998 | 0.478477 | 0.478878 |
| 2 | 6 | 0 | -2.332555 | -0.680779 | -0.110886 |
| 3 | 6 | 0 | -2.486636 | 1.311712 | 1.277966 |
| 4 | 6 | 0 | -1.629571 | -1.641970 | -0.894579 |
| 5 | 6 | 0 | -3.737520 | -0.898668 | 0.089754 |
| 6 | 6 | 0 | -3.853873 | 1.060239 | 1.518726 |
| 7 | 1 | 0 | -2.039567 | 2.205234 | 1.701662 |
| 8 | 6 | 0 | -2.274828 | -2.705438 | -1.486872 |
| 9 | 1 | 0 | -0.545940 | -1.604824 | -0.946132 |
| 10 | 6 | 0 | -4.373375 | -2.003049 | -0.536594 |
| 11 | 6 | 0 | -4.470174 | -0.011004 | 0.918251 |
| 12 | 1 | 0 | -4.418370 | 1.735925 | 2.153839 |

| | | | | | |
|---|---|---|---|---|---|
| 13 | 6 | 0 | -3.666765 | -2.882591 | -1.322541 |
| 14 | 1 | 0 | -1.704062 | -3.425291 | -2.066466 |
| 15 | 1 | 0 | -5.439689 | -2.141489 | -0.377026 |
| 16 | 1 | 0 | -5.531117 | -0.194970 | 1.066757 |
| 17 | 1 | 0 | -4.167253 | -3.721089 | -1.797083 |
| 18 | 6 | 0 | 1.260009 | -0.357577 | 0.488459 |
| 19 | 6 | 0 | 2.626152 | -0.347813 | 0.046423 |
| 20 | 6 | 0 | 0.831612 | -1.388160 | 1.310851 |
| 21 | 6 | 0 | 3.172890 | 0.694439 | -0.750724 |
| 22 | 6 | 0 | 3.495719 | -1.421612 | 0.432675 |
| 23 | 6 | 0 | 1.692615 | -2.440776 | 1.699437 |
| 24 | 1 | 0 | -0.194948 | -1.404629 | 1.665235 |
| 25 | 6 | 0 | 4.487644 | 0.669642 | -1.157429 |
| 26 | 1 | 0 | 2.549097 | 1.532652 | -1.041091 |
| 27 | 6 | 0 | 4.844719 | -1.423011 | -0.009155 |
| 28 | 6 | 0 | 2.993760 | -2.459689 | 1.260732 |
| 29 | 1 | 0 | 1.315499 | -3.231604 | 2.341306 |
| 30 | 6 | 0 | 5.333712 | -0.401910 | -0.789562 |
| 31 | 1 | 0 | 4.879895 | 1.482182 | -1.761943 |
| 32 | 1 | 0 | 5.487362 | -2.247585 | 0.289146 |
| 33 | 1 | 0 | 3.663756 | -3.266459 | 1.547513 |
| 34 | 1 | 0 | 6.368641 | -0.411060 | -1.118923 |
| 35 | 14 | 0 | 0.025043 | 1.011039 | 0.093817 |
| 36 | 17 | 0 | 0.782276 | 2.502853 | 1.345295 |
| 37 | 17 | 0 | -0.982848 | 3.041489 | -1.297120 |
| 38 | 8 | 0 | 0.260718 | 0.744255 | -1.792018 |
| 39 | 1 | 0 | -0.288656 | 1.538115 | -2.151265 |
| 40 | 1 | 0 | -0.116499 | -0.110280 | -2.084670 |

chlorodi(naphthalen-1-yl)silanol **13**$_{\mathbf{ClOH}}$

HF=-1596.0871036
Thermal correction to Gibbs Free Energy= 0.231269
NImag=0

| | | | | | |
|---|---|---|---|---|---|
| 1 | 14 | 0 | -0.037083 | 1.140379 | 0.377626 |
| 2 | 6 | 0 | 1.747597 | 0.887859 | -0.121529 |
| 3 | 6 | 0 | 2.464182 | -0.286869 | 0.302165 |
| 4 | 6 | 0 | 2.400060 | 1.818090 | -0.915271 |
| 5 | 6 | 0 | 1.878513 | -1.300151 | 1.111188 |
| 6 | 6 | 0 | 3.827275 | -0.462495 | -0.109149 |
| 7 | 6 | 0 | 3.744355 | 1.643623 | -1.315952 |
| 8 | 1 | 0 | 1.872329 | 2.707678 | -1.243315 |
| 9 | 6 | 0 | 2.594450 | -2.413910 | 1.488634 |

| | | | | | |
|---|---|---|---|---|---|
| 10 | 1 | 0 | 0.850872 | -1.196460 | 1.441083 |
| 11 | 6 | 0 | 4.538540 | -1.621416 | 0.298726 |
| 12 | 6 | 0 | 4.440390 | 0.527190 | -0.920731 |
| 13 | 1 | 0 | 4.219722 | 2.396557 | -1.937790 |
| 14 | 6 | 0 | 3.938123 | -2.579821 | 1.080773 |
| 15 | 1 | 0 | 2.122136 | -3.172846 | 2.105634 |
| 16 | 1 | 0 | 5.570856 | -1.735979 | -0.022415 |
| 17 | 1 | 0 | 5.474267 | 0.382943 | -1.224914 |
| 18 | 1 | 0 | 4.491050 | -3.463320 | 1.386384 |
| 19 | 6 | 0 | -1.176286 | -0.125078 | -0.366335 |
| 20 | 6 | 0 | -2.565940 | -0.246079 | -0.025374 |
| 21 | 6 | 0 | -0.644374 | -0.964231 | -1.334121 |
| 22 | 6 | 0 | -3.194515 | 0.571787 | 0.952083 |
| 23 | 6 | 0 | -3.365249 | -1.229543 | -0.697097 |
| 24 | 6 | 0 | -1.433106 | -1.936863 | -1.989478 |
| 25 | 1 | 0 | 0.405101 | -0.884263 | -1.600687 |
| 26 | 6 | 0 | -4.532893 | 0.430019 | 1.242860 |
| 27 | 1 | 0 | -2.605459 | 1.307388 | 1.487928 |
| 28 | 6 | 0 | -4.741466 | -1.352030 | -0.372259 |
| 29 | 6 | 0 | -2.764590 | -2.063404 | -1.676236 |
| 30 | 1 | 0 | -0.979940 | -2.578402 | -2.739550 |
| 31 | 6 | 0 | -5.317272 | -0.538974 | 0.575560 |
| 32 | 1 | 0 | -4.991015 | 1.067072 | 1.993998 |
| 33 | 1 | 0 | -5.332592 | -2.103050 | -0.890539 |
| 34 | 1 | 0 | -3.382349 | -2.805788 | -2.175823 |
| 35 | 1 | 0 | -6.371887 | -0.639540 | 0.815621 |
| 36 | 17 | 0 | -0.618533 | 3.048464 | -0.277053 |
| 37 | 8 | 0 | -0.219996 | 1.095528 | 2.030795 |
| 38 | 1 | 0 | 0.424331 | 1.618349 | 2.530411 |

chlorodi(naphthalen-1-yl)silanol **$13_{ClOH}$** to di(naphthalen-1-yl)silanediol **1** $TS_{front}$ **$13_{ClOH}$**

HF=-1672.4874734
Thermal correction to Gibbs Free Energy= 0.253637
NImag=1
Frequencies -- -137.2984

| | | | | | |
|---|---|---|---|---|---|
| 1 | 6 | 0 | 1.846112 | -0.747651 | -0.078352 |
| 2 | 6 | 0 | 2.457123 | 0.543675 | -0.262620 |
| 3 | 6 | 0 | 2.654820 | -1.823622 | 0.249298 |
| 4 | 6 | 0 | 1.723345 | 1.721337 | -0.577678 |
| 5 | 6 | 0 | 3.877210 | 0.674388 | -0.094567 |
| 6 | 6 | 0 | 4.054556 | -1.696210 | 0.386548 |

| | | | | | |
|---|---|---|---|---|---|
| 7 | 1 | 0 | 2.205651 | -2.791444 | 0.441399 |
| 8 | 6 | 0 | 2.345392 | 2.941326 | -0.712991 |
| 9 | 1 | 0 | 0.651896 | 1.660865 | -0.715615 |
| 10 | 6 | 0 | 4.489961 | 1.946728 | -0.244211 |
| 11 | 6 | 0 | 4.651725 | -0.470980 | 0.220503 |
| 12 | 1 | 0 | 4.648692 | -2.569968 | 0.637993 |
| 13 | 6 | 0 | 3.744124 | 3.060862 | -0.545695 |
| 14 | 1 | 0 | 1.754858 | 3.821571 | -0.950840 |
| 15 | 1 | 0 | 5.566513 | 2.020500 | -0.111322 |
| 16 | 1 | 0 | 5.726901 | -0.357015 | 0.335639 |
| 17 | 1 | 0 | 4.222844 | 4.029690 | -0.655330 |
| 18 | 6 | 0 | -1.290233 | 0.041170 | 0.459956 |
| 19 | 6 | 0 | -2.621578 | 0.216986 | -0.038967 |
| 20 | 6 | 0 | -0.886879 | 0.753479 | 1.577056 |
| 21 | 6 | 0 | -3.138446 | -0.512198 | -1.147094 |
| 22 | 6 | 0 | -3.492454 | 1.154251 | 0.611462 |
| 23 | 6 | 0 | -1.746850 | 1.672766 | 2.217456 |
| 24 | 1 | 0 | 0.112700 | 0.607321 | 1.976390 |
| 25 | 6 | 0 | -4.422829 | -0.310204 | -1.601374 |
| 26 | 1 | 0 | -2.520776 | -1.265073 | -1.626109 |
| 27 | 6 | 0 | -4.810242 | 1.339235 | 0.117249 |
| 28 | 6 | 0 | -3.020197 | 1.873088 | 1.739645 |
| 29 | 1 | 0 | -1.393578 | 2.219263 | 3.086880 |
| 30 | 6 | 0 | -5.267954 | 0.630130 | -0.968856 |
| 31 | 1 | 0 | -4.793963 | -0.885481 | -2.444783 |
| 32 | 1 | 0 | -5.456260 | 2.054090 | 0.620908 |
| 33 | 1 | 0 | -3.688228 | 2.581904 | 2.222441 |
| 34 | 1 | 0 | -6.279121 | 0.780874 | -1.335327 |
| 35 | 8 | 0 | -0.078687 | -0.430183 | -2.005504 |
| 36 | 1 | 0 | -0.882539 | 0.065606 | -2.215073 |
| 37 | 14 | 0 | 0.014511 | -0.978100 | -0.403007 |
| 38 | 8 | 0 | -0.355987 | -2.707962 | -1.132756 |
| 39 | 1 | 0 | 0.139124 | -2.886145 | -1.954296 |
| 40 | 1 | 0 | -0.210189 | -3.360535 | -0.386791 |
| 41 | 17 | 0 | -0.200619 | -2.645812 | 1.528318 |

chlorodi(naphthalen-1-yl)silanol **13**$_{\mathbf{ClOH}}$ to di(naphthalen-1-yl)silanediol **1** TS$_{side}$ **13**$_{\mathbf{ClOH}}$

HF=-1672.4883422
Thermal correction to Gibbs Free Energy= 0.255458
NImag=1
Frequencies -- -119.7494

| | | | | | |
|---|---|---|---|---|---|
| 1 | 6 | 0 | 1.651865 | 0.660487 | -0.360388 |
| 2 | 6 | 0 | 2.490314 | -0.327957 | 0.258758 |
| 3 | 6 | 0 | 2.100578 | 1.300831 | -1.503142 |
| 4 | 6 | 0 | 2.115405 | -1.037166 | 1.435158 |
| 5 | 6 | 0 | 3.764512 | -0.629791 | -0.330337 |
| 6 | 6 | 0 | 3.354481 | 1.002160 | -2.079600 |
| 7 | 1 | 0 | 1.485843 | 2.063843 | -1.969211 |
| 8 | 6 | 0 | 2.944523 | -1.986233 | 1.988644 |
| 9 | 1 | 0 | 1.165592 | -0.817202 | 1.909973 |
| 10 | 6 | 0 | 4.596591 | -1.611676 | 0.269645 |
| 11 | 6 | 0 | 4.167875 | 0.055304 | -1.504602 |
| 12 | 1 | 0 | 3.670490 | 1.527385 | -2.976251 |
| 13 | 6 | 0 | 4.198255 | -2.279629 | 1.403143 |
| 14 | 1 | 0 | 2.634456 | -2.512737 | 2.886772 |
| 15 | 1 | 0 | 5.558911 | -1.825062 | -0.189187 |
| 16 | 1 | 0 | 5.135049 | -0.183066 | -1.940351 |
| 17 | 1 | 0 | 4.843006 | -3.029563 | 1.852375 |
| 18 | 6 | 0 | -1.162075 | -0.478278 | -0.145862 |
| 19 | 6 | 0 | -2.589920 | -0.448938 | 0.029123 |
| 20 | 6 | 0 | -0.599780 | -1.590340 | -0.747628 |
| 21 | 6 | 0 | -3.265654 | 0.647841 | 0.640937 |
| 22 | 6 | 0 | -3.389465 | -1.542093 | -0.447056 |
| 23 | 6 | 0 | -1.382734 | -2.687753 | -1.180216 |
| 24 | 1 | 0 | 0.472683 | -1.632388 | -0.904036 |
| 25 | 6 | 0 | -4.639808 | 0.676251 | 0.746352 |
| 26 | 1 | 0 | -2.681577 | 1.443738 | 1.093004 |
| 27 | 6 | 0 | -4.801228 | -1.484419 | -0.316334 |
| 28 | 6 | 0 | -2.748383 | -2.659878 | -1.042512 |
| 29 | 1 | 0 | -0.894723 | -3.544154 | -1.636880 |
| 30 | 6 | 0 | -5.418798 | -0.396414 | 0.256955 |
| 31 | 1 | 0 | -5.126570 | 1.520398 | 1.226486 |
| 32 | 1 | 0 | -5.389711 | -2.322116 | -0.682559 |
| 33 | 1 | 0 | -3.360185 | -3.488179 | -1.391270 |
| 34 | 1 | 0 | -6.500680 | -0.364907 | 0.346922 |
| 35 | 8 | 0 | -0.373853 | 1.039194 | 1.926849 |
| 36 | 1 | 0 | -0.008894 | 1.828015 | 2.360465 |
| 37 | 14 | 0 | -0.064639 | 1.016292 | 0.289568 |
| 38 | 8 | 0 | -1.038015 | 1.886240 | -1.240616 |
| 39 | 1 | 0 | -1.025932 | 2.856507 | -1.060219 |
| 40 | 17 | 0 | 0.344569 | 3.423736 | 0.506304 |
| 41 | 1 | 0 | -1.954872 | 1.550568 | -1.303228 |

di(naphthalen-1-yl)silanediol **1**

HF=-1211.7094702
Thermal correction to Gibbs Free Energy= 0.243783
NImag=0

| | | | | | |
|---|---|---|---|---|---|
| 1 | 14 | 0 | 0.095655 | -1.234555 | 0.378628 |
| 2 | 6 | 0 | -1.678756 | -1.001736 | -0.154514 |
| 3 | 6 | 0 | -2.521035 | 0.071583 | 0.295109 |
| 4 | 6 | 0 | -2.175857 | -1.884228 | -1.102158 |
| 5 | 6 | 0 | -2.097078 | 1.028520 | 1.258048 |
| 6 | 6 | 0 | -3.840594 | 0.207362 | -0.251271 |
| 7 | 6 | 0 | -3.482359 | -1.760849 | -1.628264 |
| 8 | 1 | 0 | -1.542555 | -2.689939 | -1.462966 |
| 9 | 6 | 0 | -2.920490 | 2.061140 | 1.646638 |
| 10 | 1 | 0 | -1.116228 | 0.923585 | 1.707011 |
| 11 | 6 | 0 | -4.666861 | 1.280876 | 0.173603 |
| 12 | 6 | 0 | -4.294363 | -0.733737 | -1.211996 |
| 13 | 1 | 0 | -3.834854 | -2.475717 | -2.366416 |
| 14 | 6 | 0 | -4.217991 | 2.193133 | 1.099482 |
| 15 | 1 | 0 | -2.573421 | 2.779098 | 2.384388 |
| 16 | 1 | 0 | -5.664065 | 1.368241 | -0.250888 |
| 17 | 1 | 0 | -5.297831 | -0.622360 | -1.615900 |
| 18 | 1 | 0 | -4.857543 | 3.012342 | 1.415661 |
| 19 | 6 | 0 | 1.241525 | 0.045561 | -0.353404 |
| 20 | 6 | 0 | 2.657063 | 0.046878 | -0.110324 |
| 21 | 6 | 0 | 0.688258 | 1.094016 | -1.071046 |
| 22 | 6 | 0 | 3.315358 | -0.998996 | 0.594575 |
| 23 | 6 | 0 | 3.453371 | 1.141417 | -0.583998 |
| 24 | 6 | 0 | 1.477184 | 2.161576 | -1.559672 |
| 25 | 1 | 0 | -0.380825 | 1.110685 | -1.260105 |
| 26 | 6 | 0 | 4.669826 | -0.953352 | 0.840327 |
| 27 | 1 | 0 | 2.746209 | -1.871577 | 0.900292 |
| 28 | 6 | 0 | 4.847263 | 1.158089 | -0.316556 |
| 29 | 6 | 0 | 2.828421 | 2.187106 | -1.313186 |
| 30 | 1 | 0 | 1.005966 | 2.963584 | -2.120635 |
| 31 | 6 | 0 | 5.445392 | 0.138604 | 0.387171 |
| 32 | 1 | 0 | 5.149702 | -1.767914 | 1.375594 |
| 33 | 1 | 0 | 5.435708 | 1.996418 | -0.681478 |
| 34 | 1 | 0 | 3.441787 | 3.010060 | -1.672247 |
| 35 | 1 | 0 | 6.512803 | 0.163516 | 0.586816 |
| 36 | 8 | 0 | 0.129161 | -1.151131 | 2.048169 |
| 37 | 1 | 0 | 1.011792 | -1.002966 | 2.419016 |
| 38 | 8 | 0 | 0.633344 | -2.728422 | -0.140640 |
| 39 | 1 | 0 | 0.154486 | -3.474633 | 0.249460 |

### c. glycol-silanes

$[CH_2O]_2SiCl_2$

HF=-1439.0996514
Thermal correction to Gibbs Free Energy= 0.034383
NImag=0

| | | | | | |
|---|---|---|---|---|---|
| 1 | 14 | 0 | -0.204228 | -0.000020 | -0.000004 |
| 2 | 8 | 0 | 0.861880 | 0.001303 | -1.261716 |
| 3 | 8 | 0 | 0.861874 | -0.001408 | 1.261714 |
| 4 | 17 | 0 | -1.403498 | -1.656669 | 0.059959 |
| 5 | 17 | 0 | -1.403343 | 1.656768 | -0.059961 |
| 6 | 6 | 0 | 2.182184 | -0.242475 | -0.731840 |
| 7 | 1 | 0 | 2.394862 | -1.316143 | -0.792918 |
| 8 | 1 | 0 | 2.904816 | 0.301695 | -1.344740 |
| 9 | 6 | 0 | 2.182173 | 0.242403 | 0.731853 |
| 10 | 1 | 0 | 2.394828 | 1.316076 | 0.792930 |
| 11 | 1 | 0 | 2.904815 | -0.301753 | 1.344754 |

$[CH_2O]_2SiCl_2$ to $[CH_2O]_2SiCl(OH)$ TS

HF=-1515.5045843
Thermal correction to Gibbs Free Energy= 0.052212
NImag=1
Frequencies -- -542.4420

| | | | | | |
|---|---|---|---|---|---|
| 1 | 14 | 0 | -0.089418 | 0.161017 | 0.113565 |
| 2 | 8 | 0 | -0.695409 | -0.996653 | -0.890077 |
| 3 | 8 | 0 | -1.546680 | 0.696434 | 0.735825 |
| 4 | 17 | 0 | 0.803082 | 1.895403 | -0.515565 |
| 5 | 17 | 0 | 2.225039 | -1.112919 | -0.239748 |
| 6 | 6 | 0 | -2.136916 | -0.899452 | -0.925884 |
| 7 | 1 | 0 | -2.422226 | -0.292293 | -1.792029 |
| 8 | 1 | 0 | -2.539280 | -1.908272 | -1.043705 |
| 9 | 6 | 0 | -2.575778 | -0.238131 | 0.390323 |
| 10 | 1 | 0 | -2.677406 | -0.985325 | 1.188833 |
| 11 | 1 | 0 | -3.523099 | 0.297736 | 0.285721 |
| 12 | 8 | 0 | 0.615658 | -0.372059 | 1.675814 |
| 13 | 1 | 0 | 0.623224 | 0.291817 | 2.391378 |
| 14 | 1 | 0 | 1.600183 | -0.756424 | 1.261105 |

$[CH_2O]_2SiCl(OH)$

HF=-1054.7318979
Thermal correction to Gibbs Free Energy= 0.046244
NImag=0

| | | | | | |
|---|---|---|---|---|---|
| 1 | 14 | 0 | -0.294771 | 0.357477 | -0.031878 |
| 2 | 8 | 0 | 0.744011 | -0.013241 | -1.262059 |
| 3 | 8 | 0 | 0.728754 | 0.132912 | 1.247174 |
| 4 | 17 | 0 | -1.885840 | -0.956245 | 0.103768 |
| 5 | 8 | 0 | -0.902811 | 1.866336 | -0.186878 |
| 6 | 1 | 0 | -1.861805 | 1.968040 | -0.122308 |
| 7 | 6 | 0 | 2.055590 | -0.106555 | 0.743224 |
| 8 | 1 | 0 | 2.614326 | 0.837039 | 0.756552 |
| 9 | 1 | 0 | 2.555898 | -0.825549 | 1.397515 |
| 10 | 6 | 0 | 1.907632 | -0.641892 | -0.696419 |
| 11 | 1 | 0 | 1.765215 | -1.729701 | -0.699508 |
| 12 | 1 | 0 | 2.773492 | -0.395709 | -1.316734 |

$[CH_2O]_2SiCl(OH)$ to $[CH_2O]_2Si(OH)_2$ TS

HF=-1131.1452959
Thermal correction to Gibbs Free Energy= 0.068673
NImag=1
Frequencies -- -189.6398

| | | | | | |
|---|---|---|---|---|---|
| 1 | 14 | 0 | -0.062709 | -0.322960 | -0.147171 |
| 2 | 8 | 0 | -0.575751 | 1.233491 | -0.291797 |
| 3 | 8 | 0 | -1.526402 | -1.018715 | 0.239196 |
| 4 | 17 | 0 | 2.463509 | 0.640670 | -0.026017 |
| 5 | 8 | 0 | 0.558961 | -1.234357 | -1.348273 |
| 6 | 1 | 0 | 1.452464 | -0.937503 | -1.597267 |
| 7 | 6 | 0 | -2.572926 | -0.082538 | -0.042853 |
| 8 | 1 | 0 | -2.943210 | -0.248772 | -1.062652 |
| 9 | 1 | 0 | -3.396451 | -0.241838 | 0.659225 |
| 10 | 6 | 0 | -1.961043 | 1.324405 | 0.100024 |
| 11 | 1 | 0 | -2.004138 | 1.674275 | 1.137886 |
| 12 | 1 | 0 | -2.451424 | 2.058349 | -0.544308 |
| 13 | 8 | 0 | 0.700172 | -0.735226 | 1.467320 |
| 14 | 1 | 0 | 1.681515 | -0.417461 | 1.300094 |
| 15 | 1 | 0 | 0.607489 | -1.669740 | 1.735120 |

$[CH_2O]_2Si(OH)_2$

HF=-670.3596105
Thermal correction to Gibbs Free Energy= 0.058973
NImag=0

| | | | | | |
|---|---|---|---|---|---|
| 1 | 14 | 0 | 0.610310 | -0.000008 | -0.000021 |
| 2 | 8 | 0 | -0.462803 | -0.878679 | 0.906572 |
| 3 | 8 | 0 | -0.462820 | 0.878548 | -0.906702 |
| 4 | 8 | 0 | 1.546123 | -1.037266 | -0.874116 |
| 5 | 1 | 0 | 2.213829 | -0.625291 | -1.440173 |
| 6 | 8 | 0 | 1.545925 | 1.037381 | 0.874157 |
| 7 | 1 | 0 | 2.212948 | 0.625452 | 1.441054 |
| 8 | 6 | 0 | -1.775247 | 0.684234 | -0.357147 |
| 9 | 1 | 0 | -1.990626 | 1.494283 | 0.351471 |
| 10 | 1 | 0 | -2.509033 | 0.720411 | -1.167788 |
| 11 | 6 | 0 | -1.775271 | -0.684235 | 0.357151 |
| 12 | 1 | 0 | -1.990784 | -1.494265 | -0.351446 |
| 13 | 1 | 0 | -2.508981 | -0.720350 | 1.167864 |

**d. tetrachlorosilane**

$SiCl_4$

HF=-2130.4665922
Thermal correction to Gibbs Free Energy= -0.025732
NImag=0

| | | | | | |
|---|---|---|---|---|---|
| 1 | 14 | 0 | -0.000005 | 0.000075 | -0.000001 |
| 2 | 17 | 0 | -0.003935 | -1.186855 | -1.664245 |
| 3 | 17 | 0 | 1.683258 | 1.159942 | 0.005767 |
| 4 | 17 | 0 | -1.654683 | 1.200265 | -0.015092 |
| 5 | 17 | 0 | -0.024635 | -1.173414 | 1.673571 |

$SiCl_4$ to $SiCl_3(OH)$ TS

HF=-2206.8642365
Thermal correction to Gibbs Free Energy= -0.007501
NImag=1
Frequencies -- -415.7825

| | | | | | |
|---|---|---|---|---|---|
| 1 | 17 | 0 | 0.036314 | -1.717792 | -1.039395 |
| 2 | 17 | 0 | 0.059213 | 1.797232 | -0.943552 |

| | | | | | |
|---|---|---|---|---|---|
| 3 | 17 | 0 | 2.272203 | -0.045248 | 0.531969 |
| 4 | 14 | 0 | 0.231330 | 0.015789 | 0.030062 |
| 5 | 8 | 0 | -0.228225 | 0.025069 | 1.768232 |
| 6 | 1 | 0 | 0.317882 | -0.474693 | 2.407351 |
| 7 | 1 | 0 | -1.318640 | -0.110597 | 1.685383 |
| 8 | 17 | 0 | -2.391969 | -0.024563 | 0.353362 |

$SiCl_3(OH)$

HF=-1746.1009766
Thermal correction to Gibbs Free Energy= -0.013842
NImag=0

| | | | | | |
|---|---|---|---|---|---|
| 1 | 14 | 0 | -0.016938 | -0.000048 | 0.269229 |
| 2 | 17 | 0 | 0.977207 | 1.661665 | -0.415182 |
| 3 | 17 | 0 | -1.931228 | -0.003039 | -0.427155 |
| 4 | 17 | 0 | 0.982712 | -1.658338 | -0.415533 |
| 5 | 8 | 0 | -0.119992 | -0.000470 | 1.901467 |
| 6 | 1 | 0 | 0.709324 | -0.000474 | 2.402835 |

$SiCl_3(OH)$ to $SiCl_2(OH)_2$TS

HF=-1822.5078623
Thermal correction to Gibbs Free Energy= 0.008208
NImag=1
Frequencies -- -209.8226

| | | | | | |
|---|---|---|---|---|---|
| 1 | 17 | 0 | 2.281689 | -0.460400 | -0.095700 |
| 2 | 17 | 0 | -2.389483 | -0.302319 | -0.046539 |
| 3 | 8 | 0 | 0.010191 | -0.847455 | 1.629410 |
| 4 | 1 | 0 | -0.935396 | -0.899892 | 1.859562 |
| 5 | 14 | 0 | 0.249836 | -0.114610 | 0.196651 |
| 6 | 8 | 0 | -0.179507 | -1.048579 | -1.320623 |
| 7 | 1 | 0 | 0.335600 | -0.884533 | -2.135098 |
| 8 | 1 | 0 | -1.209998 | -0.937564 | -1.373835 |
| 9 | 17 | 0 | 0.088184 | 1.909472 | -0.067999 |

$SiCl_2(OH)_2$

HF=-1361.733971
Thermal correction to Gibbs Free Energy= -0.001124
NImag=0

| | | | | | |
|---|---|---|---|---|---|
| 1 | 14 | 0 | 0.000042 | 0.357332 | -0.008058 |
| 2 | 17 | 0 | 1.652812 | -0.865062 | -0.020343 |
| 3 | 17 | 0 | -1.652545 | -0.865464 | -0.021007 |
| 4 | 8 | 0 | -0.000701 | 1.340131 | 1.306135 |
| 5 | 1 | 0 | -0.002900 | 0.931518 | 2.183220 |
| 6 | 8 | 0 | 0.000666 | 1.312476 | -1.333097 |
| 7 | 1 | 0 | -0.001929 | 2.263923 | -1.151761 |

$SiCl_2(OH)_2$ to $SiCl(OH)_3$TS

HF=-1438.1494687
Thermal correction to Gibbs Free Energy= 0.023028
NImag=1
Frequencies -- -145.4944

| | | | | | |
|---|---|---|---|---|---|
| 1 | 17 | 0 | 2.272156 | 0.115499 | -0.018123 |
| 2 | 17 | 0 | -2.318430 | 0.085900 | -0.010331 |
| 3 | 8 | 0 | 0.045957 | -1.242430 | -1.238496 |
| 4 | 1 | 0 | -0.885893 | -1.429864 | -1.442102 |
| 5 | 14 | 0 | 0.214314 | -0.230493 | 0.030431 |
| 6 | 8 | 0 | -0.078294 | 1.568739 | -0.399751 |
| 7 | 1 | 0 | 0.534654 | 2.211620 | 0.007465 |
| 8 | 1 | 0 | -1.048187 | 1.744348 | -0.232793 |
| 9 | 8 | 0 | 0.041876 | -0.578505 | 1.620626 |
| 10 | 1 | 0 | -0.890615 | -0.705422 | 1.866086 |

$SiCl(OH)_3$

HF=-977.366064
Thermal correction to Gibbs Free Energy= 0.011647
NImag=0

| | | | | | |
|---|---|---|---|---|---|
| 1 | 14 | 0 | -0.350156 | 0.012021 | -0.024510 |
| 2 | 17 | 0 | 1.708722 | 0.014335 | 0.123412 |
| 3 | 8 | 0 | -0.798804 | -1.302260 | -0.910594 |
| 4 | 1 | 0 | -0.253752 | -1.503311 | -1.684438 |
| 5 | 8 | 0 | -1.105015 | -0.120713 | 1.425913 |
| 6 | 1 | 0 | -1.224469 | -1.025863 | 1.747104 |
| 7 | 8 | 0 | -0.724534 | 1.469093 | -0.675796 |
| 8 | 1 | 0 | -1.641058 | 1.748224 | -0.533719 |

$SiCl(OH)_3$ to $Si(OH)_4$TS

HF=-1053.7820771

Thermal correction to Gibbs Free Energy= 0.036029
NImag=1
Frequencies -- -128.5474

| | | | | | |
|---|---|---|---|---|---|
| 1 | 8 | 0 | 0.345356 | -0.192231 | 1.711552 |
| 2 | 1 | 0 | -0.581877 | -0.292466 | 1.979430 |
| 3 | 8 | 0 | 0.429053 | -1.453277 | -0.941957 |
| 4 | 1 | 0 | -0.482285 | -1.740200 | -1.109941 |
| 5 | 8 | 0 | 2.133633 | 0.219860 | -0.060213 |
| 6 | 1 | 0 | 2.619043 | -0.404083 | -0.616402 |
| 7 | 14 | 0 | 0.520104 | -0.169121 | 0.080549 |
| 8 | 8 | 0 | 0.178309 | 1.543306 | -0.642744 |
| 9 | 1 | 0 | 0.871005 | 2.186941 | -0.399674 |
| 10 | 1 | 0 | -0.745338 | 1.830094 | -0.450415 |
| 11 | 17 | 0 | -1.979578 | -0.009051 | -0.062575 |

$Si(OH)_4$

HF=-592.9959338
Thermal correction to Gibbs Free Energy= 0.025173
NImag=0

| | | | | | |
|---|---|---|---|---|---|
| 1 | 14 | 0 | -0.000025 | 0.000003 | -0.000019 |
| 2 | 8 | 0 | 1.155739 | -0.795337 | -0.866630 |
| 3 | 1 | 0 | 1.896571 | -0.236966 | -1.141150 |
| 4 | 8 | 0 | -0.795328 | -1.155965 | 0.866414 |
| 5 | 1 | 0 | -0.236761 | -1.896560 | 1.141198 |
| 6 | 8 | 0 | -1.156043 | 0.795546 | -0.866115 |
| 7 | 1 | 0 | -1.896507 | 0.237098 | -1.141453 |
| 8 | 8 | 0 | 0.795600 | 1.155755 | 0.866344 |
| 9 | 1 | 0 | 0.237308 | 1.896380 | 1.141572 |

Water

HF=-76.4273616
Thermal correction to Gibbs Free Energy= 0.002654
NImag=0

| | | | | | |
|---|---|---|---|---|---|
| 1 | 8 | 0 | 0.000000 | 0.000000 | 0.119721 |
| 2 | 1 | 0 | 0.000000 | 0.761555 | -0.478885 |
| 3 | 1 | 0 | 0.000000 | -0.761555 | -0.478885 |

Hydrogen chloride

HF=-460.7992656
Thermal correction to Gibbs Free Energy= -0.011484
NImag=0

| | | | | | |
|---|---|---|---|---|---|
| 1 | 17 | 0 | 0.000000 | 0.000000 | 0.071638 |
| 2 | 1 | 0 | 0.000000 | 0.000000 | -1.217838 |

### e. NBO geometries

$CH_3OH$ $H_2O$

| | | | |
|---|---|---|---|
| C | 1.63812900 | -0.33878900 | -0.01116500 |
| H | 2.50727300 | 0.07150500 | 0.51205400 |
| H | 1.94620800 | -0.58470000 | -1.03985200 |
| H | 1.34978600 | -1.27629400 | 0.49104700 |
| O | 0.61811400 | 0.64154400 | 0.03194700 |
| H | -0.17968800 | 0.25926400 | -0.37862900 |
| O | -2.01803800 | -0.25629500 | -0.04061400 |
| H | -1.71386500 | -0.03756500 | 0.85549100 |
| H | -2.53909300 | 0.51853200 | -0.30378900 |

$SiH_3OH$ $H_2O$

| | | | |
|---|---|---|---|
| Si | 1.16559500 | -0.33062100 | -0.00006600 |
| H | 2.60169000 | 0.03811100 | 0.00514200 |
| H | 0.89548000 | -1.18854400 | -1.19669500 |
| H | 0.88789200 | -1.19275800 | 1.19189000 |
| O | 0.28859300 | 1.07014300 | -0.00003100 |
| H | -0.67534800 | 0.89817900 | -0.00134400 |
| O | -2.23386400 | -0.13036300 | 0.00005800 |
| H | -2.22963600 | -0.72123900 | 0.76911800 |
| H | -2.23624100 | -0.72329800 | -0.76740100 |

$Si(OH)_4$ $H_2O$

| | | | |
|---|---|---|---|
| Si | -0.55228900 | -0.02310400 | -0.00964900 |
| O | 0.35927500 | -1.20388000 | -0.66792000 |
| H | 1.32761500 | -1.01800000 | -0.61479300 |
| O | 0.39426300 | 0.69659700 | 1.16607900 |
| H | 0.06518700 | 1.56065200 | 1.45324100 |
| O | -0.98955000 | 1.23947800 | -0.97982700 |
| H | -1.75718100 | 1.06558600 | -1.54226600 |

| | | | |
|---|---|---|---|
| O | -1.95287500 | -0.69736800 | 0.54038200 |
| H | -1.85493900 | -1.62663000 | 0.79266800 |
| O | 2.78355500 | -0.10731100 | -0.04564800 |
| H | 2.21759100 | 0.32584900 | 0.62641300 |
| H | 2.97642000 | 0.59587900 | -0.68470300 |

$SiCl(OH)_3$ $H_2O$ double bonding

| | | | |
|---|---|---|---|
| Si | 0.51626500 | -0.39184200 | 0.00016600 |
| O | -0.17522200 | -0.96827000 | -1.36817100 |
| H | -1.14693600 | -0.92331400 | -1.31966900 |
| O | -0.17649100 | -0.96489600 | 1.36943700 |
| H | -1.14801300 | -0.91507200 | 1.32180000 |
| O | 2.11708300 | -0.70793300 | -0.00070700 |
| H | 2.70044500 | 0.06180600 | 0.01344000 |
| Cl | 0.27029500 | 1.70706600 | -0.00131000 |
| O | -2.55597300 | -0.14916900 | 0.00026900 |
| H | -3.52054500 | -0.24330200 | -0.00014600 |
| H | -2.38284100 | 0.80770700 | -0.00210800 |

$SiCl(OH)_3$ $H_2O$ single bonding

| | | | |
|---|---|---|---|
| Si | 0.36978900 | -0.38261000 | -0.02481900 |
| O | -0.55531200 | -0.66605900 | -1.33631900 |
| H | -1.48168300 | -0.35524700 | -1.19039200 |
| O | -0.47344800 | -0.56322900 | 1.39788700 |
| H | -0.27219100 | -1.39458800 | 1.85349500 |
| O | 1.63830600 | -1.42495200 | -0.00007300 |
| H | 1.83309700 | -1.83853500 | -0.85353900 |
| Cl | 1.01552100 | 1.58002100 | -0.00638700 |
| O | -2.78545900 | 0.26501300 | -0.07213600 |
| H | -2.77688400 | 1.23295600 | -0.01039000 |
| H | -2.33594300 | -0.03459200 | 0.74198500 |

$(CH_2)_2O_2SiClOH$ $H_2O$

| | | | |
|---|---|---|---|
| Si | 0.09892300 | -0.22073700 | 0.00742600 |
| O | 1.17072600 | -0.16711700 | 1.26681400 |
| O | 1.18010000 | -0.20895800 | -1.24619400 |
| Cl | -1.02949500 | 1.54150200 | -0.09064700 |
| O | -0.91826400 | -1.47065000 | 0.08759400 |
| H | -1.89059500 | -1.30849400 | -0.00591300 |

| | | | |
|---|---|---|---|
| C | 2.50981300 | -0.33270400 | -0.71358700 |
| H | 2.81173600 | -1.38633500 | -0.75598300 |
| H | 3.19352800 | 0.25801500 | -1.32952700 |
| C | 2.46664700 | 0.17009000 | 0.74455900 |
| H | 2.60117300 | 1.25831900 | 0.78968900 |
| H | 3.22938900 | -0.30692300 | 1.36600900 |
| O | -3.50521800 | -0.68652200 | -0.03450900 |
| H | -3.31948500 | 0.23229500 | -0.29442500 |
| H | -3.78675500 | -0.62044500 | 0.89170500 |

$(CH_2)_2O_2Si(OH)_2$ $H_2O$

| | | | |
|---|---|---|---|
| Si | 0.09892300 | -0.22073700 | 0.00742600 |
| O | 1.17072600 | -0.16711700 | 1.26681400 |
| O | 1.18010000 | -0.20895800 | -1.24619400 |
| Cl | -1.02949500 | 1.54150200 | -0.09064700 |
| O | -0.91826400 | -1.47065000 | 0.08759400 |
| H | -1.89059500 | -1.30849400 | -0.00591300 |
| C | 2.50981300 | -0.33270400 | -0.71358700 |
| H | 2.81173600 | -1.38633500 | -0.75598300 |
| H | 3.19352800 | 0.25801500 | -1.32952700 |
| C | 2.46664700 | 0.17009000 | 0.74455900 |
| H | 2.60117300 | 1.25831900 | 0.78968900 |
| H | 3.22938900 | -0.30692300 | 1.36600900 |
| O | -3.50521800 | -0.68652200 | -0.03450900 |
| H | -3.31948500 | 0.23229500 | -0.29442500 |
| H | -3.78675500 | -0.62044500 | 0.89170500 |

$(CH_2)_2O_{2C}(OH)_2$ $H_2O$

| | | | |
|---|---|---|---|
| O | 0.02556300 | 0.85369200 | 0.39615000 |
| O | 1.29685200 | -0.98196100 | -0.01937600 |
| O | -0.48998400 | -0.61313700 | -1.32699900 |
| H | -1.42162000 | -0.32466200 | -1.30848100 |
| O | -0.75363900 | -1.24373500 | 0.88716800 |
| H | -1.66822600 | -0.90404700 | 0.81957100 |
| C | 2.18120800 | 0.12914000 | 0.14163500 |
| H | 2.60146700 | 0.11313500 | 1.15454800 |
| H | 2.99001500 | 0.05172700 | -0.59004600 |
| C | 1.27719500 | 1.34510400 | -0.07632200 |
| H | 1.21019300 | 1.61562200 | -1.13760000 |
| H | 1.55678000 | 2.21870000 | 0.51659500 |
| O | -2.65558100 | 0.64500900 | 0.02204100 |
| H | -3.44581900 | 1.09606600 | 0.35146600 |
| H | -1.88753300 | 1.20417200 | 0.25612100 |
| C | -0.01189300 | -0.53251900 | -0.02098600 |

BIFOXSiCl(OH) $H_2O$

| | | | |
|---|---|---|---|
| H | 5.48397600 | 2.39343400 | -0.21359400 |
| C | 4.55104600 | 1.94275900 | 0.14292900 |
| H | 4.33409300 | 2.38485100 | 1.11726500 |
| C | 3.40349600 | 2.13899300 | -0.88192900 |
| C | 4.69241500 | 0.41312100 | 0.18409900 |
| H | 3.74625500 | 2.69963700 | -1.75803900 |
| H | 2.55531700 | 2.67697100 | -0.46982300 |
| C | 3.02855000 | 0.69763900 | -1.35332600 |
| C | 4.44398000 | 0.06412200 | -1.29365300 |
| H | 5.64574300 | 0.07009200 | 0.59857500 |
| C | 3.48219200 | -0.25931300 | 0.90156000 |
| Cl | -0.01272900 | 3.18169600 | -1.10125900 |
| C | 2.27377400 | -0.02072900 | -0.15867500 |
| C | 2.29389000 | 0.74674400 | -2.69165800 |
| H | 4.50098000 | -1.00297500 | -1.50423000 |
| H | 5.13618800 | 0.57720700 | -1.97155200 |
| C | 3.82767500 | -1.74165800 | 1.13881600 |
| C | 3.16327800 | 0.35265300 | 2.27198200 |
| Si | -0.10893100 | 1.48588500 | 0.17238500 |
| H | 1.35481800 | 1.29998200 | -2.59892000 |
| O | 1.36841800 | 0.89213700 | 0.44359800 |
| C | 1.56626900 | -1.34994100 | -0.48274000 |
| H | 2.90955100 | 1.27573100 | -3.42840300 |
| H | 2.04938000 | -0.23377800 | -3.10151000 |
| H | 4.65142200 | -1.79817200 | 1.85952900 |
| H | 4.14339900 | -2.26261700 | 0.23228800 |
| H | 2.98169200 | -2.29685200 | 1.55229500 |
| H | 2.33482800 | -0.17703200 | 2.74885200 |
| H | 2.87199100 | 1.40024400 | 2.21341100 |
| H | 4.04367000 | 0.26889300 | 2.92090800 |
| O | -1.03953700 | 0.44628000 | -0.68514500 |
| O | -0.62893400 | 2.02190800 | 1.61205400 |
| C | 0.52694800 | -1.91991900 | 0.29763800 |
| C | 2.12859600 | -2.16477800 | -1.48180400 |
| C | -2.17205900 | -0.27456000 | -0.14958900 |
| H | -1.02313800 | 2.92437900 | 1.65745200 |
| C | -0.35634400 | -1.34305700 | 1.38786900 |
| C | 0.27642900 | -3.30041500 | 0.15509500 |
| H | 2.89734100 | -1.75926900 | -2.11776200 |
| C | 1.78780300 | -3.49842100 | -1.67555200 |
| C | -3.42398000 | 0.71785100 | -0.12003100 |
| C | -2.67222100 | -1.38262000 | -1.20920500 |
| C | -1.68549400 | -0.85320200 | 1.19573400 |
| C | 0.05552000 | -1.66221800 | 2.69077200 |
| C | 0.88122200 | -4.09509300 | -0.80974900 |
| H | -0.42959300 | -3.75334400 | 0.84222700 |
| H | 2.26428300 | -4.06592700 | -2.46969500 |

| | | | |
|---|---|---|---|
| C | -3.45499900 | 1.46453400 | -1.49301500 |
| C | -3.56996500 | 1.75646800 | 0.99947600 |
| C | -4.60789800 | -0.25070800 | -0.33652300 |
| C | -4.06773300 | -0.84375500 | -1.64729100 |
| C | -2.92469800 | -2.76037800 | -0.56973700 |
| C | -1.69713500 | -1.56641900 | -2.37827200 |
| C | -2.54135000 | -0.85931000 | 2.30369800 |
| C | -0.79048200 | -1.56359500 | 3.79125400 |
| H | 1.05997200 | -2.05019700 | 2.82619700 |
| H | 0.63639600 | -5.15049800 | -0.88292100 |
| H | -4.16987600 | 2.29006500 | -1.40663800 |
| H | -2.48692400 | 1.89578900 | -1.74272500 |
| C | -3.93928400 | 0.41274800 | -2.51956100 |
| H | -4.62975100 | 2.02275500 | 1.09312400 |
| H | -3.04010300 | 2.67865800 | 0.75037200 |
| H | -3.21027900 | 1.43702500 | 1.97510900 |
| H | -4.75543600 | -0.98354500 | 0.45999700 |
| H | -5.54836800 | 0.29648100 | -0.47007100 |
| H | -4.67385000 | -1.63423700 | -2.10037100 |
| H | -3.39240400 | -3.41321900 | -1.31596700 |
| H | -3.58910000 | -2.71500800 | 0.29632500 |
| H | -2.00054000 | -3.23551600 | -0.25550300 |
| H | -0.78220300 | -2.06441600 | -2.05389900 |
| H | -1.40772500 | -0.61826900 | -2.83188700 |
| H | -2.16780400 | -2.19138400 | -3.14739000 |
| H | -3.57678600 | -0.57919500 | 2.16987700 |
| C | -2.11630800 | -1.20221300 | 3.58472500 |
| H | -0.42666400 | -1.81370800 | 4.78358200 |
| H | -4.91510300 | 0.68265200 | -2.93868700 |
| H | -3.25336500 | 0.28314800 | -3.35903400 |
| H | -2.82022800 | -1.17232300 | 4.41147000 |
| O | -1.42916200 | 4.63294600 | 1.46866500 |
| H | -1.17889300 | 4.69098700 | 0.52935600 |
| H | -0.72681400 | 5.11311600 | 1.93502300 |

BIFOXSi(OH)$_2$ $H_2O$

| | | | |
|---|---|---|---|
| Si | -0.08542700 | 1.62324700 | 0.26443800 |
| O | -1.06787000 | 0.59379300 | -0.57164200 |
| O | 1.31921200 | 0.88929000 | 0.62173100 |
| O | 0.11054300 | 2.93530700 | -0.72631000 |
| O | -0.65396100 | 2.12187400 | 1.72931600 |
| C | -2.13872800 | -0.22045600 | -0.09869000 |
| C | 2.27758700 | 0.15081700 | -0.11547200 |
| H | -0.45900500 | 3.68547200 | -0.48066600 |
| C | -3.41587900 | 0.72249200 | 0.19110400 |
| C | -2.67868500 | -1.11674300 | -1.32651100 |
| C | -1.59669000 | -1.01846200 | 1.10366900 |
| C | 3.01454500 | 1.09303000 | -1.16181500 |

| | | | |
|---|---|---|---|
| C | 3.48760100 | -0.21930200 | 0.90377700 |
| C | 1.63160400 | -1.12670900 | -0.68330400 |
| C | -3.45654400 | 1.76582700 | -0.96939800 |
| C | -4.59973100 | -0.16703500 | -0.24573700 |
| C | -3.57383900 | 1.38753500 | 1.57358600 |
| C | -4.05553700 | -0.46506000 | -1.64658900 |
| C | -2.95254000 | -2.57985400 | -0.93121400 |
| C | -1.72919300 | -1.11952200 | -2.53077800 |
| C | -0.26030900 | -1.51286000 | 1.16476700 |
| C | -2.41907900 | -1.24664100 | 2.21442700 |
| C | 3.32165800 | 2.44809200 | -0.45056000 |
| C | 2.30267500 | 1.34765000 | -2.49076500 |
| C | 4.45411400 | 0.51507700 | -1.18431900 |
| C | 3.13573700 | 0.13125900 | 2.35491000 |
| C | 3.89789200 | -1.70400100 | 0.87946100 |
| C | 4.67223100 | 0.61898300 | 0.33463500 |
| C | 0.59936000 | -1.85045700 | -0.03507700 |
| C | 2.21695800 | -1.73028200 | -1.81089400 |
| C | -3.89489200 | 0.95602200 | -2.21761600 |
| H | -4.21454800 | 2.52081000 | -0.71812300 |
| H | -2.49720200 | 2.25981300 | -1.12597200 |
| H | -4.74313200 | -1.05035300 | 0.37961800 |
| H | -5.54082900 | 0.39676100 | -0.25468700 |
| H | -4.42383400 | 0.96671800 | 2.12124000 |
| H | -3.77597000 | 2.46014100 | 1.46886800 |
| H | -2.68765900 | 1.28587400 | 2.19597100 |
| H | -4.67097300 | -1.11666200 | -2.27447900 |
| H | -3.48728600 | -3.07599500 | -1.74997400 |
| H | -3.55759100 | -2.68032000 | -0.02716700 |
| H | -2.02655900 | -3.12542900 | -0.76796300 |
| H | -2.23634600 | -1.58096400 | -3.38775900 |
| H | -0.82807400 | -1.69739900 | -2.32250800 |
| H | -1.41056400 | -0.11637200 | -2.81176400 |
| C | 0.19055700 | -2.07439700 | 2.36899900 |
| C | -1.95737000 | -1.82504800 | 3.39412000 |
| H | -3.45911100 | -0.95765900 | 2.16577900 |
| H | 3.66243200 | 3.15402600 | -1.21615300 |
| H | 2.43939300 | 2.88263100 | 0.01065600 |
| C | 4.45752000 | 2.12612900 | 0.55481600 |
| H | 2.96809100 | 1.91452500 | -3.15337600 |
| H | 1.40712100 | 1.95400000 | -2.33243400 |
| H | 2.00235300 | 0.44040600 | -3.01658300 |
| H | 4.55862700 | -0.49814000 | -1.57128400 |
| H | 5.12993700 | 1.16335000 | -1.75481100 |
| H | 2.34521400 | -0.52230000 | 2.73135500 |
| H | 2.77627900 | 1.15277300 | 2.46732000 |
| H | 4.02277400 | -0.00370700 | 2.98641300 |
| H | 4.72079700 | -1.85350300 | 1.58814900 |
| H | 4.23941900 | -2.04021200 | -0.10220300 |
| H | 3.07598500 | -2.36071400 | 1.17618700 |
| H | 5.63630200 | 0.25291900 | 0.70279700 |

| | | | |
|---|---|---|---|
| C | 0.35094700 | -3.17405600 | -0.45250400 |
| C | 1.88930300 | -3.00321300 | -2.26384300 |
| H | 2.98852500 | -1.20342900 | -2.34786700 |
| H | -4.85360600 | 1.31663600 | -2.60649800 |
| H | -3.17216000 | 1.01257400 | -3.03326200 |
| H | 1.20225100 | -2.46540200 | 2.40122700 |
| C | -0.62437600 | -2.20507600 | 3.48960800 |
| H | -2.63819500 | -1.96407800 | 4.22912500 |
| H | 4.20082400 | 2.37865400 | 1.58545700 |
| H | 5.37492600 | 2.67279200 | 0.30838100 |
| H | -0.35819300 | -3.75637900 | 0.12532100 |
| C | 0.96848800 | -3.75905000 | -1.55002100 |
| H | 2.38296800 | -3.40429000 | -3.14446500 |
| H | -0.22997400 | -2.64009700 | 4.40344600 |
| H | 0.72755400 | -4.77855900 | -1.83644400 |
| H | -1.17635800 | 2.94034900 | 1.66546300 |
| O | -1.99470300 | 4.36540300 | 0.52235100 |
| H | -2.25299400 | 5.26734600 | 0.76548700 |
| H | -2.76371100 | 3.97448000 | 0.07585000 |

BIFOXSiCl(OH) $Cl^-$

1 1

| | | | |
|---|---|---|---|
| H | 5.31260800 | 2.56961700 | -0.74039100 |
| C | 4.43185900 | 2.11936800 | -0.27340700 |
| H | 4.24629300 | 2.66698400 | 0.65170200 |
| C | 3.21813600 | 2.13044800 | -1.23782300 |
| C | 4.66764600 | 0.61590100 | -0.06070600 |
| H | 3.47809200 | 2.58882600 | -2.19723200 |
| H | 2.36903000 | 2.68250800 | -0.84679800 |
| C | 2.90485000 | 0.62947000 | -1.51441100 |
| C | 4.34655700 | 0.06477500 | -1.45756800 |
| H | 5.66485600 | 0.37355900 | 0.31860500 |
| C | 3.57206500 | -0.02864800 | 0.83260500 |
| Cl | -0.26191000 | 2.98950300 | -1.27790000 |
| C | 2.23745400 | 0.02564100 | -0.17209900 |
| C | 2.07431300 | 0.45475600 | -2.77949400 |
| H | 4.45386900 | -1.01616500 | -1.54135700 |
| H | 4.96089900 | 0.51708000 | -2.24357500 |
| C | 4.02036600 | -1.44926300 | 1.21638500 |
| C | 3.28135100 | 0.73057200 | 2.13032900 |
| Si | -0.18216400 | 1.40939700 | 0.02922800 |
| H | 1.11956900 | 0.98485300 | -2.70521800 |
| O | 1.34310400 | 0.94949300 | 0.37060100 |
| C | 1.61496800 | -1.34797900 | -0.27075200 |
| H | 2.61418500 | 0.89640700 | -3.62399400 |
| H | 1.85275600 | -0.58375200 | -3.03132300 |
| H | 4.88015400 | -1.36229500 | 1.88836200 |
| H | 4.33681700 | -2.05291500 | 0.36401400 |
| H | 3.24110800 | -2.00092900 | 1.75072600 |

| | | | |
|---|---|---|---|
| H | 2.55160800 | 0.19558000 | 2.74548200 |
| H | 2.89496400 | 1.73430000 | 1.96639000 |
| H | 4.21171700 | 0.80472400 | 2.70483300 |
| O | -0.87227800 | 0.11376400 | -0.77078300 |
| O | -0.88162300 | 1.79437400 | 1.43414200 |
| C | 0.67730900 | -1.85054200 | 0.71220700 |
| C | 2.21094800 | -2.29860000 | -1.12652600 |
| C | -2.00907900 | -0.54858500 | -0.23651700 |
| H | -1.23746400 | 2.72306800 | 1.51877800 |
| C | -0.29454900 | -1.16435600 | 1.60485500 |
| C | 0.68026900 | -3.24522900 | 0.96052200 |
| H | 2.85107700 | -1.95521300 | -1.92163400 |
| C | 2.06827200 | -3.66245600 | -0.95451100 |
| C | -3.34325400 | 0.33949300 | -0.55904200 |
| C | -2.36586000 | -1.92035900 | -1.08648200 |
| C | -1.66129900 | -0.81342600 | 1.20309500 |
| C | -0.01120400 | -1.26228600 | 2.98259300 |
| C | 1.33945100 | -4.14647300 | 0.14209800 |
| H | 0.06338700 | -3.62181000 | 1.76848500 |
| H | 2.55853700 | -4.34852300 | -1.63739200 |
| C | -3.24325700 | 0.75075400 | -2.05881700 |
| C | -3.64420300 | 1.56506100 | 0.29672700 |
| C | -4.42692100 | -0.75821600 | -0.64505000 |
| C | -3.73083100 | -1.57393700 | -1.73862400 |
| C | -2.57688000 | -3.13994900 | -0.17296900 |
| C | -1.26819200 | -2.24292600 | -2.09841600 |
| C | -2.63779500 | -0.74820400 | 2.21390700 |
| C | -0.97550200 | -1.04204100 | 3.94683500 |
| H | 0.99835800 | -1.52324300 | 3.27974900 |
| H | 1.25345800 | -5.21205800 | 0.32745400 |
| H | -3.98671500 | 1.53418400 | -2.23398300 |
| H | -2.27182900 | 1.17465800 | -2.30657400 |
| C | -3.57959900 | -0.53292000 | -2.85681700 |
| H | -4.67894400 | 1.86170500 | 0.08795300 |
| H | -3.02625800 | 2.42408900 | 0.02662800 |
| H | -3.55037200 | 1.41883600 | 1.37151300 |
| H | -4.61318300 | -1.31133100 | 0.27762100 |
| H | -5.37831800 | -0.32452100 | -0.97062100 |
| H | -4.24872100 | -2.48618500 | -2.04931900 |
| H | -2.85897000 | -3.99317400 | -0.79868900 |
| H | -3.37336900 | -2.99442600 | 0.56022600 |
| H | -1.66953800 | -3.41707000 | 0.36235800 |
| H | -0.39102200 | -2.67415100 | -1.61820600 |
| H | -0.94509800 | -1.37221700 | -2.66875900 |
| H | -1.65621000 | -2.99007300 | -2.80157500 |
| H | -3.66479700 | -0.56780800 | 1.93571300 |
| C | -2.31522900 | -0.83387900 | 3.55322300 |
| H | -0.71331200 | -1.06853100 | 4.99938700 |
| H | -4.52462100 | -0.42839500 | -3.39775200 |
| H | -2.81799900 | -0.79512600 | -3.59315200 |
| H | -3.09040900 | -0.72394000 | 4.30453700 |

| | | | |
|---|---|---|---|
| Cl | -1.67647300 | 4.66840800 | 1.16402100 |

BIFOXSi(OH)$_2$ Cl$^-$

1 1

| | | | |
|---|---|---|---|
| Si | -0.11476200 | 1.51431600 | -0.08363700 |
| O | -0.84288200 | 0.16904200 | -0.80570900 |
| O | 1.35376700 | 0.95037600 | 0.38284500 |
| O | -0.10121400 | 2.67695700 | -1.21397700 |
| O | -0.84843100 | 1.89775500 | 1.32702700 |
| C | -2.01309100 | -0.36443000 | -0.24823300 |
| C | 2.18868100 | -0.00178600 | -0.19027000 |
| H | -0.31941000 | 3.57858000 | -0.87509200 |
| C | -3.29531500 | 0.59402200 | -0.66390900 |
| C | -2.47164100 | -1.77453000 | -0.99968000 |
| C | -1.70885700 | -0.54841200 | 1.20377400 |
| C | 2.80376100 | 0.54288300 | -1.58994000 |
| C | 3.56736700 | -0.09017100 | 0.73807800 |
| C | 1.50210400 | -1.34621500 | -0.23144500 |
| C | -3.12042300 | 0.89873900 | -2.17900200 |
| C | -4.44659800 | -0.43009000 | -0.71039800 |
| C | -3.53089000 | 1.88503900 | 0.10941400 |
| C | -3.78021500 | -1.36660700 | -1.72167900 |
| C | -2.79550500 | -2.89703400 | 0.00126400 |
| C | -1.38043800 | -2.26242000 | -1.95030000 |
| C | -0.36574600 | -0.96946700 | 1.64577700 |
| C | -2.70401000 | -0.39554400 | 2.19507100 |
| C | 3.16814000 | 2.04083000 | -1.37348300 |
| C | 1.90773100 | 0.35700300 | -2.80878100 |
| C | 4.22794800 | -0.06041800 | -1.58447300 |
| C | 3.36776600 | 0.68837800 | 2.04236200 |
| C | 3.99501200 | -1.51819800 | 1.11781400 |
| C | 4.63070700 | 0.51538700 | -0.21782900 |
| C | 0.56707100 | -1.74883100 | 0.80159700 |
| C | 2.02949300 | -2.36397700 | -1.05319800 |
| C | -3.50762600 | -0.41681100 | -2.89952500 |
| H | -3.81857500 | 1.70387000 | -2.42864900 |
| H | -2.12022500 | 1.25998400 | -2.41277800 |
| H | -4.70122100 | -0.90090200 | 0.24112700 |
| H | -5.35450200 | 0.04515700 | -1.09732600 |
| H | -4.52931600 | 2.24433100 | -0.16811400 |
| H | -2.83317100 | 2.67399100 | -0.17771600 |
| H | -3.50512400 | 1.79274400 | 1.19454400 |
| H | -4.35668400 | -2.25555900 | -1.99521400 |
| H | -3.12513800 | -3.77522300 | -0.56380800 |
| H | -3.59508200 | -2.63782200 | 0.69811700 |
| H | -1.92208200 | -3.19480800 | 0.58282800 |
| H | -1.82601800 | -2.99921000 | -2.63002500 |
| H | -0.57648500 | -2.76199400 | -1.41394700 |
| H | -0.94570800 | -1.46241600 | -2.54877700 |

| | | | |
|---|---|---|---|
| C | -0.11824500 | -1.03278000 | 3.03520800 |
| C | -2.41116800 | -0.45457700 | 3.53764700 |
| H | -3.71103500 | -0.15962000 | 1.88595900 |
| H | 3.41701500 | 2.45044400 | -2.35809300 |
| H | 2.34143100 | 2.63172400 | -0.98987900 |
| C | 4.41135600 | 2.02088900 | -0.44768800 |
| H | 2.45110800 | 0.69420900 | -3.69843100 |
| H | 1.00788000 | 0.97435200 | -2.73506200 |
| H | 1.59509600 | -0.67462000 | -2.98318200 |
| H | 4.29908400 | -1.14632800 | -1.64372800 |
| H | 4.81634800 | 0.35190000 | -2.41140500 |
| H | 2.66897600 | 0.17013900 | 2.70696900 |
| H | 2.98639700 | 1.69551500 | 1.88942800 |
| H | 4.33207700 | 0.75330400 | 2.55894300 |
| H | 4.88723800 | -1.44786300 | 1.74811000 |
| H | 4.25549300 | -2.14140700 | 0.26075100 |
| H | 3.22620100 | -2.04005100 | 1.69650400 |
| H | 5.64094800 | 0.26295900 | 0.11790700 |
| C | 0.52765900 | -3.12696900 | 1.13984900 |
| C | 1.84232100 | -3.70704600 | -0.78965300 |
| H | 2.65753500 | -2.08865100 | -1.88508300 |
| H | -4.41862200 | -0.29074500 | -3.49172300 |
| H | -2.73435400 | -0.77956000 | -3.57859400 |
| H | 0.87103000 | -1.33063200 | 3.36374000 |
| C | -1.08827500 | -0.72608000 | 3.96539600 |
| H | -3.18983600 | -0.27444600 | 4.27155800 |
| H | 4.26392100 | 2.58346300 | 0.47548600 |
| H | 5.28430600 | 2.45041400 | -0.94768600 |
| H | -0.08711100 | -3.43311500 | 1.97864300 |
| C | 1.12932900 | -4.09594000 | 0.35733600 |
| H | 2.28619000 | -4.45389100 | -1.43979800 |
| H | -0.84978100 | -0.72761900 | 5.02400100 |
| H | 1.00788300 | -5.14521000 | 0.60538200 |
| H | -1.17677300 | 2.83991600 | 1.38833000 |
| Cl | -1.07373800 | 4.77307100 | 0.82638900 |

Dimer

0 1

| | | | |
|---|---|---|---|
| Si | 1.98349600 | -0.26048100 | 0.01108200 |
| O | 2.21893000 | 1.34660600 | 0.14007800 |
| O | 3.37177000 | -1.06096100 | 0.37132400 |
| O | 1.41412600 | -0.41295000 | -1.55146200 |
| O | 0.85531800 | -0.91575200 | 1.00139500 |
| C | 2.98907300 | 2.17168300 | 0.99313800 |
| C | 4.18378300 | -1.76084400 | -0.58386700 |
| H | 1.88350300 | 0.16234700 | -2.17628600 |
| H | -0.01310000 | -0.47756100 | 0.92574300 |
| C | 4.48429000 | 1.83411900 | 0.83757600 |
| C | 2.68195900 | 3.70403500 | 0.55500300 |

| | | | |
|---|---|---|---|
| C | 2.41666700 | 2.13370900 | 2.47295200 |
| C | 4.35085300 | -0.77256100 | -1.76126700 |
| C | 5.57270000 | -2.22753600 | 0.08664800 |
| C | 3.48940200 | -3.17117200 | -0.88387700 |
| C | 5.07194100 | 1.29381500 | -0.33445800 |
| C | 5.36545300 | 2.25467400 | 1.84994700 |
| C | 1.90825800 | 4.25215900 | 1.79254500 |
| C | 1.87209900 | 3.76703000 | -0.74720900 |
| C | 3.94143100 | 4.56318800 | 0.34044000 |
| C | 0.86114600 | 2.17178500 | 2.39170600 |
| C | 2.64328700 | 3.58673200 | 2.96997800 |
| C | 2.86883000 | 0.95747400 | 3.33491500 |
| C | 4.46682900 | 0.64054800 | -1.56085700 |
| C | 4.28008900 | -1.23161500 | -3.08203200 |
| C | 5.44023000 | -3.78016800 | 0.11747600 |
| C | 5.79434800 | -1.61237600 | 1.47290700 |
| C | 6.80112000 | -1.90993600 | -0.78537000 |
| C | 3.07506300 | -3.77041400 | 0.50040800 |
| C | 4.70318100 | -4.06920500 | -1.20117800 |
| C | 2.28060700 | -3.26472200 | -1.81772200 |
| C | 6.46863900 | 1.40949100 | -0.49107600 |
| C | 6.74863700 | 2.26657000 | 1.71458800 |
| H | 4.95854300 | 2.61743800 | 2.77965900 |
| H | 1.88914800 | 5.34671600 | 1.80271900 |
| C | 0.51600900 | 3.60754700 | 1.92047000 |
| H | 1.56558700 | 4.80303700 | -0.93618300 |
| H | 0.98493900 | 3.13664900 | -0.72760800 |
| H | 2.48147600 | 3.43621300 | -1.59396100 |
| H | 3.63428600 | 5.57157900 | 0.03957900 |
| H | 4.57684900 | 4.15449900 | -0.45109400 |
| H | 4.56059600 | 4.65854700 | 1.23498700 |
| H | 0.46830000 | 1.40318600 | 1.73902300 |
| H | 0.46730800 | 1.97875100 | 3.39557100 |
| H | 2.14319500 | 3.75653300 | 3.93089700 |
| H | 3.67796100 | 3.91042500 | 3.07462500 |
| H | 2.47284000 | 1.07068200 | 4.35095600 |
| H | 3.95264600 | 0.85163800 | 3.40324700 |
| H | 2.47114700 | 0.01935100 | 2.93358100 |
| C | 4.32972600 | 1.48720600 | -2.67157800 |
| C | 4.17104500 | -0.37310300 | -4.17504500 |
| H | 4.28304000 | -2.29685400 | -3.26517400 |
| H | 6.41696600 | -4.26003000 | 0.23447000 |
| C | 4.40059100 | -4.23399100 | 1.15224800 |
| H | 6.63135600 | -2.12192300 | 1.96632700 |
| H | 4.91249600 | -1.69711700 | 2.10806200 |
| H | 6.04305300 | -0.55279600 | 1.39918200 |
| H | 7.68593700 | -2.36754500 | -0.32751000 |
| H | 6.98422700 | -0.84078000 | -0.84995300 |
| H | 6.71664500 | -2.29770600 | -1.80375600 |
| H | 2.52146800 | -3.05141100 | 1.10215000 |
| H | 2.40947300 | -4.61963600 | 0.31348600 |

| | | | |
|---|---|---|---|
| H | 4.40803000 | -5.11988200 | -1.30468300 |
| H | 5.26305700 | -3.78659900 | -2.09604000 |
| H | 2.10197100 | -4.32415000 | -2.03826300 |
| H | 2.37826400 | -2.73554400 | -2.76408400 |
| H | 1.38269400 | -2.88365200 | -1.33021300 |
| H | 6.89462600 | 1.09806300 | -1.43886400 |
| C | 7.31228800 | 1.88656000 | 0.50339900 |
| H | 7.36756300 | 2.60635800 | 2.53996100 |
| H | -0.05320100 | 3.62268100 | 0.98938600 |
| H | -0.07820300 | 4.14121300 | 2.67033100 |
| H | 4.41800900 | 2.55681700 | -2.50877900 |
| C | 4.14162700 | 1.00173700 | -3.96502800 |
| H | 4.09049200 | -0.78375900 | -5.17722500 |
| H | 4.56634000 | -3.80582700 | 2.14316500 |
| H | 4.43132800 | -5.32388300 | 1.26293900 |
| H | 8.38363000 | 1.93962500 | 0.33465200 |
| H | 4.02618400 | 1.69178000 | -4.79568500 |
| O | -1.26939700 | -0.57503100 | -2.14086300 |
| H | -0.29267300 | -0.61210300 | -2.02304900 |
| Si | -2.07718900 | -0.12207600 | -0.79816300 |
| O | -2.25268000 | -1.26340200 | 0.36631200 |
| O | -3.55173700 | 0.41020000 | -1.29025500 |
| O | -1.09060000 | 0.95748000 | -0.00047100 |
| C | -3.14774800 | -2.35430800 | 0.51144200 |
| C | -4.09708800 | 1.69204700 | -0.94285200 |
| H | -1.51692000 | 1.43935700 | 0.72512300 |
| C | -4.58626300 | -1.81622000 | 0.61867100 |
| C | -2.73395300 | -3.15219800 | 1.86408000 |
| C | -2.87068600 | -3.44154300 | -0.60868900 |
| C | -3.91968000 | 1.79613300 | 0.59102600 |
| C | -5.62841300 | 1.80177200 | -1.43276700 |
| C | -3.39552000 | 2.80499500 | -1.86259600 |
| C | -4.92308100 | -0.55090700 | 1.16534500 |
| C | -5.64947700 | -2.69592500 | 0.35364800 |
| C | -2.19624400 | -4.50085300 | 1.29690900 |
| C | -1.69970600 | -2.38601800 | 2.69748800 |
| C | -3.91908200 | -3.45494100 | 2.80158000 |
| C | -1.32456800 | -3.59238900 | -0.75338400 |
| C | -3.15751000 | -4.77630100 | 0.12713600 |
| C | -3.51395800 | -3.19044900 | -1.97098900 |
| C | -4.07269200 | 0.66802500 | 1.46034000 |
| C | -3.50399600 | 3.00371500 | 1.16923000 |
| C | -5.56108700 | 2.87469900 | -2.56004700 |
| C | -6.19183400 | 0.45937000 | -1.91321200 |
| C | -6.56857600 | 2.36424400 | -0.35065600 |
| C | -3.38010600 | 2.23150000 | -3.31803600 |
| C | -4.51459300 | 3.85728100 | -2.00887700 |
| C | -1.99071100 | 3.33158200 | -1.55356300 |
| C | -6.24669400 | -0.34870800 | 1.60395000 |
| C | -6.97073800 | -2.43208900 | 0.69760800 |
| H | -5.43848700 | -3.64726100 | -0.10840300 |

| | | | |
|---|---|---|---|
| H | -2.15686600 | -5.27445900 | 2.07046700 |
| C | -0.86756900 | -4.30977900 | 0.54195000 |
| H | -1.40843600 | -2.99820200 | 3.55983900 |
| H | -0.79849500 | -2.13285300 | 2.14565200 |
| H | -2.12301500 | -1.45217200 | 3.07813300 |
| H | -3.54272700 | -3.99335400 | 3.67880400 |
| H | -4.40345300 | -2.53839500 | 3.15134800 |
| H | -4.69221800 | -4.07299200 | 2.34066600 |
| H | -0.82589900 | -2.64234500 | -0.91318200 |
| H | -1.13111500 | -4.20725300 | -1.63913700 |
| H | -2.84888700 | -5.63491000 | -0.48092800 |
| H | -4.18909500 | -4.94355500 | 0.43681100 |
| H | -3.33342300 | -4.05176100 | -2.62506900 |
| H | -4.59115600 | -3.02138600 | -1.92512000 |
| H | -3.06135800 | -2.31956300 | -2.45521100 |
| C | -3.67582700 | 0.79875000 | 2.79955200 |
| C | -3.11927200 | 3.11080100 | 2.50584100 |
| H | -3.46543100 | 3.89036200 | 0.55312200 |
| H | -6.55020700 | 3.29516200 | -2.76702200 |
| C | -4.84519500 | 2.33655700 | -3.80717100 |
| H | -7.14909700 | 0.63019400 | -2.42134700 |
| H | -5.51828000 | -0.04898500 | -2.60313300 |
| H | -6.37045400 | -0.21641300 | -1.07660900 |
| H | -7.55782300 | 2.52752700 | -0.79395600 |
| H | -6.69352400 | 1.67191200 | 0.47715100 |
| H | -6.22762100 | 3.31806300 | 0.06013700 |
| H | -2.97980000 | 1.21943800 | -3.34893300 |
| H | -2.71940400 | 2.86388000 | -3.92062600 |
| H | -4.23824300 | 4.63674200 | -2.72842300 |
| H | -4.80871600 | 4.34689400 | -1.07731400 |
| H | -1.80219000 | 4.19079700 | -2.20837400 |
| H | -1.82913300 | 3.66375000 | -0.52872700 |
| H | -1.22864200 | 2.58178700 | -1.77084900 |
| H | -6.46739700 | 0.57302900 | 2.13151900 |
| C | -7.27043300 | -1.26216900 | 1.38368400 |
| H | -7.74346000 | -3.15881600 | 0.46402300 |
| H | -0.12612300 | -3.73545200 | 1.09874900 |
| H | -0.42106600 | -5.28570300 | 0.32015300 |
| H | -3.80692300 | -0.05760500 | 3.45271100 |
| C | -3.16522400 | 1.98534400 | 3.32133800 |
| H | -2.77914200 | 4.06642400 | 2.89306800 |
| H | -5.25075400 | 1.38554500 | -4.15848200 |
| H | -4.94003500 | 3.05154400 | -4.63234600 |
| H | -8.27806000 | -1.04930300 | 1.72787300 |
| H | -2.85113300 | 2.03463700 | 4.35965700 |

8. **X-ray details**

BIFOXSiCl$_2$ **7**

| | | |
|---|---|---|
| Empirical formula | C41 H50.50 Cl2 O2 Si | |
| Moiety formula | C32 H40 Cl2 O2 Si, 3/4(C6 H14) | |
| Formula weight | 674.30 | |
| Temperature | 0(2) K | |
| Wavelength | 1.54178 Å | |
| Crystal system | Orthorhombic | |
| Space group | $P2_12_12_1$ | |
| Unit cell dimensions | a = 10.4216(14) Å | $\alpha$= 90°. |
| | b = 16.463(2) Å | $\beta$= 90°. |
| | c = 18.727(2) Å | $\gamma$ = 90°. |
| Volume | 3213.0(7) Å$^3$ | |
| Z | 4 | |
| Density (calculated) | 1.394 Mg/m$^3$ | |
| Absorption coefficient | 2.462 mm$^{-1}$ | |
| F(000) | 1442 | |
| Crystal size | 0.200 x 0.200 x 0.200 mm$^3$ | |
| Theta range for data collection | 3.575 to 72.283°. | |
| Index ranges | -6<=h<=12, -20<=k<=20, -23<=l<=19 | |
| Reflections collected | 13374 | |
| Independent reflections | 5946 [R(int) = 0.0239] | |
| Completeness to theta = 67.679° | 97.9 % | |
| Absorption correction | Semi-empirical from equivalents | |
| Max. and min. transmission | 0.7536 and 0.5292 | |
| Refinement method | Full-matrix least-squares on F$^2$ | |
| Data / restraints / parameters | 5946 / 0 / 340 | |
| Goodness-of-fit on F$^2$ | 0.558 | |
| Final R indices [I>2sigma(I)] | R1 = 0.0286, wR2 = 0.0862 | |
| R indices (all data) | R1 = 0.0295, wR2 = 0.0892 | |
| Absolute structure parameter | 0.041(5) | |
| Extinction coefficient | n/a | |
| Largest diff. peak and hole | 0.234 and -0.478 e.Å$^{-3}$ | |

BIFOXSiCl(OH) **8**

| | |
|---|---|
| Empirical formula | C36.50 H51.50 Cl O3 Si |
| Moiety formula | C32 H41 Cl O3 Si, 3/4(C6 H14) |
| Formula weight | 601.81 |

| | | |
|---|---|---|
| Temperature | 100(2) K | |
| Wavelength | 1.54178 Å | |
| Crystal system | Orthorhombic | |
| Space group | $P2_12_12_1$ | |
| Unit cell dimensions | a = 10.4969(12) Å | $\alpha$= 90°. |
| | b = 15.9441(16) Å | $\beta$= 90°. |
| | c = 18.801(3) Å | $\gamma$ = 90°. |
| Volume | 3146.6(7) $Å^3$ | |
| Z | 4 | |
| Density (calculated) | 1.270 $Mg/m^3$ | |
| Absorption coefficient | 1.708 $mm^{-1}$ | |
| F(000) | 1302 | |
| Crystal size | 0.300 x 0.250 x 0.200 $mm^3$ | |
| Theta range for data collection | 3.635 to 72.173°. | |
| Index ranges | -12<=h<=9, -15<=k<=19, -22<=l<=21 | |
| Reflections collected | 12083 | |
| Independent reflections | 5493 [R(int) = 0.0408] | |
| Completeness to theta = 67.679° | 98.8 % | |
| Absorption correction | Semi-empirical from equivalents | |
| Max. and min. transmission | 0.7535 and 0.5042 | |
| Refinement method | Full-matrix least-squares on $F^2$ | |
| Data / restraints / parameters | 5493 / 0 / 340 | |
| Goodness-of-fit on $F^2$ | 1.046 | |
| Final R indices [I>2sigma(I)] | R1 = 0.0335, wR2 = 0.0846 | |
| R indices (all data) | R1 = 0.0348, wR2 = 0.0855 | |
| Absolute structure parameter | 0.091(8) | |
| Extinction coefficient | n/a | |
| Largest diff. peak and hole | 0.283 and -0.409 e.$Å^{-3}$ | |

BIFOXSi$(OH)_2$ **9** in toluene

| | | |
|---|---|---|
| Empirical formula | C32 H42 O4 Si | |
| Moiety formula | C32 H42 O4 Si | |
| Formula weight | 518.74 | |
| Temperature | 100(2) K | |
| Wavelength | 1.54178 Å | |
| Crystal system | Monoclinic | |
| Space group | $P2_1/c$ | |
| Unit cell dimensions | a = 15.3371(5) Å | $\alpha$= 90°. |

| | | |
|---|---|---|
| | b = 12.8277(5) Å | β= 107.4350(10)°. |
| | c = 14.6917(5) Å | γ = 90°. |
| Volume | 2757.65(17) Å$^3$ | |
| Z | 4 | |
| Density (calculated) | 1.249 Mg/m$^3$ | |
| Absorption coefficient | 1.028 mm$^{-1}$ | |
| F(000) | 1120 | |
| Crystal size | 0.200 x 0.200 x 0.100 mm$^3$ | |
| Theta range for data collection | 4.584 to 72.350°. | |
| Index ranges | -18<=h<=18, -15<=k<=15, -18<=l<=17 | |
| Reflections collected | 36980 | |
| Independent reflections | 5435 [R(int) = 0.0233] | |
| Completeness to theta = 67.679° | 99.8 % | |
| Absorption correction | Semi-empirical from equivalents | |
| Max. and min. transmission | 0.7536 and 0.6518 | |
| Refinement method | Full-matrix least-squares on F$^2$ | |
| Data / restraints / parameters | 5435 / 0 / 348 | |
| Goodness-of-fit on F$^2$ | 1.047 | |
| Final R indices [I>2sigma(I)] | R1 = 0.0337, wR2 = 0.0841 | |
| R indices (all data) | R1 = 0.0344, wR2 = 0.0847 | |
| Extinction coefficient | n/a | |
| Largest diff. peak and hole | 0.318 and -0.401 e.Å$^{-3}$ | |

BIFOXSi(OH)$_2$ **9** in hexane

| | | |
|---|---|---|
| Empirical formula | C131 H175 O16 Si4 | |
| Moiety formula | 4(C32 H42 O4 Si), 0.5(C6 H14) | |
| Formula weight | 2118.06 | |
| Temperature | 100(2) K | |
| Wavelength | 1.54178 Å | |
| Crystal system | Monoclinic | |
| Space group | C2 | |
| Unit cell dimensions | a = 29.2834(10) Å | α= 90°. |
| | b = 12.5929(4) Å | β= 113.8850(10)°. |
| | c = 34.1671(11) Å | γ = 90°. |
| Volume | 11520.5(7) Å$^3$ | |
| Z | 4 | |
| Density (calculated) | 1.221 Mg/m$^3$ | |
| Absorption coefficient | 0.994 mm$^{-1}$ | |

| | |
|---|---|
| F(000) | 4580 |
| Crystal size | 0.200 x 0.150 x 0.100 $mm^3$ |
| Theta range for data collection | 3.301 to 72.230°. |
| Index ranges | -36<=h<=36, -15<=k<=14, -39<=l<=42 |
| Reflections collected | 69567 |
| Independent reflections | 22299 [R(int) = 0.0325] |
| Completeness to theta = 67.679° | 99.9 % |
| Absorption correction | Semi-empirical from equivalents |
| Max. and min. transmission | 0.7536 and 0.6262 |
| Refinement method | Full-matrix least-squares on $F^2$ |
| Data / restraints / parameters | 22299 / 1 / 1417 |
| Goodness-of-fit on $F^2$ | 1.031 |
| Final R indices [I>2sigma(I)] | R1 = 0.0327, wR2 = 0.0841 |
| R indices (all data) | R1 = 0.0339, wR2 = 0.0851 |
| Absolute structure parameter | 0.028(4) |
| Extinction coefficient | n/a |
| Largest diff. peak and hole | 0.391 and -0.353 e.$Å^{-3}$ |

BIFOXSi$(OH)_2$ **9** in acetone

| | | |
|---|---|---|
| Empirical formula | C35 H48 O5 Si | |
| Moiety formula | C32 H42 O4 Si, C3 H6 O | |
| Formula weight | 576.82 | |
| Temperature | 100(2) K | |
| Wavelength | 1.54178 Å | |
| Crystal system | Tetragonal | |
| Space group | $P4_12_12$ | |
| Unit cell dimensions | a = 12.8327(4) Å | $\alpha$= 90°. |
| | b = 12.8327(4) Å | $\beta$= 90°. |
| | c = 37.7649(11) Å | $\gamma$ = 90°. |
| Volume | 6219.1(4) $Å^3$ | |
| Z | 8 | |
| Density (calculated) | 1.232 $Mg/m^3$ | |
| Absorption coefficient | 0.986 $mm^{-1}$ | |
| F(000) | 2496 | |
| Crystal size | 0.200 x 0.200 x 0.100 $mm^3$ | |
| Theta range for data collection | 3.638 to 74.528°. | |
| Index ranges | -13<=h<=15, -16<=k<=13, -47<=l<=46 | |
| Reflections collected | 51773 | |

| | |
|---|---|
| Independent reflections | 6351 [R(int) = 0.0298] |
| Completeness to theta = 67.679° | 99.9 % |
| Absorption correction | Semi-empirical from equivalents |
| Max. and min. transmission | 0.7538 and 0.6308 |
| Refinement method | Full-matrix least-squares on $F^2$ |
| Data / restraints / parameters | 6351 / 0 / 410 |
| Goodness-of-fit on $F^2$ | 1.086 |
| Final R indices [I>2sigma(I)] | R1 = 0.0306, wR2 = 0.0799 |
| R indices (all data) | R1 = 0.0318, wR2 = 0.0801 |
| Absolute structure parameter | 0.032(4) |
| Extinction coefficient | n/a |
| Largest diff. peak and hole | 0.222 and -0.324 e.Å$^{-3}$ |

BIFOXSiCl(OH) **8** in acetone

| | | |
|---|---|---|
| Empirical formula | C35 H47 Cl O4 Si | |
| Moiety formula | C32 H41 Cl O3 Si, C3 H6 O | |
| Formula weight | 595.26 | |
| Temperature | 100(2) K | |
| Wavelength | 1.54178 Å | |
| Crystal system | Orthorhombic | |
| Space group | $P2_12_12_1$ | |
| Unit cell dimensions | a = 10.5180(16) Å | $\alpha$= 90°. |
| | b = 16.3280(13) Å | $\beta$= 90°. |
| | c = 18.1007(18) Å | $\gamma$ = 90°. |
| Volume | 3108.6(6) Å$^3$ | |
| Z | 4 | |
| Density (calculated) | 1.272 Mg/m$^3$ | |
| Absorption coefficient | 1.750 mm$^{-1}$ | |
| F(000) | 1280 | |
| Crystal size | 0.300 x 0.200 x 0.100 mm$^3$ | |
| Theta range for data collection | 3.646 to 72.159°. | |
| Index ranges | -12<=h<=12, -18<=k<=20, -21<=l<=22 | |
| Reflections collected | 23558 | |
| Independent reflections | 6088 [R(int) = 0.0407] | |
| Completeness to theta = 67.679° | 99.9 % | |
| Absorption correction | Semi-empirical from equivalents | |
| Max. and min. transmission | 0.7536 and 0.5440 | |
| Refinement method | Full-matrix least-squares on $F^2$ | |

| | |
|---|---|
| Data / restraints / parameters | 6088 / 1 / 382 |
| Goodness-of-fit on $F^2$ | 1.020 |
| Final R indices [I>2sigma(I)] | R1 = 0.0303, wR2 = 0.0733 |
| R indices (all data) | R1 = 0.0316, wR2 = 0.0742 |
| Absolute structure parameter | 0.051(6) |
| Extinction coefficient | n/a |
| Largest diff. peak and hole | 0.237 and -0.356 e.Å$^{-3}$ |

## 9. Selected NMR spectra

### a. BIFOXSiCl$_2$

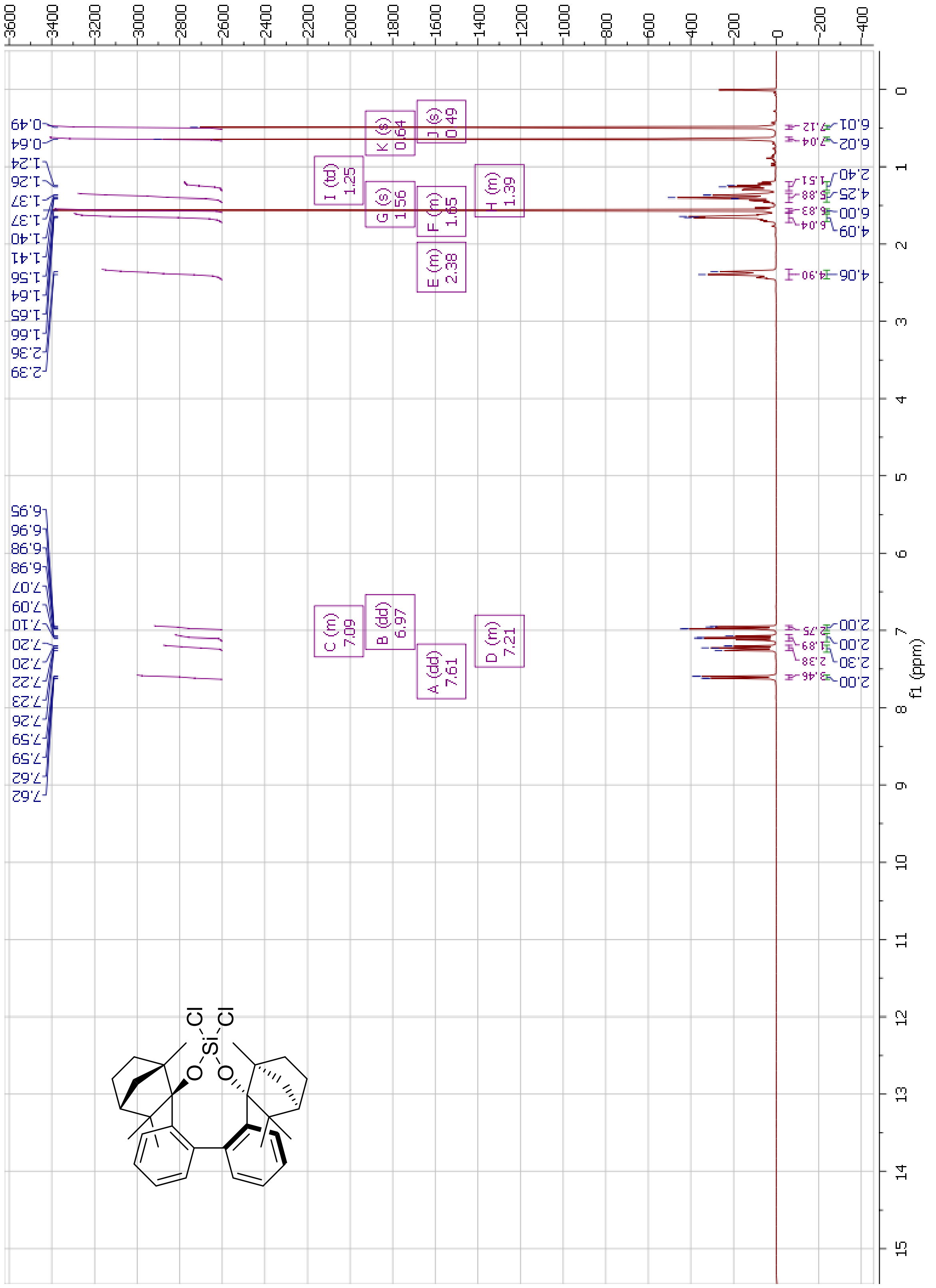

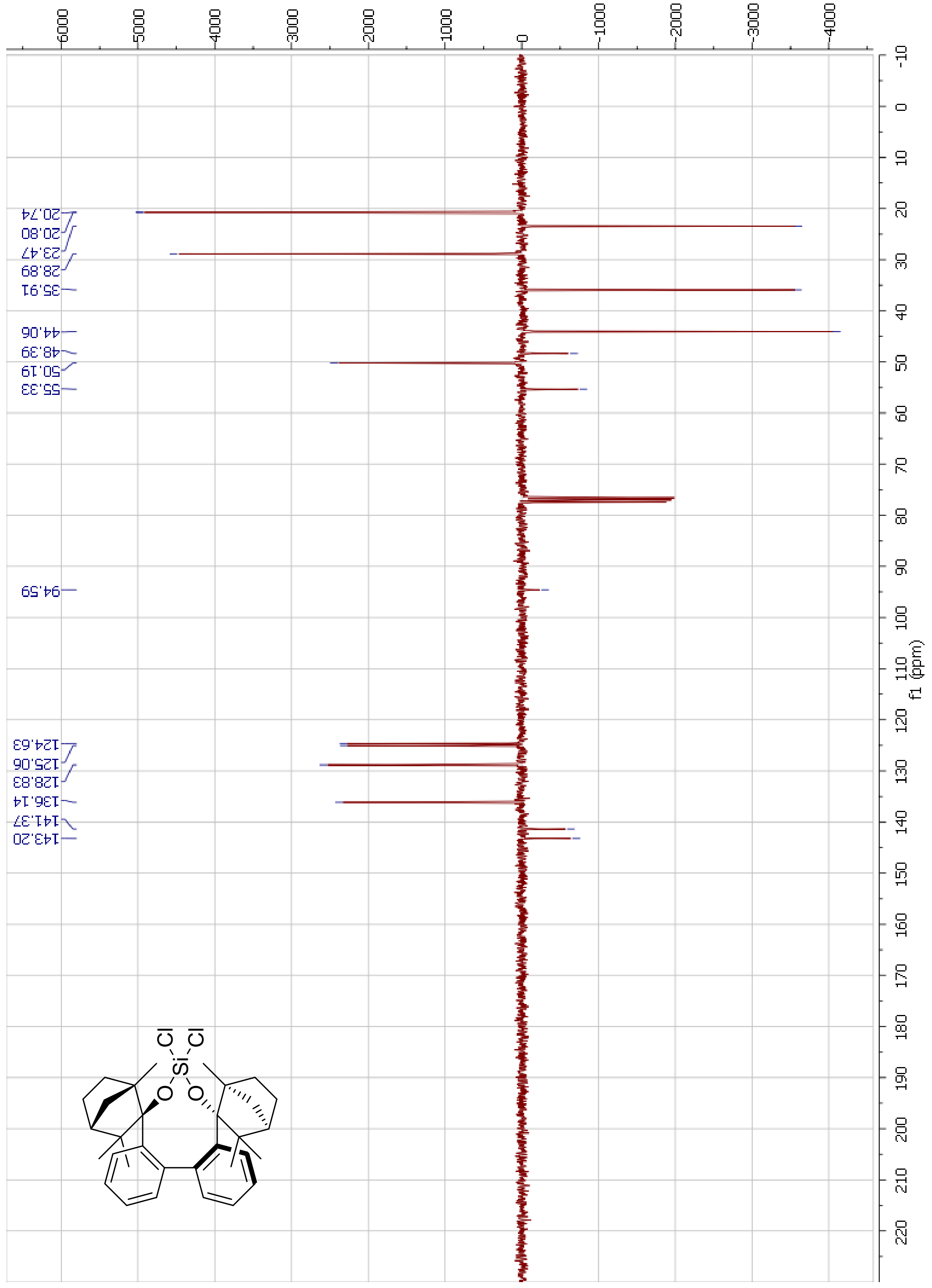
20.74
20.80
23.47
28.89
35.91
44.06
48.39
50.19
55.33
94.59
124.63
125.06
128.83
136.14
141.37
143.20
f1 (ppm)
Cl
Cl
Si
O
O

**b. BIFOXSiCl(OH) 8**

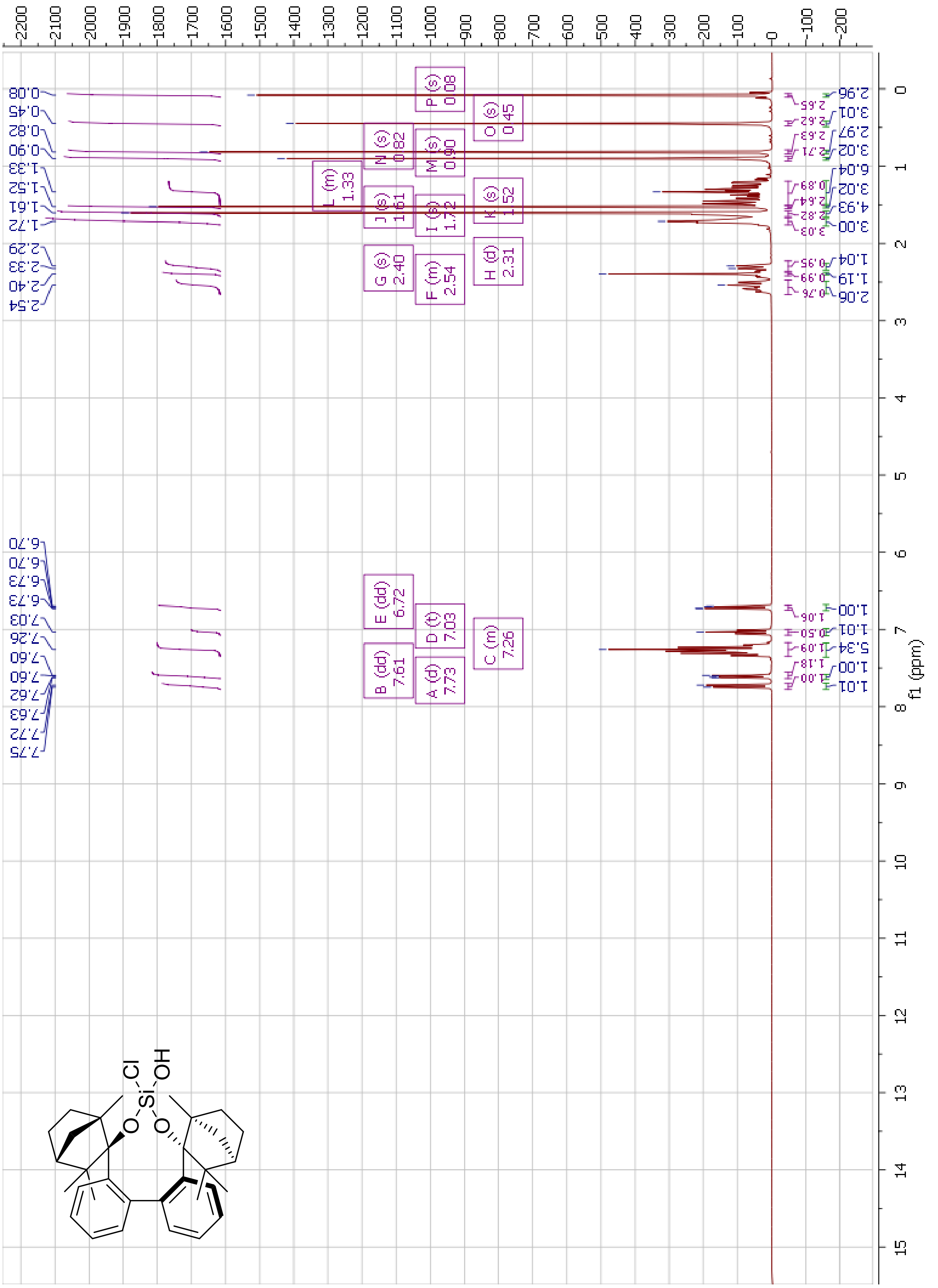

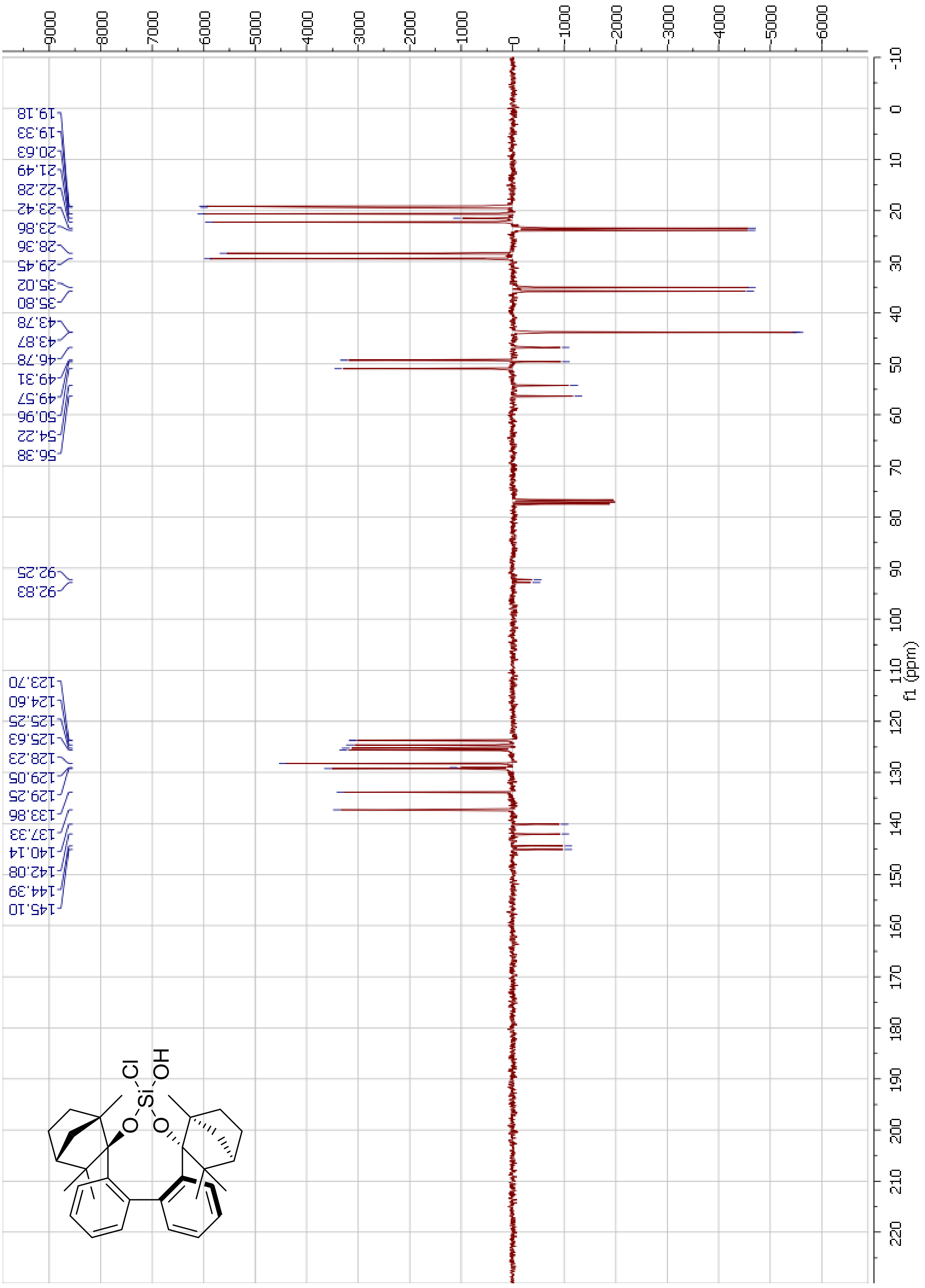
19.18
19.33
20.63
21.49
22.28
23.42
23.86
28.36
29.45
35.02
35.80
43.78
43.87
46.78
49.31
49.57
50.96
54.22
56.38
92.25
92.83
123.70
124.60
125.25
125.63
128.23
129.05
129.25
133.86
137.33
140.14
142.08
144.39
145.10
f1 (ppm)
Cl
Si
OH
O
O

## c. BIFOXSi(OH)$_2$ 9

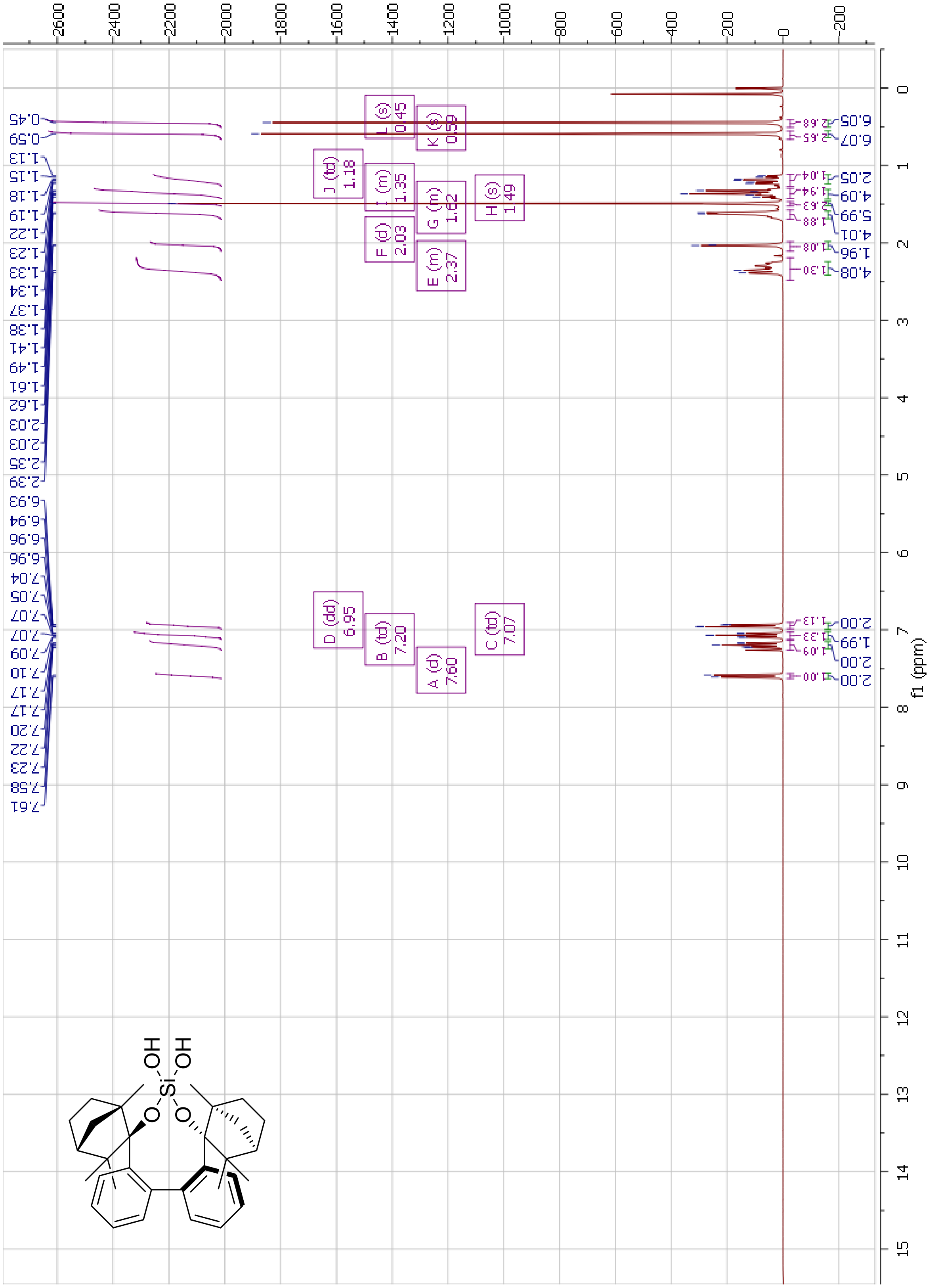

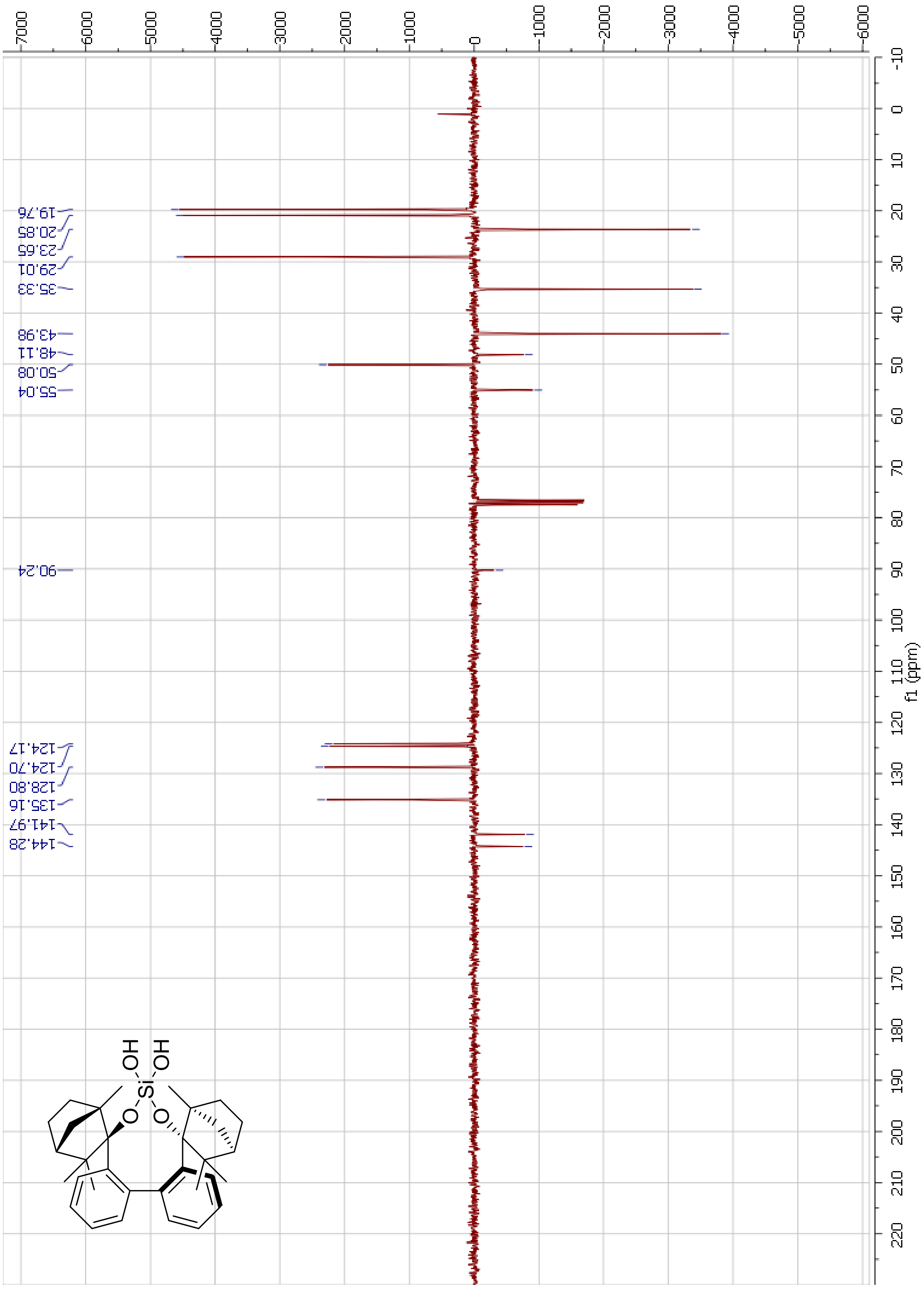

19.76
20.85
23.65
29.01
35.33
43.98
48.11
50.08
55.04
90.24
124.17
124.70
128.80
135.16
141.97
144.28
f1 (ppm)

**d. Kondo-$SiCl_2$ 13**

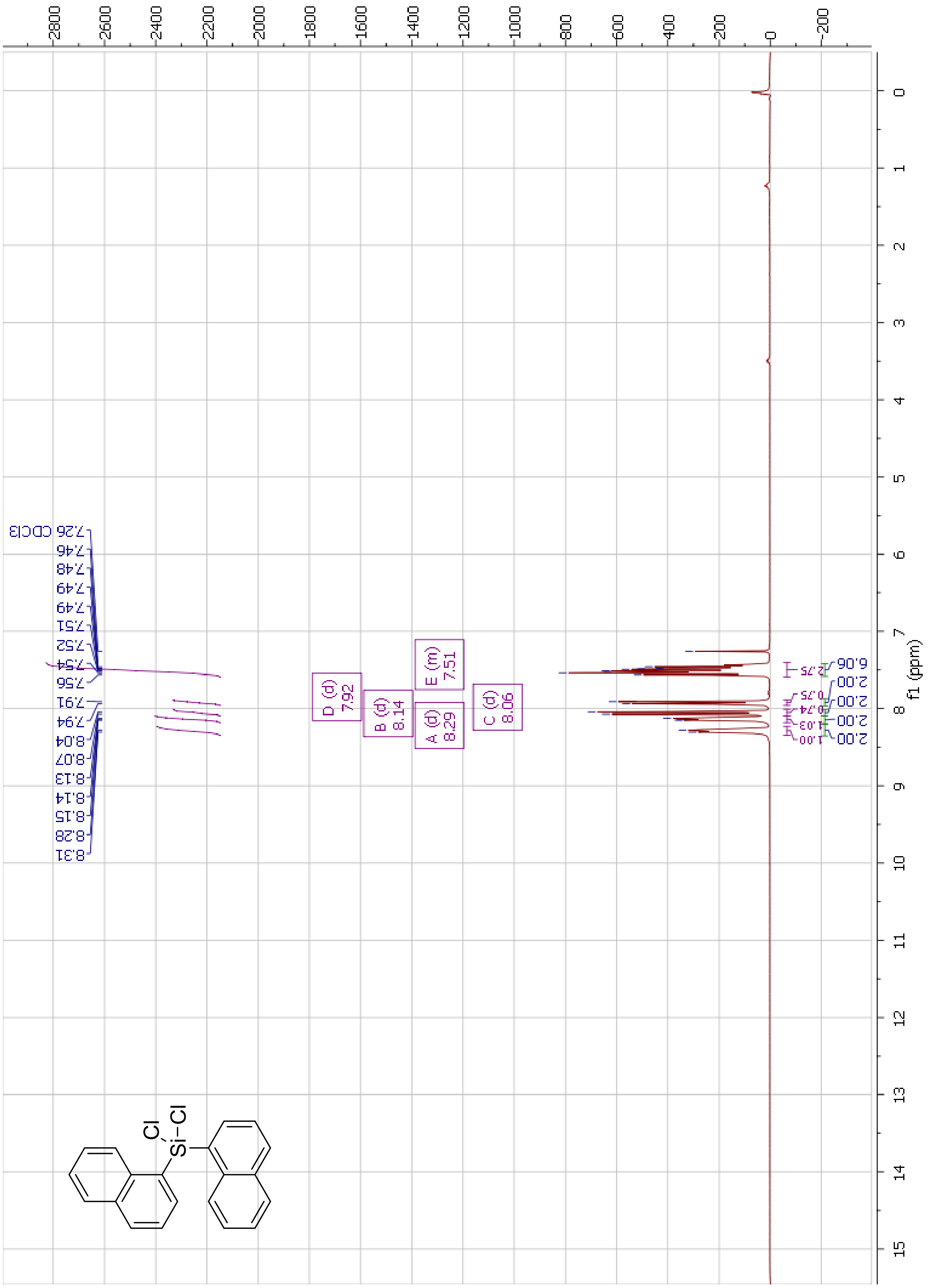

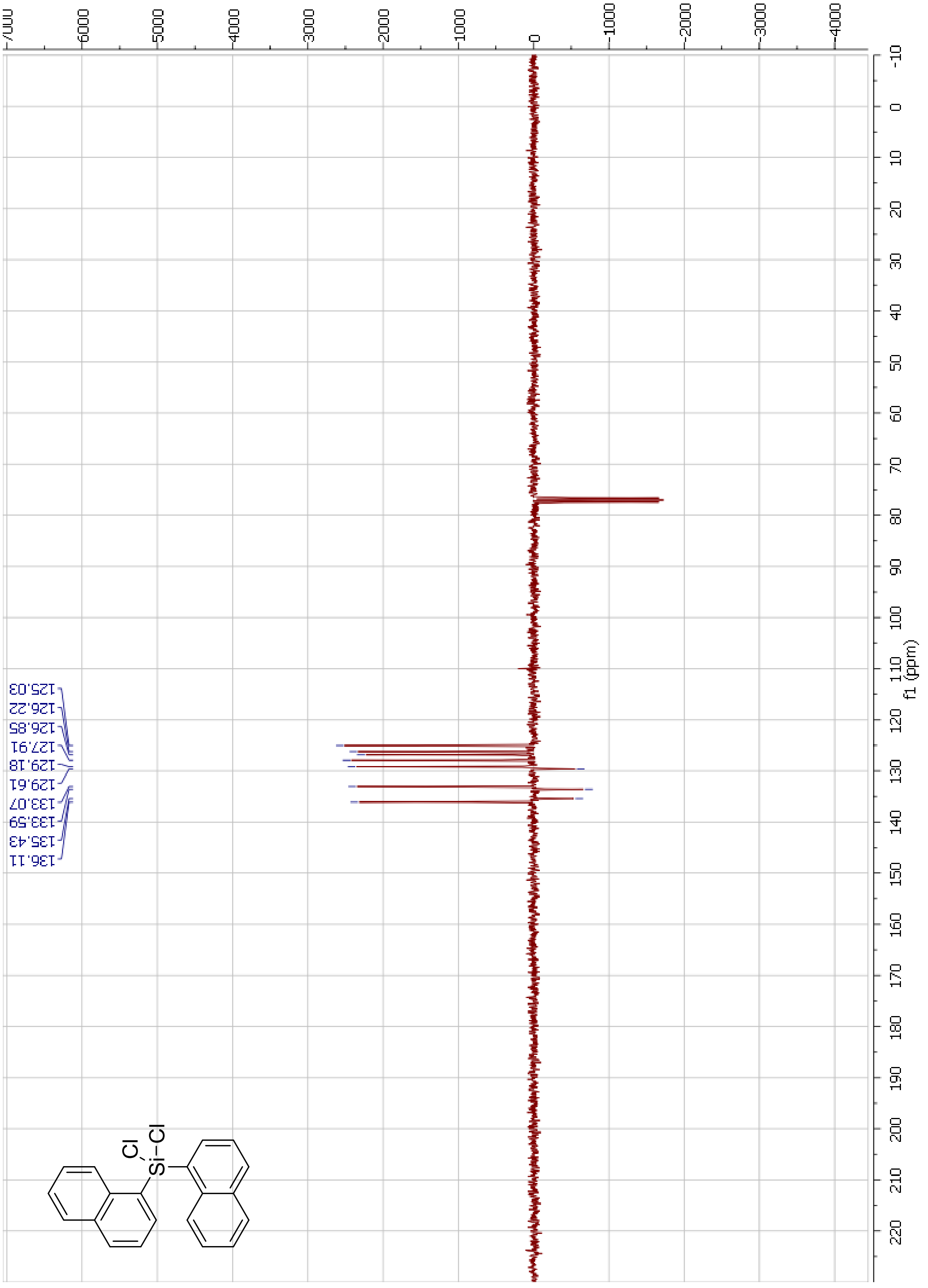

125.03
126.22
126.85
127.91
129.18
129.61
133.07
133.59
135.43
136.11
f1 (ppm)
Cl
Si–Cl

## e. KondoSi(OH)$_2$ 1

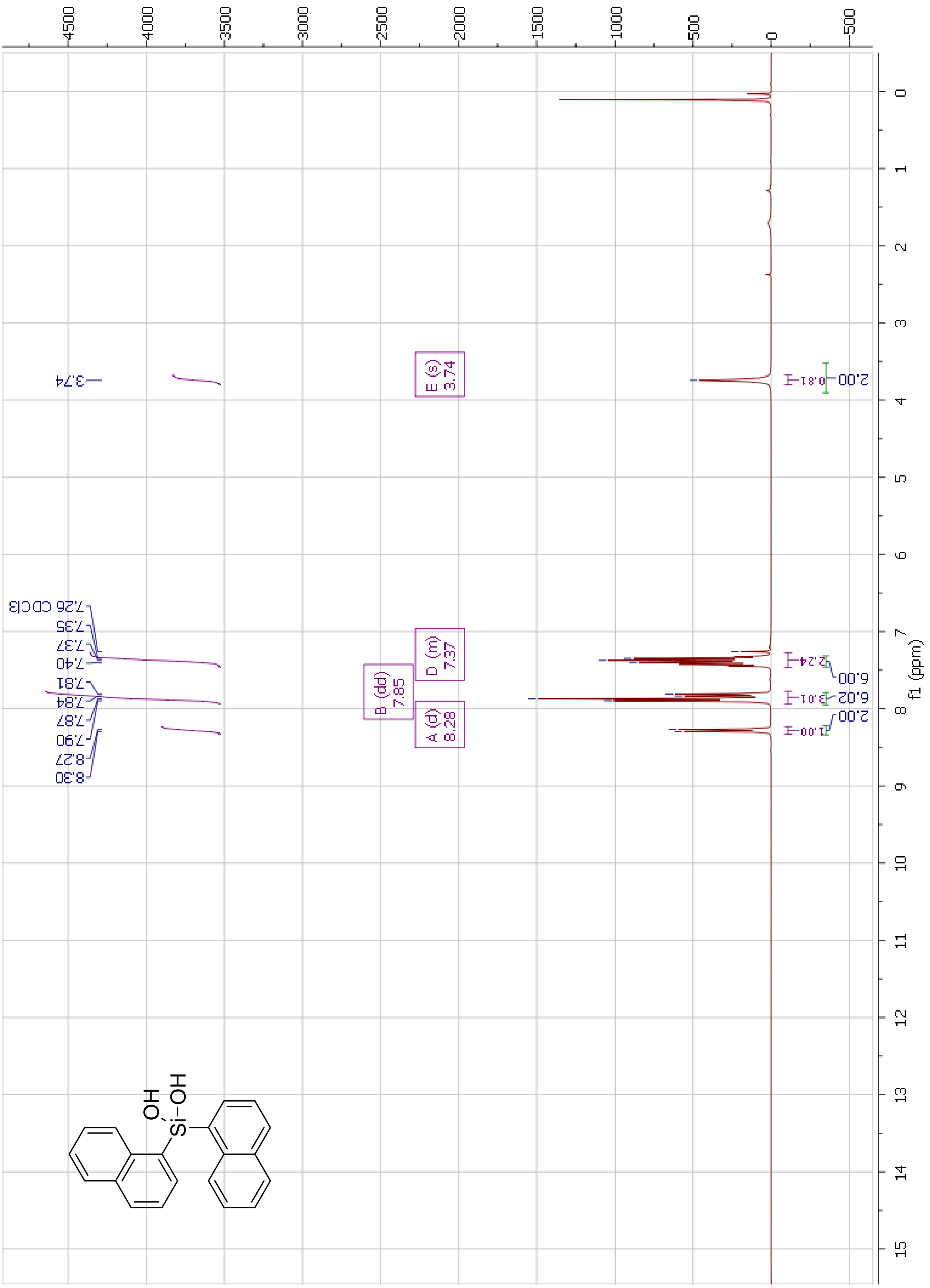

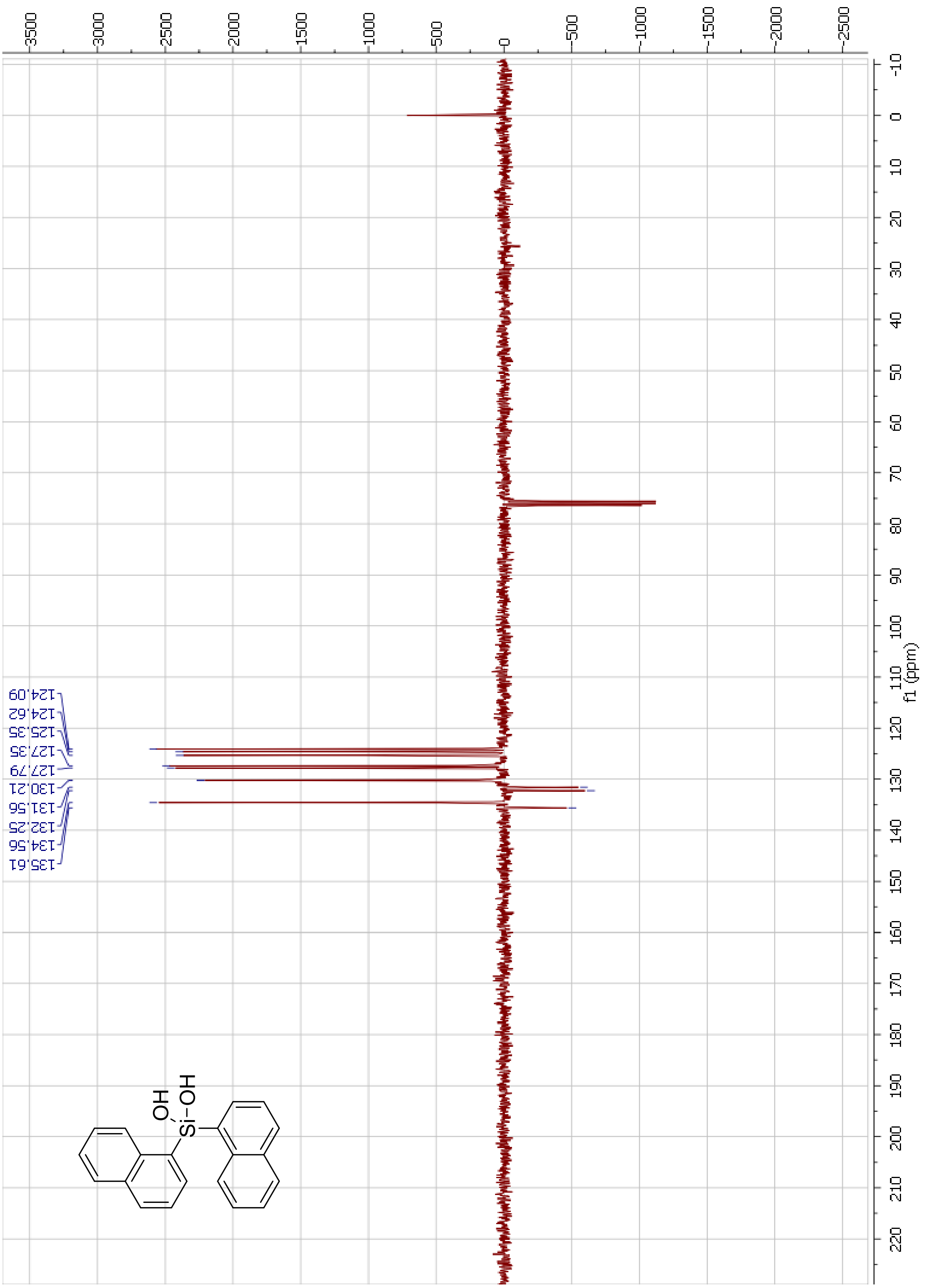
135.61
134.56
132.25
131.56
130.21
127.79
127.35
125.35
124.62
124.09
f1 (ppm)
Si
OH
OH

## f. dichlorobis(2,4,6-tri-*tert*-butylphenoxy)silane 14

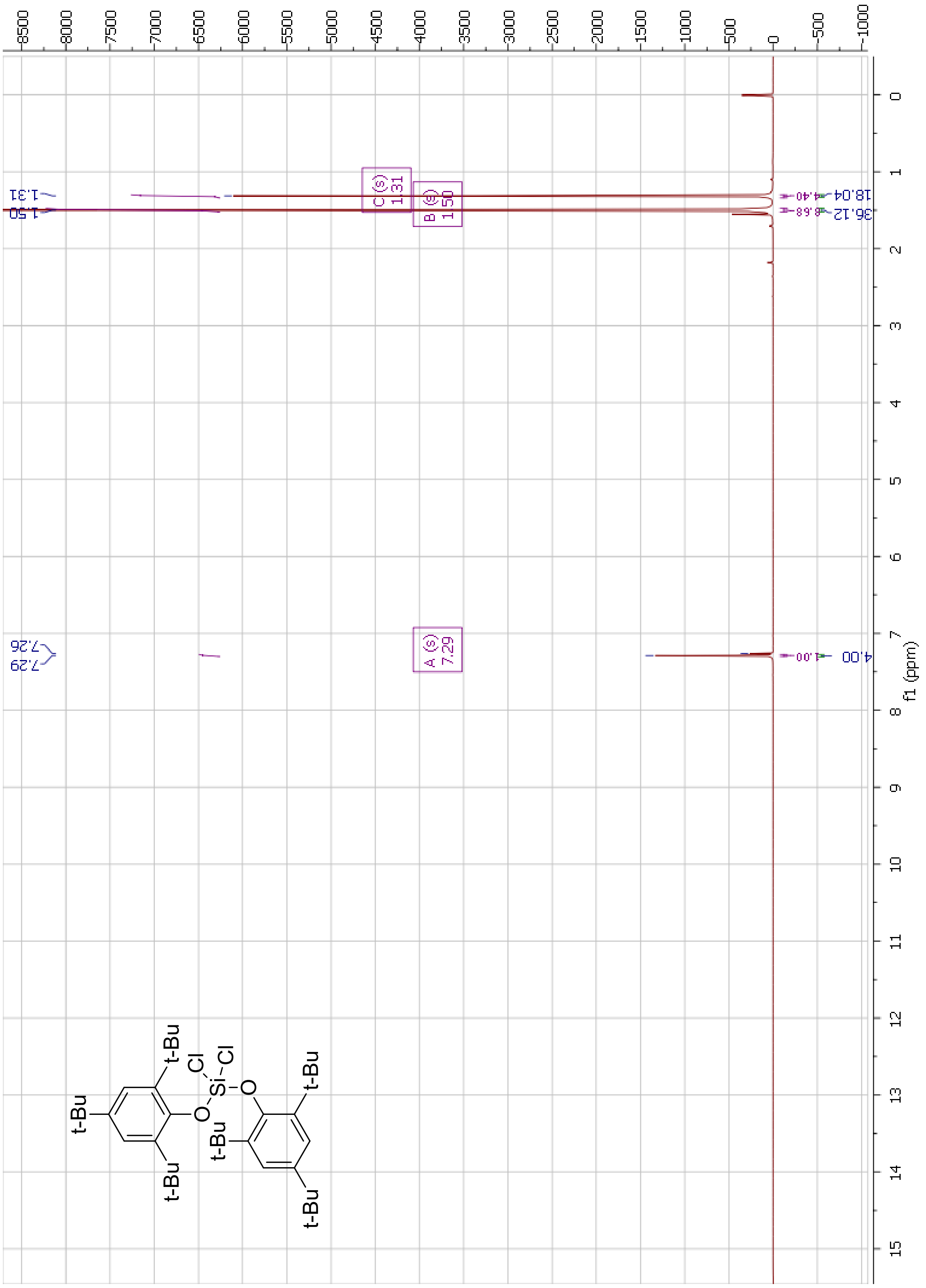

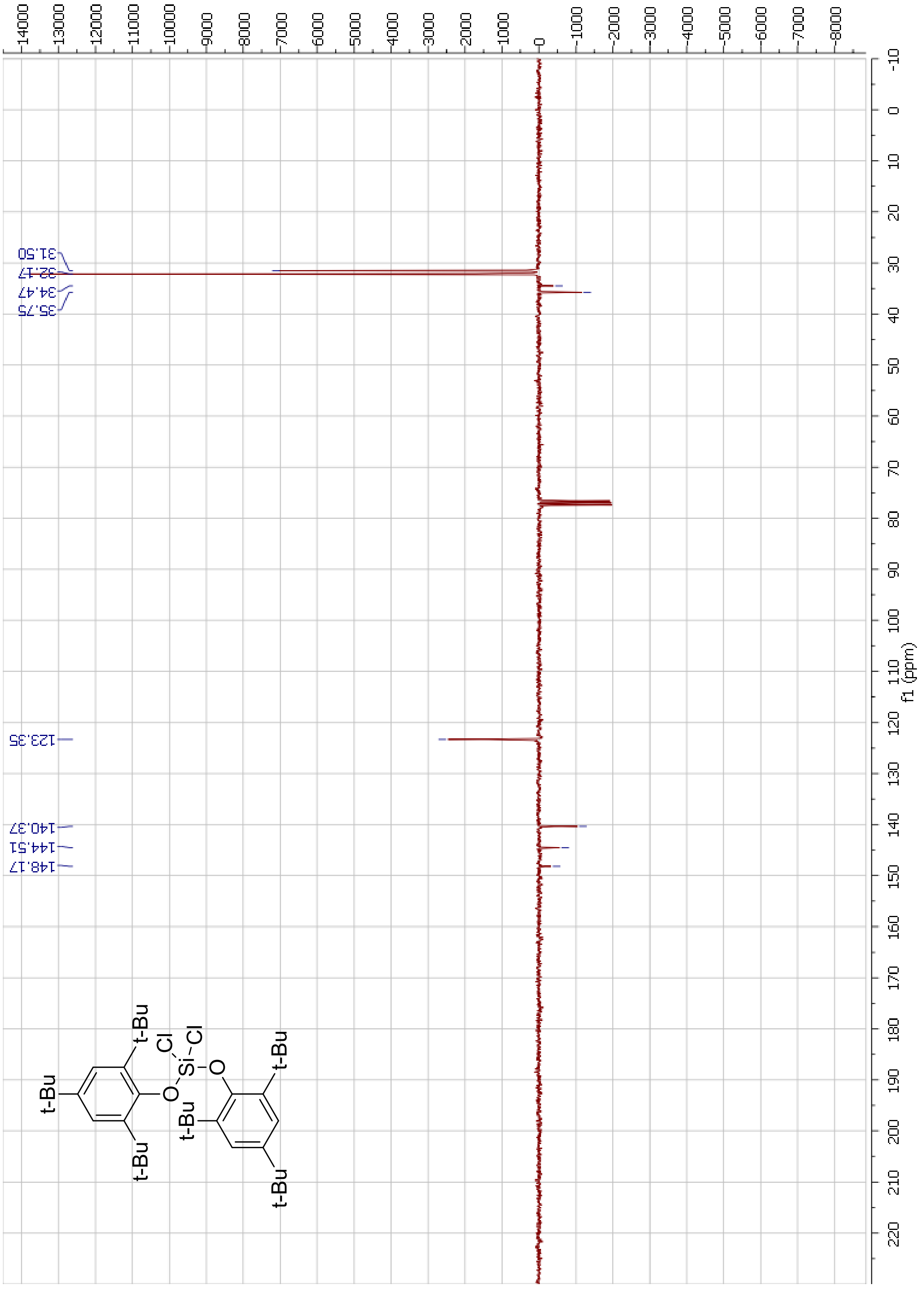
31.50
32.17
34.47
35.75
123.35
140.37
144.51
148.17
f1 (ppm)
t-Bu
Cl
Si
Cl
O
O
t-Bu
t-Bu
t-Bu
t-Bu
t-Bu

## g. bis(2,4,6-tri-*tert*-butylphenoxy)silandiol 15

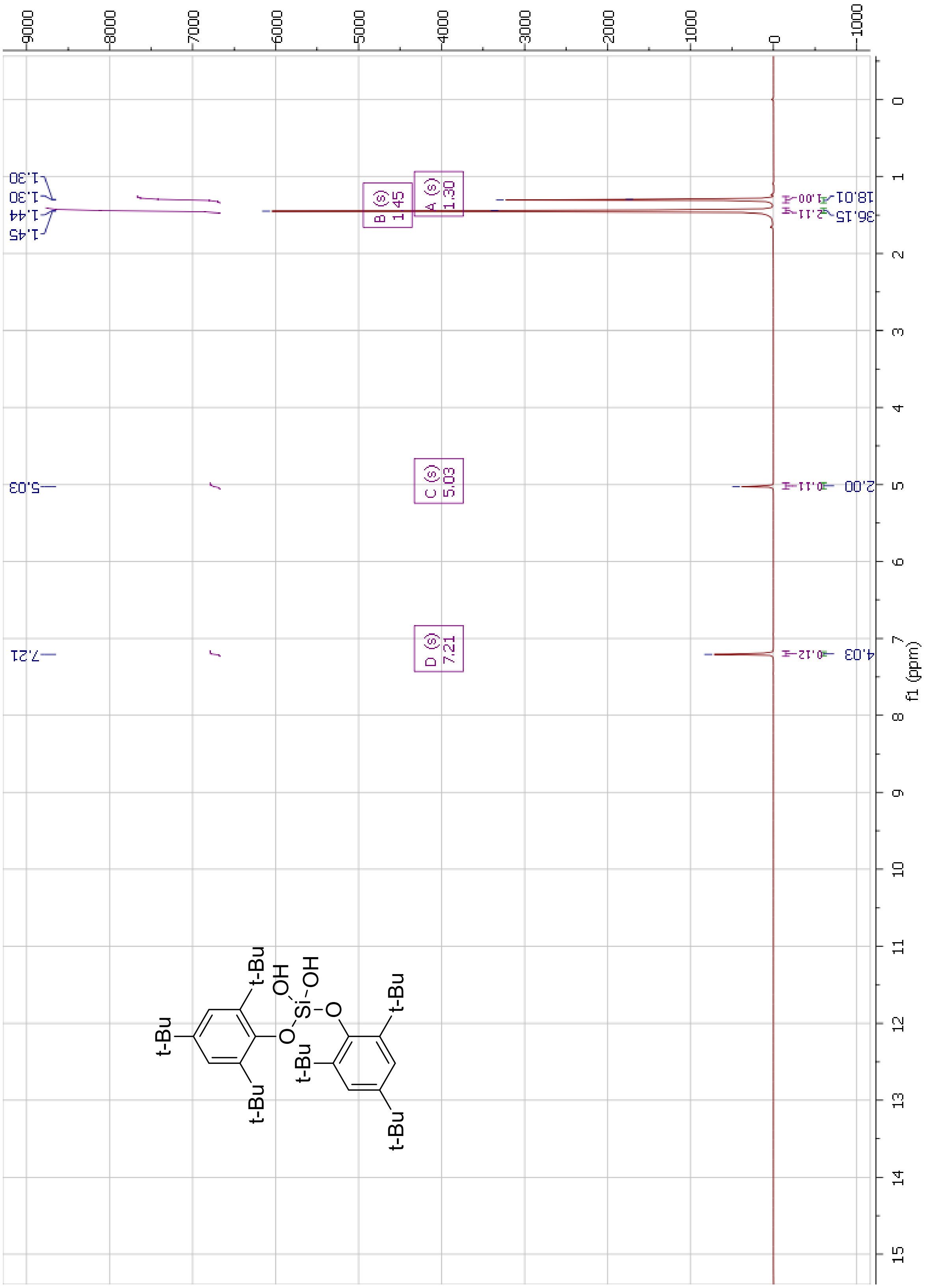

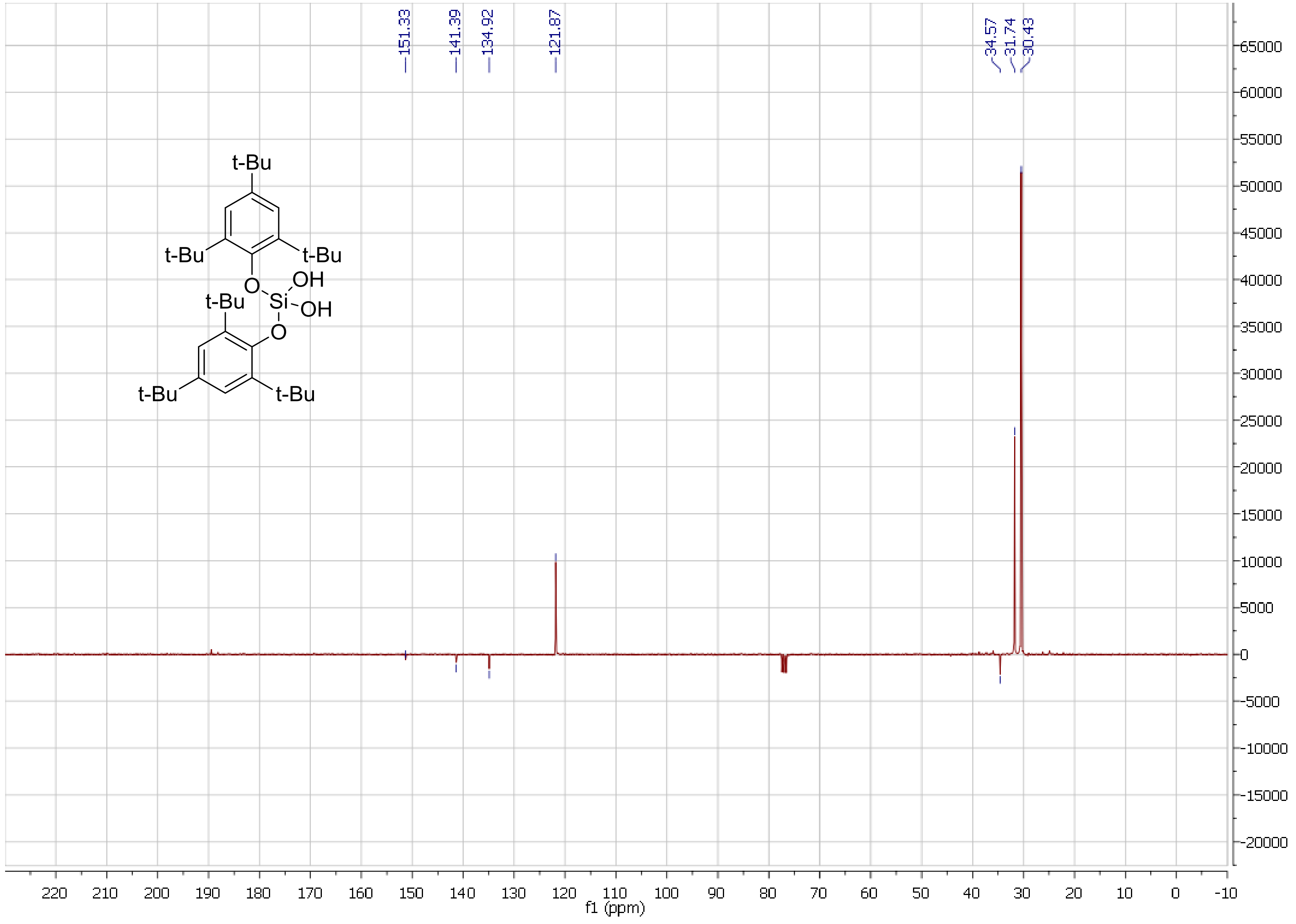

151.33
141.39
134.92
121.87
34.57
31.74
30.43
t-Bu
O
Si
OH
f1 (ppm)
220 210 200 190 180 170 160 150 140 130 120 110 100 90 80 70 60 50 40 30 20 10 0 -10
65000 60000 55000 50000 45000 40000 35000 30000 25000 20000 15000 10000 5000 0 -5000 -10000 -15000 -20000

## 10. References


[1] a) G. Schott, L. Engelbrecht, H. J. Holdt, *Z. anorg. allg. Chem.* **1979**(459), 177–186; b) J. Beckmann, D. Dakternieks, A. Duthie, M. L. Larchin, E. R. T. Tiekink, *Appl. Organometal. Chem.* **2003**, *17*(1), 52–62;

[2] a) A. G. Schafer, J. M. Wieting, T. J. Fisher, A. E. Mattson, *Angew. Chem. Int. Ed.* **2013**, *52*(43), 11321–11324; b) M. S. Taylor, N. Tokunaga, E. N. Jacobsen, *Angew. Chem. Int. Ed.* **2005**, *44*(41), 6700–6704;

[3] S. E. Reisman, A. G. Doyle, E. N. Jacobsen, *J. Am. Chem. Soc.* **2008**, *130*(23), 7198–7199.

[4] A. M. Hardman-Baldwin, M. D. Visco, J. M. Wieting, C. Stern, S.-I. Kondo, A. E. Mattson, *Org. Lett.* **2016**, *18*(15), 3766–3769.

[5] M. J. Frisch, G. W. Trucks, H. B. Schlegel, G. E. Scuseria, M. A. Robb, J. R. Cheeseman, G. Scalmani, V. Barone, B. Mennucci, G. A. Petersson, H. Nakatsuji, M. Caricato, X. Li, H. P. Hratchian, A. F. Izmaylov, J. Bloino, G. Zheng, J. L. Sonnenberg, M. Hada, M. Ehara, K. Toyota, R. Fukuda, J. Hasegawa, M. Ishida, T. Nakajima, Y. Honda, O. Kitao, H. Nakai, T. Vreven, J. A. Montgomery, Jr, J. E. Peralta, F. Ogliaro, M. Bearpark, J. J. Heyd, E. Brothers, K. N. Kudin, V. N. Staroverov, T. Keith, R. Kobayashi, J. Normand, K. Raghavachari, A. Rendell, J. C. Burant, S. S. Iyengar, J. Tomasi, M. Cossi, N. Rega, J. M. Millam, M. Klene, J. E. Knox, J. B. Cross, V. Bakken, C. Adamo, J. Jaramillo, R. Gomperts, R. E. Stratmann, O. Yazyev, A. J. Austin, R. Cammi, C. Pomelli, J. W. Ochterski, R. L. Martin, K. Morokuma, V. G. Zakrzewski, G. A. Voth, P. Salvador, J. J. Dannenberg, S. Dapprich, A. D. Daniels, O. Farkas, J. B. Foresman, J. V. Ortiz, J. Cioslowski, and D. J. Fox, *Gaussian 09 Rev. D.01*, Gaussian, Inc, Wallingford CT, **2013**.

[6] NIST Computational Chemistry Comparison and Benchmark Database, "NIST Standard Reference Database Number 101", to be found under http://cccbdb.nist.gov/, **Release 18, 2016**.
